# Prehospital evaluation and economic analysis of different coronary syndrome treatment strategies - PREDICT - Rationale, Development and Implementation

**DOI:** 10.1186/1471-227X-11-4

**Published:** 2011-03-29

**Authors:** Laurie J Morrison, Valeria E Rac, James M Bowen, Brian Schwartz, Tyrone Perreira, Welson Ryan, Cathy Zahn, Rishab Chadha, Alan Craig, Daria O'Reilly, Ron Goeree

**Affiliations:** 1 Rescue, Keenan Research Centre, Li Ka Shing Knowledge Institute, St. Michael's Hospital, University of Toronto, Toronto, Ontario, Canada; 2 Division of Emergency Medicine; Department of Medicine, University of Toronto, Toronto, Ontario, Canada; 3 Health Policy, Management & Evaluation, University of Toronto, Toronto, Ontario, Canada; 4 Programs for Assessment of Technology in Health (PATH) Research Institute, St., Joseph's Healthcare Hamilton, Hamilton, Ontario, Canada, Department of Clinical Epidemiology and Biostatistics, Faculty of Health Sciences, McMaster University, Hamilton, Ontario, Canada; 5 Sunnybrook Osler Centre for Prehospital Care, Sunnybrook Health Sciences Centre, Division of Emergency Medicine, Department of Family and Community Medicine, University of Toronto, Toronto Ontario, Canada; 6 Toronto Emergency Medical Services, Canada

## Abstract

**Background:**

A standard of prehospital care for patients presenting with ST-segment elevation myocardial infarction (STEMI) includes prehospital 12-lead and advance Emergency Department notification or prehospital bypass to percutaneous coronary intervention centres. Implementation of either care strategies is variable across communities and neither may exist in some communities. The main objective is to compare prehospital care strategies for time to treatment and survival outcomes as well as cost effectiveness.

**Methods/Design:**

PREDICT is a multicentre, prospective population-based cohort study of all chest pain patients 18 years or older presenting within 30 mins to 6 hours of symptom onset and treated with nitroglycerin, transported by paramedics in a number of different urban and rural regions in Ontario. The primary objective of this study is to compare the proportion of study subjects who receive reperfusion within the target door-to-reperfusion times in subjects obtained after four prehospital strategies: 12-lead ECG and advance emergency department (ED) notification or 3-lead ECG monitoring and alert to dispatch prior to hospital arrival; either with or without the opportunity to bypass to a PCI centre.

**Discussion:**

We anticipate four challenges to successful study implementation and have developed strategies for each: 1) diversity in the interpretation of the ethical and privacy issues across 47 research ethics boards/commiittees covering 71 hospitals, 2) remote oversight of data guardian abstraction, 3) timeliness of implementation, and 4) potential interference in the study by concurrent technological advances. Research ethics approvals from academic centres were obtained initially and submitted to non academic centre applications. Data guardians were trained by a single investigator and data entry is informed by a detailed data dictionary including variable definitions and abstraction instrucations and subjected to error and logic checks. Quality oversight provided by a single investigator. The window of the trial in each community has been confirmed with the basehospital medical director to correspond to the planned technological advances of the system of care. We hope this comparative analysis across treatment strategies for clinical outcomes and cost will provide sufficient evidence to implement the superior strategy across all communities and improve outcomes for all STEMI patients.

**Trial registration:**

ClinicalTrials.gov: NCT00747656

## Background

Cardiovascular disease accounts for more deaths than any other disease and ischemic heart diseases, such as acute myocardial infarction (AMI), account for a large proportion of these deaths [1-3]. Timely recognition and reperfusion are life saving interventions [3-7]. Randomized controlled trials have demonstrated the superiority of prehospital fibrinolysis [8-11]; whereas other interventions such as 12-lead [12-15] and bypass to interventional hospitals [6,16-23] have not been subjected to the same rigorous analysis. Based on associations with improved time to treatment, 12-lead ECG and bypass to an interventional hospital has been implemented in many communities which makes it difficult to conduct a controlled trial. In contrast, many rural and urban communities with small volumes may not have implemented any one of these interventions, as such the standard of care for patients presenting with ST-segment elevation myocardial infarction (STEMI) varies between communities[24]. In 2004, the expert panel of Cardiac Care Network of Ontario report recommended that urgent angioplasty should be adopted as the standard of care[24]. Concurrent with this report the Ontario Health Technology Advisory Committee (OHTAC), upon their review of the literature regarding primary angioplasty for the treatment of STEMI, recommended that "every effort should be made to decrease the access time for patients with AMI from onset to symptoms to administration of fibrinolysis or primary angioplasty"[25,26]. This study is being conducted to evaluate different implementation strategies in place currently that could reduce symptom to intervention time in Ontario[25]. The objective of this paper is to describe the design of a study to compare these strategies against time to treatment and survival outcomes as well as cost effectiveness. We hope that this prospective cohort trial will promote the adoption of the optimal implementation strategy into the healthcare system and may provide the information required to directly change health policy and funding for systematic multidisciplinary care involving local EMS systems. We anticipate that the identification and implementation of the best care strategy may provide consistent and optimal care of patients presenting with STEMI across all communities in Ontario,

## Methods/Design

### Study Design

PREDICT is a prospective, population-based cohort study of four patient care strategies provided by regional EMS services to patients with chest pain and suspected ischemia.

1. 3-lead PHECG and transported to the nearest receiving ED who were not eligible for bypass based on transport time.

2. 3-lead PHECG and transported to the nearest receiving ED who were eligible for bypass based on transport time, if 12 lead PHECG was possible.

3. 12-lead PHECG and prehospital notification transported to the nearest receiving ED who were not eligible for bypass to a PCI center based on transport time.

4. 12-lead PHECG with prehospital notification and eligible for bypassing the nearest receiving ED with transport to a PCI center.

Bypass eligibility was based on transport distance of patients from their pick-up location to PCI center and the cut-off point was 60 kilometres.

### Inclusion and Exclusion Criteria

#### Inclusion criteria

○ Patients who call 911, and are:

○ Suspected by the paramedics to have ischemic chest pain for greater than 30 minutes but less than 6 hours, and

○ 18 years of age or older

○ Experiencing chest pain that fails to resolve with nitrates given as per protocol

#### Exclusion Criteria

○ Age < 18 years of age

### Setting

This study is set in regions of Ontario with a population of 3,043,853 served by 14 EMS services, under the medical control of 4 regional Base Hospital programs (Table 1)[27]. These regions represent 25% of the population of Ontario and 9.6% of the population of Canada. This geographic region covers 206,727 km^2 ^with variable population densities from 0.6 to 574 persons per km^2 ^representing rural, suburban, urban, and metropolis areas[27].

**Table 1:** List of regional base hospital programs and emergency medical services participating in PREDICT study

Regional Basehospital Programs	Participating Emergency Medical Services (EMS)
Hamilton Health Sciences Centre for Paramedic Education & Research - Hamilton	County of Brant Ambulance Service Haldimand EMS Hamilton EMS Hamilton-Wentworth Regional Ambulance Service Norfolk EMS Six Nations Ambulance Service

North-East Ontario Regional Base Hospital Program - Sudbury	Algoma EMS Manitoulin-Sudbury EMS Sudbury EMS Sault Ste. Marie EMS Timmins EMS

Northwest Region Base Hospital Program	Superior North EMS

Sunnybrook Osler Centre for Prehospital Care - Toronto	County of Simcoe Paramedic Services, Peel Regional Paramedic Services

### Sample Size Calculation

The recruitment goal is to enrol 100 STEMI prospective subjects per group (e.g. as in the WEST trial)[28], for a total of 400 STEMI subjects. The primary estimate is based on a difference in the proportion of patients who received reperfusion (fibrinolysis or PCI) within target door-to-intervention times. We based our calculation using estimates from Canto et al., 2002[29]. In that study there was an increase in the percentage of the patients who received lytic therapy within 30 minutes, from 31% to 50% (an absolute difference of 19%). The percentage of patients who received PCI within 90 minutes increased from 29% to 48% (an absolute difference of 19%). Furthermore there was a combined 24% higher odds of receiving fibrinolytic therapy or PCI with the active EMS involvement (odds ratio 1.24, 95% CI 1.21 to 1.28, p < 0.001). Calculations were conducted using PASS software, assuming an alpha (α) of 0.05 and power of 80% (Table 2).

**Table 2:** Sample size calculation for the PREDICT study

Baseline %	Intervention %	Absolute Difference	Odds Ratio	Number per arm
37.4	47.4	10%	1.5	402

37.4	52.4	15%	1.8	185

37.4	57.4	20%	2.3	106

To estimate the number of potential subjects that could be enrolled in the study, the annual rate of STEMIs that would occur within a 60 minute transport time of the closest PCI centre was determined. The surrounding areas within 60 minutes of a PCI centre were first identified using data from a Cardiac Care Network of Ontario (CCN) report published in 2004[6]. Current population estimates were then assigned to each of the surrounding areas using population estimates for 2006[27]. For counties or regions where a proportion of the population resided outside a 60 minute radius, population data from the 2006 Canadian census was used from the census subdivisions to adjust the 2006 population estimates[27]. To determine the rate of STEMI, an estimate of 571 per 1,000,000 inhabitants was calculated by taking an estimate obtained using CIHI data of 6524 STEMIs in Ontario for fiscal 2001/02 and dividing it by the 2006 Ontario Census Population and determining the rate per million inhabitants[6,27]. The number of potential subjects to be entered in per year was then estimated by assuming a 50% transport by EMS rate and a potential recruitment rate of 70%.

### Study Outcomes

#### Primary Outcome

The primary outcome of this study is to compare the proportion of study subjects who receive reperfusion within the target door-to-reperfusion times across the four care strategies. Target door to reperfusion times are 90 minutes for primary PCI intervention (door-to-balloon time) and 30 minutes for fibrinolysis (door-to-needle time)[30-32].

### Secondary Outcomes

#### Survival

Survival at 30 days and one year after episode date (brief telephone assessment) for STEMI patients

### Treatment Time Intervals

• Prehospital scene time interval defined as time from arrival at scene to departure from scene;

• Transport time interval defined as time from departure from scene to arrival at destination hospital;

• Symptom onset time interval defined as time from symptom onset reported by subject to reperfusion intervention (defined as time to drug administration or balloon inflation);

• Primary hospital reperfusion time interval defined as the time from arrival at primary destination hospital to reperfusion intervention at the primary destination (defined as time to drug administration or balloon inflation);

• PCI transfer reperfusion time interval defined as the time from arrival at primary destination hospital and transport to a PCI capable site to the reperfusion intervention at the PCI site (defined as time to drug administration or balloon inflation).

• PCI site reperfusion time interval defined as the time from arrival at PCI site to reperfusion intervention at the PCI site (defined as time to drug administration or balloon inflation)

### STEMI Identification with 12-Lead PHECG

• Proportion of STEMI subjects within the target door-to-reperfusion times comparing basic vs. advanced life support paramedics.

• Paramedics and computer software interpretation of the 3-lead and 12-lead PHECG of all STEMI subjects will be compared with a gold standard (defined as consensus between two investigators' independent interpretation blinded to paramedic or software interpretation)

### Access to Interventions

The rate of reperfusion strategy utilization across groups including fibrinolysis, percutaneous coronary angiography and intervention, coronary artery bypass surgery, bypass to PCI centre directly vs. transfer from a non PCI centre.

### Economic Outcomes

• The direct costs of the 12 lead PHECG program will be estimated.

• Impact on life expectancy gains through reductions in mortality based on the age and gender of subjects [33-35];

• Cost savings with survival benefits for the dominant treatment strategy or cost and outcome trade-offs if one treatment strategy demonstrates cost increasing with survival benefits

• Incremental cost-effectiveness, as measured through additional cost per reduction in door-to-reperfusion time and additional cost per life year gained, will be calculated [36,37].

### Patients Enrolment

A single trained data guardian/abstractor at each base hospital will screen all ambulance call reports and identify all eligible cases. Trained inhospital data abstractors will be notified to conduct a chart review at each receiving hospital.

### Data Management

All prehospital and inhospital data will be abstracted by trained staff and entered on a web based interface employing a structured data set (Additional file 1 - Prehospital Data Variables and Additional file 2 - Inhospital Data Variables) that complies with institutional, privacy and ethical requirements. A manual of operations defines the data name, definition and abstraction instruction for each variable. Error and logic checks were built into the database to screen for abnormal values across forms and within forms at point of entry.

### Analysis Plan

The primary outcome, and treatment time intervals, will be analyzed with one-way ANOVA and subsequent pair-wise multiple comparison procedures across the four treatment strategies. Different covariates (Table 3) will be analyzed using multiple linear regressions and they will be introduced into the model and evaluated as potential confounders. Variables will be retained if they have had an effect of 5% or greater on the coefficients for door-to-reperfusion time. Survival at 30 days and one year for STEMI patients will be analyzed as a binary outcome (Chi square) and as a survival analysis. Covariates that may affect survival will be analyzed with a Cox PH Regression Model. Adverse event rates will be analyzed with a Chi square analysis or a Fisher's exact test.

**Table 3:** Analysis Plan - List of Covariates

1. Presence of cardiac catheterization lab in the primary destination hospital with the ability to perform 24-7 emergent primary angioplasty;
2. Fully affiliated teaching hospital;
3. Is the ED on Time Consideration (TC) or deferral from ambulances based on workload of acutely ill or injured patients?
4. Is the ED on Consideration (C) or deferral from ambulances based on workload?
5. Weekday;
6. Weekend;
7. Time of day in 8 hour intervals (08:00-16:00, 16:00-24:00, 24:00-08:00);
8. Age (as a continuous variable);
9. Sex;
10. History of MI;
11. History of CABG;
12. History of angioplasty;
13. Location of STEMI (anterior, posterior, inferior);
14. Off load delay (minutes);
15. PCA or PCI or bypass surgery.

Economic outcomes will be analyzed using decision analysis supplemented with probabilistic sensitivity analysis in a simulation model. Direct costs will be estimated for each of the four treatment strategies. Door-to-reperfusion times and mortality will be available at the patient level, which will allow for the calculation of averages as well as variability estimates for analysis of uncertainty. Average cost and effectiveness (time-to-reperfusion interval and life years) will be calculated and if one treatment strategy is found to be superior (i.e. cost savings with survival benefits), and then these results will be reported in a cost consequence format. If the superior strategy is found to involve cost and outcome trade-offs (i.e. cost increasing with survival benefits), then incremental cost-effectiveness, as measured through additional cost per reduction in time-to-reperfusion interval and additional cost per life year gained, will be calculated.

### A Priori Subgroup Analysis

• Rural vs. urban settings and academic vs non academic destination hospitals

• Geographical bias subgroup analysis comparing all non PCI capable sites for distance from PCI site;

### Ethical Considerations and Human Subjects Protection

PREDICT is an observational, prospective non-interventional study based on review of routinely collected source data and as such meets the requirements for minimal risk research[38-40]. Approval by 47 research ethics boards/committees covering 71 hospitals will be sought to launch the study.

## Discussion

There is a lack of a comprehensive dataset for Acute Coronary Syndrome (ACS) patients, which includes the prehospital component of care[3]. We anticipate that this study will bridge this gap, providing valuable information on processes of care and the benefits of different prehospital treatment strategies. We have planned to address four threats to protocol compliance and internal validity; 1) ethics approval and privacy requirements from 47 research ethics boards/committees covering 71 hospitals, 2) temporal bias of comparison induced by delays to implementation across sites, 3) data guardian training and oversight of timeliness and quality, and 4) technological advances that may outpace the study and affect recruitment.

This trial involves rural and urban centres and this means that many research ethics boards will need to review this protocol and our request for waiver of consent. We anticipate that rural and small community hospitals will struggle with the request for waiver of consent and the privacy issues associated with chart abstraction, acquisition of personal information enabling telephone follow up at 30 days and at one year. Our strategy will be to obtain approval from all the academic centres first and enclose a copy of their approval with submission to the smaller centres. In addition we have established a data sharing agreement template that has the approval of the administration and legal advisors of the 18 academic and community hospitals in our largest metropolitan area. This agreement has been used successfully in other trials and we hope this will facilitate reaching agreement more quickly with hospitals that have not been involved with research previously. And, finally our investigators will be on call to the research ethics board to participate in the discussion at the time of review. Many small hospital boards request this level of participation by the investigator. We have found in the past that this strategy is helpful in minimizing correspondence back and forth between the research team and the ethics board and reduces time to approval.

We recognize the limitations and challenges that might affect the study's successful implementation and generalizability such as time and spatial challenges. If at all possible, the study should be launched in all centres at the same time. However prior to launching, each PREDICT participating site will need to confer with stakeholders to ensure capture of all cases, procurement of all source documents, optimization of timely data flow, and training of data abstractors. For some centres this preparation and initiation phase will be more elaborative and time consuming then for the others based on volume and existing infrastructure and prior research experience. To address these potential delays we will target the sites we anticipate the REB will be slower to approve the protocol, with follow up calls and offers to complete additional information or speak by teleconference to the board or to the ethics board chair to facilitate understanding and a timely response to queries. We will engage the medical directors in all the sites to ensure the flow of documentation allows for timely data entry and to encourage them to identify and support a high quality data guardian for their site. This engagement will take the form of web based reports of site performance and patient outcomes available 24-7 that enable medical directors to see their data and compare to the aggregate site data. To date most medical directors do not have access to performance or outcome data and we hope providing this accessibility will speed implementation and timely quality data. We anticipate these interventions may be sufficient to allow all sites to participate concurrently and limit the bias related to temporal changes in practices during the trial.

By protocol, the data is collected by trained data guardians in all the sites and since almost all the data guardians are geographically remote from the central research coordinating centre the quality of data may suffer and poise a threat to internal validity. In total there are 10 data guardians abstracting prehospital data and 16 data guardians abstracting inhospital data. To encourage uniformity in data collection and to provide oversight and ensure quality, a number of interventions are planned. All data guardians will be individually trained by one of the investigators (VR). The web based data entry system has built in data definitions and abstraction instructions which are accessible through point and click technology on the variable name at the time of data entry. The abstraction instructions are listed hierarchically ensuring that the data is abstracted from the best source if at all possible. All variables are subject to error and logic checks across other variables and across forms (inhospital and prehospital) which are applied at the time of completion and the case will not close without reconciliation of all the error. Web conferences are conducted for all data guardians to highlight changes to the data set structure, upgrades to the software and discuss difficult variables identified by the data guardians or by the investigators. Data reports to test uniformity are planned and will be discussed at weekly team meetings of the research staff and investigator steering meetings.

Technological advances may outpace the study. Some regions/counties that provide 3-lead ECG in the prehospital setting are not currently considering the change in technology, while other areas are in the planning or transitional stages. Any change from 3-lead to 12-lead in a participating site will compromise recruitment rates and regional comparisons. If this happens an additional 3-lead site with similar geographic and demographic characteristics will be recruited and retrospective data collection will occur to permit concurrent comparisons. In anticipation of this threat to the protocol we have engaged each of the EMS medical directors in the decision to participate. The window of the trial has been confirmed to correspond to the planned changes in the services considering a change.

We have planned a prospective cohort study to compare outcomes across two different prehospital interventions (12-lead and 3-lead) and two system changes (transfer to closest hospital versus bypass closest hospital to transfer directly to a PCI capable hospital) that do not lend themselves to evaluation by a randomized controlled trial. We anticipate there will be challenges related to ethical and privacy, oversight of data guardian abstraction, timeliness of implementation, and technological advances. We hope that this evaluation may be helpful to those involved in developing and enhancing multidisciplinary systems of care including EMS services to advance local care of patients with STEMI and to inform policy decision making and evidence based budgetary decisions that ultimately will affect care across the Province.

## List of abbreviations

ECG: Electrocardiogram; PHECG: Prehospital electrocardiogram; STEMI: ST segment elevated myocardial infarction; EMS: Emergency Medical Services; PCI: Percutaneous coronary intervention; ED: Emergency Department; AMI: Acute myocardial infarction

## Competing interests

The authors declare that they have no competing interests.

## Authors' contributions

RG obtained funding for this study. All authors contributed to the study design and the development of the protocol. WR, CZ and RC contributed to the design of PREDICT web based interface. VER will carry out the study under the guidance of LJM. VER drafted the methods manuscript and all authors contributed to the various iterations prior to publication. All authors read and approved the final manuscript.

## Pre-publication history

The pre-publication history for this paper can be accessed here:

http://www.biomedcentral.com/1471-227X/11/4/prepub

## Supplementary Material

Additional file 1 **PREDICT Prehospital Variables - Structured data set with variables abstracted from Ambulance Call Reports (ACRs)**. Click here for file

Additional file 2 **PREDICT Hospital Variables - Structured data set with variables abstracted from hospital charts**. Click here for file
