# Supplementary material for: Prehospital evaluation and economic analysis of different coronary syndrome treatment strategies - PREDICT - Rationale, Development and Implementation
Source: BMC Emerg Med. 2011 Mar 29;11:4. doi: 10.1186/1471-227X-11-4 (PMC3076236; doi:10.1186/1471-227X-11-4)
Supplement: Additional file 1 — PREDICT Prehospital Variables - Structured data set with variables abstracted from Ambulance Call Reports (ACRs). [file 1471-227X-11-4-S1.PDF]

PREDICT - Prehospital Variables

Variable	Type	Caption	List Options	Abstraction Instruction
**p_ci**	**section**	**Call Identifiers**		
p_id	textbox	Patient ID		
p_epdt	textbox	Episode Date		Earliest call received date of the dispatch centre that has the ability to dispatch EMS or Fire vehicles.
p_ecacc	dropdown	Earliest CACC	listid: ny<br><br>0. no<br>1. yes	Did paramedic personnel record the CACC?<br><br>ACR and AACR - Administration – CACC*<br>0= unknown/not noted<br>1=yes
p_ecaccid	textbox	Earliest CACC ID		ACR and AACR - Administration – CACC*<br>Enter the CACC numerical identifier for the dispatch centre that dispatched the first EMS vehicle (3 digit for Land EMS and 4 digit for Air Ambulance)
p_callnum	textbox	Call Number / Patient Number		ACR - Administration - Call Number/Patient Number<br>AACR - Administration - Patient Number.<br><br>NOTE: On the AACR, please record the 'Patient Number NOT the 'Flight Number.'<br><br>Ensure that the Call Number on the ACR matches the one on the CAD. If they do not match and it appears that the paramedic made an error transcribing the number, use the CAD version.<br>In the case of multiple vehicles, use the Call Number associated with the ACR that was completed by the EMS crew that treated and transported the patient.<br><br>In Toronto use 2 digit year with dash and 7 digits with leading zeros (e.g., “_ 0 5 – 0 0 0 0 0 3 4”)
**p_pip**	**section**	**Patient Identifiers – Prehospital**		
p_bypass	dropdown	Bypass	listid: ny<br>0. no<br>1. yes	
p_stemi	dropdown	STEMI	listid: ny<br>0. no<br>1. yes	
p_cohort	dropdown	Episode Characteristics	listid: p_cohort<br><br>0. CA<br>1. TI<br>2. CP<br><br>3. CA+CP	Cardiac Arrest Registry Criteria = Out of hospital cardiac arrest (NOT associated with burn, blunt or penetrating trauma,), were evaluated by EMS personnel that are part of an organized response<br><br>Treated by EMS;<br>Receives attempts at external defibrillation (by lay responders or emergency personnel),<br><br>Receives chest compressions by organized EMS personnel.

Variable	Type	Caption	List Options	Abstraction Instruction
				External defibrillation does not include attempted cardioversion.<br>Attempted defibrillation does NOT include cases where an AED or defibrillator is applied, no shock is advised, and the patient is determined to not be in cardiac arrest (such as a drug overdose, or presents with a very low GCS).<br><br>Chest compressions does not include precordial thump.<br>Chest compressions does not include those provided by lay responders, EMS, or healthcare providers not part of the organized EMS response.<br><br>NOTE; This group will also include patients to whom a monitor/defibrillator or AED was applied to determine asystole (“no shock advised”) to confirm death and/or patients who may have received CPR by layresponders prior to arrival of the organized EMS response.<br><br>Trauma Registry Criteria = if a patient (irrespective of whether they have a pulse) experienced an out-of-hospital injury, characterized as blunt, penetrating, or burn trauma, is evaluated by ROC EMS personnel (dispatched as part of an organized EMS response) and meets at least ONE of four physiologic critera;<br><br>1. Systolic blood pressure of 90mmHG or less at any time during the call,<br>2. Respiratory rate less than 10, at any time during the call,<br>3. Respiratory rate greater than 29, at any time during the call,<br>4. The patient received and ADVANCED AIRWAY procedures (presumed to be successful placement of oral or nasal endotrachealtube, cricothyrotomy/other surgical approach, or supraglottic airways such as laryngeal mask airway, or combitube)<br><br>5. Glasgow Coma Scale (GCS) of 12 or less at any time during the call<br><br>Also included; patient (irrespective of whether they have a pulse) experienced an out-of-hospital injury, characterized as blunt, penetrating, or burn trauma, is evaluated by ROC EMS personnel (dispatched as part of an organized EMS response) and;

Variable	Type	Caption	List Options	Abstraction Instruction
				a. Is dead in field with EMS treatment and no physiologic criteria documentedâ€”patient died despite EMS treatment,either at the scene or enroute to ED/hospital AND no physiologic criteria were documented during the course of prehospital EMS care. This category does not include those patients that died at the scene or enroute AND had one or more physiologic criteria documented in the patient care record (already included in Epistry) e.g. Traumatic Mechanism.<br><br>Chest Pain Registry Criteria = Patients will be considered for inclusion into the study if all of the following criteria are present: They must:<br><br>1) call 911 for assistance<br>2) are suspected by the paramedics to have ischemic chest pain for greater than 30 minutes but less than 6 hours<br>3) be = 18 years of age and<br>4) experience chest pain that fails to completely respond to nitrates as per standard provincial chest pain protocol.
p_trial	dropdown	Clinical Trial?	listid: trial<br><br>0. No Indication<br>1. Yes - ROC Trial<br>2. Yes - Non-ROC Trial<br>3. Yes - Both	Report if there is any indication that the patient was enrolled in a pre-hospital clinical intervention trialâ€”either a ROC or non-ROC trial â€”prior to admission to the ED/Hospital.<br><br>0=No Indication<br>1=Yes - ROC Trial<br>2=Yes - Non-ROC Trial<br>3=Yes - Both<br><br>If the patient was enrolled in a ROC clinical trial, indicate whether it was a Cardiac or a Trauma study and list that study's CTC Episode ID number. If the patient was enrolled in a non-ROC clinical trial during the course of pre-hospital care, specify the name or briefly describe the type of pre-hospital clinical trial (maximum 60 characters). This data element allows cross reference with the ROC clinical trial database to confirm the complete capture of Epistry or ROC clinical trial candidates

PREDICT - Prehospital Variables

Variable	Type	Caption	List Options	Abstraction Instruction
				Any indication that the patient was enrolled in a clinical trial as indicated by the RDC.<br><br>A list of ROC clinical trials will be provided to all sites<br><br>Not Applicable refers to no ongoing trials in the service.<br>Yes and No refers to enrolment or not enrolment in trials ongoing in the service<br><br>ACR:<br>ACR - Clinical Treatment/Procedures & Results<br><br>if no mention go to<br><br>ACR - General Administration – Remarks / Orders<br><br>AACR:<br>Clinical Treatment / Procedures & Results<br><br>if no mention go to<br><br>AACR - Clinical Treatment / Procedures & Results<br><br>Code 800 or text indicating that the patient was enrolled in a study.
p_rcttyp	dropdown	Type of ROC Study	listid: study<br><br>1. Cardiac study<br>2. Trauma study<br>3. PROPHET study	Was the patient Enrolled in the ROC-Cardiac Study or ROC-Trauma study?<br><br>1=Cardiac study<br>2=Trauma study<br><br>ACR/AACR Possible sources;<br>Incident History<br>Treatment Prior to Arrival<br>Physical Exam – General Appearance<br>Clinical Treatment / Procedures & Results – 800 (Study Drugs)<br>General Administration; Remarks
p_rctspe	textbox	Specify Non-ROC Trial		If patient was part of a NON-ROC clinical trial (during EMS care) indicate what that study was<br><br>Value – Text<br><br>ACR/AACR Possible sources;<br>Incident History<br>Treatment Prior to Arrival<br>Physical Exam – General Appearance

PREDICT - Prehospital Variables

Variable	Type	Caption	List Options	Abstraction Instruction
				Clinical Treatment / Procedures & Results – 800 (Study Drugs)<br>General Administration; Remarks
p_missed	dropdown	Is this a missed case?	listid: ny<br>0. no<br>1. yes	Did patient meet clinical guideline for study trial but was not included?<br><br>ROC Trauma;<br><br>Blunt/Penetrating Trauma<br>Systolic BP 70 or less<br>Age 18 years or older<br><br>OR<br>Systolic BP between 71 – 90<br>Heart Rate 108 or higher<br>Age 18 years or older<br><br>Blunt Head Trauma<br>Traumatic Brain Injury<br>GCS 8 or less before sedation/intubation<br>Age 18 years or older<br><br>ROC Cardiac – Coming Soon
p_pm1	div	Paramedic 1		
p_medic1id	textbox	OASIS ID		Land – General Administration; Heading = Crew Member 1 No. (number)<br><br>Air – Clinical Treatment/Procedures & Results; Heading = Crew Member 1 No. (number)<br><br>Transcribe as is
p_medic1fn	textbox	First Name		Land – General Administration; Heading = Crew Member 1 Name (text)<br><br>Air – Clinical Treatment/Procedures & Results; Heading = Crew Member 1 First, Last name (text)<br><br>Transcribe as is – May only have first initial written in
p_medic1ln	textbox	Last Name		Land – General Administration; Heading = Crew Member 1 No. (number)<br><br>Air – Clinical Treatment/Procedures & Results; Heading = Crew Member 1 First, Last name (text)<br><br>Transcribe as is – May only have first initial written in
p_pm2	div	Paramedic 2		

PREDICT - Prehospital Variables

Variable	Type	Caption	List Options	Abstraction Instruction
p_medic2id	textbox	OASIS ID		Land – General Administration; Heading = Crew Member 2 No. (number)<br><br>Air – Clinical Treatment/Procedures & Results; Heading = Crew Member 2 No. (number)<br><br>Transcribe as is
p_medic2fn	textbox	First Name		Land – General Administration; Heading = Crew Member 2 Name (text)<br><br>Air – Clinical Treatment/Procedures & Results; Heading = Crew Member First, Last name (text)<br><br>Transcribe as is
p_medic2ln	textbox	Last Name		Land – General Administration; Heading = Crew Member 1 No. (number)<br><br>Air – Clinical Treatment/Procedures & Results; Heading = Crew Member 1 First, Last name (text)<br><br>Transcribe as is
p_pi	div	Patient Information		
p_plname	textbox	Surname		ACR - Administration – Surname<br><br>Transcribe as-is.<br><br>Caps Lock<br><br>In the case of multiple vehicles, use the Surname associated with the ACR that was completed by the EMS crew that treated and transported the patient
p_pfname	textbox	Given Name		ACR - Administration – Given Name<br><br>Transcribe as-is.<br><br>Caps Lock<br><br>In the case of multiple vehicles, use the Given Name associated with the ACR that was completed by the EMS crew that treated and transported the patient.
p_paddr	textbox	Mailing Address		ACR - Administration – Mailing Address

Variable	Type	Caption	List Options	Abstraction Instruction
				Transcribe as-is.<br><br>Caps Lock<br><br>In the case of multiple vehicles, use the Mailing Address associated with the ACR that was completed by the EMS crew that treated and transported the patient
p_pcity	textbox	City/Town		ACR - Administration – City / Town<br><br>Transcribe as-is.<br><br>Caps Lock<br><br>In the case of multiple vehicles, use the City / Town associated with the ACR that was completed by the EMS crew that treated and transported the patient
p_pprov	dropdown	Province	listid: prov<br>1. AB<br>2. BC<br>3. MB<br>4. NB<br>5. NF<br>6. NS<br>7. NT<br>8. NU<br>9. ON<br>10. PE<br>11. QC<br>12. SK<br>13. YT<br>99. Out of Country	ACR - Administration – Province<br><br>Transcribe as-is.<br><br>NOTE: If this field is left blank on the ACR or AACR, please leave blank here.<br><br>Caps Lock<br><br>In the case of multiple vehicles, use the Province associated with the ACR that was completed by the EMS crew that treated and transported the patient<br><br>If a State is entered instead of a Province, enter as-is
p_ppostc	textbox	Postal Code		ACR - Administration – Postal Code<br><br>Transcribe as-is.<br><br>Caps Lock<br><br>In the case of multiple vehicles, use the Postal Code associated with the ACR that was completed by the EMS crew that treated and transported the patient

Variable	Type	Caption	List Options	Abstraction Instruction
				If a Zip Code is entered instead of a Postal Code, enter the first 6 digits of the Zip Code as-is.
p_phospreg	textbox	Hospital Registration Number / Patient Chart Number		ACR - Administration – Hospital Registration Number<br><br>**NOTE: On the AACR, this is NOT the 'patient number.'**<br><br>AACR- please scan Administration section to see if this information is recorded anywhere. There is not a specific field for this item.<br><br>Transcribe as-is.<br><br>Caps Lock<br><br>In the case of multiple vehicles, use the Hospital Registration Number associated with the ACR that was completed by the EMS crew that treated and transported the patient<br><br>Note this field will be not applicable for patients who are not transported to an ED or hospital and paramedics will not always be able to retrieve this in the ED.
p_hc	div	Health Card		
p_phlthcn	textbox	Number		ACR Administration – Health Card No<br><br>RDC – Health Card No.<br><br>Transcribe as-is
p_phlthcv	textbox	Version Code		RDC – ACR Administration – Version No.<br><br>Health Card Version No<br><br>Transcribe as-is<br><br>Caps Lock

PREDICT - Prehospital Variables

Variable	Type	Caption	List Options	Abstraction Instruction
p_pdob	textbox	Date of Birth		ACR - Administration – Date of Birth ()<br><br>Transcribe as-is<br><br>Due to conditions in the field, a full date of birth might not be available. A partial date is acceptable. For example if only year is available, transcribe year.<br><br>In the case of multiple vehicles, use the Date of Birth associated with the ACR that was completed by the EMS crew that treated and transported the patient<br><br>This field is actually three (Year, Month, & Day)
p_ea	div	Estimated Age		
p_estageu	dropdown	Units	listid: ymd<br><br>1. years<br>2. months<br>3. days	Depending on whether patient is an infant, young child or adult age may be estimated in days, months or years.<br><br>This value will indicate unit of age value.<br><br>1 = Years<br>2 = Months<br>3 = Days<br><br>Land – Physical Exam; Heading = Age<br><br>Air – Administration; Heading = Age
p_estagev	textbox	Value		Numerical value determined or estimated by treating EMS crew.<br><br>Land – Physical Exam; Heading = Age<br><br>Air – Administration; Heading = Age<br><br>If age range is given (E.g. 60-70) take the mid point = 65.<br>If approximate number is given, transcribe value as is (E.g. ~60 = 60)
p_agecat	dropdown	Age Category (years)	listid: agecat<br><br>1. infant (<1)<br>2. child (1-11)<br>3. adolencent (12-19)<br>4. young adult (20-39)<br>5. middle age (40-60)<br>6. older (61-75)	Categorical value assigned based on age as recorded by treating crew<br><br>Land – Physical Exam; Heading = Age<br><br>Air – Administration; Heading = Age<br><br>1=infant (<1)

Variable	Type	Caption	List Options	Abstraction Instruction
			7. elderly (>75)<br>8. unknown/not noted	2=child (1-11)<br>3=adolescent (12-19)<br><br>4=young adult (20-39)<br>5=middle age (40-60)<br>6=older (61-75)<br>7=elderly (>75)<br>8=unknown/not noted
p_sexp	dropdown	Gender	listid: sex<br>0. female<br>1. male<br>2. not noted	ACR - Physical Exam – Gender<br><br>AACR - Administration – Gender<br><br>Transcribe as-is<br><br>In the case of multiple vehicles, use the ACR that was completed by the EMS crew that treated and transported the patient<br><br>In some cases the medic might not be able to ascertain the gender of an accident victim, i.e., gender=”Unknown”. Cross gender is categorized here as “Other”.
p_wght	dropdown	Weight	listid: uy<br>0. unknown/not noted<br><br>1. yes	ACR - Physical Exam – Weight<br><br>AACR - Administration – Weight<br><br>Transcribe as-is<br><br>In the case of multiple vehicles, use the ACR that was completed by the EMS crew that treated and transported the patient.<br><br>In some cases the medic might not be able to ascertain the weight of a patient, i.e., weight = unknown/not noted.
p_wghtkg	textbox	Weight (kg)		ACR - Physical Exam – Weight<br><br>AACR - Administration – Weight<br><br>Transcribe as-is<br><br>In the case of multiple vehicles, use the ACR that was completed by the EMS crew that treated and transported the patient.<br><br>In some cases the medic might not be able to ascertain the weight of a patient, i.e., gender=”Unknown”.
**p_ccg**	**section**	**Call Characteristics**		
p_pu	div	Pick-up Location		

PREDICT - Prehospital Variables

Variable	Type	Caption	List Options	Abstraction Instruction
p_puloc	textbox	Description		ACR/AACR - Administration – Pick-up Location<br><br>Pick-up location is defined here as the location of the episode. Transcribe closest street address to location of episode from this field. If an intersection is entered, please find the closet street address.<br><br>NOTE: In some instances you may have to abstract some information from the ''Mailing Address'' for the Land ACRs or from the ''Sending Facility'' section on the ACR.<br><br>The importance of this field and the method of completing it will be emphasized in future medic training sessions.<br><br>Caps Lock<br><br>In the case of multiple vehicles, use the ACR that was completed by the EMS crew that treated and transported the patient
p_loctype	textbox	Type		ACR - Administration –Pick-up Location Code<br><br>if no code then go to<br><br>ACR - Clinical Information – Incident History<br><br>if no mention then go to<br><br>ACR - General Administration – Remarks / Orders
p_gi	div	Geographic Identifier		
p_pupc	textbox	Postal Code		Obtain the postal code using the Canada Post website http://www.canadapost.ca/tools/pcl/bin/advanced-e.asp?sblid=pcl
p_utm	div	UTM		
p_lnorth	textbox	Northing		Universal Transverse Mercator mapping code for given call location.<br><br>This number includes a 2 digit zone code, 3 digit easting code and a 4 digit northing code.<br><br>E.g. 22 477 4273<br><br>22 = digit code<br>477 = easting code<br>4273 = northing code

Variable	Type	Caption	List Options	Abstraction Instruction
				Land – General Administration; Heading UTM Code/Flight # (number)<br><br>Air – UTM codes not issued to Air Ambulance calls.
p_least	textbox	Easting		Universal Transverse Mercator mapping code for given call location.<br><br>This number includes a 2 digit zone code, 3 digit easting code and a 4 digit northing code.<br><br>E.g. 22 477 4273<br><br>22 = digit code<br>477 = easting code<br>4273 = northing code<br><br>Land – General Administration; Heading UTM Code/Flight # (number).<br><br>Air – UTM codes not issued to Air Ambulance calls.
p_lzone	textbox	Zone		Universal Transverse Mercator mapping code for given call location.<br><br>This number includes a 2 digit zone code, 3 digit easting code and a 4 digit northing code.<br><br>E.g. 22 477 4273<br><br>22 = digit code<br>477 = easting code<br>4273 = northing code<br><br>Land – General Administration; Heading UTM Code/Flight # (number).<br><br>Air – UTM codes not issued to Air Ambulance calls.
p_lt	div	Lat/Long		
p_llat	textbox	Latitude		Geographic coordinate system for a given call location.<br><br>Land – Up to 8 digit number on CAD; Heading = Latitude (number).<br><br>Air – AACR Administration; Heading = On Scene GPS – 4 numbers following N (number).
p_llong	textbox	Longitude		Geographic coordinate system for a given call location.

PREDICT - Prehospital Variables

Variable	Type	Caption	List Options	Abstraction Instruction
				Land – Up to 8 digit number on CAD; Heading = Longitude (number).<br><br>Air – AACR Administration; Heading = On Scene GPS – 4 numbers following W.
p_ldatum	dropdown	Datum	listid: Datum<br>1. nad83<br>2. nad27<br>3. wgs84Â	The topographic map standard to which the lat/long coordinates are applied.<br><br>1=nad83<br>2=nad27<br>3=wgs84<br><br>There are three map datums used in the western hemisphere:<br>NAD27 (North American Datum 1927)<br>NAD83 (North American Datum 1983)<br>WGS84 (World Geodetic System 1984, based on satellite measurements).
p_locunk	dropdown	Unknown/None Noted	listid: ny<br>0. no<br>1. yes	Location not noted on ACR, MAR or CAD = Call location unknown<br><br>1 = Yes, location unknown<br>2 = No, call location known
p_emsres	dropdown	EMS Response	listid: emsres<br>1. one vehicle<br>2. two vehicles<br>3. three vehicles<br>4. four vehicles<br>5. more than four vehicles<br>6. not noted	CAD - Arr. Scene<br><br>ACR – Clinical Information – Treatment ptior to arrival<br><br>Please do NOT interpret from the 'Incident History' description.<br><br>Collect all print-outs relevant to the patient and attempt to ascertain how many vehicles arrived on scene (time stamped arrived on scene).<br><br>Note that the number of ACRs does not necessarily equate to the number of vehicles on scene. The CAD is a better truer indication of the number of vehicles on scene.
p_emsresv	textbox	How Many?		If 5 or greater EMS vehicles were on scene indicate numerical value here.<br><br>Collect all CAD printouts relevant to the patient and attempt to ascertain how many vehicles arrived on scene.<br><br>Includes Land Ambulance, Air Ambulance and Fire Responders.
p_resveh	grid	Responding Vehicles		
p_rig1dsr	dropdown	Source	listid: srcnt<br>0. no time	Source of information for the first vehicle dispatch time.

Variable	Type	Caption	List Options	Abstraction Instruction
			1. acr<br>2. cad<br>3. mar<br>4. aacr	0=acr – Ambulance Call Report (Land)<br>1=cad – Computer Aided Dispatch (Land)<br>2=mar – Medical Assist Report (Fire)<br>3=aacr – Air Ambulance Call Report (Air)
p_rig1dtm	textbox	Time		CAD - Crew Ntf<br><br>Data Guardian abstracts from appropriate CAD. If not available obtain from ACR “Call Events”<br><br>NOTE: Air does not have a crew notified time.<br><br>Time when the first arriving vehicle was notified by dispatch.
p_v1ag	dropdown	Service Number	listid: p_services<br>** See list items in appendix	ACR - Administration – Service<br><br>AACR - not a field<br><br>NOTE: If prior crew and vehicle information are unattainable, please leave blank.<br><br>In the case of multiple vehicles, use the ACR that was completed by the EMS crew that treated and transported the patient.<br><br>EMS is 3 digits and Fire is 4 digits.<br><br>Land EMS:<br><br>262. Sault Ste Marie EMS<br><br>491. Toronto EMS<br><br>701. Brant EMS<br><br>701. Grey EMS<br><br>720. Muskoka EMS

Variable	Type	Caption	List Options	Abstraction Instruction
				722. Norfolk EMS<br><br>724. Durham EMS<br><br>727. Peel EMS<br><br>733. Simcoe EMS<br><br>738. Hamilton EMS<br><br>740. Algoma EMS<br><br>747. Sudbury EMS<br><br>749. Thunder Bay EMS (Superior North EMS)<br><br>752. Manitoulin-Sudbury EMS (East Side)<br><br>753. Haldimand EMS<br><br>782. Manitoulin-Sudbury EMS (West Side)<br><br>789. York EMS<br><br>818. OAASC<br><br>1801. Durham Fire Pickerin<br><br>1805. Durham Fire Ajax<br><br>1809. Durham Fire Whitby<br><br>1813. Durham Fire Oshawa<br><br>1817. Durham Fire Claringt<br><br>1820. Durham Fire Scugog<br><br>1829. Durham Fire Uxbridge<br><br>1839. Durham Fire Brock<br><br>2005. Toronto Fire<br><br>2105. Peel Fire Mississaug<br><br>2110. Peel Fire Brampton<br><br>2124. Peel Fire Caledon

PREDICT - Prehospital Variables

Variable	Type	Caption	List Options	Abstraction Instruction
				2505. Hamilton Fire<br><br>4307. Simcoe Fire New Tec<br><br>4312. Simcoe Fire Bradford<br><br>4317. Simcoe Fire Innisfil<br><br>4319. Simcoe Fire Ramara<br><br>4321. Simcoe Fire Essa<br><br>4323. Simcoe Fire Oro Med<br><br>4342. Simcoe Fire Barrie<br><br>4350. Simcoe Fire Mnjikani<br><br>4352. Simcoe Fire Orillia<br><br>4364. Simcoe Fire Wasaga<br><br>4465. Muskoka Fire Georg<br><br>9999 =Other<br><br>Null=Not available/missing
p_v1id	dropdown	Vehicle Number	listid: rigs	ACR – Administration - Vehicle Number<br><br>CAD Veh<br><br>NOTE: If prior crew and vehicle information are unattainable, please leave blank.<br><br>Toronto uses a 3 digit vehicle ID number. Other services use a 4 digit number. Fire uses an alphanumeric ID i.e. P101. Air uses 5 character alpha vehicle ID.
p_rig1np	textbox	Number of Personnel		Number of crew members staffing the vehicle at the time of the call.

PREDICT - Prehospital Variables

Variable	Type	Caption	List Options	Abstraction Instruction
				Indicate the number of crew members staffing the vehicle at the time of the call. This includes all Students (Level 1, 2, 3, & CCP) with valid OASIS Numbers. This does not include observers or any kind (media, laypersons, students on non-precept observation shifts, physicians, RNs, other health care workers).<br><br>ACR - General Administration – Crew member 1 / Crew member 2 / Crew member 3 / Crew member 4 Can obtain from CAD if available.<br><br>in conjunction with<br><br>ACR - General Administration – Remarks / Orders<br><br>AACR - Clinical Treatment / Procedures & Results – Paramedic 1 / Paramedic 2 / Paramedic 3<br><br>in conjunction with<br><br>AACR - Clinical Treatment / Procedures & Results – Physician's Orders/Remarks<br><br>Text indicating number of crew members on the vehicle. The ACR - General Administration – Remarks / Orders or theAACR -Clinical Treatment / Procedures & Results – Physician's Orders/Remarks may need to be referenced to determine if crew members 3 &/or 4 were staffing the vehicle or came from another responding vehicle.
p_v1sl	dropdown	Service Level	listid: crewtype<br><br>1. ema<br>2. pcp<br>3. acp<br>4. ccp<br>5. flight<br>6. fr<br>7. fire<br>8. air pcp (code 5)	ACR - small print top right corner page 1 - Crew Type. If not marked, determine from the ACR.<br><br>AACR - Administration – level of care (A/C). Codes: 5 = primary care; 6 = advanced care; 7 = critical care<br><br>AACR - Administration – level of care (A/C).<br><br>If Code = 5 (primary care), then enter 7<br>6 (advanced care), then enter 8<br>7 (critical care), then enter 9

PREDICT - Prehospital Variables

Variable	Type	Caption	List Options	Abstraction Instruction
			9. air acp (code 6)<br><br>10. air ccp (code 7)<br><br>11. not noted	
p_rig2dsr	dropdown	Source	listid: srcnt<br><br>0. no time<br>1. acr<br>2. cad<br>3. mar<br>4. aacr	Source of information for the second vehicle dispatch time.<br><br>0=acr – Ambulance Call Report (Land)<br>1=cad – Computer Aided Dispatch (Land)<br>2=mar – Medical Assist Report (Fire)<br>3=aacr – Air Ambulance Call Report (Air)
p_rig2dtm	textbox	Dispatch Time		CAD - Crew Ntf<br><br>Data Guardian abstracts from appropriate CAD. If not available obtain from ACR "Call Events"<br><br>Time when the second arriving vehicle was notified by dispatch.
p_v2src	dropdown	Source	listid: srcnt<br><br>0. no time<br>1. acr<br>2. cad<br>3. mar<br>4. aacr	Source of information for the second vehicle arrival time.<br><br>0=No time (E.g. not recorded)<br>1=acr – Ambulance Call Report (Land)<br>2=cad – Computer Aided Dispatch (Land)<br>3=aacr – Air Ambulance Call Report (Air)<br><br>4=mar – Medical Assist Report (Fire)
p_rig2tm	textbox	Time		CAD – Arr. Scene<br><br>Data Guardian abstracts from appropriate CAD. If not available obtain from ACR "Call Events"<br><br>Time when the second arriving vehicle arrived at the scene.
p_v2ag	dropdown	Service Number	listid: p_services<br>** See list items in appendix	ACR - Administration – Service<br><br>AACR - not a field<br><br>NOTE: If prior crew and vehicle information are unattainable, please leave blank.

PREDICT - Prehospital Variables

Variable	Type	Caption	List Options	Abstraction Instruction
				In the case of multiple vehicles, use the ACR that was completed by the EMS crew that treated and transported the patient.<br><br>EMS is 3 digits and Fire is 4 digits.<br><br>Land EMS:<br><br>262. Sault Ste Marie EMS<br><br>491. Toronto EMS<br><br>701. Brant EMS<br><br>701. Grey EMS<br><br>720. Muskoka EMS<br><br>722. Norfolk EMS<br><br>724. Durham EMS<br><br>727. Peel EMS<br><br>733. Simcoe EMS<br><br>738. Hamilton EMS<br><br>740. Algoma EMS<br><br>747. Sudbury EMS<br><br>749. Thunder Bay EMS (Superior North EMS)<br><br>752. Manitoulin-Sudbury EMS (East Side)<br><br>753. Haldimand EMS<br><br>782. Manitoulin-Sudbury EMS (West Side)<br><br>789. York EMS<br><br>818. OAASC<br><br>1801. Durham Fire Pickerin

PREDICT - Prehospital Variables

Variable	Type	Caption	List Options	Abstraction Instruction
				1805. Durham Fire Ajax<br><br>1809. Durham Fire Whitby<br><br>1813. Durham Fire Oshawa<br><br>1817. Durham Fire Claringt<br><br>1820. Durham Fire Scugog<br><br>1829. Durham Fire Uxbridge<br><br>1839. Durham Fire Brock<br><br>2005. Toronto Fire<br><br>2105. Peel Fire Mississaug<br><br>2110. Peel Fire Brampton<br><br>2124. Peel Fire Caledon<br><br>2505. Hamilton Fire<br><br>4307. Simcoe Fire New Tec<br><br>4312. Simcoe Fire Bradford<br><br>4317. Simcoe Fire Innisfil<br><br>4319. Simcoe Fire Ramara<br><br>4321. Simcoe Fire Essa<br><br>4323. Simcoe Fire Oro Med<br><br>4342. Simcoe Fire Barrie<br><br>4350. Simcoe Fire Mnjikani<br><br>4352. Simcoe Fire Orillia<br><br>4364. Simcoe Fire Wasaga<br><br>4465. Muskoka Fire Georg<br><br>9999 =Other<br><br>Null=Not available/missing
p_v2id	dropdown	Vehicle Number	listid: rigs	ACR – Administration - Vehicle Number<br>CAD Veh

PREDICT - Prehospital Variables

Variable	Type	Caption	List Options	Abstraction Instruction
				Toronto uses a 3 digit vehicle ID number. Other services use a 4 digit number. Fire uses an alphanumeric ID i.e. P101.<br><br>Air uses 5 character alpha vehicle ID.
p_rig2np	textbox	Number of Personnel		RDC – Number of crew members staffing the vehicle at the time of the call.<br><br>Indicate the number of crew members staffing the vehicle at the time of the call. This includes all Students (Level 1, 2, 3, & CCP) with valid OASIS Numbers. This does not include observers or any kind (media, laypersons, students on non-precept observation shifts, physicians, RNs, other health care workers).<br><br>ACR - General Administration – Crew member 1 / Crew member 2 / Crew member 3 / Crew member 4. Can obtain from CAD if available.<br><br>in conjunction with<br><br>ACR - General Administration – Remarks / Orders<br><br>AACR -Clinical Treatment / Procedures & Results – Paramedic 1 / Paramedic 2 / Paramedic 3<br><br>in conjunction with<br><br>AACR - Clinical Treatment / Procedures & Results – Physician's Orders/Remarks<br><br>Text indicating number of crew members on the vehicle. The ACR - General Administration – Remarks / Orders or theAACR -Clinical Treatment / Procedures & Results – Physician's Orders/Remarks may need to be referenced to determine if crew members 3 &/or 4 were staffing the vehicle or came from another responding vehicle.
p_v2sl	dropdown	Service Level	listid: crewtype<br><br>1. ema<br>2. pcp<br><br>3. acp<br>4. ccp	ACR - small print top right corner page 1 - Crew Type. If not marked, determine from the ACR.<br><br>AACR - Administration – level of care (A/C). Codes: 5 = primary care; 6 = advanced care; 7 = critical care<br><br>AACR - Administration – level of care (A/C).

PREDICT - Prehospital Variables

Variable	Type	Caption	List Options	Abstraction Instruction
			5. flight<br>6. fr<br>7. fire<br>8. air pcp (code 5)<br>9. air acp (code 6)<br>10. air ccp (code 7)<br>11. not noted	If Code = 5 (primary care), then enter 7<br>6 (advanced care), then enter 8<br>7 (critical care), then enter 9
p_rig3dsr	dropdown	Source	listid: srcnt<br>0. no time<br>1. acr<br>2. cad<br>3. mar<br>4. aacr	Source of information for the third vehicle dispatch time.<br>0=acr – Ambulance Call Report (Land)<br>1=cad – Computer Aided Dispatch (Land)<br>2=mar – Medical Assist Report (Fire)<br>3=aacr – Air Ambulance Call Report (Air)
p_rig3dtm	textbox	Dispatch Time		CAD - Crew Ntf<br>Data Guardian abstracts from appropriate CAD. If not available obtain from ACR “Call Events”<br>Time when the third arriving vehicle was notified by dispatch.
p_v3src	dropdown	Source	listid: srcnt<br>0. no time<br>1. acr<br>2. cad<br>3. mar<br>4. aacr	Source of information for third vehicle arrival time.<br>0=No time (E.g. Non-Roc or not recorded)<br>1=acr – Ambulance Call Report (Land)<br>2=cad – Computer Aided Dispatch (Land)<br>3=aacr – Air Ambulance Call Report (Air)<br>4=mar – Medical Assist Report (Fire)
p_rig3tm	textbox	Time		CAD – Arr. Scene<br>Data Guardian abstracts from appropriate CAD. If not available obtain from ACR “Call Events”<br>Time when the third arriving vehicle arrived sceme
p_v3ag	dropdown	Service Number	listid: p_services<br>** See list items in appendix	ACR - Administration - Service<br>AACR - not a field<br>EMS is 3 digits and Fire is 4 digits.<br>Land EMS:<br>262. Sault Ste Marie EMS

PREDICT - Prehospital Variables

Variable	Type	Caption	List Options	Abstraction Instruction
				491. Toronto EMS<br>701. Brant EMS<br>701. Grey EMS<br>720. Muskoka EMS<br>722. Norfolk EMS<br>724. Durham EMS<br>727. Peel EMS<br>733. Simcoe EMS<br>738. Hamilton EMS<br>740. Algoma EMS<br>749. Thunder Bay EMS (Superior North EMS)<br><br>753. Haldimand EMS<br>789. York EMS<br>818. OAASC<br>1801. Durham Fire Pickerin<br>1805. Durham Fire Ajax<br>1809. Durham Fire Whitby<br>1813. Durham Fire Oshawa<br>1817. Durham Fire Claringt<br>1820. Durham Fire Scugog<br>1829. Durham Fire Uxbridge<br>1839. Durham Fire Brock<br>2005. Toronto Fire<br>2105. Peel Fire Mississaug<br>2110. Peel Fire Brampton<br>2124. Peel Fire Caledon<br>2505. Hamilton Fire<br>4307. Simcoe Fire New Tec<br>4312. Simcoe Fire Bradford<br>4317. Simcoe Fire Innisfil<br>4319. Simcoe Fire Ramara<br>4321. Simcoe Fire Essa<br>4323. Simcoe Fire Oro Med<br>4342. Simcoe Fire Barrie<br>4350. Simcoe Fire Mnjikani<br>4352. Simcoe Fire Orillia<br>4364. Simcoe Fire Wasaga<br>4465. Muskoka Fire Georg<br>9999 =Other<br>Null=Not available/missing
p_v3id	dropdown	Vehicle Number	listid: rigs	ACR – Administration - Vehicle Number<br>CAD Veh<br><br>Toronto uses a 3 digit vehicle ID number. Other sercives use a 4 digit number. Fite uses an alphanumeric ID i.e. P101.<br><br>Air uses 5 character alpha vehicle ID.

PREDICT - Prehospital Variables

Variable	Type	Caption	List Options	Abstraction Instruction
p_rig3np	textbox	Number of Personnel		RDC – Number of crew members staffing the vehicle at the time of the call.<br><br>Indicate the number of crew members staffing the vehicle at the time of the call. This includes all Students (Level 1, 2, 3, & CCP) with valid OASIS Numbers. This does not include observers or any kind (media, laypersons, students on non-precept observation shifts, physicians, RNs, other health care workers).<br><br>ACR - General Administration – Crew member 1 / Crew member 2 / Crew member 3 / Crew member 4. Can obtain from CAD if available.<br><br>in conjunction with<br><br>ACR - General Administration – Remarks / Orders<br><br>AACR -Clinical Treatment / Procedures & Results – Paramedic 1 / Paramedic 2 / Paramedic 3<br><br>in conjunction with<br><br>AACR -Clinical Treatment / Procedures & Results – Physician's Orders/Remarks<br><br>Text indicating number of crew members on the vehicle. The ACR - General Administration – Remarks / Orders or theAACR -Clinical Treatment / Procedures & Results – Physician's Orders/Remarks may need to be referenced to determine if crew members 3 &/or 4 were staffing the vehicle or came from another responding vehicle.
p_v3sl	dropdown	Service Level	listid: crewtype<br><br>1. ema<br>2. pcp<br>3. acp<br>4. ccp<br>5. flight<br>6. fr<br>7. fire	ACR - small print top right corner page 1 - Crew Type. If not marrked determine from the ACR.<br><br>AACR - Administration – level of care (A/C). Codes: 5 = primary care; 6 = advanced care; 7 = critical care<br><br>AACR - Administration – level of care (A/C).<br><br>If Code = 5 (primary care), then enter 7<br>6 (advanced care), then enter 8<br>7 (critical care), then enter 9

Variable	Type	Caption	List Options	Abstraction Instruction
			8. air pcp (code 5)<br>9. air acp (code 6)<br>10. air ccp (code 7)<br>11. not noted	
p_rig4dsr	dropdown	Source	listid: srcnt<br>0. no time<br>1. acr<br>2. cad<br>3. mar<br>4. aacr	Source of information for first vehicle arrival time.<br>0=acr – Ambulance Call Report (Land)<br>1=cad – Computer Aided Dispatch (Land)<br>2=mar – Medical Assist Report (Fire)<br>3=aacr – Air Ambulance Call Report (Air)
p_rig4dtm	textbox	Dispatch Time		CAD - Crew Ntf<br>Data Guardian abstracts from appropriate CAD. If not available obtain from ACR "Call Events".<br>Time when the fourth arriving vehicle was notified by dispatch.
p_v4src	dropdown	Source	listid: srcnt<br>0. no time<br>1. acr<br>2. cad<br>3. mar<br>4. aacr	Source of information for fourth vehicle arrival time.<br>0=No time (E.g. Non-Roc or not recorded)<br>1=acr – Ambulance Call Report (Land)<br>2=cad – Computer Aided Dispatch (Land)<br>3=aacr – Air Ambulance Call Report (Air)<br>4=mar – Medical Assist Report (Fire)
p_rig4tm	textbox	Time		CAD – Arr. Scene<br>Data Guardian abstracts from appropriate CAD. If not available obtain from ACR "Call Events".<br>Time when the fourth arriving vehicle arrived scene.
p_v4ag	dropdown	Service Number	listid: p_services<br>** See list items in appendix	ACR - Administration – Service<br>AACR - not a field<br>NOTE: If prior crew and vehicle information are unattainable, please leave blank.

PREDICT - Prehospital Variables

Variable	Type	Caption	List Options	Abstraction Instruction
				In the case of multiple vehicles, use the ACR that was completed by the EMS crew that treated and transported the patient.<br><br>EMS is 3 digits and Fire is 4 digits.<br><br>Land EMS:<br>262. Sault Ste Marie EMS<br>491. Toronto EMS<br>701. Brant EMS<br>701. Grey EMS<br>720. Muskoka EMS<br>722. Norfolk EMS<br>724. Durham EMS<br>727. Peel EMS<br>733. Simcoe EMS<br>738. Hamilton EMS<br>740. Algoma EMS<br>747. Sudbury EMS<br>749. Thunder Bay EMS (Superior North EMS)<br>752. Manitoulin-Sudbury EMS (East Side)<br>753. Haldimand EMS<br>782. Manitoulin-Sudbury EMS (West Side)<br>789. York EMS<br>818. OAASC<br>1801. Durham Fire Pickerin

PREDICT - Prehospital Variables

Variable	Type	Caption	List Options	Abstraction Instruction
				1805. Durham Fire Ajax<br><br>1809. Durham Fire Whitby<br><br>1813. Durham Fire Oshawa<br><br>1817. Durham Fire Claringt<br><br>1820. Durham Fire Scugog<br><br>1829. Durham Fire Uxbridge<br><br>1839. Durham Fire Brock<br><br>2005. Toronto Fire<br><br>2105. Peel Fire Mississaug<br><br>2110. Peel Fire Brampton<br><br>2124. Peel Fire Caledon<br><br>2505. Hamilton Fire<br><br>4307. Simcoe Fire New Tec<br><br>4312. Simcoe Fire Bradford<br><br>4317. Simcoe Fire Innisfil<br><br>4319. Simcoe Fire Ramara<br><br>4321. Simcoe Fire Essa<br><br>4323. Simcoe Fire Oro Med<br><br>4342. Simcoe Fire Barrie<br><br>4350. Simcoe Fire Mnjikani<br><br>4352. Simcoe Fire Orillia<br><br>4364. Simcoe Fire Wasaga<br><br>4465. Muskoka Fire Georg<br><br>9999 =Other<br><br>Null=Not available/missing
p_v4id	dropdown	Vehicle Number	listid: rigs	ACR – Administration - Vehicle Number<br>CAD Veh

Variable	Type	Caption	List Options	Abstraction Instruction
				Toronto uses a 3 digit vehicle ID number. Other services use a 4 digit number. Fire uses an alphanumeric ID i.e. P101. Air uses 5 character alpha vehicle ID.
p_rig4np	textbox	Number of Personnel		RDC – Number of crew members staffing the vehicle at the time of the call.<br><br>Indicate the number of crew members staffing the vehicle at the time of the call. This includes all Students (Level 1, 2, 3, & CCP) with valid OASIS Numbers. This does not include observers or any kind (media, laypersons, students on non-precept observation shifts, physicians, RNs, other health care workers).<br><br>ACR - General Administration – Crew member 1 / Crew member 2 / Crew member 3 / Crew member 4. Can obtain from CAD if available.<br><br>in conjunction with<br><br>ACR - General Administration – Remarks / Orders<br><br>AACR -Clinical Treatment / Procedures & Results – Paramedic 1 / Paramedic 2 / Paramedic 3<br><br>in conjunction with<br><br>AACR -Clinical Treatment / Procedures & Results – Physician's Orders/Remarks<br><br>Text indicating number of crew members on the vehicle. The ACR - General Administration – Remarks / Orders or theAACR -Clinical Treatment / Procedures & Results – Physician's Orders/Remarks may need to be referenced to determine if crew members 3 &/or 4 were staffing the vehicle or came from another responding vehicle.
p_v4sl	dropdown	Service Level	listid: crewtype<br><br>1. ema<br>2. pcp<br><br>3. acp<br>4. ccp	ACR - small print top right corner page 1 - Crew Typw. If not marked, determine from ACR.<br><br>AACR - Administration – level of care (A/C). Codes: 5 = primary care; 6 = advanced care; 7 = critical care<br><br>AACR - Administration – level of care (A/C).

PREDICT - Prehospital Variables

Variable	Type	Caption	List Options	Abstraction Instruction
			5. flight<br>6. fr<br>7. fire<br>8. air pcp (code 5)<br>9. air acp (code 6)<br>10. air ccp (code 7)<br>11. not noted	If Code = 5 (primary care), then enter 7<br>6 (advanced care), then enter 8<br>7 (critical care), then enter 9
p_mtc	div	Main Treating Crew		
p_mtcseq	dropdown	Arrive at Scene Sequence	listid: vehord<br>1. 1st vehicle<br>2. 2nd vehicle<br>3. 3rd vehicle<br>4. 4th vehicle<br>5. other	Where in sequence of EMS vehicle arrival did Main Treating Crew (MTC) arrive?<br>1=MTC was 1st vehicle<br>2=MTC was 2nd vehicle<br>3=MTC was 3rd vehicle<br>4=MTC was 4th vehicle<br>5=MTC was 5th vehicle or later<br>E.g. Fire arrive first, MTC arrive second – Value = 2
p_mtcsqs	textbox	Specify		If Main Treating Crew (MTC) arrived as the 5th vehicle or later, indicate where in sequence of EMS vehicles they arrived.<br>Value = 5 or greater
p_crewtype	dropdown	Crew Type	listid: crewtype<br>1. ema<br>2. pcp<br>3. acp<br>4. ccp<br>5. flight<br>6. fr<br>7. fire<br>8. air pcp (code 5)<br>9. air acp (code 6)<br>10. air ccp (code 7)<br>11. not noted	ACR - small print top right corner page 1 - Crew Type. If not marked, determine from ACR.<br>AACR - Administration – level of care (A/C). Codes: 5 = primary care; 6 = advanced care; 7 = critical care<br>AACR - Administration – level of care (A/C).<br>If Code = 5 (primary care), then enter 7<br>6 (advanced care), then enter 8<br>7 (critical care), then enter 9<br>In the case of multiple vehicles, use the ACR that was completed by the EMS crew that treated and transported the patient.

PREDICT - Prehospital Variables

Variable	Type	Caption	List Options	Abstraction Instruction
p_cacc	textbox	CACC		ACR/AACR - Administration - CACC *<br><br>NOTE: If left blank, please attempt to fill in using ''Service Name'' under the ''Administration section''.<br><br>As per *Emergency Health Services Branch Directory of Ambulance System Services, July 11, 2003* by the Ministry of Health and Long-Term Care.
p_svcnum	dropdown	Service Number	listid: p_services<br>** See list items in appendix	ACR - Administration – Service<br><br>AACR - not a field; please transcribe 0818<br><br>In the case of multiple vehicles, use the ACR that was completed by the EMS crew that treated and transported the patient.<br><br>EMS is 3 digits and Fire is 4 digits.<br><br>Land EMS:<br><br>262. Sault Ste Marie EMS<br><br>491. Toronto EMS<br><br>701. Brant EMS<br><br>701. Grey EMS<br><br>720. Muskoka EMS<br><br>722. Norfolk EMS<br><br>724. Durham EMS<br><br>727. Peel EMS<br><br>733. Simcoe EMS<br><br>738. Hamilton EMS<br><br>740. Algoma EMS

PREDICT - Prehospital Variables

Variable	Type	Caption	List Options	Abstraction Instruction
				747. Sudbury EMS
				749. Thunder Bay EMS (Superior North EMS)
				752. Manitoulin-Sudbury EMS (East Side)
				753. Haldimand EMS
				782. Manitoulin-Sudbury EMS (West Side)
				789. York EMS
				818. OAASC
				1801. Durham Fire Pickerin
				1805. Durham Fire Ajax
				1809. Durham Fire Whitby
				1813. Durham Fire Oshawa
				1817. Durham Fire Claringt
				1820. Durham Fire Scugog
				1829. Durham Fire Uxbridge
				1839. Durham Fire Brock
				2005. Toronto Fire
				2105. Peel Fire Mississaug
				2110. Peel Fire Brampton
				2124. Peel Fire Caledon
				2505. Hamilton Fire
				4307. Simcoe Fire New Tec
				4312. Simcoe Fire Bradford
				4317. Simcoe Fire Innisfil
				4319. Simcoe Fire Ramara
				4321. Simcoe Fire Essa

PREDICT - Prehospital Variables

Variable	Type	Caption	List Options	Abstraction Instruction
				4323. Simcoe Fire Oro Med<br><br>4342. Simcoe Fire Barrie<br><br>4350. Simcoe Fire Mnjikani<br><br>4352. Simcoe Fire Orillia<br><br>4364. Simcoe Fire Wasaga<br><br>4465. Muskoka Fire Georg<br><br>9999 =Other<br><br>Null=Not available/missing
p_vehnum	dropdown	Vehicle Number	listid: rigs	ACR – Administration - Vehicle Number<br><br>CAD Veh<br><br>AACR - Administration – A/C reg./Veh #<br><br>Transcribe as-is<br><br>In the case of multiple vehicles, use the ACR that was completed by the EMS crew that treated and transported or pronounced the patient.<br><br>There are multiple ways that the crew records vehicle number. This field is for local purposes only.
p_calltype	dropdown	Call Type	listid: calltype<br><br>1. bls<br>2. sr<br>3. aed<br>4. iv only<br><br>5. als<br>6. cct<br>7. not noted	ACR - small print top right corner page 1 - Call Type<br><br>AACR - not a field<br><br>In the case of multiple vehicles, use the ACR that was completed by the EMS crew that treated and transported the patient.
p_vehstation	textbox	Station		ACR - Administration - Station

PREDICT - Prehospital Variables

Variable	Type	Caption	List Options	Abstraction Instruction
				AACR - Administration – Base<br>Air code is 3 digit numeric (e.g. 799 for Toronto)<br><br>In the case of multiple vehicles, use the ACR that was completed by the EMS crew that treated and transported the patient.
p_vehstatus	dropdown	Status	listid: vehloc<br>0. At Base<br>77. Mobile<br>88. Standby loc.<br>99. Maintenance	ACR/AACR - Administration – Status<br><br>**A:** In the case of multiple vehicles, use the ACR that was completed by the EMS crew that treated and transported the patient.
p_crew1no	textbox	Crew Member 1 (Attendant) No		ACR/AACR – Crew Member 1<br>RDC - Crew Member 1<br><br>ACR - General Administration – Crew Member 1 (Attendant) No. 1<br><br>Transcribe as-is<br><br>In the case of multiple vehicles, use the ACR that was completed by the EMS crew that treated and transported the patient.<br><br>Note: Crew Member 1 is the medic to contact for episode clarification.
p_crew2no	textbox	Crew Member 2 (Attendant) No		ACR/AACR – Crew Member 2<br>RDC - Crew Member 2<br><br>ACR - General Administration – Crew Member 2 (Attendant) No. 2<br><br>Transcribe as-is<br><br>In the case of multiple vehicles, use the ACR that was completed by the EMS crew that treated and transported the patient.<br><br>Note: Crew Member 1 is the medic to contact for episode clarification.
p_crew3no	textbox	Crew Member 3 (Attendant) No		ACR/AACR – Crew Member 3<br>RDC - Crew Member 3<br><br>ACR - General Administration – Crew Member 3 (Attendant) No. 3<br><br>Transcribe as-is

PREDICT - Prehospital Variables

Variable	Type	Caption	List Options	Abstraction Instruction
				In the case of multiple vehicles, use the ACR that was completed by the EMS crew that treated and transported the patient.<br><br>Note: Crew Member 1 is the medic to contact for episode clarification.
p_crew4no	textbox	Crew Member 4 (Attendant) No		ACR/AACR – Crew Member 4<br>RDC - Crew Member 4<br><br>ACR - General Administration – Crew Member 4 (Attendant) No. 4<br><br>Transcribe as-is<br><br>In the case of multiple vehicles, use the ACR that was completed by the EMS crew that treated and transported the patient.<br><br>Note: Crew Member 1 is the medic to contact for episode clarification.
**p_pph**	**section**	**Patient Past History**		
p_phx	dropdown	Patient Past History	listid: nyn<br><br>0. no<br>1. yes<br><br>2. not noted	Past History (from ACR - do not use ED/Hospital records).<br><br>If no past history is noted on the ACR, choose not noted.<br><br>Previously healthy<br>Cardiac<br>Afib/Flutter – atrial fibrillation/flutter<br><br>MI - myocardial infarction<br>CAD - coronary artery disease<br>HTN - hypertension<br>CHF - congestive heart failure<br>Syncope<br>Cardiac medications (e.g., digoxin, beta-blockers, antiarrhythmics, nitrates, etc.)<br><br>Respiratory<br>Stroke/TIA - transitory ischemic attack<br><br>Hypertension<br>Seizure<br>Diabetes<br>Psychiatric<br>Heart surgery (other than CABG which is noted above)<br>ICD - implantable cardiodefibrillator<br><br>CABG - coronary artery bypass graph<br>Pacemaker<br>Other surgery<br>Recreational drugs

Variable	Type	Caption	List Options	Abstraction Instruction
				Alcohol abuse<br>Cancer<br>Other: (fill in) - indicate any other significant medical history noted<br><br>Specify other not listed
p_phphlt	dropdown	Previously Healthy	listid: nyn<br><br>0. no<br>1. yes<br><br>2. not noted	Past History (from ACR - do not use ED/Hospital records).<br><br>If no past history is noted on the ACR, choose not noted.
p_phcar	dropdown	Cardiac	listid: nyn<br><br>0. no<br>1. yes<br><br>2. not noted	Past History (from ACR - do not use ED/Hospital records).<br><br>If no past history is noted on the ACR, choose not noted.
p_phafi	dropdown	Afib/Flutter	listid: nyn<br><br>0. no<br>1. yes<br><br>2. not noted	Past History (from ACR - do not use ED/Hospital records).<br><br>If no past history is noted on the ACR, choose not noted.
p_phmi	dropdown	MI	listid: nyn<br><br>0. no<br>1. yes<br><br>2. not noted	Past History (from ACR - do not use ED/Hospital records).<br><br>If no past history is noted on the ACR, choose not noted.
p_phcad	dropdown	CAD	listid: nyn<br><br>0. no<br>1. yes<br><br>2. not noted	Past History (from ACR - do not use ED/Hospital records).<br><br>If no past history is noted on the ACR, choose not noted.
p_phchf	dropdown	CHF	listid: nyn<br><br>0. no<br>1. yes<br><br>2. not noted	Past History (from ACR - do not use ED/Hospital records).<br><br>If no past history is noted on the ACR, choose not noted.
p_phsyn	dropdown	Syncope	listid: nyn<br><br>0. no<br>1. yes<br><br>2. not noted	Past History (from ACR - do not use ED/Hospital records).<br><br>If no past history is noted on the ACR, choose not noted.
p_phcam	dropdown	Cardiac Medications	listid: nyn<br><br>0. no<br>1. yes<br><br>2. not noted	Past History (from ACR - do not use ED/Hospital records).<br><br>If no past history is noted on the ACR, choose not noted.
p_phrsp	dropdown	Respiratory	listid: nyn	Past History (from ACR - do not use ED/Hospital records).

Variable	Type	Caption	List Options	Abstraction Instruction
			0. no<br>1. yes<br>2. not noted	If no past history is noted on the ACR, choose not noted.
p_phstr	dropdown	Stroke/TIA	listid: nyn<br>0. no<br>1. yes<br>2. not noted	Past History (from ACR - do not use ED/Hospital records).<br>If no past history is noted on the ACR, choose not noted.
p_phhyp	dropdown	Hypertension	listid: nyn<br>0. no<br>1. yes<br>2. not noted	Past History (from ACR - do not use ED/Hospital records).<br>If no past history is noted on the ACR, choose not noted.
p_phsei	dropdown	Seizure	listid: nyn<br>0. no<br>1. yes<br>2. not noted	Past History (from ACR - do not use ED/Hospital records).<br>If no past history is noted on the ACR, choose not noted.
p_phdia	dropdown	Diabetes	listid: nyn<br>0. no<br>1. yes<br>2. not noted	Past History (from ACR - do not use ED/Hospital records).<br>If no past history is noted on the ACR, choose not noted.
p_phpsy	dropdown	Psychiatric	listid: nyn<br>0. no<br>1. yes<br>2. not noted	Past History (from ACR - do not use ED/Hospital records).<br>If no past history is noted on the ACR, choose not noted.
p_phhs	dropdown	Heart Surgery	listid: nyn<br>0. no<br>1. yes<br>2. not noted	Past History (from ACR - do not use ED/Hospital records).<br>If no past history is noted on the ACR, choose not noted.
p_phicd	dropdown	ICD	listid: nyn<br>0. no<br>1. yes<br>2. not noted	Past History (from ACR - do not use ED/Hospital records).<br>If no past history is noted on the ACR, choose not noted.
p_phcabg	dropdown	CABG	listid: nyn<br>0. no<br>1. yes<br>2. not noted	Past History (from ACR - do not use ED/Hospital records).<br>If no past history is noted on the ACR, choose not noted.
p_phpac	dropdown	Pacemaker	listid: nyn<br>0. no<br>1. yes	Past History (from ACR - do not use ED/Hospital records).<br>If no past history is noted on the ACR, choose not noted.

Variable	Type	Caption	List Options	Abstraction Instruction
			2. not noted	
p_phos	dropdown	Other Surgery	listid: nyn<br><br>0. no<br>1. yes<br><br>2. not noted	Past History (from ACR - do not use ED/Hospital records).<br><br>If no past history is noted on the ACR, choose not noted.
p_phrec	dropdown	Recreational Drugs	listid: nyn<br><br>0. no<br>1. yes<br><br>2. not noted	Past History (from ACR - do not use ED/Hospital records).<br><br>If no past history is noted on the ACR, choose not noted.
p_phaa	dropdown	Alcohol Abuse	listid: nyn<br><br>0. no<br>1. yes<br><br>2. not noted	Past History (from ACR - do not use ED/Hospital records).<br><br>If no past history is noted on the ACR, choose not noted.
p_phcan	dropdown	Cancer	listid: nyn<br><br>0. no<br>1. yes<br><br>2. not noted	Past History (from ACR - do not use ED/Hospital records).<br><br>If no past history is noted on the ACR, choose not noted.
p_photh	dropdown	Other	listid: nyn<br><br>0. no<br>1. yes<br><br>2. not noted	Past History (from ACR - do not use ED/Hospital records).<br><br>If no past history is noted on the ACR, choose not noted.
p_phospc	textbox	Specify Other Not listed		Past History (from ACR - do not use ED/Hospital records).<br><br>If no past history is noted on the ACR, choose not noted.
**p_pmh**	**section**	**Patient Medication History**		
p_mhx	dropdown	Patient Past Medication History	listid: nyn<br><br>0. no<br>1. yes<br><br>2. not noted	Patient Medication History (from ACR — do not use ED/Hospital records).<br><br>If no patient medication history is noted on the ACR, choose not noted.<br><br>None<br>Nitrates<br>Digoxin<br>Insulin<br>ASA - acetyl salicylic acid (Aspirin)<br><br>Ventolin<br>Oral diabetic medication<br>Lasix<br>OCP - oral contraceptive pills

Variable	Type	Caption	List Options	Abstraction Instruction
				Other<br>Specify other not listed<br>Not determined
p_mhno	dropdown	None	listid: nyn<br>0. no<br>1. yes<br>2. not noted	Patient Medication History (from ACR - do not use ED/Hospital records).<br>If no patient medication history is noted on the ACR, choose not noted.
p_mhnit	dropdown	Nitrates	listid: nyn<br>0. no<br>1. yes<br>2. not noted	Patient Medication History (from ACR - do not use ED/Hospital records).<br>If no patient medication history is noted on the ACR, choose not noted.
p_mhdig	dropdown	Digoxin	listid: nyn<br>0. no<br>1. yes<br>2. not noted	Patient Medication History (from ACR - do not use ED/Hospital records).<br>If no patient medication history is noted on the ACR, choose not noted.
p_mhins	dropdown	Insulin	listid: nyn<br>0. no<br>1. yes<br>2. not noted	Patient Medication History (from ACR - do not use ED/Hospital records).<br>If no patient medication history is noted on the ACR, choose not noted.
p_mhasa	dropdown	ASA	listid: nyn<br>0. no<br>1. yes<br>2. not noted	Patient Medication History (from ACR - do not use ED/Hospital records).<br>If no patient medication history is noted on the ACR, choose not noted.
p_mhven	dropdown	Ventolin	listid: nyn<br>0. no<br>1. yes<br>2. not noted	Patient Medication History (from ACR - do not use ED/Hospital records).<br>If no patient medication history is noted on the ACR, choose not noted.
p_mhodm	dropdown	Oral Diabetic Medication	listid: nyn<br>0. no<br>1. yes<br>2. not noted	Patient Medication History (from ACR - do not use ED/Hospital records).<br>If no patient medication history is noted on the ACR, choose not noted.
p_mhlas	dropdown	Lasix	listid: nyn<br>0. no<br>1. yes<br>2. not noted	Patient Medication History (from ACR - do not use ED/Hospital records).<br>If no patient medication history is noted on the ACR, choose not noted.
p_mhocp	dropdown	OCP	listid: nyn<br>0. no<br>1. yes<br>2. not noted	Patient Medication History (from ACR - do not use ED/Hospital records).<br>If no patient medication history is noted on the ACR, choose not noted.

PREDICT - Prehospital Variables

Variable	Type	Caption	List Options	Abstraction Instruction
p_mhoth	dropdown	Other	listid: nyn<br>0. no<br>1. yes<br>2. not noted	Patient Medication History (from ACR - do not use ED/Hospital records).<br>If no patient medication history is noted on the ACR, choose not noted.
p_mhospc	textbox	Specify Other Not Listed		Patient Medication History (from ACR – do not use ED/Hospital records).<br>If no patient medication history is noted on the ACR, choose not noted.
p_mhndet	dropdown	Not Determined	listid: nyn<br>0. no<br>1. yes<br>2. not noted	Patient Medication History: (from ACR – do not use ED/Hospital records).<br>The ACR selection of not determined implies the medic did not ask as opposed to not noted which implies the abstractor could not find the information.
**p_ppal**	**section**	**Patient Allergies**		
p_pal	dropdown	Patient Allergies	listid: nynd<br>0. no<br>1. yes<br>2. not noted<br>3. not determined	Patient Allergies (from ACR – do not use ED/Hospital records).<br>If no patient allergy history is noted on the ACR, choose not noted.<br>NKA - no known allergies<br>ASA - acetyl salicylic acid (Aspirin)<br>Sulpha – sulpha medications<br>Penicillin<br>Codein<br>Other<br>Not determined
p_palnka	dropdown	NKA	listid: nyn<br>0. no<br>1. yes<br>2. not noted	Patient Allergies (from ACR - do not use ED/Hospital records).<br>If no patient allergy history is noted on the ACR, choose not noted.
p_palasa	dropdown	ASA	listid: nyn<br>0. no<br>1. yes<br>2. not noted	Patient Allergies (from ACR - do not use ED/Hospital records).<br>If no patient allergy history is noted on the ACR, choose not noted.
p_palsul	dropdown	Sulpha	listid: nyn<br>0. no<br>1. yes<br>2. not noted	Patient Allergies (from ACR - do not use ED/Hospital records).<br>If no patient allergy history is noted on the ACR, choose not noted.
p_palpen	dropdown	Penicillin	listid: nyn	Patient Allergies (from ACR - do not use ED/Hospital records).

PREDICT - Prehospital Variables

Variable	Type	Caption	List Options	Abstraction Instruction
			0. no<br>1. yes<br>2. not noted	If no patient allergy history is noted on the ACR, choose not noted.
p_palcod	dropdown	Codein	listid: nyn<br>0. no<br>1. yes<br>2. not noted	Patient Allergies (from ACR - do not use ED/Hospital records).<br>If no patient allergy history is noted on the ACR, choose not noted.
p_paloth	dropdown	Other	listid: nyn<br>0. no<br>1. yes<br>2. not noted	Patient Allergies (from ACR - do not use ED/Hospital records).<br>If no patient allergy history is noted on the ACR, choose not noted.
p_palspc	textbox	Specify Other Not Listed		Patient Allergies (from ACR – do not use ED/Hospital records).<br>If no patient allergy history is noted on the ACR, choose not noted.
p_palndet	dropdown	Not Determined	listid: nyn<br>0. no<br>1. yes<br>2. not noted	Patient Allergies (from ACR – do not use ED/Hospital records).<br>The ACR selection of not determined implies the medic did not ask as opposed to not noted which implies the abstractor could not find the information.
**p_pptr**	**section**	**Prehospital Time Record**		
p_ptr	dropdown	Prehospital Time Record	listid: nyn<br>0. no<br>1. yes<br>2. not noted	Is 'Prehospital Time Record' reported on source document?<br>0=no<br>1=yes<br>2=not noted
p_air	dropdown	Was Air Ambulance Involved?	listid: ny<br>0. no<br>1. yes	*Was Air Ambulance involved in the care of patient in the prehospital setting?*<br>*0=no*<br>*1=yes*
p_cr911	dropdown	Call Received at 911	listid: nyn<br>0. no<br>1. yes<br>2. not noted	Is 'Call Received at 911' time documented on souce document?<br>0=no<br>1=yes<br>2=not noted
p_cr911_pri	div	Primary		

PREDICT - Prehospital Variables

Variable	Type	Caption	List Options	Abstraction Instruction
p_psr911	dropdown	Source	listid: src<br><br>1. acr<br>2. cad<br>3. mar<br>4. aacr	Source for 'Call Received at 911' time.<br><br>1 = acr<br>2 = cad<br>3 = MAR<br>4 = aacr
p_ptm911	textbox	Time		911 Call Centre - Time of the earliest call received at the initial public safety answering point.<br><br>Indicate the time that the call was received at the 911 Call Centre.<br><br>Note: This time is different then “Call Received” Time (comes from EMS or Fire CAD).
p_cr911_alt	div	Alternate		
p_asr911	dropdown	Source	listid: src<br><br>1. acr<br>2. cad<br>3. mar<br>4. aacr	Alternative source for 'Call Received at 911' time – record if different than primary source.<br><br>1=acr<br>2=cad<br>3=mar<br>4=aacr
p_atm911	textbox	Time		Alternative value for 'Call Received at 911' – record if different than primary source.<br><br>Numerical value based on 24 hour clock<br><br>00:00:00 – hour:min:sec<br><br>If no value for seconds data available – do not do not use value 00.
p_cr911_det	div	Doc/Est Time		
p_ndt911	dropdown	Doc/Est Time	listid: notime<br><br>-9999. doc time<br>1. est time	Documented/Estimated time data available for 'Call Received at 911'.<br><br>Doc time - Documented time exists for 'Call Received at 911' 1=est time - Estimated time exists for 'Call Received at 911'
p_etm911	textbox	Est Time		Alternative value for ''Call Recieved at 911'' time - record if different than primary source.<br><br>Numerical value based on 24 hour clock<br><br>00:00:00 – hour:min:sec<br><br>If no value for seconds data available – do not do not use value 00.

PREDICT - Prehospital Variables

Variable	Type	Caption	List Options	Abstraction Instruction
p_prcv	dropdown	Call Received At Dispatch	listid: nyn<br><br>0. no<br>1. yes<br>2. not noted	Is 'Call Received at Dispatch' time reported on source document?<br><br>0=no<br>1=yes<br>2=not noted
p_prcv_pri	div	Primary		
p_psrrcv	dropdown	Source	listid: src<br><br>1. acr<br>2. cad<br>3. mar<br>4. aacr	Source for time 'Call Received at Dispatch'.<br><br>1 = acr<br>2 = cad<br>3 = MAR<br>4 = aacr
p_ptmrcv	textbox	Time		*This field is a replication of field number 2 for ease of EMS times interpretation.*<br><br>911 Call Centre - Time of the earliest call received at the emergency communication center responsible for dispatching a vehicle as part of the EMS organized response; includes all organized EMS respondents (i.e. fire and paramedics)<br><br>Indicate the time that the call was received at the 911 Call Centre.<br><br>Note: This time is different then “Call Received” Time (comes from EMS or Fire CAD).
p_prcv_alt	div	Alternate		
p_asrrcv	dropdown	Source	listid: src<br><br>1. acr<br>2. cad<br>3. mar<br>4. aacr	Alternative source for 'Call Received at Dispatch' time – record if different than primary source.<br><br>1=acr<br>2=cad<br>3=mar<br>4=aacr
p_atmrcv	textbox	Time		Alternative value for 'Call Received at Dispatch' time – record if different than primary source.<br><br>Numerical value based on 24 hour clock<br><br>00:00:00 – hour:min:sec<br><br>If no value for seconds data available – do not do not use value 00.
p_prcv_det	div	Doc/Est Time		

PREDICT - Prehospital Variables

Variable	Type	Caption	List Options	Abstraction Instruction
p_ndtrcv	dropdown	Doc/Est Time	listid: notime<br><br>-9999. doc time<br>1. est time	Documented/Estimated time data available for 'Call Received at Dispatch'.<br><br>Doc time - Documented time exists for ''Call Recieved at Dispatch''<br>1=est time - Estimated time exists for 'Call Received at Dispatch'
p_etmrcv	textbox	Est Time		Alternative value for 'Call Received at Dispatch' – record if different than primary source.<br><br>Numerical value based on 24 hour clock<br><br>00:00:00 – hour:min:sec<br><br>If no value for seconds data available – do not do not use value 00.
p_pcat	dropdown	Call Accepted (air only)	listid: nyn<br><br>0. no<br>1. yes<br>2. not noted	Is 'Call Accepted' time reported on source document?<br><br>0=no<br>1=yes<br>2=not noted
p_pcat_pri	div	Primary		
p_psrcat	dropdown	Source	listid: src<br>1. acr<br>2. cad<br>3. mar<br>4. aacr	Source for time 'Call Accepted'.<br><br>1 = acr<br>2 = cad<br>3 = MAR<br>4 = aacr
p_ptmcat	textbox	Time		Call accepted time.<br><br>Indicate the time that the call was accepted.
p_pcat_det	div	Doc/Est Time		
p_etmcat	textbox	Est Time		Alternative value for 'Call Accepted' time.<br><br>Numerical value based on 24 hour clock<br><br>00:00:00 – hour:min:sec<br><br>If no value for seconds data available – do not do not use value 00.
p_pdb	dropdown	Depart Base (air only)	listid: nyn<br><br>0. no<br>1. yes<br>2. not noted	Is 'Depart Base' time reported on source document?<br><br>0=no<br>1=yes<br>2=not noted
p_pdb_pri	div	Primary		
p_psrdpb	dropdown	Source	listid: src	Source for time ''Depart Base''.

PREDICT - Prehospital Variables

Variable	Type	Caption	List Options	Abstraction Instruction
			1. acr<br>2. cad<br>3. mar<br>4. aacr	1 = acr<br>2 = cad<br>3 = MAR<br>4 = aacr
p_ptmdpb	textbox	Time		Depart base time.<br><br>Indicate the depart base time.
p_pdsp	dropdown	1st Vehicle Dispatched	listid: nyn<br><br>0. no<br>1. yes<br>2. not noted	Is '1st Vehicle Dispatched' time reported on source document?<br><br>0=no<br>1=yes<br>2=not noted
p_pdsp_pri	div	Primary		
p_psrdsp	dropdown	Source	listid: src<br><br>1. acr<br>2. cad<br>3. mar<br>4. aacr	Source for time '1st Vehicle Dispatched'.<br><br>1 = acr<br>2 = cad<br>3 = MAR<br>4 = aacr
p_ptmdsp	textbox	Time		CAD - Crew Ntf - The time recorded when the crew of the first dispatched responding vehicle was notified. The 1st dispatch time may or may not be associated with the vehicle that is 1st arrival at scene.<br><br>Data Guardian abstracts from appropriate CAD. If not available obtain from ACR “Call Events”.<br><br>NOTE: Air does not have a crew notified time.<br><br>Time when the first arriving vehicle was notified by dispatch.
p_pdsp_alt	div	Alternate		
p_asrdsp	dropdown	Source	listid: src<br><br>1. acr<br>2. cad<br>3. mar<br>4. aacr	Alternative source for '1st Vehicle Dispatched' time – record if different than primary source.<br><br>1=acr<br>2=cad<br>3=mar<br>4=aacr
p_atmdsp	textbox	Time		Alternative value for '1st Vehicle Dispatched' time – record if different than primary source.<br><br>Numerical value based on 24 hour clock<br><br>00:00:00 – hour:min:sec

PREDICT - Prehospital Variables

Variable	Type	Caption	List Options	Abstraction Instruction
				If no value for seconds data available – do not do not use value 00.
p_pdsp_det	div	Doc/Est Time		
p_ndtdsp	dropdown	Doc/Est Time	listid: notime<br><br>-9999. doc time<br>1. est time	Documented/Estimated time data available for '1st Vehicle Dispatched'.<br><br>Doc time - Documented time exists for ''1st Vehicle Dispatched''<br>1=est time - Estimated time exists for '1st Vehicle Dispatched'
p_etmdsp	textbox	Est Time		Alternative value for '1st Vehicle Dispatched' – record if different than primary source.<br><br>Numerical value based on 24 hour clock<br><br>00:00:00 – hour:min:sec<br><br>If no value for seconds data available – do not do not use value 00.
p_papl	dropdown	Arrive Pick-Up Land (air only)	listid: nyn<br>0. no<br>1. yes<br>2. not noted	Is ''''Arrive Pick-Up Land'''' time reported on source document?<br><br>0=no<br>1=yes<br>2=not noted
p_papl_pri	div	Primary		
p_psrapl	dropdown	Source	listid: src<br>1. acr<br>2. cad<br>3. mar<br>4. aacr	Source for 'Arrive Pick-Up Land' time.<br><br>1 = acr<br>2 = cad<br>3 = MAR<br>4 = aacr
p_ptmapl	textbox	Time		Arrive pick-up land time.<br><br>Indicate the arrive pick-up land time.
p_papl_det	div	Doc/Est Time		
p_etmapl	textbox	Est Time		Alternative value for 'Arrive Pick-Up Land' – record if different than primary source.<br><br>Numerical value based on 24 hour clock<br><br>00:00:00 – hour:min:sec<br><br>If no value for seconds data available – do not do not use value 00.
p_paer	dropdown	Arrive Patient Site	listid: nyn<br>0. no<br>1. yes<br>2. not noted	Is 'Arrive Patient Site' time reported on source document?<br><br>0=no<br>1=yes

PREDICT - Prehospital Variables

Variable	Type	Caption	List Options	Abstraction Instruction
				2=not noted
p_paer_pri	div	Primary		
p_psraer	dropdown	Source	listid: src<br>1. acr<br>2. cad<br>3. mar<br>4. aacr	Source for time 'Arrive Patient Site'.<br><br>1 = acr<br>2 = cad<br>3 = MAR<br>4 = aacr
p_ptmaer	textbox	Time		Arrive patient site time - The time recorded when the crew of the air responding service arrived at patient site.<br><br>Indicate the depart base time.
p_paer_alt	div	Alternate		
p_asraer	dropdown	Source	listid: src<br><br>1. acr<br>2. cad<br>3. mar<br>4. aacr	Alternative source for 'Arrive Patient Site' time – record if different than primary source.<br><br>1=acr<br>2=cad<br>3=mar<br>4=aacr
p_atmaer	textbox	Time		Alternative value for 'Arrive Patient Site' time – record if different than primary source.<br><br>Numerical value based on 24 hour clock<br><br>00:00:00 – hour:min:sec<br><br>If no value for seconds data available – do not do not use value 00.
p_paer_det	div	Doc/Est Time		
p_ndtaer	dropdown	Doc/Est Time	listid: notime<br><br>-9999. doc time<br>1. est time	Documented/Estimated time data available for 'Arrive Patient Site'.<br><br>Doc time - Documented time exists for ''Arrive Patient Site''<br>1=est time - Estimated time exists for 'Arrive Patient Site'
p_etmaer	textbox	Est Time		Alternative value for 'Arrive Patient Site' time – record if different than primary source.<br><br>Numerical value based on 24 hour clock<br><br>00:00:00 – hour:min:sec

PREDICT - Prehospital Variables

Variable	Type	Caption	List Options	Abstraction Instruction
				If no value for seconds data available – do not do not use value 00.
p_parr	dropdown	1st Vehicle Arrive at Scene	listid: nyn<br><br>0. no<br>1. yes<br>2. not noted	Is '1st Vehicle Arrive at Scene' time reported on source document?<br><br>0=no<br>1=yes<br>2=not noted
p_parr_pri	div	Primary		
p_psrarr	dropdown	Source	listid: src<br><br>1. acr<br>2. cad<br>3. mar<br>4. aacr	Source for time '1st Vehicle Arrive at Scene'.<br>1 = acr<br>2 = cad<br>3 = MAR<br>4 = aacr
p_ptmarr	textbox	Time		CAD – Arr. Scene - Time the first responding vehicle arrives on the scene, wheels stopped.<br><br>Data Guardian abstracts from appropriate CAD. If not available obtain from ACR "Call Events"<br><br>Time when the first arriving vehicle arrived scene.
p_parr_alt	div	Alternate		
p_asrarr	dropdown	Source	listid: src<br><br>1. acr<br>2. cad<br>3. mar<br>4. aacr	Alternative source for '1st Vehicle Arrive at Scene' time – record if different than primary source.<br><br>1=acr<br>2=cad<br>3=mar<br>4=aacr
p_atmarr	textbox	Time		Alternative value for '1st Vehicle Arrive at Scene' time – record if different than primary source.<br><br>Numerical value based on 24 hour clock<br><br>00:00:00 – hour:min:sec<br><br>If no value for seconds data available – do not do not use value 00.
p_parr_det	div	Doc/Est Time		
p_ndtarr	dropdown	Doc/Est Time	listid: notime<br><br>-9999. doc time<br>1. est time	Documented/Estimated time data available for '1st Vehicle Arrive at Scene'.<br><br>Doc time - documented time exists for ''1st Vehicle Arrive at Scene''<br>1=est time - Estimated time exists for '1st Vehicle Arrive at Scene'

PREDICT - Prehospital Variables

Variable	Type	Caption	List Options	Abstraction Instruction
p_etmarr	textbox	Est Time		Alternative value for '1st Vehicle Arrive at Scene' time – record if different than primary source.<br><br>Numerical value based on 24 hour clock<br><br>00:00:00 – hour:min:sec<br><br>If no value for seconds data available – do not do not use value 00.
p_pals	dropdown	1st ALS Vehicle Arrive at Scene	listid: nyn<br><br>0. no<br>1. yes<br>2. not noted	Is '1 st ALS Vehicle Arrive at Scene' time reported on source document?<br><br>0=no<br>1=yes<br>2=not noted
p_pals_pri	div	Primary		
p_psrals	dropdown	Source	listid: src<br><br>1. acr<br>2. cad<br>3. mar<br>4. aacr	Source for time '1 st ALS Vehicle Arrive at Scene'.<br>1 = acr<br>2 = cad<br>3 = MAR<br>4 = aacr
p_ptmals	textbox	Time		CAD – Arr. Scene - Time when the first arriving vehicle arrived scene.<br><br>Data Guardian abstracts from appropriate CAD. If not available obtain from ACR "Call Events"<br><br>Time when the first arriving vehicle arrived scene.
p_pals_alt	div	Alternate		
p_asrals	dropdown	Source	listid: src<br><br>1. acr<br>2. cad<br>3. mar<br>4. aacr	Alternative source for '1st ALS Vehicle Arrive at Scene' time – record if different than primary source.<br><br>1=acr<br>2=cad<br>3=mar<br>4=aacr
p_atmals	textbox	Time		Alternative value for '1st ALS Vehicle Arrive at Scene' time – record if different than primary source.<br><br>Numerical value based on 24 hour clock<br><br>00:00:00 – hour:min:sec<br><br>If no value for seconds data available – do not do not use value 00.

PREDICT - Prehospital Variables

Variable	Type	Caption	List Options	Abstraction Instruction
p_pals_det	div	Doc/Est Time		
p_ndtals	dropdown	Doc/Est Time	listid: notime<br><br>-9999. doc time<br>1. est time	Documented/Estimated time data available for '1st ALS Vehicle Arrive at Scene'.<br><br>Doc time - Documented time exists for ''1st ALS Vehicle Arrive at Scene'<br>1=est time - Estimated time exists for '1st ALS Vehicle Arrive at Scene'
p_etmals	textbox	Est Time		Alternative value for '1st ALS Vehicle Arrive at Scene' time – record if different than primary source.<br><br>Numerical value based on 24 hour clock<br><br>00:00:00 – hour:min:sec<br><br>If no value for seconds data available – do not do not use value 00.
p_paed	dropdown	1st EMS AED/Defib Turned On	listid: nyn<br><br>0. no<br>1. yes<br>2. not noted	Is '1st AED Defib Turned On' time reported on source document?<br><br>0=no<br>1=yes<br>2=not noted
p_paed_pri	div	Primary		
p_psraed	dropdown	Source	listid: srcdef<br><br>1. acr<br>2. cad<br>3. mar<br>4. aacr<br>5. defib	Source for time '1st AED Defib Turned On'.<br><br>1 = acr<br>2 = cad<br>3 = MAR<br>4 = aacr<br>5 = defibrillator (printed out summary)
p_ptmaed	textbox	Time		ACR - Clinical Treatment / Procedures & Results<br>Time - The time the EMS responder powers on the automatic external defibrillator (AED) or monitor/defibrillator.<br><br>also reference<br><br>ECG<br><br>Indicate the time of the rhythm.
p_paed_fib	div	Defib		
p_dsraed	dropdown	Source	listid: aedno<br>1. ECG 1<br>2. ECG 2<br>3. ECG 3	What Defib/AED provided the time used?<br><br>1=1st AED/Defib Applied<br>2=2nd or greater

Variable	Type	Caption	List Options	Abstraction Instruction
				ACR/AACR Possible sources;<br>Incident History<br>Treatment Prior to Arrival<br>Physical Exam – General Appearance<br>Clinical Treatment / Procedures & Results – 301 (Cardiac Monitor)<br>Clinical Treatment / Procedures & Results – 306 (Defibrillation Manual)<br>Clinical Treatment / Procedures & Results – 307 (Defibrillation Semi-Auto)<br>Clinical Treatment / Procedures & Results – 308 (Automatic)
p_synaed	dropdown	Synched?	listid: aedsyn<br>-9999. no<br>1. yes	Does the Defib/AED appear synchronized with the Atomic Clock?<br>null=no<br>1=yes<br>For example; If defib times appear 1 hour ahead or behind during daylight savings time it can be assumed that they have not yet been re-synchronized = NO.<br>Another example (From MOO): a defibrillator provides a time that is 5 minutes different than a Watch time documented for the same ordered event; you know the EMS agency synchronizes monthly; you understand that wristwatch and clock times are often subject to drift and sloppy setting; therefore you conclude that the defibrillator DOES appear synchronized and you DO mark the corresponding box for ''''Defib Appears Synched....''''.
p_paed_alt	div	Alternate		
p_asraed	dropdown	Source	listid: srcdef<br>1. acr<br>2. cad<br>3. mar<br>4. aacr<br>5. defib	Alternative source for '1st AED Defib Turned On' time – record if different than primary source.<br>1=acr<br>2=cad<br>3=mar<br>4=aacr<br>5 = defibrillator (printed out summary)
p_atmaed	textbox	Time		Alternative value for '1st AED Defib Turned On' time – record if different than primary source.

PREDICT - Prehospital Variables

Variable	Type	Caption	List Options	Abstraction Instruction
				Numerical value based on 24 hour clock<br><br>00:00:00 – hour:min:sec<br><br>If no value for seconds data available – do not do not use value 00.
p_paed_det	div	Doc/Est Time		
p_ndtaed	dropdown	Doc/Est Time	listid: notime<br><br>-9999. doc time<br>1. est time	Documented/Estimated time data available for '1st AED Defib Turned On'.<br><br>Doc time - Documented time exists for ''1st AED Defib Turned On''<br>1=est time - Estimated time exists for '1st AED Defib Turned On'
p_etmaed	textbox	Est Time		Alternative value for '1st AED Defib Turned On' time – record if different than primary source.<br><br>Numerical value based on 24 hour clock<br><br>00:00:00 – hour:min:sec<br><br>If no value for seconds data available – do not do not use value 00.<br>.
p_ppad	dropdown	Pads Placed	listid: nyn<br><br>0. no<br>1. yes<br>2. not noted	Is the time when organized EMS personnel including fire first placed defibrillator pads onto the patient reported on source document?<br><br>0=no<br>1=yes<br>2=not noted
p_ppad_pri	div	Primary		
p_psrpad	dropdown	Source	listid: srcdef<br><br>1. acr<br>2. cad<br>3. mar<br>4. aacr<br>5. defib	The time when organized EMS personnel including fire first placed defibrillator pads onto the patient.<br><br>Source of that time;<br><br>1 = acr<br>2 = cad<br>3 = MAR<br>4 = aacr<br>5 = defibrillator (printed out summary)<br><br>ACR/AACR source – Clinical Procedures/Treatments & Results – Treatment prior to arrival.

Variable	Type	Caption	List Options	Abstraction Instruction
				Defibrillator summary source – 'PADS PLACED' time<br><br>May NOT be available for every patient.
p_ptmpad	textbox	Time		ACR - Clinical Treatment / Procedures & Results<br>Time.<br><br>also reference<br><br>ECG<br><br>When EMS responder powers on the automatic external defibrillator (AED) or monitor/defibrillator.<br><br>Indicate the time of the rhythm.
p_ppad_det	div	Doc/Est Time		
p_etmpad	textbox	Est Time		Alternative value for the time when organized EMS personnel including fire first placed defibrillator pads onto the patient – record if different than primary source.<br><br>Numerical value based on 24 hour clock<br><br>00:00:00 – hour:min:sec<br><br>If no value for seconds data available – do not do not use value 00.
p_paap	dropdown	Advanced Airway Placement	listid: nyn<br><br>0. no<br>1. yes<br>2. not noted	Is the time when EMS personnel first performed Advanced Airway maneuvers reported on source document?<br><br>0=no<br>1=yes<br>2=not noted
p_paap_pri	div	Primary		
p_psraap	dropdown	Source	listid: srcdef<br>1. acr<br>2. cad<br>3. mar<br>4. aacr<br>5. defib	Time when EMS personnel first performed Advanced Airway maneuvers.<br><br>Advanced Airway procedures;<br>Documented in 'Clinical Procedures/Treatments & Results' OR 'Treatment prior to arrival'<br><br>Combitube/LMA/EOA – code 171<br>Nasal ET (Endotracheal Intubation) – code 324<br>CPAP (Continuous Positive Airway Pressure) – code = none

PREDICT - Prehospital Variables

Variable	Type	Caption	List Options	Abstraction Instruction
				RSI (Rapid Sequence Intubation) – code 655 (Succinylcholine/Anectine) or code 645 (Rocuronium) AND text indicating RSI was used<br>Oral ET (Endotracheal Intubation) – code 326<br>Cricothyrotomy – code 322<br>Ventilator – code 142 OR text indicating mechanical ventilation was used<br><br>Source of information;<br>1=acr<br>2=cad<br>3=mar<br>4=aacr<br>5 = defibrillator (printed out summary)
p_ptmaap	textbox	Time		Time when EMS personnel first performed Advanced Airway maneuvers.<br><br>Numerical value based on 24 hour clock<br><br>00:00:00 – hour:min:sec<br><br>If no value for seconds data available – do not use value 00.
p_paap_fib	div	Defib		
p_dsraap	dropdown	Source	listid: aedno<br>1. ECG 1<br>2. ECG 2<br>3. ECG 3	What Defib/AAP provided the time used?<br><br>1=1st AAP/Defib Applied<br>2=2nd or greater<br><br>ACR/AACR Possible sources;<br>Incident History<br>Treatment Prior to Arrival<br>Physical Exam – General Appearance<br>Clinical Treatment / Procedures & Results – 301 (Cardiac Monitor)<br>Clinical Treatment / Procedures & Results – 306 (Defibrillation Manual)<br>Clinical Treatment / Procedures & Results – 307 (Defibrillation Semi-Auto)<br>Clinical Treatment / Procedures & Results – 308 (Automatic)
p_synaap	dropdown	Synched?	listid: aedsyn<br><br>-9999. no<br>1. yes	Does the Defib/AAP appear synchronized with the Atomic Clock?<br><br>null=no<br>1=yes

Variable	Type	Caption	List Options	Abstraction Instruction
				For example; If defib times appear 1 hour ahead or behind during daylight savings time it can be assumed that they have not yet been re-synchronized = NO.<br><br>Another example (From MOO): a defibrillator provides a time that is 5 minutes different than a Watch time documented for the same ordered event; you know the EMS agency synchronizes monthly; you understand that wristwatch and clock times are often subject to drift and sloppy setting; therefore you conclude that the defibrillator DOES appear synchronized and you DO mark the corresponding box for ''Defib Appears Synched....''.
p_paap_alt	div	Alternate		
p_asraap	dropdown	Source	listid: srcdef<br><br>1. acr<br>2. cad<br>3. mar<br>4. aacr<br>5. defib	Alternative source for '1st AAP Defib Turned On' time – record if different than primary source.<br><br>1=acr<br>2=cad<br>3=mar<br>4=aacr<br>5 = defibrillator (printed out summary)
p_atmaap	textbox	Time		Alternative value for '1st AAP Defib Turned On' time – record if different than primary source.<br><br>Numerical value based on 24 hour clock<br><br>00:00:00 – hour:min:sec<br><br>If no value for seconds data available – do not do not use value 00.
p_paap_det	div	Doc/Est Time		
p_ndtaap	dropdown	Doc/Est Time	listid: notime<br><br>-9999. doc time<br>1. est time	Documented/Estimated time data available for '1st AAP Defib Turned On'.<br><br>Doc time - Documented time exists for ''1st AAP Defib Turned On''<br>1=est time - Estimated time exists for '1st AAP Defib Turned On'
p_etmaap	textbox	Est Time		Alternative value for '1st AAP Defib Turned On' time – record if different than primary source.<br><br>Numerical value based on 24 hour clock<br><br>00:00:00 – hour:min:sec

Variable	Type	Caption	List Options	Abstraction Instruction
				If no value for seconds data available – do not do not use value 00.
p_pdps	dropdown	Depart Patient Site	listid: nyn<br>0. no<br>1. yes<br>2. not noted	Is 'Depart Patient Site' time reported on source document?<br>0=no<br>1=yes<br>2=not noted
p_pdps_pri	div	Primary		
p_psrdps	dropdown	Source	listid: srcdef<br>1. acr<br>2. cad<br>3. mar<br>4. aacr<br>5. defib	Source for time 'Depart Patient Site'.<br>1 = acr<br>2 = cad<br>3 = MAR<br>4 = aacr
p_ptmdps	textbox	Time		CAD. If not available obtain from ACR "Call Events".<br>Time when vehicle transported from scene.<br>In the case of multiple vehicles, use the ACR that was completed by the EMS crew that treated and transported the patient.
p_pdps_det	div	Doc/Est Time		
p_etmdps	textbox	Est Time		*Alternative value for 'Depart Patient Site' time – record if different than primary source.*<br>*Numerical value based on 24 hour clock*<br>*00:00:00 – hour:min:sec*

Variable	Type	Caption	List Options	Abstraction Instruction
				If no value for seconds data available – do not do not use value 00.
p_plft	dropdown	Patient Transported from Scene	listid: nyn<br>0. no<br>1. yes<br>2. not noted	Is 'Patient Transported from Scene' time reported on source document?<br>0=no<br>1=yes<br>2=not noted
p_plft_pri	div	Primary		
p_psrlft	dropdown	Source	listid: src<br>1. acr<br>2. cad<br>3. mar<br>4. aacr	Source for time Patient Transported from Scene.<br>1 = acr<br>2 = cad<br>3 = MAR<br>4 = aacr
p_ptmlft	textbox	Time		CAD. If not available obtain from ACR "Call Events" - Time patient is transported away from scene of initial call by EMS personnel: vehicle starts moving.<br>Possible ACR/AACR locations;<br>Treatment/Procedures & Results; Code 231 (Transported Supine), 232 (Transported Semi-prone), 233 (Transported prone), 234 (Transported semi-sitting), 235 (Transported sitting), 236 (Patient Ambulatory), 237 Call Events; Departed Scene (LAND) or Depart Landing Site (AIR).<br>In the case of multiple vehicles, use the ACR that was completed by the EMS crew that treated and transported the patient.
p_plft_alt	div	Alternate		
p_asrlft	dropdown	Source	listid: src<br>1. acr<br>2. cad<br>3. mar<br>4. aacr	Alternative source for 'Patient Transported from Scene' time – record if different than primary source.<br>1=acr<br>2=cad<br>3=mar<br>4=aacr
p_atmlft	textbox	Time		Alternative time of 'Patient Transported From Scene'.<br>Numerical value based on 24 hour clock

Variable	Type	Caption	List Options	Abstraction Instruction
				00:00:00 – hour:min:sec<br><br>If no value for seconds data available – do not use value 00.
p_plft_det	div	Doc/Est Time		
p_ndtlft	dropdown	Doc/Est Time	listid: notime<br><br>-9999. doc time<br>1. est time	Documented/Estimated time data available for 'Patient Transported from Scene'.<br><br>Doc time - Documented time exists for ''Patient Transported from Scene''<br>1=est time - Estimated time exists for 'Patient Transported from Scene'
p_etmlft	textbox	Est Time		Alternative value for 'Patient Transported from Scene' time – record if different than primary source.<br><br>Numerical value based on 24 hour clock<br><br>00:00:00 – hour:min:sec<br><br>If no value for seconds data available – do not do not use value 00.
p_pdls	dropdown	Depart Landing Site (air only)	listid: nyn<br><br>0. no<br>1. yes<br>2. not noted	Is 'Depart Landing Site' time reported on source document?<br><br>0=no<br>1=yes<br>2=not noted
p_pdls_pri	div	Primary		
p_psrdls	dropdown	Source	listid: srcdef<br>1. acr<br>2. cad<br>3. mar<br>4. aacr<br>5. defib	Source for time 'Depart Landing Site'.<br>1 = acr<br>2 = cad<br>3 = MAR<br>4 = aacr<br>5 = defibrillator (printed out summary)
p_ptmdls	textbox	Time		CAD. If not available obtain from ACR "Call Events".<br><br>Time when vehicle transported from scene.<br><br>In the case of multiple vehicles, use the ACR that was completed by the EMS crew that treated and transported the patient.
p_pdls_alt	div	Alternate		
p_asrdls	dropdown	Source	listid: srcdef<br><br>1. acr<br>2. cad<br>3. mar<br>4. aacr	Alternative source for 'Depart Landing Site' time – record if different than primary source.<br><br>1=acr<br>2=cad<br>3=mar

PREDICT - Prehospital Variables

Variable	Type	Caption	List Options	Abstraction Instruction
			5. defib	4=aacr<br>5 = defibrillator (printed out summary)
p_atmdls	textbox	Time		Alternative time of 'Depart Landing Site'.<br>Numerical value based on 24 hour clock<br>00:00:00 – hour:min:sec<br>If no value for seconds data available – do not use value 00.
p_pdls_det	div	Doc/Est Time		
p_etmdls	textbox	Est Time		Alternative value for 'Depart Landing Site' time – record if different than primary source.<br>Numerical value based on 24 hour clock<br>00:00:00 – hour:min:sec<br>If no value for seconds data available – do not do not use value 00.
p_padl	dropdown	Arrive Destination Land Site (air only)	listid: nyn<br>0. no<br>1. yes<br>2. not noted	Is 'Arrive Destination Land Site' time reported on source document?<br>0=no<br>1=yes<br>2=not noted
p_padl_pri	div	Primary		
p_psradl	dropdown	Source	listid: srcdef<br>1. acr<br>2. cad<br>3. mar<br>4. aacr<br>5. defib	Source for time 'Arrive Destination Land Site'.<br>1 = acr<br>2 = cad<br>3 = MAR<br>4 = aacr<br>5 = defibrillator (printed out summary)
p_ptmadl	textbox	Time		CAD. If not available obtain from ACR "Call Events".<br>Time when vehicle arrived at destination land site.<br>In the case of multiple vehicles, use the ACR that was completed by the EMS crew that treated and transported the patient.
p_padl_alt	div	Alternate		
p_asradl	dropdown	Source	listid: srcdef<br>1. acr<br>2. cad<br>3. mar	Alternative source for 'Arrive Destination Land Site' time – record if different than primary source.<br>1=acr<br>2=cad

Variable	Type	Caption	List Options	Abstraction Instruction
			4. aacr<br>5. defib	3=mar<br>4=aacr<br>5 = defibrillator (printed out summary)
p_atmadl	textbox	Time		Alternative time of 'Arrive Destination Land Site'.<br>Numerical value based on 24 hour clock<br><br>00:00:00 – hour:min:sec<br><br>If no value for seconds data available – do not use value 00.
p_padl_det	div	Doc/Est Time		
p_etmadl	textbox	Est Time		Alternative value for 'Arrive Destination Land Site' time – record if different than primary source.<br><br>Numerical value based on 24 hour clock<br><br>00:00:00 – hour:min:sec<br><br>If no value for seconds data available – do not do not use value 00.
p_pdlp	dropdown	Delivery Patient Site (air only)	listid: nyn<br><br>0. no<br>1. yes<br>2. not noted	Is ''''Delivery Patient Site'''' time reported on source document?<br><br>0=no<br>1=yes<br>2=not noted
p_pdlp_pri	div	Primary		
p_psrdlp	dropdown	Source	listid: srcdef<br><br>1. acr<br>2. cad<br>3. mar<br>4. aacr<br>5. defib	Source for time 'Delivery Patient Site'.<br><br>1 = acr<br>2 = cad<br>3 = MAR<br>4 = aacr<br>5 = defibrillator (printed out summary)
p_ptmdlp	textbox	Time		CAD. If not available obtain from ACR “Call Events”.<br><br>Time when vehicle arrived at delivery patient site.<br>In the case of multiple vehicles, use the ACR that was completed by the EMS crew that treated and transported the patient.
p_pdlp_alt	div	Alternate		
p_asrdlp	dropdown	Source	listid: srcdef<br><br>1. acr<br>2. cad	Alternative source for 'Delivery Patient Site' time – record if different than primary source.<br><br>1=acr

PREDICT - Prehospital Variables

Variable	Type	Caption	List Options	Abstraction Instruction
			3. mar<br>4. aacr<br>5. defib	2=cad<br>3=mar<br>4=aacr<br>5 = defibrillator (printed out summary)
p_atmdlp	textbox	Time		Alternative time of 'Delivery Patient Site'<br><br>Numerical value based on 24 hour clock<br><br>00:00:00 – hour:min:sec<br><br>If no value for seconds data available – do not use value 00.
p_pdlp_det	div	Doc/Est Time		
p_etmdlp	textbox	Est Time		Alternative value for 'Delivery Patient Site' time – record if different than primary source.<br><br>Numerical value based on 24 hour clock<br><br>00:00:00 – hour:min:sec<br><br>If no value for seconds data available – do not do not use value 00.
p_peda	dropdown	ED Arrival	listid: nyn<br><br>0. no<br>1. yes<br>2. not noted	**Is 'ED Arrival' time reported on source document?**<br><br>0=no<br>1=yes<br>2=not noted
p_peda_pri	div	Primary		
p_psreda	dropdown	Source	listid: src<br>1. acr<br>2. cad<br>3. mar<br>4. aacr	Source for time 'ED Arrival'.<br>1 = acr<br>2 = cad<br>3 = MAR<br>4 = aacr
p_ptmeda	textbox	Time		CAD. If not available obtain from ACR "Call Events".<br><br>Time the vehicle transporting the patient arrives at emergency department or hospital destination is the point where the vehicle wheels stop moving.<br><br>Possible ACR/AACR locations;<br>Clinical Treatment/Procedure & Results – Documented Emergency Department arrival Clinical Treatment/Procedure & Results; Call events – Arrive Destination (ACR) OR Delivery patient site (AACR).

PREDICT - Prehospital Variables

Variable	Type	Caption	List Options	Abstraction Instruction
				In the case of multiple vehicles, use the ACR that was completed by the EMS crew that treated and transported the patient.
p_peda_alt	div	Alternate		
p_asreda	dropdown	Source	listid: src<br><br>1. acr<br><br>2. cad<br>3. mar<br>4. aacr	Alternative source for 'ED Arrival' time – record if different than primary source.<br><br>1=acr<br>2=cad<br>3=mar<br>4=aacr
p_atmeda	textbox	Time		Alternative time of 'ED Arrival'.<br>Numerical value based on 24 hour clock<br><br>00:00:00 – hour:min:sec<br><br>If no value for seconds data available – do not use value 00.
p_peda_det	div	Doc/Est Time		
p_ndteda	dropdown	Doc/Est Time	listid: notime<br><br>-9999. doc time<br>1. est time	Documented/Estimated time data available for 'ED Arrival'.<br><br>Doc time - Documented time exists for ''ED Arrival''<br>1=est time - Estimated time exists for 'ED Arrival'
p_etmeda	textbox	Est Time		Alternative value for 'Delivery Patient Site' time – record if different than primary source.<br><br>Numerical value based on 24 hour clock<br><br>00:00:00 – hour:min:sec<br><br>If no value for seconds data available – do not do not use value 00.
**p_pvtl**	**section**	**Prehospital Vitals**		
p_p1vtl	div	First Recorded Vitals		
p_p1vsa	dropdown	Patient in VSA?	listid: nyu<br><br>0. no<br>1. yes<br>2. unknown	Was patient vital signs absent (VSA) when EMS personnel performed first set of Vital signs?<br><br>0=no<br>1=yes<br>2=unknown<br><br>ACR/AACR Possible sources;<br>Incident History

PREDICT - Prehospital Variables

Variable	Type	Caption	List Options	Abstraction Instruction
				Treatment Prior to Arrival<br>Physical Exam – General Appearance<br>General Administration – Remarks<br>Clinical Treatment / Procedures & Results – 020, 030 (patient assessment)<br><br>Clinical Treatment / Procedures & Results – code 010 (vital signs)<br>ECG code = 30 (VF), 31 (Pulseless VT), 32 (PEA), 33 (Asystole)<br><br>Example – documented VSA vital signs<br>HR = 0<br>RR = 0<br>BP = 0<br>ECG code = 30 (VF), 31 (Pulseless VT), 32 (PEA), 33 (Asystole)<br><br>NOTE - If yes, DO NOT indicate ''NA/NR'' or ''Done'' for each of the listed vital sign components.<br><br>NOTE - If no, indicate ''NA/NR'' or ''Done'' for each component. Where ''done'', enter the appropriate values.
p_p1vtl_bp	div	Blood Pressure		
p_p1bpr	dropdown	Done?	listid: ny<br><br>0. no<br>1. yes	Did the patient receive a Blood Pressure (BP) assessment by EMS personnel during the FIRST vital sign assessment?<br><br>0=no<br>1=yes<br><br>ACR/AACR Possible sources;<br>Incident History<br>Treatment Prior to Arrival<br>Physical Exam – General Appearance<br>Clinical Treatment / Procedures & Results – 020, 030 (patient assessment)<br><br>Clinical Treatment / Procedures & Results – code 010 (vital signs)
p_p1bpnd	dropdown	SBP Detectable?	listid: ny<br><br>0. no<br>1. yes	Did the patient have a detectable Systolic Blood Pressure when BP taken by EMS personnel during the FIRST vital sign assessment?<br><br>0=no<br>1=yes

PREDICT - Prehospital Variables

Variable	Type	Caption	List Options	Abstraction Instruction
				NOTE – This DOES NOT apply to cases where no SBP was RECORDED.<br>E.g. systolic blood pressure was indicated in the PCR as being ''not detectable''.<br><br>NOTE - If no SBP is recorded in the pre-hospital record, indicate not available/not recorded (NA/NR). DO NOT enter ZERO.<br><br>ACR/AACR Possible sources;<br>Incident History<br>Treatment Prior to Arrival<br>Physical Exam – General Appearance<br>Clinical Treatment / Procedures & Results – 020, 030 (patient assessment)<br><br>Clinical Treatment / Procedures & Results – code 010 (vital signs)
p_p1sbp	textbox	Systolic BP (mmHg)		ACR – Clinical Treatment/Procedures & Results –Blood Pressure – Systolic.<br><br>Can be obtained form Incident History or Clinical Information - Treatment prior to arrival if references a value from a previous crew or from a previous crew's ACR.<br><br>Document the first recorded systolic blood pressure. Recorded values may have been obtained by either palpation, auscultation, or automated device.<br><br>NOTE - If no SBP is recorded in the pre-hospital record, indicate not available/not recorded (NA/NR). DO NOT enter ZERO.
p_p1dbp	textbox	Diastolic BP (mmHg)		ACR – Clinical Treatment/Procedures & Results – Blood Pressure –Diastolic.<br><br>Can be obtained form Incident History or Clinical Information - Treatment prior to arrival if references a value from a previous crew or from a previous crew's ACR.

Variable	Type	Caption	List Options	Abstraction Instruction
				Document the first recorded diastolic blood pressure corresponding to first systolic blood pressure. Recorded values may have been obtained by either auscultation, or automated device. If first systolic blood pressure is recorded by palpation (indicated by letter "P" in first diastolic blood pressure value) leave blank.<br><br>NOTE - If no DBP is recorded in the pre-hospital record, indicate not available/not recorded (NA/NR). DO NOT enter ZERO.
p_p1vtl_rspr	div	Respiratory Rate		
p_p1rspr	dropdown	Done?	listid: ny<br><br>0. no<br>1. yes	Did the patient receive a Respiratory Rate (RR) assessment by EMS personnel during the FIRST vital sign assessment?<br><br>0=no<br>1=yes<br><br>ACR/AACR Possible sources;<br>Incident History<br>Treatment Prior to Arrival<br>Physical Exam – General Appearance<br>Clinical Treatment / Procedures & Results – 020, 030 (patient assessment)<br><br>Clinical Treatment / Procedures & Results – code 010 (vital signs)
p_p1rsp	textbox	Rate		What was the recorded Respiratory Rate during the FIRST vital sign assessment by EMS personnel?<br><br>Rate – Value between 0 and 100<br><br>ACR/AACR Possible sources;<br>Incident History<br>Treatment Prior to Arrival<br>Physical Exam – General Appearance<br>Clinical Treatment / Procedures & Results – 020, 030 (patient assessment)<br><br>Clinical Treatment / Procedures & Results – code 010 (vital signs)<br><br>NOTE; If respiratory rate is assisted (for example with bag-mask), mark "not available/not recorded.

PREDICT - Prehospital Variables

Variable	Type	Caption	List Options	Abstraction Instruction
p_p1vtl_hr	div	Heart Rate		
p_p1hrr	dropdown	Done?	listid: ny<br><br>0. no<br>1. yes	Did the patient receive a Heart Rate (HR) assessment by EMS personnel during the FIRST vital sign assessment?<br><br>0=no<br>1=yes<br><br>ACR/AACR Possible sources;<br>Incident History<br>Treatment Prior to Arrival<br>Physical Exam – General Appearance<br>Clinical Treatment / Procedures & Results – 020, 030 (patient assessment)<br><br>Clinical Treatment / Procedures & Results – code 010 (vital signs)<br>Clinical Treatment / Procedures & Results – code 301 (cardiac monitor)
p_p1hr	textbox	Rate		What was the recorded Heart Rate during the FIRST vital sign assessment by EMS personnel?<br><br>Rate – Value between 0 and 250<br><br>ACR/AACR Possible sources;<br>Incident History<br>Treatment Prior to Arrival<br>Physical Exam – General Appearance<br>Clinical Treatment / Procedures & Results – 020, 030 (patient assessment)<br><br>Clinical Treatment / Procedures & Results – code 010 (vital signs)<br>Clinical Treatment / Procedures & Results – code 301 (cardiac monitor)<br><br>NOTE - Indicate “not applicable/not recorded” if pulse not recorded in the prehospital ACR.
p_p1vtl_soxy	div	SpO2		
p_p1o2r	dropdown	Done?	listid: ny<br><br>0. no<br>1. yes	Did the patient receive a Pulse Oximetry (SpO_2) assessment by EMS personnel during the FIRST vital sign assessment?<br><br>0=no<br>1=yes<br><br>ACR/AACR Possible sources;<br>Incident History

Variable	Type	Caption	List Options	Abstraction Instruction
				Treatment Prior to Arrival<br>Physical Exam – General Appearance<br>Clinical Treatment / Procedures & Results – 020, 030 (patient assessment)<br><br>Clinical Treatment / Procedures & Results – code 010 (vital signs)<br>Clinical Treatment / Procedures & Results – code 338 (pulse oximeter)
p_p1o2	textbox	Value		ACR – Clinical Treatment/Procedures & Results – O2 Sat.<br><br>Can be obtained form Incident History or Clinical Information - Treatment prior to arrival if references a value from a previous crew or from a previous crew's ACR.<br><br>AACR - Clinical Treatment/Procedures & Results - SaO2<br><br>Indicated by code 338 or indicated by text that SpO2 or oxygen saturation was acquired.<br><br>NOTE - Indicate “not available/not recorded” if SpO2 not recorded in the pre-hospital patient care record.
p_p1soxy	dropdown	Supplemental Oxygen?	listid: ny<br><br>0. no<br>1. yes	ACR – Clinical Treatment/Procedures & Results.<br><br>Can be obtained form Incident History or Clinical Information - Treatment prior to arrival if references a value from a previous crew or from a previous crew's ACR.<br><br>Indicated by code 130, 131, 132, 133 141, 142, 143 (land only) Air code: 149 or indicated by the text oxygen high concentration mask, Oxygen simple face mask, oxygen nasal cannula, oxygen other, oxygen BVM, Oxygen (mechanical) or Oxygen jet ventilation Air text: Hi-Ox mask.<br><br>AACR– Also indicated in Treatment / Procedures / Medication / Results – Ventilation - FiO2 > 0.2.
p_pgt1vs	dropdown	More than One Set of Vitals?	listid: ny<br><br>0. no<br>1. yes	Did EMS personnel perform and record more than one set of vital signs?<br><br>0=no<br>1=yes

PREDICT - Prehospital Variables

Variable	Type	Caption	List Options	Abstraction Instruction
				Supplemental sets of vital signs DO NOT need to have been performed by the MTC (main treating crew) to be counted and included.
p_p2vtl	div	Second Recorded Vitals		
p_p2vsa	dropdown	Patient in VSA?	listid: nyu<br><br>0. no<br>1. yes<br>2. unknown	Was patient vital signs absent (VSA) when EMS personnel performed SECOND set of Vital signs?<br><br>0=no<br>1=yes<br>2=unknown<br><br>ACR/AACR Possible sources;<br>Incident History<br>Treatment Prior to Arrival<br>Physical Exam – General Appearance<br>General Administration – Remarks<br>Clinical Treatment / Procedures & Results – 020, 030 (patient assessment)<br><br>Clinical Treatment / Procedures & Results – code 010 (vital signs)<br>ECG code = 30 (VF), 31 (Pulseless VT), 32 (PEA), 33 (Asystole)<br><br>Example – documented VSA vital signs<br>HR = 0<br>RR = 0<br>BP = 0<br>ECG code = 30 (VF), 31 (Pulseless VT), 32 (PEA), 33 (Asystole)<br><br>NOTE - If yes, DO NOT indicate ''NA/NR'' or ''Done'' for each of the listed vital sign components.<br><br>NOTE - If no, indicate ''NA/NR'' or ''Done'' for each component. Where ''done'', enter the appropriate values.
p_p2vtl_bp	div	Blood Pressure		
p_p2bpr	dropdown	Done?	listid: ny<br><br>0. no<br>1. yes	Did the patient receive a Blood Pressure (BP) assessment by EMS personnel during the SECOND vital sign assessment?<br><br>0=no<br>1=yes<br><br>ACR/AACR Possible sources;<br>Incident History

PREDICT - Prehospital Variables

Variable	Type	Caption	List Options	Abstraction Instruction
				Treatment Prior to Arrival<br>Physical Exam – General Appearance<br>Clinical Treatment / Procedures & Results – 020, 030 (patient assessment)<br><br>Clinical Treatment / Procedures & Results - code 010 (vital signs)
p_p2bpnd	dropdown	SBP Detectable?	listid: ny<br><br>0. no<br>1. yes	Did the patient have a detectable Systolic Blood Pressure when BP taken by EMS personnel during the SECOND vital sign assessment?<br><br>0=no<br>1=yes<br><br>NOTE – This DOES NOT apply to cases where no SBP was RECORDED.<br>E.g. systolic blood pressure was indicated in the PCR as being ''not detectable''.<br><br>NOTE - If no SBP is recorded in the pre-hospital record, indicate not available/not recorded (NA/NR). DO NOT enter ZERO.<br><br>ACR/AACR Possible sources;<br>Incident History<br>Treatment Prior to Arrival<br>Physical Exam – General Appearance<br>Clinical Treatment / Procedures & Results – 020, 030 (patient assessment)<br><br>Clinical Treatment / Procedures & Results – code 010 (vital signs)
p_p2sbp	textbox	Systolic BP (mmHg)		ACR – Clinical Treatment/Procedures & Results – Blood Pressure – Systolic.<br><br>Can be obtained form Incident History or Clinical Information - Treatment prior to arrival if references a value from a previous crew or from a previous crew's ACR.<br><br>Document the second recorded systolic blood pressure. Recorded values may have been obtained by either palpation, auscultation, or automated device.<br><br>NOTE - If no SBP is recorded in the pre-hospital record, indicate not available/not recorded (NA/NR). DO NOT enter ZERO.

Variable	Type	Caption	List Options	Abstraction Instruction
p_p2dbp	textbox	Diastolic BP (mmHg)		ACR – Clinical Treatment/Procedures & Results – Blood Pressure –Diastolic<br><br>Can be obtained form Incident History or Clinical Information - Treatment prior to arrival if references a value from a previous crew or from a previous crew's ACR.<br><br>Document the second recorded diastolic blood pressure corresponding to second systolic blood pressure. Recorded values may have been obtained by either auscultation, or automated device. If second systolic blood pressure is recorded by palpation (indicated by letter “P” in second diastolic blood pressure value) leave blank.
p_p2vtl_rspr	div	Respiratory Rate		
p_p2rspr	dropdown	Done?	listid: ny<br><br>0. no<br>1. yes	Did the patient receive a Respiratory Rate (RR) assessment by EMS personnel during the SECOND vital sign assessment?<br><br>0=no<br>1=yes<br><br>ACR/AACR Possible sources;<br>Incident History<br>Treatment Prior to Arrival<br>Physical Exam – General Appearance<br>Clinical Treatment / Procedures & Results – 020, 030 (patient assessment)<br><br>Clinical Treatment / Procedures & Results – code 010 (vital signs)
p_p2rsp	textbox	Rate		What was the recorded Respiratory Rate during the SECOND vital sign assessment by EMS personnel?<br><br>Rate – Value between 0 and 100<br><br>NOTE; If respiratory rate is assisted (for example with bag-mask), mark “not available/not recorded.<br><br>ACR/AACR Possible sources;<br>Incident History<br>Treatment Prior to Arrival<br>Physical Exam – General Appearance<br>Clinical Treatment / Procedures & Results – 020, 030 (patient assessment)

PREDICT - Prehospital Variables

Variable	Type	Caption	List Options	Abstraction Instruction
				Clinical Treatment / Procedures & Results - code 010 (vital signs)
p_p2vtl_hr	div	Heart Rate		
p_p2hrr	dropdown	Done?	listid: ny<br><br>0. no<br>1. yes	Did the patient receive a Heart Rate (HR) assessment by EMS personnel during the SECOND vital sign assessment?<br><br>0=no<br>1=yes<br><br>ACR/AACR Possible sources;<br>Incident History<br>Treatment Prior to Arrival<br>Physical Exam – General Appearance<br>Clinical Treatment / Procedures & Results – 020, 030 (patient assessment)<br><br>Clinical Treatment / Procedures & Results – code 010 (vital signs)<br>Clinical Treatment / Procedures & Results – code 301 (cardiac monitor)
p_p2hr	textbox	Rate		What was the recorded Heart Rate during the SECOND vital sign assessment by EMS personnel?<br><br>Rate – Value between 0 and 250<br><br>NOTE - Indicate “not applicable/not recorded” if pulse not recorded in the prehospital ACR.<br><br>ACR/AACR Possible sources;<br>Incident History<br>Treatment Prior to Arrival<br>Physical Exam – General Appearance<br>Clinical Treatment / Procedures & Results – 020, 030 (patient assessment)<br><br>Clinical Treatment / Procedures & Results – code 010 (vital signs)<br>Clinical Treatment / Procedures & Results - code 301 (cardiac monitor)
p_p2vtl_soxy	div	SpO2		
p_p2o2r	dropdown	Done?	listid: ny<br><br>0. no<br>1. yes	Did the patient receive a Pulse Oximetry (SpO_2) assessment by EMS personnel during the SECOND vital sign assessment?<br><br>0=no<br>1=yes

Variable	Type	Caption	List Options	Abstraction Instruction
				ACR/AACR Possible sources;<br>Incident History<br>Treatment Prior to Arrival<br>Physical Exam – General Appearance<br>Clinical Treatment / Procedures & Results – 020, 030 (patient assessment)<br>Clinical Treatment / Procedures & Results – code 010 (vital signs)<br>Clinical Treatment / Procedures & Results – code 338 (pulse oximeter)
p_p2o2	textbox	Value		ACR – Clinical Treatment/Procedures & Results – O2 Sat.<br><br>Can be obtained form Incident History or Clinical Information - Treatment prior to arrival if references a value from a previous crew or from a previous crew's ACR.<br><br>AACR - Clinical Treatment/Procedures & Results - SaO2<br><br>Indicated by code 338 or indicated by text that SpO2 or oxygen saturation was acquired.<br><br>NOTE - Indicate "not available/not recorded" if SpO2 not recorded in the pre-hospital patient care record.
p_p2soxy	dropdown	Supplemental Oxygen?	listid: ny<br>0. no<br>1. yes	ACR – Clinical Treatment/Procedures & Results.<br><br>Can be obtained form Incident History or Clinical Information - Treatment prior to arrival if references a value from a previous crew or from a previous crew's ACR.<br><br>Indicated by code 130, 131, 132, 133 141, 142, 143 (land only) Air code: 149 or indicated by the text oxygen high concentration mask, Oxygen simple face mask, oxygen nasal cannula, oxygen other, oxygen BVM, Oxygen (mechanical) or Oxygen jet ventilation Air text: Hi-Ox mask.<br><br>AACR– Also indicated in Treatment / Procedures / Medication / Results – Ventilation - FiO2 > 0.2.
p_pwrvtl	div	Worst Recorded Vitals		
p_pwvsa	dropdown	Patient in VSA?	listid: nyu	Was patient vital signs absent (VSA) when EMS personnel performed WORST set of Vital signs?

Variable	Type	Caption	List Options	Abstraction Instruction
			0. no<br>1. yes<br>2. unknown	0=no<br>1=yes<br>2=unknown<br><br>ACR/AACR Possible sources;<br>Incident History<br>Treatment Prior to Arrival<br>Physical Exam – General Appearance<br>General Administration – Remarks<br>Clinical Treatment / Procedures & Results – 020, 030 (patient assessment)<br><br>Clinical Treatment / Procedures & Results – code 010 (vital signs)<br>ECG code = 30 (VF), 31 (Pulseless VT), 32 (PEA), 33 (Asystole)<br><br>Example – documented VSA vital signs<br>HR = 0<br>RR = 0<br>BP = 0<br>ECG code = 30 (VF), 31 (Pulseless VT), 32 (PEA), 33 (Asystole)<br><br>NOTE - Worst recorded values need not have been obtained at the same time or be considered a set of vital signs.<br><br>NOTE - Where a vital sign was recorded only once in the prehospital care record, enter that value as both the lowest and highest (for respiratory and pulse rates) recorded value.
p_pwrvtl_lsbp	div	Lowest SBP		
p_pwbpr	dropdown	Done?	listid: ny<br><br>0. no<br>1. yes	Is there a recorded value for the LOWEST systolic blood pressure as taken by EMS personnel?<br><br>0=no<br>1=yes<br><br>ACR/AACR Possible sources;<br>Incident History<br>Treatment Prior to Arrival<br>Physical Exam – General Appearance<br>Clinical Treatment / Procedures & Results – 020, 030 (patient assessment)<br><br>Clinical Treatment / Procedures & Results – code 010 (vital signs)

PREDICT - Prehospital Variables

Variable	Type	Caption	List Options	Abstraction Instruction
p_pwbpnd	dropdown	Lowest SBP Detectable?	listid: ny<br><br>0. no<br>1. yes	Did the patient have a detectable Systolic Blood Pressure when BP taken by EMS personnel during the WORST vital sign assessment?<br><br>Did EMS personnel record value for Systolic Blood Pressure for patient?<br><br>0=no<br>1=yes<br><br>ACR/AACR Possible sources;<br>Incident History<br>Treatment Prior to Arrival<br>Physical Exam – General Appearance<br>Clinical Treatment / Procedures & Results – 020, 030 (patient assessment)<br><br>Clinical Treatment / Procedures & Results – code 010 (vital signs)<br><br>NOTE – This DOES NOT apply to cases where no SBP was RECORDED.<br>E.g. systolic blood pressure was indicated in the PCR as being ''not detectable''.<br><br>NOTE - If no SBP is recorded in the pre-hospital record, indicate not available/not recorded (NA/NR). DO NOT enter ZERO.<br><br>NOTE - Worst recorded values need not have been obtained at the same time or be considered a set of vital signs.
p_pwsbp	textbox	Lowest SBP (mmHg)		
p_pwdbp	textbox	DBP @ Lowest SBP (mmHg)		What was the value for the DIASTOLIC Blood pressure at the same time of the lowest SYSTOLIC Blood pressure?<br><br>Value – Number between 0 and 160mmHg<br><br>ACR/AACR Possible sources;<br><br>Incident History<br>Treatment Prior to Arrival<br>Physical Exam – General Appearance

Variable	Type	Caption	List Options	Abstraction Instruction
				Clinical Treatment / Procedures & Results – 020, 030 (patient assessment)<br><br>Clinical Treatment / Procedures & Results – code 010 (vital signs)
p_pwrvtl_hrr	div	Highest Respiratory Rate		
p_pwhrspr	dropdown	Done?	listid: ny<br><br>0. no<br>1. yes	Did the patient receive a Respiratory Rate (RR) assessment by EMS personnel during patient care?<br><br>0=no<br>1=yes<br><br>ACR/AACR Possible sources;<br>Incident History<br>Treatment Prior to Arrival<br>Physical Exam – General Appearance<br>Clinical Treatment / Procedures & Results – 020, 030 (patient assessment)<br><br>Clinical Treatment / Procedures & Results – code 010 (vital signs)
p_pwhrsp	textbox	Highest Rate		What was the HIGHEST recorded Respiratory Rate during assessment by EMS personnel?<br><br>Rate – Value between 0 and 100<br><br>ACR/AACR Possible sources;<br>Incident History<br>Treatment Prior to Arrival<br>Physical Exam – General Appearance<br>Clinical Treatment / Procedures & Results – 020, 030 (patient assessment)<br><br>Clinical Treatment / Procedures & Results – code 010 (vital signs)<br><br>NOTE; If respiratory rate is assisted (for example with bag-mask), mark “not available/not recorded.<br><br>NOTE - Worst recorded values need not have been obtained at the same time or be considered a set of vital signs.<br><br>NOTE - Where a vital sign was recorded only once in the prehospital care record, enter that value as both the lowest and highest (for respiratory and pulse rates) recorded value.
p_pwrvtl_lrr	div	Lowest Respiratory Rate		

Variable	Type	Caption	List Options	Abstraction Instruction
p_pwlrspr	dropdown	Done?	listid: ny<br><br>0. no<br>1. yes	Did the patient receive a Respiratory Rate (RR) assessment by EMS personnel during patient care?<br><br>0=no<br>1=yes<br><br>ACR/AACR Possible sources;<br>Incident History<br>Treatment Prior to Arrival<br>Physical Exam – General Appearance<br>Clinical Treatment / Procedures & Results – 020, 030 (patient assessment)<br><br>Clinical Treatment / Procedures & Results – code 010 (vital signs)
p_pwlrsp	textbox	Lowest Rate		What was the LOWEST recorded Respiratory Rate during assessment by EMS personnel?<br><br>Rate – Value between 0 and 100<br><br>ACR/AACR Possible sources;<br>Incident History<br>Treatment Prior to Arrival<br>Physical Exam – General Appearance<br>Clinical Treatment / Procedures & Results – 020, 030 (patient assessment)<br><br>Clinical Treatment / Procedures & Results – code 010 (vital signs)<br><br>NOTE; If respiratory rate is assisted (for example with bag-mask), mark “not available/not recorded.<br><br>NOTE - Worst recorded values need not have been obtained at the same time or be considered a set of vital signs.<br><br>NOTE - Where a vital sign was recorded only once in the prehospital care record, enter that value as both the lowest and highest (for respiratory and pulse rates) recorded value.
p_pwrvtl_hhr	div	Highest Heart Rate		
p_pwhhrr	dropdown	Done?	listid: ny<br><br>0. no<br>1. yes	Did the patient receive a Heart Rate (HR) assessment by EMS personnel during patient care?<br><br>0=no<br>1=yes

PREDICT - Prehospital Variables

Variable	Type	Caption	List Options	Abstraction Instruction
				ACR/AACR Possible sources;<br>Incident History<br>Treatment Prior to Arrival<br>Physical Exam – General Appearance<br>Clinical Treatment / Procedures & Results – 020, 030 (patient assessment)<br><br>Clinical Treatment / Procedures & Results – code 010 (vital signs)<br>Clinical Treatment / Procedures & Results – code 301 (cardiac monitor)
p_pwhhr	textbox	Rate		What was the HIGHEST recorded Heart Rate during assessment by EMS personnel?<br><br>Rate – Value between 0 and 250<br><br>ACR/AACR Possible sources;<br>Incident History<br>Treatment Prior to Arrival<br>Physical Exam – General Appearance<br>Clinical Treatment / Procedures & Results – 020, 030 (patient assessment)<br><br>Clinical Treatment / Procedures & Results – code 010 (vital signs)<br>Clinical Treatment / Procedures & Results – code 301 (cardiac monitor)<br><br>NOTE - Indicate “not applicable/not recorded” if pulse not recorded in the prehospital ACR<br><br>NOTE - Worst recorded values need not have been obtained at the same time or be considered a set of vital signs.<br><br>NOTE - Where a vital sign was recorded only once in the prehospital care record, enter that value as both the lowest and highest (for respiratory and pulse rates) recorded value.
p_pwrvtl_lhr	div	Lowest Heart Rate		
p_pwlhrr	dropdown	Done?	listid: ny<br><br>0. no<br>1. yes	Did the patient receive a Heart Rate (HR) assessment by EMS personnel during patient care?<br><br>0=no<br>1=yes<br><br>ACR/AACR Possible sources;

Variable	Type	Caption	List Options	Abstraction Instruction
				Incident History<br>Treatment Prior to Arrival<br>Physical Exam – General Appearance<br>Clinical Treatment / Procedures & Results – 020, 030 (patient assessment)<br><br>Clinical Treatment / Procedures & Results – code 010 (vital signs)<br>Clinical Treatment / Procedures & Results – code 301 (cardiac monitor)
p_pwlhr	textbox	Rate		What was the LOWEST recorded Heart Rate during assessment by EMS personnel?<br><br>Rate – Value between 0 and 250<br><br>ACR/AACR Possible sources;<br>Incident History<br>Treatment Prior to Arrival<br>Physical Exam – General Appearance<br>Clinical Treatment / Procedures & Results – 020, 030 (patient assessment)<br><br>Clinical Treatment / Procedures & Results – code 010 (vital signs)<br>Clinical Treatment / Procedures & Results – code 301 (cardiac monitor)<br><br>NOTE - Indicate “not applicable/not recorded” if pulse not recorded in the prehospital ACR.<br><br>NOTE - Worst recorded values need not have been obtained at the same time or be considered a set of vital signs.<br><br>NOTE - Where a vital sign was recorded only once in the prehospital care record, enter that value as both the lowest and highest (for respiratory and pulse rates) recorded value.
**p_ecgr**	**section**	**ECG Recording**		
p_ecg	dropdown	Does ECG Recording Exist for the EMS or Fire Treatment	listid: ny<br><br>0. no<br>1. yes	Did EMS providers possess a defibrillation device to record the ECG?<br><br>0=no<br><br>1=yes

PREDICT - Prehospital Variables

Variable	Type	Caption	List Options	Abstraction Instruction
				Indicate no or yes, whether an electronic ECG recording of the EMS effort (either in part or whole) was downloaded from EMS (includes fire) to the site.<br><br>This is also not intended to include electronic ECG<br><br>recordings from lay or bystander use of AEDs.<br><br>Devices must have Accelerometer or Trans Thoracic Impedance enabled.<br><br>E.g.<br><br>Lifepack 12<br>Zoll E series
p_totecg	dropdown	For the Entire Treatment Period or Only Part	listid: totecg<br><br>0. part of the resuscitation<br>1. entire	Was the ECG recording done for the ENTIRE or only PART of the treatment?<br><br>0=part of the treatment<br>1=entire
p_ecgrec	dropdown	How Many ECG Recordings (total number)	listid: 3plus<br><br>1. one<br>2. two<br>3. three<br>4. more than three	How many recordings of the ECG process (total number) were made in ED?<br><br>We expect there to be one ECG on arrival and we are looking to collect the ECG prior to reperfusion strategy and post reperfusion strategy thus we are looking for three ECGs usually unless there is a very complicated course that requires more than one reperfusion strategy or reperfusion complication such as reischemia or cardiac arrest. If you find the three we are looking for this is all we want to collect.<br><br>1=one<br>2=two<br>3=three<br>4=more than three<br><br>Source = Patient follow up information / ED chart
p_ecgrecnum	textbox	How Many		How many more than 3 recordings of the ECG process (total number) were made in prehospital setting?

Variable	Type	Caption	List Options	Abstraction Instruction
				Value = number 4 to 99
p_ecguse	dropdown	How Many Electronic Recordings	listid: p_3plus<br><br>0. none<br>1. one<br>2. two<br>3. three<br>4. more than three	How many electronic recordings of the ECG process were made in prehospital setting?<br><br>1=one<br>2=two<br>3=three<br>4=more than three
p_numecg	textbox	How Many		How many more than 3 electronic recordings of the ECG process were made in prehospital setting?<br><br>Value = number 4 to 99
p_devuse	dropdown	How Many Devices Used	listid: 3plus<br><br>1. one<br>2. two<br>3. three<br>4. more than three	D & A: How many DIFFERENT ECG recording devices were used?<br><br>1=one<br>2=two<br>3=three<br>4=more than three
p_devnum	textbox	How Many		How many more than 3 ECG recording devices were used?<br><br>Value = number between 4 and 9
p_1ecg	div	ECG 1		
p_ecgtm1	textbox	Time		CAD. If not available obtain from ACR “Call Events”.<br><br>Time when the first ECG was taken and recorded.<br><br>ACR/AACR Possible sources;<br>Clinical Treatment / Procedures & Results – 020, 030 (patient assessment)<br><br>In the case of multiple vehicles, use the ACR that was completed by the EMS crew that treated and transported the patient.
p_ecgveh1	dropdown	Vehicle	listid: vehsc<br><br>1. 1st on scene<br>2. 2nd on scene<br>3. 3rd on scene<br>4. 4th on scene<br>5. 5th or greater	Which vehicle + EMS crew provided the first recorded ECG?<br><br>1=1st vehicle on scene<br>2=2nd vehicle on scene<br>3=3rd vehicle on scene<br>4=4th vehicle on scene<br>5=5th or greater
p_ecgmfg1	dropdown	Manufacture	listid: dev<br><br>1. Medtronics<br>2. Philips<br>3. Zoll	Who was the manufacturer of the defibrillator responsible for the first recorded ECG?<br><br>1=medtronics<br>2=Philips

Variable	Type	Caption	List Options	Abstraction Instruction
			4. other	3=zoll<br>4=other
p_spomfg1	textbox	Specify Other		Specify manufacturer of the "OTHER" device.<br><br>Value = text
p_pecg1	dropdown	Paper ECG Exists	listid: ny<br>0. no<br>1. yes	Is there a paper recording of the first ECG data available on request?<br><br>0=no<br>1=yes<br><br>Only one recording is intended to be present for each of the three listed devices. Sometimes more than one recording exists for the same device; such as when a device was applied, then turned off, and later turned on.
p_recrd1	dropdown	Electronic ECG File Exists	listid: ny<br><br>0. no<br>1. yes	Is there an electronic recording of the first ECG data available on request?<br><br>0=no<br>1=yes<br><br>Only one recording is intended to be uploaded for each of the three listed devices. Where more than one recording exists for the same device (such as when a device was applied, then turned off, and later turned on) can recordings into one zip file and attach it (using the required file naming conventions, as above) for upload.
p_ecgdg1	dropdown	Diagnosis - Computer Assisted	listid: ny<br><br>0. no<br>1. yes	Indicate whether or not there was computer assisted diagnosis for the first ECG.<br><br>0=no<br>1=yes<br><br>ACR/AACR Possible sources;<br>Clinical Treatment / Procedures & Results – 020, 030 (patient assessment)<br><br>In the case of multiple vehicles, use the ACR that was completed by the EMS crew that treated and transported the patient.
p_ecgdgsp1	textbox	Diagnosis - Computer Assisted - Specify 1		Please specify computer assisted diagnosis for the first ECG.<br><br>Value = text

Variable	Type	Caption	List Options	Abstraction Instruction
				ACR/AACR Possible sources;<br>Clinical Treatment / Procedures & Results – 020, 030 (patient assessment)<br><br>In the case of multiple vehicles, use the ACR that was completed by the EMS crew that treated and transported the patient.
p_ecgdgsp1_2	textbox	Diagnosis - Computer Assisted - Specify 2 (cont)		
p_ecgdgsp1_3	textbox	Diagnosis - Computer Assisted - Specify 3 (cont)		
p_ecgrev1	dropdown	Electronic ECG Reviewed	listid: nyu<br><br>0. no<br>1. yes<br>2. unknown	Indicate whether or not the first electronic ECG was reviewed by the site or if the attendant annotations and QA data was generated solely by the device software.<br><br>0=no<br>1=yes<br>2=unknown<br><br>The goal is for sites to review the recordings so as to ''correct'' any oversights by the software.<br><br>E.g. Was the EMS/Defibrillator analysis of the ECG accurate based upon data guardian's analysis of the data?
p_ecgd1	dropdown	ECG Data Exists	listid: ny<br><br>0. no<br>1. yes	Is there the first ECG data available on request?<br><br>0=no<br>1=yes<br><br>Only one recording is intended to be present for each of the three listed devices. Sometimes more than one recording exists for the same device; such as when a device was applied, then turned off, and later turned on.
p_ecg3lead1	dropdown	3 Lead ECG	listid: ny<br><br>0. no<br>1. yes	Was the first ECG recording obtained on the patient a 3 lead ECG recording?<br><br>0=no<br>1=yes<br><br>Only one recording is intended to be present for each of the three listed devices. Sometimes more than one recording exists for the same device; such as when a device was applied, then turned off, and later turned on.

Variable	Type	Caption	List Options	Abstraction Instruction
p_ecg12lead1	dropdown	12 Lead ECG	listid: ny<br><br>0. no<br>1. yes	Was the first ECG recording obtained on the patient a 12 lead ECG recording?<br><br>0=no<br>1=yes<br><br>Only one recording is intended to be present for each of the three listed devices. Sometimes more than one recording exists for the same device; such as when a device was applied, then turned off, and later turned on.
p_2ecg	div	ECG 2		
p_ecgtm2	textbox	Time		CAD. If not available obtain from ACR "Call Events".<br><br>Time when the second ECG was taken and recorded.<br><br>ACR/AACR Possible sources;<br>Clinical Treatment / Procedures & Results – 020, 030 (patient assessment)<br><br>In the case of multiple vehicles, use the ACR that was completed by the EMS crew that treated and transported the patient.
p_ecgveh2	dropdown	Vehicle	listid: vehsc<br><br>1. 1st on scene<br>2. 2nd on scene<br>3. 3rd on scene<br>4. 4th on scene<br>5. 5th or greater	Which vehicle + EMS crew provided the second recorded ECG?<br><br>1=1st vehicle on scene<br>2=2nd vehicle on scene<br>3=3rd vehicle on scene<br>4=4th vehicle on scene<br>5=5th or greater
p_ecgmfg2	dropdown	Manufacture	listid: dev<br><br>1. Medtronics<br>2. Philips<br>3. Zoll<br>4. other	Who was the manufacturer of the defibrillator responsible for the second recorded ECG?<br><br>1=medtronics<br>2=Philips<br>3=zoll<br>4=other
p_spomfg2	textbox	Specify Other		Specify manufacturer of the "OTHER" device.<br><br>Value = text
p_pecg2	dropdown	Paper ECG Exists	listid: ny<br><br>0. no<br>1. yes	Is there a paper recording of the second ECG data available on request?<br><br>0=no<br>1=yes

Variable	Type	Caption	List Options	Abstraction Instruction
				Only one recording is intended to be present for each of the three listed devices. Sometimes more than one recording exists for the same device; such as when a device was applied, then turned off, and later turned on.
p_recrd2	dropdown	Electronic ECG File Exists	listid: ny<br><br>0. no<br>1. yes	Is there an electronic recording of the second ECG data available on request?<br><br>0=no<br>1=yes<br><br>Only one recording is intended to be uploaded for each of the three listed devices. Where more than one recording exists for the same device (such as when a device was applied, then turned off, and later turned on) can recordings into one zip file and attach it (using the required file naming conventions, as above) for upload.
p_ecgdg2	dropdown	Diagnosis - Computer Assisted	listid: ny<br><br>0. no<br>1. yes	Indicate whether or not there was computer assisted diagnosis for the second ECG.<br><br>0=no<br>1=yes<br><br>ACR/AACR Possible sources;<br>Clinical Treatment / Procedures & Results – 020, 030 (patient assessment)<br><br>In the case of multiple vehicles, use the ACR that was completed by the EMS crew that treated and transported the patient.
p_ecgdgsp2	textbox	Diagnosis - Computer Assisted - Specify 1		Please specify computer assisted diagnosis for the second ECG.<br><br>Value = text<br><br>ACR/AACR Possible sources;<br>Clinical Treatment / Procedures & Results – 020, 030 (patient assessment)<br><br>In the case of multiple vehicles, use the ACR that was completed by the EMS crew that treated and transported the patient.
p_ecgdgsp2_2	textbox	Diagnosis - Computer Assisted - Specify 2 (cont)		

Variable	Type	Caption	List Options	Abstraction Instruction
p_ecgdgsp2_3	textbox	Diagnosis - Computer Assisted - Specify 3 (cont)		
p_ecgrev2	dropdown	Electronic ECG Reviewed	listid: nyu<br>0. no<br>1. yes<br>2. unknown	Indicate whether or not the second electronic ECG was reviewed by the site or if the attendant annotations and QA data was generated solely by the device software.<br>0=no<br>1=yes<br>2=unknown<br>The goal is for sites to review the recordings so as to ''correct'' any oversights by the software.<br>E.g. Was the EMS/Defibrillator analysis of the ECG accurate based upon data guardian's analysis of the data?
p_ecgd2	dropdown	ECG Data Exists	listid: ny<br>0. no<br>1. yes	Is there the second ECG data available on request?<br>0=no<br>1=yes<br>Only one recording is intended to be present for each of the three listed devices. Sometimes more than one recording exists for the same device; such as when a device was applied, then turned off, and later turned on.
p_ecg3lead2	dropdown	3 Lead ECG	listid: ny<br>0. no<br>1. yes	Was the second ECG recording obtained on the patient a 3 lead ECG recording?<br>0=no<br>1=yes<br>Only one recording is intended to be present for each of the three listed devices. Sometimes more than one recording exists for the same device; such as when a device was applied, then turned off, and later turned on.
p_ecg12lead2	dropdown	12 Lead ECG	listid: ny<br>0. no<br>1. yes	Was the second ECG recording obtained on the patient a 12 lead ECG recording?<br>0=no<br>1=yes

Variable	Type	Caption	List Options	Abstraction Instruction
				Only one recording is intended to be present for each of the three listed devices. Sometimes more than one recording exists for the same device; such as when a device was applied, then turned off, and later turned on.
p_3ecg	div	ECG 3		
p_ecgtm3	textbox	Time		CAD. If not available obtain from ACR "Call Events".<br><br>Time when the third ECG was taken and recorded.<br><br>ACR/AACR Possible sources;<br>Clinical Treatment / Procedures & Results – 020, 030 (patient assessment)<br><br>In the case of multiple vehicles, use the ACR that was completed by the EMS crew that treated and transported the patient.
p_ecgveh3	dropdown	Vehicle	listid: vehsc<br>1. 1st on scene<br>2. 2nd on scene<br>3. 3rd on scene<br>4. 4th on scene<br>5. 5th or greater	Which vehicle + EMS crew provided the third recorded ECG?<br><br>1=1st vehicle on scene<br>2=2nd vehicle on scene<br>3=3rd vehicle on scene<br>4=4th vehicle on scene<br>5=5th or greater
p_ecgmfg3	dropdown	Manufacture	listid: dev<br><br>1. Medtronics<br>2. Philips<br>3. Zoll<br>4. other	Who was the manufacturer of the defibrillator responsible for the third recorded ECG?<br><br>1=medtronics<br>2=Philips<br>3=zoll<br>4=other
p_spomfg3	textbox	Specify Other		Specify manufacturer of the "OTHER" device.<br><br>Value = text
p_pecg3	dropdown	Paper ECG Exists	listid: ny<br>0. no<br>1. yes	Is there a paper recording of the third ECG data available on request?<br><br>0=no<br>1=yes<br><br>Only one recording is intended to be present for each of the three listed devices. Sometimes more than one recording exists for the same device; such as when a device was applied, then turned off, and later turned on.

PREDICT - Prehospital Variables

Variable	Type	Caption	List Options	Abstraction Instruction
p_recrd3	dropdown	Electronic ECG File Exists	listid: ny<br><br>0. no<br>1. yes	Is there an electronic recording of the third ECG data available on request?<br><br>0=no<br>1=yes<br><br>Only one recording is intended to be uploaded for each of the three listed devices. Where more than one recording exists for the same device (such as when a device was applied, then turned off, and later turned on) can recordings into one zip file and attach it (using the required file naming conventions, as above) for upload.
p_ecgdg3	dropdown	Diagnosis - Computer Assisted	listid: ny<br><br>0. no<br>1. yes	Indicate whether or not there was computer assisted diagnosis for the third ECG.<br><br>0=no<br>1=yes<br><br>ACR/AACR Possible sources;<br>Clinical Treatment / Procedures & Results – 020, 030 (patient assessment)<br><br>In the case of multiple vehicles, use the ACR that was completed by the EMS crew that treated and transported the patient.
p_ecgdgsp3	textbox	Diagnosis - Computer Assisted - Specify 1		Please specify computer assisted diagnosis for the third ECG.<br><br>Value = text<br><br>ACR/AACR Possible sources;<br>Clinical Treatment / Procedures & Results – 020, 030 (patient assessment)<br><br>In the case of multiple vehicles, use the ACR that was completed by the EMS crew that treated and transported the patient.
p_ecgdgsp3_2	textbox	Diagnosis - Computer Assisted - Specify 2 (cont)		
p_ecgdgsp3_3	textbox	Diagnosis - Computer Assisted - Specify 3 (cont)		

Variable	Type	Caption	List Options	Abstraction Instruction
p_ecgrev3	dropdown	Electronic ECG Reviewed	listid: nyu<br><br>0. no<br>1. yes<br>2. unknown	Indicate whether or not the third electronic ECG was reviewed by the site or if the attendant annotations and QA data was generated solely by the device software.<br><br>0=no<br>1=yes<br>2=unknown<br><br>The goal is for sites to review the recordings so as to ''correct'' any oversights by the software.<br><br>E.g. Was the EMS/Defibrillator analysis of the ECG accurate based upon data guardian's analysis of the data?
p_ecgd3	dropdown	ECG Data Exists	listid: ny<br><br>0. no<br>1. yes	Is there third ECG data available on request?<br><br>0=no<br>1=yes<br><br>Only one recording is intended to be present for each of the three listed devices. Sometimes more than one recording exists for the same device; such as when a device was applied, then turned off, and later turned on.
p_ecg3lead3	dropdown	3 Lead ECG	listid: ny<br><br>0. no<br>1. yes	Was the third ECG recording obtained on the patient a 3 lead ECG recording?<br><br>0=no<br>1=yes<br><br>Only one recording is intended to be present for each of the three listed devices. Sometimes more than one recording exists for the same device; such as when a device was applied, then turned off, and later turned on.
p_ecg12lead3	dropdown	12 Lead ECG	listid: ny<br><br>0. no<br>1. yes	Was the third ECG recording obtained on the patient a 12 lead ECG recording?<br><br>0=no<br>1=yes<br><br>Only one recording is intended to be present for each of the three listed devices. Sometimes more than one recording exists for the same device; such as when a device was applied, then turned off, and later turned on.
**p_pis**	**section**	**Prehospital Interventions**		

PREDICT - Prehospital Variables

Variable	Type	Caption	List Options	Abstraction Instruction
p_pnoint	dropdown	Prehospital Interventions	listid: ny<br><br>0. no<br>1. yes	Select “Yes” if the EMS or Fire Responders applied any of the following interventions.<br><br>ACR:<br>ACR - Clinical Treatment / Procedures & Results<br><br>If no mention go to<br><br>ACR - Clinical Information - Incident History<br><br>If no mention go to<br><br>ACR - General Administration - Remarks / Orders<br><br>if no mention go to<br><br>ACR - Clinical Information - Treatment prior to arrival<br><br>AACR:<br>AACR - Clinical Treatment / Procedures & Results<br><br>if no mention go to<br><br>AACR - Clinical Information – Incident history / Treatment prior to arrival<br><br>if no mention go to<br><br>AACR - Clinical Treatment / Procedures & Results – Physician's Orders/Remarks
p_pfluid2	dropdown	IV/IO Line	listid: p_ndnr<br><br>0. no<br>1. yes<br>2. not recorded	Was an IV or IO line established in the prehospital setting**?**<br>ACR:<br>ACR - Clinical Treatment / Procedures & Results<br><br>If no mention go to<br><br>ACR - Clinical Information - Incident History<br><br>If no mention go to<br><br>ACR - General Administration - Remarks / Orders<br><br>if no mention go to<br><br>ACR - Clinical Information - Treatment prior to arrival

Variable	Type	Caption	List Options	Abstraction Instruction
				AACR:<br>AACR - Clinical Treatment / Procedures & Results<br><br>If no mention go to<br><br>AACR - Clinical Information – Incident history / Treatment prior to arrival<br><br>if no mention go to<br><br>AACR - Clinical Treatment / Procedures & Results – Physician's Orders/Remarks<br><br>Indicated by Code 340, 358, 342, 343, 344, 345, 346, 347, 348, 351, 358 or text indicating initiation of an IV or IO line.
p_pio	dropdown	IO Intravascular Line Attempted	listid: p_ndnr<br><br>0. no<br>1. yes<br>2. not recorded	Was an Intraosseous line attempted but not established in the prehospital setting?<br><br>ACR:<br>ACR - Clinical Treatment / Procedures & Results<br><br>If no mention go to<br><br>ACR - Clinical Information - Incident History<br><br>If no mention go to<br><br>ACR - General Administration - Remarks / Orders<br><br>If no mention go to<br><br>ACR - Clinical Information - Treatment prior to arrival<br><br>AACR:<br>AACR - Clinical Treatment / Procedures & Results<br><br>If no mention go to<br><br>AACR - Clinical Information – Incident history / Treatment prior to arrival<br><br>if no mention go to<br><br>AACR - Clinical Treatment / Procedures & Results – Physician's Orders/Remarks

Variable	Type	Caption	List Options	Abstraction Instruction
				Indicated by Code 358 or text indicating an intra-osseous (IO) line.
p_piv	dropdown	Initiation and/or continuation of an IV attempt	listid: p_ndnr<br><br>0. no<br>1. yes<br>2. not recorded	Was an intravenous line attempted but not extablished in the prehospital setting?<br><br>ACR:<br>ACR - Clinical Treatment / Procedures & Results<br><br>If no mention go to<br><br>ACR - Clinical Information - Incident History<br><br>If no mention go to<br><br>ACR - General Administration - Remarks / Orders<br><br>If no mention go to<br><br>ACR - Clinical Information - Treatment prior to arrival<br><br>AACR:<br>AACR - Clinical Treatment / Procedures & Results<br><br>If no mention go to<br><br>AACR - Clinical Information – Incident history / Treatment prior to arrival<br><br>If no mention go to<br><br>AACR - Clinical Treatment / Procedures & Results – Physician's Orders/Remarks<br><br>Indicated by Code 340, 342, 343, 344, 345, 346, 347, 348, 349, 351, 601.<br>Air codes: land codes not used are 343, 344, 345, 346, 347, 349 & 351. Additional air codes are 315, 317, 341 & 348 or text indicating an intraveneous (IV) line, or saline lock was placed or fluid bolus was given (meaning IV initiation had to have occurred).
p_pfldlv	dropdown	Fluid Delivered	listid: tko<br><br>0. no<br>1. yes<br>2. TKO (to keep open)<br><br>3. not recorded	Was any intravenous fluid delivered to the patient in the prehospital setting?<br><br>ACR:<br>ACR – General Administration – Intake

Variable	Type	Caption	List Options	Abstraction Instruction
				ACR - Clinical Treatment / Procedures & Results<br><br>If no mention go to<br><br>ACR - Clinical Information - Incident History<br><br>If no mention go to<br><br>ACR - General Administration - Remarks / Orders<br><br>If no mention go to<br><br>ACR - Clinical Information - Treatment prior to arrival<br><br>AACR:<br>AACR - Clinical Information and/or Clinical Treatment / Procedures & Results –Fluid – Intake<br><br>If no mention go to<br><br>AACR - Clinical Treatment / Procedures & Results<br><br>if no mention go to<br><br>AACR - Clinical Information – Incident history / Treatment prior to arrival<br><br>If no mention go to<br><br>AACR - Clinical Treatment / Procedures & Results – Physician's Orders/Remarks<br><br>Indicated by Code 340, 342, 343, 344, 345, 346, 347, 348, 349, 351. Air codes: land codes not used are 343, 344, 345, 346, 347, 349 & 351. Additional air codes are 315, 317, 341 & 348 or text indicating an intraveneous (IV) line, or saline lock was placed or fluid bolus was given.

Variable	Type	Caption	List Options	Abstraction Instruction
				Fluid will be considered given when code 351 Air codes: land code 351 not used. Air codes used are 315 & 317 or as found in "Intake" as described above under 'AACR ' is used, (regardless if followed by text indicating amount given) or where text indicates that fluid given by the report of xx ml / cc infused / bolus / administered.<br><br>OR<br><br>Defined by then lack of a successful IV or IO line.<br><br>Indicated by Code 350 or text indicating an unsuccessful intraveneous (IV) line or saline lock or IO.
p_pd5w	dropdown	D5W Fluid Used	listid: p_ndnr<br><br>0. no<br>1. yes<br>2. not recorded	Was the D5W fluid delivered to the patient in the prehospital setting?<br><br>ACR:<br>ACR – General Administration – Intake<br><br>ACR - Clinical Treatment / Procedures & Results<br><br>If no mention go to<br><br>ACR - Clinical Information - Incident History<br><br>If no mention go to<br><br>ACR - General Administration - Remarks / Orders<br><br>if no mention go to<br><br>ACR - Clinical Information - Treatment prior to arrival<br><br>AACR:<br>AACR - Clinical Information and/or Clinical Treatment / Procedures & Results –Fluid – Intake<br><br>If no mention go to<br><br>AACR - Clinical Treatment / Procedures & Results<br><br>If no mention go to

Variable	Type	Caption	List Options	Abstraction Instruction
				AACR - Clinical Information – Incident history / Treatment prior to arrival<br><br>If no mention go to<br><br>AACR - Clinical Treatment / Procedures & Results – Physician's Orders/Remarks<br><br>Indicated by Code 346 Air code: land code 346 not used or text indicating D5W was administered.
p_pns	dropdown	Normal Saline Used	listid: p_ndnr<br><br>0. no<br>1. yes<br>2. not recorded	Was the normal saline delivered to the patient in the prehospital setting?<br><br>ACR:<br>ACR – General Administration – Intake<br><br>ACR - Clinical Treatment / Procedures & Results<br><br>If no mention go to<br><br>ACR - Clinical Information - Incident History<br><br>If no mention go to<br><br>ACR - General Administration - Remarks / Orders<br><br>If no mention go to<br><br>ACR - Clinical Information - Treatment prior to arrival<br><br>AACR:<br>AACR - Clinical Information and/or Clinical Treatment / Procedures & Results –Fluid – Intake<br><br>If no mention go to<br><br>AACR - Clinical Treatment / Procedures & Results<br><br>If no mention go to<br><br>AACR - Clinical Information – Incident history / Treatment prior to arrival<br><br>If no mention go to<br><br>AACR - Clinical Treatment / Procedures & Results – Physician's Orders/Remarks

Variable	Type	Caption	List Options	Abstraction Instruction
				Indicated by Code 345 Air code: land code 345 not used. Air code used is 317 or text indicating normal saline (NS) was administered.
p_plr	dropdown	Lactated Ringers Used	listid: p_ndnr<br>0. no<br>1. yes<br>2. not recorded	Were lactated ringers delivered to the patient in the prehospital setting?<br>ACR:<br>ACR – General Administration – Intake<br>ACR - Clinical Treatment / Procedures & Results<br>If no mention go to<br>ACR - Clinical Information - Incident History<br>If no mention go to<br>ACR - General Administration - Remarks / Orders<br>If no mention go to<br>ACR - Clinical Information - Treatment prior to arrival<br>AACR:<br>AACR - Clinical Information and/or Clinical Treatment / Procedures & Results –Fluid – Intake<br>If no mention go to<br>AACR - Clinical Treatment / Procedures & Results<br>If no mention go to<br>AACR - Clinical Information – Incident history / Treatment prior to arrival<br>If no mention go to<br>AACR - Clinical Treatment / Procedures & Results – Physician's Orders/Remarks<br>Indicated by Code 344 Air code: land code 344 not used. Air code used is 315 or text indicating Lactated Ringers (Ringers Lactate) was administered.
p_pothfl	dropdown	Other Fluid Used	listid: p_ndnr	Any fluid other than lactated ringers, normal saline or D5W infused during the prehospital setting?

Variable	Type	Caption	List Options	Abstraction Instruction
			0. no<br>1. yes<br>2. not recorded	ACR:<br>ACR – General Administration – Intake<br><br>ACR - Clinical Treatment / Procedures & Results<br><br>If no mention go to<br><br>ACR - Clinical Information - Incident History<br><br>If no mention go to<br><br>ACR - General Administration - Remarks / Orders<br><br>If no mention go to<br><br>ACR - Clinical Information - Treatment prior to arrival<br><br>AACR:<br>AACR - Clinical Information and/or Clinical Treatment / Procedures & Results –Fluid – Intake<br><br>If no mention go to<br><br>AACR - Clinical Treatment / Procedures & Results<br><br>if no mention go to<br><br>AACR - Clinical Information – Incident history / Treatment prior to arrival<br><br>If no mention go to<br><br>AACR - Clinical Treatment / Procedures & Results – Physician's Orders/Remarks<br><br>Indicated by Code 347, 348, 349 Air codes: land codes not used are 347 & 349. Additional air code is 360 or text indicating another type of fluid was administered.
p_pothflsp	textbox	Specify Other Fluid Used		Please specify any fluid other than lactated ringers, normal saline or D5W infused during the prehospital setting<br><br>ACR:<br>ACR – General Administration – Intake<br><br>ACR - Clinical Treatment / Procedures & Results

PREDICT - Prehospital Variables

Variable	Type	Caption	List Options	Abstraction Instruction
				If no mention go to<br><br>ACR - Clinical Information - Incident History<br><br>If no mention go to<br><br>ACR - General Administration - Remarks / Orders<br><br>If no mention go to<br><br>ACR - Clinical Information - Treatment prior to arrival<br><br>AACR:<br>AACR - Clinical Information and/or Clinical Treatment / Procedures & Results –Fluid – Intake<br><br>If no mention go to<br><br>AACR - Clinical Treatment / Procedures & Results<br><br>if no mention go to<br><br>AACR - Clinical Information – Incident history / Treatment prior to arrival<br><br>If no mention go to<br><br>AACR - Clinical Treatment / Procedures & Results – Physician's Orders/Remarks<br><br>Indicated by Code 347, 348, 349 Air codes: land codes not used are 347 & 349. Additional air code is 360 or text indicating another type of fluid was administered.
p_pecg12latt	dropdown	12 Lead attempted but not completed	listid: p_ndnr<br><br>0. no<br>1. yes<br>2. not recorded	Was a 12 lead ECG attempted but unobtainable in the prehospital settting?<br><br>ACR:<br>ACR - Clinical Treatment / Procedures & Results<br><br>If no mention go to<br><br>ACR - Clinical Information - Incident History<br><br>If no mention go to

PREDICT - Prehospital Variables

Variable	Type	Caption	List Options	Abstraction Instruction
				ACR - General Administration - Remarks / Orders<br><br>If no mention go to<br><br>ACR - Clinical Information - Treatment prior to arrival<br><br>AACR:<br>AACR - Clinical Treatment / Procedures & Results<br><br>If no mention go to<br><br>AACR - Clinical Information – Incident history / Treatment prior to arrival<br><br>If no mention go to<br><br>AACR - Clinical Treatment / Procedures & Results – Physician's Orders/Remarks<br><br>Indicated by Code 313 or text indicating that a 12 Lead was performed on the patient.
p_ppace	dropdown	Pacing Attempted	listid: nyyn<br><br>0. No<br><br>1. Yes - Successful<br><br>2. Yes - Unsuccessful<br><br>3. Not Recorded	Was transcutaneous pacing in the prehospital setting attempted and was pacing successful?<br><br>Success is defined as mechanical capture (i.e. with every paced beat there is a palpable pulse).<br><br>ACR:<br><br>ACR - Clinical Treatment / Procedures & Results<br><br>If no mention go to<br><br>ACR - Clinical Information - Incident History<br><br>If no mention go to<br><br>ACR - General Administration - Remarks / Orders<br><br>If no mention go to<br><br>ACR - Clinical Information - Treatment prior to arrival<br><br>AACR:<br>AACR - Clinical Treatment / Procedures & Results

Variable	Type	Caption	List Options	Abstraction Instruction
				If no mention go to<br><br>AACR - Clinical Information – Incident history / Treatment prior to arrival<br><br>If no mention go to<br><br>AACR - Clinical Treatment / Procedures & Results – Physician's Orders/Remarks<br><br>Indicated by Code 309 or 310 or text indicating that pacing was used on the patient.
p_pcpr2	dropdown	CPR	listid: p_ndnr<br><br>0. no<br>1. yes<br>2. not recorded	At any time during the prehospital setting and transport was CPR provided by any organized EMS responder?<br><br>ACR:<br>ACR - Clinical Treatment / Procedures & Results<br><br>If no mention go to<br><br>ACR - Clinical Information - Incident History<br><br>If no mention go to<br><br>ACR - General Administration - Remarks / Orders<br><br>If no mention go to<br><br>ACR - Clinical Information - Treatment prior to arrival<br><br>If no mention go to<br><br>ACR - Clinical Information – Cardiac Arrest Information<br><br>AACR:<br>AACR - Clinical Treatment / Procedures & Results<br><br>If no mention go to<br><br>AACR - Clinical Information – Incident history / Treatment prior to arrival<br><br>If no mention go to<br><br>AACR - Clinical Treatment / Procedures & Results – Physician's Orders/Remarks

PREDICT - Prehospital Variables

Variable	Type	Caption	List Options	Abstraction Instruction
				Indicated by Code 200 or text indicating CPR was performed.
p_dtn	div	Drug Therapy Noted		
p_pasa2	dropdown	ASA (Aspirin) Given	listid: nynr<br><br>0. no<br>1. yes<br>2. not recorded	Indicate if ASA was administered at any time during the prehospital course of care.<br><br>0=no<br>1=yes<br>2=not recorded<br><br>ACR - Clinical Treatment / Procedures & Results.<br><br>ASA (Aspirin) Code 504<br><br>NOTE —<br>Where only partial EMS documentation is available to the coordinator (as where the ALS or BLS record is missing), mark ''yes'' for those drug therapies for which documentation is available.<br><br>Where only partial EMS documentation is available to the coordinator (as where the ALS or BLS record is missing), leave ''not recorded'' and ''yes'' blank for a prehospital drug therapy for which the skill-related documentation is missingâ€"then override the error message and indicate which documentation is missing. In most cases this response option will be used when the ALS record is missing.<br><br>DO NOT list any drug therapy provided prior to arrival of any EMS personnel in the organized response.
p_asaftm	textbox	First Given Time		The earliest time noted when administration of ASA is confirmed on the ambulance call report — usually noted in the list of procedures and text.<br><br>ACR - Clinical Treatment / Procedures & Results.
p_pasamg	textbox	Total Dose (mg)		The total in mg of all doses of ASA administered to patient throughout the prehospital course of care.

Variable	Type	Caption	List Options	Abstraction Instruction
				Â· Usual dose of ASA is 160 mg (2x80 mg) for the patient to chew and swallow. This dose may be administered regardless of any previous ASA self-administration.<br><br>ACR - Clinical Treatment / Procedures & Results.
p_pnitro2	dropdown	Nitrolingual Spray Given	listid: nynr<br><br>0. no<br>1. yes<br>2. not recorded	Indicate if Nitrolingual spray was administered at any time during the prehospital course of care.<br><br>0=no<br>1=yes<br>2=not recorded<br><br>ACR - Clinical Treatment / Procedures & Results.<br><br>Nitrolingual Spray 0.4 mg/spray Code 615<br><br>NOTE –<br>Where only partial EMS documentation is available to the coordinator (as where the ALS or BLS record is missing), mark ''yes'' for those drug therapies for which documentation is available.<br><br>Where only partial EMS documentation is available to the coordinator (as where the ALS or BLS record is missing), leave ''not recorded'' and ''yes'' blank for a prehospital drug therapy for which the skill-related documentation is missingâ€"then override the error message and indicate which documentation is missing. In most cases this response option will be used when the ALS record is missing.<br><br>DO NOT list any drug therapy provided prior to arrival of any EMS personnel in the organized response.
p_pnitroftm	textbox	First Given Time		The earliest time noted when administration of Nitrolingual spray is confirmed on the ambulance call report – usually noted in the list of procedures and text.<br><br>ACR - Clinical Treatment / Procedures & Results.
p_pnitromg1	textbox	Total Dose (mg)		The total in mg of all doses of Nitrolingual spray administered to patient throughout the prehospital course of care.

PREDICT - Prehospital Variables

Variable	Type	Caption	List Options	Abstraction Instruction
				Â· Maximal number of Nitrolingual spray 0.4 mg/spray is 6-8 depending on EMS service medical directive; usually a single spray is administered every 5 minutes.<br><br>Example: If 4 sprays given of 0.4mg then record 1.6 mg<br><br>ACR - Clinical Treatment / Procedures & Results.
p_pmor2	dropdown	Morphine Given	listid: nynr<br><br>0. no<br>1. yes<br>2. not recorded	Indicate if IV Morphine Sulphate (2 mg) was administered at any time during the prehospital course of care.<br><br>0=no<br>1=yes<br>2=not recorded<br><br>ACR - Clinical Treatment / Procedures & Results.<br><br>Morphine Code 604<br><br>NOTE —<br>Where only partial EMS documentation is available to the coordinator (as where the ALS or BLS record is missing), mark ''yes'' for those drug therapies for which documentation is available.<br><br>Where only partial EMS documentation is available to the coordinator (as where the ALS or BLS record is missing), leave ''not recorded'' and ''yes'' blank for a prehospital drug therapy for which the skill-related documentation is missingâ€”then override the error message and indicate which documentation is missing. In most cases this response option will be used when the ALS record is missing.<br><br>DO NOT list any drug therapy provided prior to arrival of any EMS personnel in the organized response.

Variable	Type	Caption	List Options	Abstraction Instruction
p_morftm	textbox	First Given Time		The earliest time noted when administration of IV Morphine Sulphate is confirmed on the ambulance call report – usually noted in the list of procedures and text.<br><br>ACR - Clinical Treatment / Procedures & Results.
p_pmormg	textbox	Total Dose (mg)		The total in mg of all doses of IV Morphine Sulphate administered to patient throughout the prehospital course of care.<br><br>Â· Maximum of 3 doses (single dose 2 mg; total of 6 mg for 3 doses) of IV Morphine Sulphate may be administered in accordance with most medical directives; usually a single dose is administered every 5 minutes.<br><br>ACR - Clinical Treatment / Procedures & Results.
p_pepi2	dropdown	Epinephrine Given	listid: nynr<br><br>0. no<br>1. yes<br>2. not recorded	Indicate if epinephrine was administered at any time during the prehospital course of care.<br><br>0=no<br>1=yes<br>2=not recorded<br><br>ACR - Clinical Treatment / Procedures & Results.<br><br>Epinephrine Code 540 or 541<br><br>NOTE –<br>Where only partial EMS documentation is available to the coordinator (as where the ALS or BLS record is missing), mark ''yes'' for those drug therapies for which documentation is available.<br><br>Where only partial EMS documentation is available to the coordinator (as where the ALS or BLS record is missing), leave ''not recorded'' and ''yes'' blank for a prehospital drug therapy for which the skill-related documentation is missingâ€"then override the error message and indicate which documentation is missing. In most cases this response option will be used when the ALS record is missing.

Variable	Type	Caption	List Options	Abstraction Instruction
				DO NOT list any drug therapy provided prior to arrival of any EMS personnel in the organized response.
p_pepftm	textbox	First Given Time		The earliest time noted when administration of Epinephrine is confirmed on the ambulance call report – usually noted in the list of procedures and text.<br><br>ACR - Clinical Treatment / Procedures & Results.
p_pepimg	textbox	Total Dose (mg)		The total in mg of all doses of Epinephrine administered to patient throughout the prehospital course of care.<br><br>ACR - Clinical Treatment / Procedures & Results.<br><br>NOTE: Epinephrine is supplied in two volumes 1:10,000 and 1:1,000. The units of measurement used by the EMS crew can be mg, ml, amp or units. Please use the following conversions to determine the total volume of epinephrine given in mg:<br><br>Â· 1:10, 000 reported in mg - no conversion<br>Â· 1:10,000 reported in ml - # ml x 0.1 mg = # mg<br>Â· 1:10,000 reported in amp - # amp x 1.0 mg = # mg<br>Â· 1:1,000 reported in mg – no conversion<br>Â· 1:1,000 reported in ml - # ml x 1.0 mg = # mg<br>Â· 1:1,000 reported in units - # units x 0.1 mg = # mg<br>Â· 1:1,000 reported in amp - # amp x 1 mg = # mg
p_pepiiv	dropdown	IV	listid: nynr<br><br>0. no<br>1. yes<br>2. not recorded	Did patient receive Epinephrine via IV?<br><br>0=no<br>1=yes<br>2=not recorded<br><br>ACR - Clinical Treatment / Procedures & Results.
p_pepiet	dropdown	ET/ETT	listid: nynr<br><br>0. no<br>1. yes	Did patient receive Epinephrine via ETT (“down the tube”)?<br><br>0=no

Variable	Type	Caption	List Options	Abstraction Instruction
			2. not recorded	1=yes<br>2=not recorded<br><br>ACR - Clinical Treatment / Procedures & Results.
p_pepiio	dropdown	IO	listid: nynr<br><br>0. no<br>1. yes<br>2. not recorded	Did patient receive Epinephrine via IO (Intraosseous)?<br><br>0=no<br>1=yes<br>2=not recorded<br><br>ACR - Clinical Treatment / Procedures & Results.<br><br>Route indicated for any Epinephrine given is IO or intraosseous.
p_pamio2	dropdown	Amiodarone Given	listid: nynr<br><br>0. no<br>1. yes<br>2. not recorded	Did patient receive Amiodarone during the prehospital course of care?<br><br>0=no<br>1=yes<br>2=not recorded<br><br>ACR - Clinical Treatment / Procedures & Results.<br><br>Indicate if amiodarone was administered at any time during the prehospital course of care.<br><br>Amiodarone Code 502<br><br>NOTE —<br>Where only partial EMS documentation is available to the coordinator (as where the ALS or BLS record is missing), mark ''yes'' for those drug therapies for which documentation is available.<br><br>Where only partial EMS documentation is available to the coordinator (as where the ALS or BLS record is missing), leave ''not recorded'' and ''yes'' blank for a prehospital drug therapy for which the skill-related documentation is missingâ€"then override the error message and indicate which documentation is missing. In most cases this response option will be used when the ALS record is missing.<br><br>DO NOT list any drug therapy provided prior to arrival of any EMS personnel in the organized response.

Variable	Type	Caption	List Options	Abstraction Instruction
p_pamftm	textbox	First Given Time		The earliest time noted when administration of Amiodarone is confirmed on the ambulance call report – usually noted in the list of procedures and text.<br><br>ACR - Clinical Treatment / Procedures & Results.
p_pamimg	textbox	Total Dose (mg)		The total in mg of all doses of Amiodarone administered to patient throughout the prehospital course of care.<br><br>ACR - Clinical Treatment / Procedures & Results.<br><br>NOTE: Amiodarone is supplied in two volumes 1:10,000 and 1:1,000. The units of measurement used by the EMS crew can be mg, ml, amp or units. Please use the following conversions to determine the total volume of Amiodarone given in mg:<br><br>1:10,000 reported in mg - no conversion<br>1:10,000 reported in ml - # ml x 0.1 mg = # mg<br>1:10,000 reported in amp - # amp x 1.0 mg = # mg<br>1:1,000 reported in mg - no conversion<br>1:1,000 reported in ml - # ml x 1.0 mg = # mg<br>1:1,000 reported in units - # units x 0.1 mg = # mg<br>1:1,000 reported in amp - # amp x 1 mg = # mg
p_patro2	dropdown	Atropine Given	listid: nynr<br><br>0. no<br>1. yes<br>2. not recorded	Did patient receive Atropine during the course of prehospital patient care?<br><br>0=no<br>1=yes<br>2=not recorded<br><br>ACR - Clinical Treatment / Procedures & Results.<br><br>Indicate if atropine was administered at any time during the prehospital course of care.<br><br>Atropine Code 505

PREDICT - Prehospital Variables

Variable	Type	Caption	List Options	Abstraction Instruction
				NOTE — Where only partial EMS documentation is available to the coordinator (as where the ALS or BLS record is missing), mark ''yes'' for those drug therapies for which documentation is available. <br>Where only partial EMS documentation is available to the coordinator (as where the ALS or BLS record is missing), leave ''not recorded'' and ''yes'' blank for a prehospital drug therapy for which the skill-related documentation is missingâ€”then override the error message and indicate which documentation is missing. In most cases this response option will be used when the ALS record is missing. <br>DO NOT list any drug therapy provided prior to arrival of any EMS personnel in the organized response.
p_patftm	textbox	First Given Time		The earliest time noted when administration of Atropine is confirmed on the ambulance call report — usually noted in the list of procedures and text. <br>ACR - Clinical Treatment / Procedures & Results.
p_patrmg	textbox	Total Dose (mg)		The total in mg of all doses of Atropine administered to patient throughout the prehospital course of care. <br>ACR - Clinical Treatment / Procedures & Results. <br>NOTE: Atropine is supplied in two volumes 1:10,000 and 1:1,000. The units of measurement used by the EMS crew can me mg, ml, amp or units. Please use the following conversions to determine the total volume of Atropine given in mg: <br>1:10,000 reported in mg - no conversion 1:10,000 reported in ml - # ml x 0.1 mg = # mg 1:10,000 reported in amp - # amp x 1.0 mg = # mg 1:1,000 reported in mg - no conversion 1:1,000 reported in ml - # ml x 1.0 mg = # mg

PREDICT - Prehospital Variables

Variable	Type	Caption	List Options	Abstraction Instruction
				1:1,000 reported in units - # units x 0.1 mg = # mg<br>1:1,000 reported in amp - # amp x 1 mg = # mg
p_plido2	dropdown	Lidocaine	listid: nynr<br><br>0. no<br>1. yes<br>2. not recorded	Did patient receive Lidocaine during the course of prehospital patient care?<br><br>0=no<br>1=yes<br>2=not recorded<br><br>ACR - Clinical Treatment / Procedures & Results.<br><br>Indicate if lidocaine was administered at any time during the prehospital course of care.<br><br>Lidocaine Code 591<br><br>NOTE –<br>Where only partial EMS documentation is available to the coordinator (as where the ALS or BLS record is missing), mark ''yes'' for those drug therapies for which documentation is available.<br><br>Where only partial EMS documentation is available to the coordinator (as where the ALS or BLS record is missing), leave ''not recorded'' and ''yes'' blank for a prehospital drug therapy for which the skill-related documentation is missingâ€”then override the error message and indicate which documentation is missing. In most cases this response option will be used when the ALS record is missing.<br><br>DO NOT list any drug therapy provided prior to arrival of any EMS personnel in the organized response.
p_pliftm	textbox	First Given Time		The earliest time noted when administration of Lidocaine is confirmed on the ambulance call report – usually noted in the list of procedures and text.<br><br>ACR - Clinical Treatment / Procedures & Results.

Variable	Type	Caption	List Options	Abstraction Instruction
p_plidmg	textbox	Total Dose (mg)		The total of all doses of Lidocaine administered to patient throughout the prehospital course of care.<br><br>ACR - Clinical Treatment / Procedures & Results.<br><br>NOTE: Lidocaine is supplied in two volumes 1:10,000 and 1:1,000. The units of measurement used by the EMS crew can be mg, ml, amp or units. Please use the following conversions to determine the total volume of Lidocaine given in mg:<br><br>1:10,000 reported in mg - no conversion<br>1:10,000 reported in ml - # ml x 0.1 mg = # mg<br>1:10,000 reported in amp - # amp x 1.0 mg = # mg<br>1:1,000 reported in mg - no conversion<br>1:1,000 reported in ml - # ml x 1.0 mg = # mg<br>1:1,000 reported in units - # units x 0.1 mg = # mg<br>1:1,000 reported in amp - # amp x 1 mg = # mg
p_pdopa2	dropdown	Dopamine	listid: nynr<br><br>0. no<br>1. yes<br>2. not recorded	Did patient receive Dopamine during the course of prehospital patient care?<br><br>0=no<br>1=yes<br>2=not recorded<br><br>ACR - Clinical Treatment / Procedures & Results.<br><br>Indicate if Dopamine was administered at any time during the prehospital course of care.<br><br>Dopamine Code 536<br><br>NOTE –<br>Where only partial EMS documentation is available to the coordinator (as where the ALS or BLS record is missing), mark ''yes'' for those drug therapies for which documentation is available.

PREDICT - Prehospital Variables

Variable	Type	Caption	List Options	Abstraction Instruction
				Where only partial EMS documentation is available to the coordinator (as where the ALS or BLS record is missing), leave ''not recorded'' and ''yes'' blank for a prehospital drug therapy for which the skill-related documentation is missingâ€”then override the error message and indicate which documentation is missing. In most cases this response option will be used when the ALS record is missing.<br><br>DO NOT list any drug therapy provided prior to arrival of any EMS personnel in the organized response.
p_pdoftm	textbox	First Given Time		The earliest time noted when administration of IV Dopamine is confirmed on the ambulance call report – usually noted in the list of procedures and text.<br><br>ACR - Clinical Treatment / Procedures & Results.
p_pdopmg	textbox	Total Dose (mg)		The total of all doses of IV Dopamine administered to patient throughout the prehospital course of care.<br><br>ACR - Clinical Treatment / Procedures & Results.<br><br>Dopamine is usually started with IV administration of 5 Âµg/kg and then tittred to up to 20 Âµg/kg.
p_pflytic	dropdown	Fibrinolytic Given	listid: nynr<br><br>0. no<br>1. yes<br>2. not recorded	Did patient recieve fibrinolytic therepy during the course of prehospital patient care? This may be recorded as TNK, tPA (TPA ot alteplase), RPA or thrombolytic.<br><br>0=no<br>1=yes<br>2=not recorded<br><br>ACR - Clinical Treatment / Procedures & Results.<br>NOTE –<br>Where only partial EMS documentation is available to the coordinator (as where the ALS or BLS record is missing), mark ''yes'' for those drug therapies for which documentation is available.

PREDICT - Prehospital Variables

Variable	Type	Caption	List Options	Abstraction Instruction
				Where only partial EMS documentation is available to the coordinator (as where the ALS or BLS record is missing), leave ''not recorded'' and ''yes'' blank for a prehospital drug therapy for which the skill-related documentation is missingâ€”then override the error message and indicate which documentation is missing. In most cases this response option will be used when the ALS record is missing.<br><br>DO NOT list any drug therapy provided prior to arrival of any EMS personnel in the organized response.
p_pflyticinel	dropdown	Fibrinolytic Ineligible	listid: ny<br><br>0. no<br>1. yes	Please specify the reason why patient was deemed ineligible to receive fibrinolytic therepy during the course of prehospital patient care.<br><br>ACR - Clinical Treatment / Procedures & Results.
p_pflyticinelsp	textbox	Specify Reason		Please specify the reason why patient was deemed ineligible to receive fibrinolytic therepy during the course of prehospital patient care.<br><br>ACR - Clinical Treatment / Procedures & Results.
p_pflyticstartdt	textbox	Fibrinolysis Start Date		What was the fibrinolysis start date during the course of prehospital patient care?<br><br>Date value = yyyy/mm/dd<br><br>ACR - Clinical Treatment / Procedures & Results.
p_pflyticstarttm	textbox	Fibrinolysis Start Time		What was the fibrinolysis start time during the course of prehospital patient care?<br><br>Numerical value based on 24 hour clock<br><br>00:00:00 – hour:min:sec<br><br>If no value for seconds data available – do not do not use value 00.<br><br>ACR - Clinical Treatment / Procedures & Results.
p_pflyticdrug	dropdown	Fibrinolytic Drug Given	listid: drug2	Which fibrinolytic drug did patient receive during the course of prehospital patient care?

Variable	Type	Caption	List Options	Abstraction Instruction
			1. TNK<br>2. tPA (TPA)<br>3. Activase (Alteplase)<br>4. RPA<br>5. Other	1=TNK (Tenecteplase, Recombinant TPA) no code<br>2=TPA (Tissue Plasminogen Activator) Code 661<br>3=Activase (Alteplase)<br>4=RPA (Reteplase, Retavase) Code 646<br>5=other<br><br>ACR - Clinical Treatment / Procedures & Results.
p_pflyticdrugsp	textbox	Specify Other		Please specify which other fibrinolytic drug patient received during the course of prehospital patient care.<br><br>ACR - Clinical Treatment / Procedures & Results.
p_pflytictotd	textbox	Total Dose Given (mg)		Indicate the total in mg of TNK administered to patient throughout the course of prehospital patient care.<br><br>ACR - Clinical Treatment / Procedures & Results.<br><br>TNK Code<br><br>TNK dose is usually given with IV administration of 30 mg for patients's weight <60 kg; 35 mg for > 60 to < 70 kg; 40 mg for > 70 to < 80 kg; 45 mg for > 80 to < 90 kg; and 50 mg for > 90 kg.
p_pflytictotd2	textbox	Total Dose Given (mg)		Indicate the total in mg of tPA administered to patient throughout the course of prehospital patient care.

Variable	Type	Caption	List Options	Abstraction Instruction
				ACR - Clinical Treatment / Procedures & Results.<br><br>tPA Code 661
p_pflytictotd3	textbox	Total Dose Given (mg)		Indicate the total in mg of Activase (Alteplase) administered to patient throughout the course of prehospital patient care.<br><br>ACR - Clinical Treatment / Procedures & Results.<br><br>Recommended total Activase (Alteplase) dose is based on patient's weight and for AMI the total dose should not exceed 100 mg.<br><br>Activase totsl dose is usually given with IV administration of accelerated infusion (1.5 hours): 15 mg IV bolus for all patients; then: 1) for patients >67 kg 50 mg infused over the next 30 minutes and then 35 mg over the next 60 minutes; 2) for patients ≤67 kg infusion of 0.75 mg/kg over next 30 minutes (not to exceed 50 mg); and then 0.5 mg/kg over 60 minutes (not to exceed 35 mg).<br><br>Activase could also be administered over the 3 hour IV infusion of the total dose of 100 mg: 60 mg in the first hour (bolus dose of 6-10 mg); 20 mg over the second hour and 20 mg over the third hour. For smaller patients (≤65 kg) a dose of 1.25 mg/kg will be administered over the period of 3 hours.
p_pflytictotd4	textbox	Total Dose Given (U)		Indicate the total dose of RPA administered to patient during the ED course of care.<br><br>Source = Patient follow up information / ED chart<br><br>RPA dose is usually given with IV administration of 10 U bolus over 2 mintes; 30 minutes later give second 10 U IV bolus over 2 minutes (give NS flush before and after each bolus); give heparin and aspirin conjuctively.
p_pflytictotd5	textbox	Total Dose Given		Indicate the total of other fibrinolytic drug patient received throughout the course of prehospital patient care.<br><br>ACR - Clinical Treatment / Procedures & Results.

PREDICT - Prehospital Variables

Variable	Type	Caption	List Options	Abstraction Instruction
p_pflyticinter	dropdown	Fibrinolysis Interrupted	listid: ny<br><br>0. no<br>1. yes	Was the fibrinolysis administration interrupted during the course of prehospital patient care?<br><br>0=no<br>1=yes<br><br>ACR - Clinical Treatment / Procedures & Results.
p_pflyticinrep	dropdown	Fibrinolysis Repeated	listid: ny<br><br>0. no<br>1. yes	Was the fibrinolysis administration repeated during the course of prehospital patient care?<br><br>0=no<br>1=yes<br><br>ACR - Clinical Treatment / Procedures & Results.
p_pflyticenddt	textbox	Fibrinolysis End Date		What was the fibrinolysis end date during the course of prehospital patient care?<br><br>Date value = yyyy/mm/dd<br><br>ACR - Clinical Treatment / Procedures & Results.
p_pflyticendtm	textbox	Fibrinolysis End Time		What was the fibrinolysis end time during the course of prehospital patient care?<br><br>Numerical value based on 24 hour clock<br><br>00:00:00 – hour:min:sec<br><br>If no value for seconds data available – do not do not use value 00.<br><br>ACR - Clinical Treatment / Procedures & Results.
p_punfhep	dropdown	Unfractionated Heparin Given	listid: nynr<br><br>0. no<br>1. yes<br>2. not recorded	Did patient recive IV Unfractionated Heparin during the course of prehospital patient care?<br><br>0=no<br>1=yes<br>2=not recorded<br><br>ACR - Clinical Treatment / Procedures & Results<br><br>Indicate if IV Unfractionated Heparin was administered at any time during the prehospital course of care.<br><br>Unfractionated Heparin Code 570<br>NOTE –

PREDICT - Prehospital Variables

Variable	Type	Caption	List Options	Abstraction Instruction
				Where only partial EMS documentation is available to the coordinator (as where the ALS or BLS record is missing), mark ''yes'' for those drug therapies for which documentation is available.<br><br>Where only partial EMS documentation is available to the coordinator (as where the ALS or BLS record is missing), leave ''not recorded'' and ''yes'' blank for a prehospital drug therapy for which the skill-related documentation is missingâ€”then override the error message and indicate which documentation is missing. In most cases this response option will be used when the ALS record is missing.<br><br>DO NOT list any drug therapy provided prior to arrival of any EMS personnel in the organized response.
p_punfhepibdt	textbox	Initial Bolus Date		The date when administration of IV Unfractionated Heparin initial bolus dose is confirmed on the ambulance call report — usually noted in the list of procedures and text.<br><br>ACR - Clinical Treatment / Procedures & Results
p_punfhepibtm	textbox	Initial Bolus Time		The earliest time noted when administration of IV Unfractionated Heparin initial bolus dose is confirmed on the ambulance call report — usually noted in the list of procedures and text.<br><br>ACR - Clinical Treatment / Procedures & Results.
p_punhepibdu	textbox	Initial Bolus Dose (U)		The total in units of IV Unfractionated Heparin initial bolus dose administered to patient throughout the prehospital course of care.<br><br>ACR - Clinical Treatment / Procedures & Results.<br><br>Unfractionated Heparin initial bolus dose is usually started with IV administration of 60 U/kg to maximum of 4000 U in conjuction with thrombolytics or 80 U/kg to maximum of 5000 U when no trombolytics were administered.

PREDICT - Prehospital Variables

Variable	Type	Caption	List Options	Abstraction Instruction
p_punfhepidt	textbox	Infusion Date		The date when administration of IV Unfractionated Heparin infusion is confirmed on the ambulance call report – usually noted in the list of procedures and text.<br><br>ACR - Clinical Treatment / Procedures & Results.
p_punfhepitm	textbox	Infusion Time		The earliest time noted when administration of IV Unfractionated Heparin infusion is confirmed on the ambulance call report – usually noted in the list of procedures and text.<br><br>ACR - Clinical Treatment / Procedures & Results.
p_punhepidu	textbox	Infusion Dose (U)		The total in units of IV Unfractionated Heparin infusion dose administered to patient throughout the prehospital course of care.<br><br>ACR - Clinical Treatment / Procedures & Results.<br><br>Unfractionated Heparin initial bolus dose is usually started with IV administration of 12 U/kg/hr to maximum of 1000 U/hr in conjuction with fibrinolytics or 18 U/kg/hr to maximum of 1500 U/hr when no fibrinolytics were administered.<br><br>Dose is adjusted to maintain PTT at 1.5 – 2 times control value.
p_penox	dropdown	Enoxaparin Given	listid: nynr<br><br>0. no<br>1. yes<br>2. not recorded	Did patient receive Enoxaparin during the course of prehospital patient care?<br><br>0=no<br>1=yes<br>2=not recorded<br><br>ACR - Clinical Treatment / Procedures & Results.<br><br>Low Molecular Weight Heparin Code<br>NOTE –<br>Where only partial EMS documentation is available to the coordinator (as where the ALS or BLS record is missing), mark ''yes'' for those drug therapies for which documentation is available.

Variable	Type	Caption	List Options	Abstraction Instruction
				Where only partial EMS documentation is available to the coordinator (as where the ALS or BLS record is missing), leave ''not recorded'' and ''yes'' blank for a prehospital drug therapy for which the skill-related documentation is missingâ€”then override the error message and indicate which documentation is missing. In most cases this response option will be used when the ALS record is missing.<br><br>DO NOT list any drug therapy provided prior to arrival of any EMS personnel in the organized response.
p_penoxdt	textbox	Initial Bolus Date		The date when administration of IV Enoxaparin initial bolus dose is confirmed on the ambulance call report – usually noted in the list of procedures and text.<br><br>ACR - Clinical Treatment / Procedures & Results.
p_penoxtm	textbox	Initial Bolus Time		The earliest time noted when administration of IV Enoxaparin initial bolus dose is confirmed on the ambulance call report – usually noted in the list of procedures and text.<br><br>ACR - Clinical Treatment / Procedures & Results.
p_penoxibdmg	textbox	Initial Bolus Dose (mg)		The total in mg of IV Enoxaparin initial bolus dose administered to patient throughout the prehospital course of care.<br><br>ACR - Clinical Treatment / Procedures & Results.<br><br>Enoxaparin initial bolus dose is usually started with IV administration of 30 mg for patients < 75 years of age. Patients ≥ 75 years of age do not receive Enoxaparin IV bolus.
p_penoxidt	textbox	Infusion Date		The date when administration of subcutaneous (SC) Enoxaparin infusion is confirmed on the ambulance call report – usually noted in the list of procedures and text.<br><br>ACR - Clinical Treatment / Procedures & Results.
p_penoxitm	textbox	Infusion Time		The earliest time noted when administration of SC Enoxaparin infusion is confirmed on the ambulance call report – usually noted in the list of procedures and text.

PREDICT - Prehospital Variables

Variable	Type	Caption	List Options	Abstraction Instruction
				ACR - Clinical Treatment / Procedures & Results.
p_penoxidmg	textbox	Infusion Dose (mg)		The total in mg of SC Enoxaparin infusion dose administered to patient thoughout the prehospital course of care.<br><br>ACR - Clinical Treatment / Procedures & Results.<br><br>Enoxaparin infusion dose is usually started with SC administration of 1 mg/kg (maximum 100 mg) for patients < 75 years of age and 0.75 mg/kg (maximum 75 mg) for patients ≥ 75 years of age.
p_clop	dropdown	Clopidogrel (Plavix)	listid: nynr<br><br>0. no<br>1. yes<br>2. not recorded	Did patient receive Clopidogrel during the prehospital course of patient care?<br><br>0=no<br>1=yes<br>2=not recorded<br><br>ACR - Clinical Treatment / Procedures & Results.<br><br>NOTE —<br>Where only partial EMS documentation is available to the coordinator (as where the ALS or BLS record is missing), mark ''yes'' for those drug therapies for which documentation is available.<br><br>Where only partial EMS documentation is available to the coordinator (as where the ALS or BLS record is missing), leave ''not recorded'' and ''yes'' blank for a prehospital drug therapy for which the skill-related documentation is missingâ€"then override the error message and indicate which documentation is missing. In most cases this response option will be used when the ALS record is missing.<br><br>DO NOT list any drug therapy provided prior to arrival of any EMS personnel in the organized response.<br><br>Indicate if Clopidogrel was administered at any time during during the prehospital course of patient care.

Variable	Type	Caption	List Options	Abstraction Instruction
p_cloptm	textbox	First Given Time		The earliest time noted when administration of Clopidogrel is confirmed during the prehospital course of patient care.<br><br>ACR - Clinical Treatment / Procedures & Results
p_cloptotd	textbox	Total Dose Given (mg)		Indicate the total in mg of Clopidogrel administered to patient throughout the prehospital course of care.<br><br>ACR - Clinical Treatment / Procedures & Results<br><br>Clopidogrel dose is usually given with a loading dose of 300 mg for 75 years and under, and 75 mg for 76 years and older. The decision to administer additional clopidogrel to patients is usually left to the discretion of the interventional cardiologist.
p_glycinh	dropdown	Glycoprotein (GP) llb/llla Inhibitors	listid: nynr<br><br>0. no<br>1. yes<br>2. not recorded	Did patient recive IV glycoprotein IIb/IIIa inhibitors during the prehospital course of patient care?<br><br>0=no<br>1=yes<br>2=not recorded<br><br>ACR - Clinical Treatment / Procedures & Results<br><br>NOTE –<br>Where only partial EMS documentation is available to the coordinator (as where the ALS or BLS record is missing), mark ''yes'' for those drug therapies for which documentation is available.<br><br>Where only partial EMS documentation is available to the coordinator (as where the ALS or BLS record is missing), leave ''not recorded'' and ''yes'' blank for a prehospital drug therapy for which the skill-related documentation is missingâ€”then override the error message and indicate which documentation is missing. In most cases this response option will be used when the ALS record is missing.<br><br>DO NOT list any drug therapy provided prior to arrival of any EMS personnel in the organized response.

PREDICT - Prehospital Variables

Variable	Type	Caption	List Options	Abstraction Instruction
				Indicate if IV Glycoprotein IIb/IIIa Inhibitors were administered at any time during during the prehospital course of patient care.
p_glycinhtype	dropdown	Glycoprotein (GP) llb/llla Inhibitors Type	listid: glycinh2<br><br>1. Abciximab (ReoPro)<br>2. Eptifibatide (Intergrilin)<br>3. Tirofiban (Aggrastat)<br>4. Other	What type of glycoprotein IIb/IIa inhibitor did patient recive during the prehospital course of patient care?<br><br>1=Abeiximab (ReoPro)<br>2=Eptifibatide (Integrilin)<br>3=Tirofiban (Aggrastat)<br>4=other (specify)<br><br>ACR - Clinical Treatment / Procedures & Results<br><br>Indicate what type of Glycoprotein IIb/IIIa Inhibitor was administered at any time during during the prehospital course of patient care.
p_glycinhtypesp	textbox	Specify Other		Please specify which other glycoprotein IIb/IIa inhibitor patient received during the course of prehospital patient care.<br><br>ACR - Clinical Treatment / Procedures & Results.
p_glycinhtm	textbox	First Given Time		The earliest time noted when administration of glycoprotein IIb/IIa inhibitor is confirmed during the prehospital course of patient care.<br><br>ACR - Clinical Treatment / Procedures & Results
p_glycinhtotd	textbox	Total Dose Given (mg)		Indicate the total in mg of Abciximab administered to patient throughout the prehospital course of care.<br><br>ACR - Clinical Treatment / Procedures & Results<br><br>Abciximab dose is usually given with IV bolus administration of 0.25 mg/kg 10-60 minutes before the start of PCI, followed by a continuous intravenous infusion of 0.125 Âµg/kg/min (to a maximum of 10 Âµg/min) for 12 hours.
p_glycinhtotd2	textbox	Total Dose Given (mg)		Indicate the total in mg of Eptifibatide administered to patient throughout the prehospital course of care.

PREDICT - Prehospital Variables

Variable	Type	Caption	List Options	Abstraction Instruction
				ACR - Clinical Treatment / Procedures & Results<br><br>Eptifibatide dose is usually given with IV bolus administration of 180 Âµg/kg as soon as possible followed by a continuous infusion of 2.0 Âµg/kg/min until hospital discharge or initiation of CABG surgery, up to 72 hours. If a patient is to undergo a percutaneous coronary intervention (PCI) while receiving eptifibatide, the infusion should be continued up to hospital discharge, or for up to 18 to 24 hours after the procedure, whichever comes first, allowing for up to 96 hours of therapy .
p_glycinhtotd3	textbox	Total Dose Given (mg)		Indicate the total dose of Tirofiban administered to patient during the ED course of care.<br><br>ACR - Clinical Treatment / Procedures & Results<br><br>Tirofiban dose is usually given with IV at an initial rate of 0.4 Âµg/kg/min for 30 minutes and then continued at 0.1 Âµg/kg/min.
p_glycinhtotd4	textbox	Total Dose Given (mg)		Indicate the total in mg of the other of glycoprotein IIb/IIa inhibitor administered to patient throughout the prehospital course of care.<br><br>ACR - Clinical Treatment / Procedures & Results
**p_pd**	**section**	**Prehospital Disposition**		
p_pdisp	dropdown	Disposition	listid: disp<br><br>0. died at scene or enroute<br>1. transported by EMS to ED/hospital with ROSC or ongoing resuscitation<br><br>2. alive and not transported by EMS to ED/hospital	ACR - Clinical Treatment / Procedures & Results<br><br>if not mention go to<br><br>ACR – General Administration<br><br>if no mention go to<br><br>ACR – Physical Exam – Deceased

PREDICT - Prehospital Variables

Variable	Type	Caption	List Options	Abstraction Instruction
				AACR - Clinical Information – C.T.A.S. Deceased – Pronounced by BHP – Time of pronouncement and/or Clinical Treatment / Procedures & Results – Final Status<br><br>AACR - where “Final Status” is coded as 8 (Died enroute) or 9 (Died at scene)<br><br>Indicated by check mark in the Pronounced Dead By BHP or “Obviously Dead” check box.
p_pemstx	dropdown	Treated by EMS	listid: ny<br><br>0. no<br>1. yes	ACR - Clinical Information , Physical Exam, and/or Clinical Treatment / Procedures & Results<br><br>AACR - Clinical Information – C.T.A.S. Deceased and/or Clinical Treatment / Procedures & Results<br><br>Indicated by check mark in the “Obviously Dead” check box and Code 200 (CPR being performed by EMS).
p_pyhalt	dropdown	Why was Treatment Halted	listid: treatment<br><br>1. considered futile<br><br>2. DNR (written or verbal)<br>3. obviously dead	Considered futile: Termination of care at discretion or with judgment by the responder and/or medical control.<br>DNR (written or verbal): Directive to not resuscitate the patient, either verbal or in writing, by the patient, family, or guardian<br><br>ACR - Clinical Information , Physical Exam, and/or Clinical Treatment / Procedures & Results<br><br>AACR - Clinical Information – C.T.A.S. Deceased and/or Clinical Treatment / Procedures & Results
p_pynotx	dropdown	Why Not Treated by EMS	listid: treatment<br><br>1. considered futile<br><br>2. DNR (written or verbal)<br>3. obviously dead	Considered futile: Termination of care at discretion or with judgment by the responder and/or medical control.<br>DNR (written or verbal): Directive to not resuscitate the patient, either verbal or in writing, by the patient, family, or guardian<br><br>ACR - Clinical Information , Physical Exam, and/or Clinical Treatment / Procedures & Results<br><br>AACR - Clinical Information – C.T.A.S. Deceased and/or Clinical Treatment / Procedures & Results<br><br>Considered Futile – Not used in Ontario

PREDICT - Prehospital Variables

Variable	Type	Caption	List Options	Abstraction Instruction
				DNR - text indicating this patient had a valid written or verbal Do Not Resuscitate (DNR) order<br><br>Legally per local legislation- Indicated by check mark in the "Obviously Dead" check box.
p_ptrans	dropdown	Transport Mode	listid: trans<br>1. by land<br>2. by air	Where a patient has been transported by both land and air, select Air<br><br>ACR – General Administration<br><br>If a patient was taken to ED then "Yes" regardless of vital status.
p_prosc	dropdown	Patient Status at ED Arrival	listid: p_prosc<br>1. much better<br>2. moderately better<br>3. slightly better<br>4. No change<br>5. Slightly Worse<br>6. Moderately Worse<br>7. Much Worse<br>8. Not Recorded	Based on the paramedic record what was the final patient status on the 7 point scale at the time of transferr to the ED staff?<br><br>1=much better<br>2=moderately better<br>3=slightly better<br>4=no change<br>5=slightly worse<br>6=moderately worse<br>7=much worse<br>8=not recorded<br><br>ACR/AACR General Administration section page 2 bottom left FINAL STATUS.
p_pfinprimprob	textbox	Final Primary Problem		Based on the paramedic record what was recorded as the final primary problem?<br><br>ACR/AACR General Administration section page 2 bottom left FINAL PRIMARY PROBLEM
p_pprobcode	textbox	Problem Code		Based on the paramedic record what was recorded as the primary problem code?<br><br>ACR/AACR General Administration section page 2 bottom left PROBLEM CODE
**p_ehi**	**section**	**ED & Hospital Information**		
p_hospid	dropdown	ID of ED/Hospital Transported to	listid: p_hosp<br>** See list items in appendix	ED Ministry of Health and Long Term Care Name
p_edaha	div	ED Arrival / Hospital Admission		

PREDICT - Prehospital Variables

Variable	Type	Caption	List Options	Abstraction Instruction
p_admdt	textbox	Date		What was the date of the patients ED arrival / hospital admission?<br><br>Date value = yyyy/mm/dd<br><br>Source = Patient follow up information / Hospital chart
p_admtm	textbox	Time		What was the time of the patients ED arrival / hospital admission?<br><br>00:00:00 – hour:min:sec<br><br>Source = Patient follow up information / Hospital chart
p_ptxfer	dropdown	Was Patient Transferred to Another Acute Care Hospital	listid: xfers<br><br>0. no<br>1. yes<br>2. yes - two<br>3. yes - three<br>4. yes - four<br>5. yes - five	After initial admission to ED was patient then transferred to another acute care facility?<br><br>0=no<br>1=yes – 1 transfer<br>2=yes – 2 transfers<br>3=yes – 3 transfers<br>4=yes – 4 transfers<br>5=yes – 5 transfers<br><br>Source = Patient follow up information / Hospital chart
p_edhospno	dropdown	How many transfers?	listid: p_edhospno<br>1. one<br>2. two<br>3. three<br>4. four<br>5. five	
p_1stth	div	1st Transfer Hospital		
p_xhosp1	dropdown	Name	listid: p_hosp<br><br>** See list items in appendix	What is the name of the 1st Hospital Patient was transferred to?<br><br>Source = Patient follow up information / Hospital chart<br><br>Pulldown menu = list of hospitals in database<br><br>Unknown Hospital = select when destination hospital will never be known.<br><br>NOTE;<br>Do not provide the names of nursing home, rehabilitation, or other non-acute care facilities.<br>Transfer to one of these three entities constitutes an ''''ED/hospital discharge, reclassification, or death''''.

PREDICT - Prehospital Variables

Variable	Type	Caption	List Options	Abstraction Instruction
p_xfer1dt	textbox	Date of Transfer		What was the date of the patients transfer to the first transfer hospital?<br><br>Date value = yyyy/mm/dd<br><br>Source = Patient follow up information / Hospital chart
p_2stth	div	2nd Transfer Hospital		
p_xhosp2	dropdown	Name	listid: p_hosp<br><br>** See list items in appendix	What is the name of the 2nd Hospital Patient was transferred to?<br><br>Source = Patient follow up information / Hospital chart<br><br>Pulldown menu = list of hospitals in database<br><br>Unknown Hospital = select when destination hospital will never be known.<br><br>NOTE;<br>Do not provide the names of nursing home, rehabilitation, or other non-acute care facilities.<br>Transfer to one of these three entities constitutes an ''''ED/hospital discharge, reclassification, or death''''.
p_xfer2dt	textbox	Date of Transfer		What was the date of the patients transfer to the second transfer hospital?<br><br>Date value = yyyy/mm/dd<br><br>Source = Patient follow up information / Hospital chart
p_3stth	div	3rd Transfer Hospital		
p_xhosp3	dropdown	Name	listid: p_hosp<br><br>** See list items in appendix	What is the name of the 3rd Hospital Patient was transferred to?<br><br>Source = Patient follow up information<br><br>Pulldown menu = list of hospitals in database<br><br>Unknown Hospital = select when destination hospital will never be known.<br><br>NOTE;<br>Do not provide the names of nursing home, rehabilitation, or other non-acute care facilities.

Variable	Type	Caption	List Options	Abstraction Instruction
				Transfer to one of these three entities constitutes an ''ED/hospital discharge, reclassification, or death''.
p_xfer3dt	textbox	Date of Transfer		What was the date of the patients transfer to the third transfer hospital?<br><br>Date value = yyyy/mm/dd<br><br>Source = Patient follow up information / Hospital chart
p_4stth	div	4th Transfer Hospital		
p_xhosp4	dropdown	Name	listid: p_hosp<br><br>** See list items in appendix	What is the name of the 4th Hospital Patient was transferred to?<br><br>Source = Patient follow up information / Hospital chart<br><br>Pulldown menu = list of hospitals in database<br><br>Unknown Hospital = select when destination hospital will never be known.<br><br>NOTE;<br>Do not provide the names of nursing home, rehabilitation, or other non-acute care facilities.<br>Transfer to one of these three entities constitutes an ''ED/hospital discharge, reclassification, or death''.
p_xfer4dt	textbox	Date of Transfer		What was the date of the patients transfer to the fourth transfer hospital?<br><br>Date value = yyyy/mm/dd<br><br>Source = Patient follow up information / Hospital chart
p_5stth	div	5th Transfer Hospital		
p_xhosp5	dropdown	Name	listid: p_hosp<br><br>** See list items in appendix	What is the name of the 5th Hospital Patient was transferred to?<br><br>Source = Patient follow up information / Hospital chart<br><br>Pulldown menu = list of hospitals in database

PREDICT - Prehospital Variables

Variable	Type	Caption	List Options	Abstraction Instruction
				Unknown Hospital = select when destination hospital will never be known.<br><br>NOTE;<br>Do not provide the names of nursing home, rehabilitation, or other non-acute care facilities.<br>Transfer to one of these three entities constitutes an ''ED/hospital discharge, reclassification, or death''.
p_xfer5dt	textbox	Date of Transfer		What was the date of the patients transfer to the fifth transfer hospital?<br><br>Date value = yyyy/mm/dd<br><br>Source = Patient follow up information / Hospital chart
p_surv	dropdown	Final Vital Status	listid: fvs<br>0. Died<br>1. Alive	Hospital Chart
p_dthloc	dropdown	Where?	listid: edhosp<br><br>1. ED<br>2. Hospital	Where was the patient's final vital status assigned?<br><br>1=ED<br>2=Hospital<br><br>Source = Patient follow up information / Hospital chart

Appendix:

Hospital List

1696. Alexandra Hospital
1206. Alexandra Marine & General Hospital
2057. Blind River District Health
4418. Bluewater Health - CEEH
4415. Bluewater Health - Mitton
1006. Brant Community Healthcare - Brantford
4309. Brant Community Healthcare - Willett
1905. Cambridge Memorial Hospital
1597. Campbellford Memorial Hospital
2173. Chapleau Health Services
4238. Chatham-Kent Health Alliance
1239. Chatham-Kent Health Alliance - Sydenham
4197. Collingwood General and Marine Hospital
2174. Espanola General Hospital
1507. Four Counties Health Services
2175. Geraldton District Hospital
3734. Grand River Hospital - K-W
1030. Grey Bruce Health Services - Lion's Head
4025. Grey Bruce Health Services - Markdale
4027. Grey Bruce Health Services - Meaford
3944. Grey Bruce Health Services - Owen Sound
4030. Grey Bruce Health Services - Southhampton
4033. Grey Bruce Health Services - Wiarton
1936. Groves Memorial Community Hospital
1946. Guelph General Hospital
1146. Haldimand War Memorial Hospital
3737. Haliburton Highlands Health Services - Haliburton
4192. Halton Heathcare Services - Oakville-Trafalgar
4246. Halton Heathcare Services - Georgetown
4193. Halton Heathcare Services - Milton
1982. Hamilton Health Sciences Corporation - General
1983. Hamilton Health Sciences Corporation - Henderson
1994. Hamilton Health Sciences Corporation - McMaster
1124. Hanover & District Hospital
4161. Headwaters Health - Orangeville (Dufferin)
2061. Hornepayne Community Hospital
4260. Humber River Regional Hospital - Church
4089. Humber River Regional Hospital - Finch
1199. Huron Perth Healthcare Alliance - Clinton
1213. Huron Perth Healthcare Alliance - Seaforth
1748. Huron Perth Healthcare Alliance - St. Mary's
1754. Huron Perth Healthcare Alliance - Stratford
4142. HÃ´tel-Dieu Grace Hospital
4144. Joseph Brant Memorial Hospital
2211. Kirkland & District Hospital, Kirkland Lake
2076. Lady Dunn Health Centre
2078. Lady Minto Hospital
4171. Lakeridge Health Corporation - Oshawa
4170. Lakeridge Health Corporation - Bowmanville
4172. Lakeridge Health Corporation - Port Perry
1067. Leamington District Hospital
1740. Listowel Memorial Hospital
4310. London Health Sciences Centre - University Hospital
4247. London Health Sciences Centre - Victoria Hospital
2121. Manitoulin Health Centre - Little Current
2123. Manitoulin Health Centre - Mindemoya
2176. Manitouwadge General Hospital
4235. Markham-Stouffville Hospital Corporation
4467. Markham-Stouffville Hospital Corporation - Uxbridge
2126. Mattawa Hospital
4110. Mount Sinai Hospital
4258. Muskoka Algonquin - Huntsville District Memorial Hospital
4320. Muskoka Algonquin - South Muskoka Memorial Hospital
4210. Niagara Health System - Douglas Memorial Hospital
4213. Niagara Health System - Greater Niagara General
4219. Niagara Health System - Port Colborne General

4224. Niagara Health System - St. Catharines General
4227. Niagara Health System - Welland Hospital
2178. Nipigon District Memorial Hospital
1591. Norfolk General Hospital
3411. North Bay General Hospital
4241. Georgian Bay General Hospital (Huronia District Hospital)
4323. North Wellington Health Care - Mount Forest
4326. North Wellington Health Care - Palmerston
4233. North York General Hospital
4234. North York General Hospital - Branson
3860. Northumberland Hills Hospital
2082. Notre -Dame Hospital Hearst
4108. Orillia Soldiers' Memorial Hospital
1768. Peterborough General Hospital
2115. Red Lake Margaret Cochenour Memorial Hospital
2148. Riverside Health Care Facilities Inc . - Emo
2150. Riverside Health Care Facilities Inc . - Laverendye
2153. Riverside Health Care Facilities Inc . - Rainy River
4177. Ross Memorial Hospital
4140. Rouge Valley Health System - Ajax Pickering
4139. Rouge Valley Health System - Centennary
2064. Sault Area Hospitals - Mathews Memorial
4407. Sault Area Hospitals - S. S.Marie
2075. Sault Area Hospitals - Thessalon
2088. Sensenbrenner Hospital
4353. Sioux Lookout Meno Ya Win Health Centre
2094. Smooth Rock Falls Hospital
4042. South Bruce Grey Health Centre - Chesley
4036. South Bruce Grey Health Centre - Durham
3907. South Bruce Grey Health Centre - Kincardine
4039. South Bruce Grey Health Centre - Walkerton
1203. South Huron Hospital Alliance
4001. Southlake Regional Health
2058. St. Joseph's General Hospital
4056. St. Joseph's Health Centre
2003. St. Joseph's Healthcare
1921. St. Mary's Hospital
3985. St. Michael's Hospital
1059. St. Thomas Elgin General Hospital
4249. The Stevenson Memorial Hospital Alliston
1515. Strathroy Middlesex General Hospital
4063. Sudbury Regional Hospital - Laurentian
4069. Sudbury Regional Hospital - Memorial
4066. Sudbury Regional Hospital - St. Joseph's
4205. Sunnybrook Health Sciences Centre
2207. Temiskaming Hospital
3986. The Credit Valley Hospital
2180. The McCausland Hospital
3987. The Royal Victoria Hospital
3975. The Scarborough Hospital - General
3984. The Scarborough Hospital - Grace
3853. Thunder Bay Regional Health Sciences
1709. Tillsonburg District Memorial Hospital
3414. Timmins & District Base Hospital Program
4209. Toronto East General Hospital
4090. Trillium Health Centre
4265. University Health Network - General
4266. University Health Network - Western
1149. West Haldimand General Hospital
1538. West Lincoln Memorial Hospital
2812. West Nipissing General Hospital
4236. West Parry Sound Health Centre
4685. William Osler Health System - Brampton
4245. William Osler Health System - Etobicoke
2177. Wilson Memorial General Hospital
1079. Windsor Regional Hospital - Metropolitan
1217. Wingham & District Hospital
1716. Woodstock General Hospital Trust
4138. York Central Hospital

Services List

0262. Sault Ste Marie EMS
0491. Toronto EMS
0701. Brant EMS
0707. Grey EMS
0720. Muskoka EMS
0722. Norfolk EMS
0724. Durham EMS
0727. Peel EMS
0733. Simcoe EMS
0738. Hamilton EMS
0740. Algoma EMS
0749. Thunder Bay EMS
0753. Haldimand EMS
0789. York EMS
0818. OAASC
1801. Durham Fire Pickering
1805. Durham Fire Ajax
1809. Durham Fire Whitby
1813. Durham Fire Oshawa
1817. Durham Fire Clarington
1820. Durham Fire Scugog
1829. Durham Fire Uxbridge
1839. Durham Fire Brock
2005. Toronto Fire
2105. Peel Fire Mississauga
2110. Peel Fire Brampton
2124. Peel Fire Caledon
2505. Hamilton Fire
4307. Simcoe Fire New Tecumseh
4312. Simcoe Fire Bradford
4317. Simcoe Fire Innisfil
4319. Simcoe Fire Ramara
4321. Simcoe Fire Essa
4323. Simcoe Fire Oro Medonte
4342. Simcoe Fire Barrie
4350. Simcoe Fire Mnjikani
4352. Simcoe Fire Orillia
4364. Simcoe Fire Wasaga
4465. Muskoka Fire Georg
0747. Sudbury EMS
0752. Manitoulin-Sudbury EMS (East Side)
0782. Manitoulin-Sudbury EMS (West Side)
0110. Six Nations Ambulance Service
0433. Central Erie Ems
0788. Hamilton-Wentworth Regional Ambulance Service
0910. Cacc - London
0920. Cacc - Hamilton
0710. Halton EMS
