# Supplementary material for: Prehospital evaluation and economic analysis of different coronary syndrome treatment strategies - PREDICT - Rationale, Development and Implementation
Source: BMC Emerg Med. 2011 Mar 29;11:4. doi: 10.1186/1471-227X-11-4 (PMC3076236; doi:10.1186/1471-227X-11-4)
Supplement: Additional file 2 — PREDICT Hospital Variables - Structured data set with variables abstracted from hospital charts. [file 1471-227X-11-4-S2.PDF]

PREDICT - Hospital Variables

Variable	Type	Caption	List Options	Abstraction Instruction
**p_edform**	**section**	**Emergency Department (ED) form**		
p_pihed	div	Patient Identifiers		
p_ilnameed	textbox	Surname		This field is used to obtain the ED chart version of the patient's surname.<br><br>Transcribe as-is.<br><br>Caps Lock
p_ifnameed	textbox	Given Name		This field is used to obtain the ED chart version of the patient's given name.<br><br>Transcribe as-is.<br><br>Caps Lock
p_iaddred	textbox	Mailing Address		This field is used to obtain the ED chart version of a patient's mailing/street address.<br><br>Transcribe as-is.<br><br>Caps Lock
p_icityed	textbox	City/Town		This field is used to obtain the ED chart version of a patient's city / town.<br><br>Transcribe as-is.<br><br>Caps Lock
p_iproved	dropdown	Province	listid: prov<br><br>1. AB<br>2. BC<br>3. MB<br>4. NB<br>5. NF<br>6. NS<br>7. NT<br>8. NU<br>9. ON<br>10. PE<br>11. QC<br>12. SK<br>13. YT<br>99. Out of Country	This field is used to obtain the ED chart version of a patient's province.<br><br>Transcribe as-is.<br><br>Caps Lock
p_ipostced	textbox	Postal Code		This field is used to obtain the ED chart version of a patient's postal code.<br><br>Transcribe as-is.<br><br>Caps Lock

PREDICT - Hospital Variables

Variable	Type	Caption	List Options	Abstraction Instruction
p_idobed	textbox	Date of Birth		This field is used to obtain the ED chart version of patient's date of birth (MDY).<br><br>Transcribe as-is.<br><br>Caps Lock
p_wghted	dropdown	Weight	listid: uy<br><br>0. unknown/not noted<br><br>1. yes	This field is used to obtain the ED chart version of a patient's weight.<br><br>Transcribe as-is
p_wghtedkg	textbox	Weight (kg)		ACR - Physical Exam – Weight<br><br>AACR - Administration – Weight<br><br>Transcribe as-is<br><br>In the case of multiple vehicles, use the ACR that was completed by the EMS crew that treated and transported the patient.<br><br>In some cases the medic might not be able to ascertain the weight of a patient, i.e., gender=”Unknown”.
p_ihospreg ed	textbox	ED Registration Number/Patient Chart Number		ED chart number from Health Records Department (not from ACR).<br><br>Transcribe as-is.<br><br>Caps Lock
p_phospreg ed	textbox	Hospital Registration Number / Patient Chart Number		Hospital Registration Number<br><br>Transcribe as-is.<br><br>Caps Lock<br><br>Note this field will be not applicable for patients who are not transported to an ED or hospital.
p_hlthcrd	div	Health Card		
p_ihealthc ned	textbox	Number		This field is used to obtain the ED chart version of Health Card No.<br><br>Transcribe as-is
p_ihealthc ved	textbox	Version Code		This field is used to obtain the ED chart version of Version No.<br><br>Transcribe as-is
p_isexed	dropdown	Gender	listid: sex<br><br>0. female	This field is used to obtain the ED chart version of Gender.

PREDICT - Hospital Variables

Variable	Type	Caption	List Options	Abstraction Instruction
			1. male<br>2. not noted	Transcribe as-is
p_iphone1ed	textbox	Phone 1 (Home)		Patient s home telephone number.<br><br>Transcribe as-is
p_iphone2ed	textbox	Phone 2		Another contact number for the patient.<br><br>Transcribe as-is
p_ptred	div	Prehospital/ED Episode Date/Time Record		
p_sodt	textbox	Symptom Onset Date		What was the date of the patient symptom onset?<br><br>Date value = yyyy/mm/dd<br><br>Source = Patient follow up information / ED chart
p_sotm	textbox	Symptom Onset Time		What was the time of the patient symptom onset?<br><br>Numerical value based on 24 hour clock<br><br>00:00:00 – hour:min:sec<br><br>If no value for seconds data available – do not do not use value 00.<br><br>Source = Patient follow up information / ED chart
p_edardt	textbox	ED Arrival Date		What was the date of the patient ED arrival?<br><br>Date value = yyyy/mm/dd<br><br>Source = Patient follow up information / ED chart
p_edartm	textbox	ED Arrival Time		What was the time of the patient ED arrival?<br><br>Numerical value based on 24 hour clock<br><br>00:00:00 – hour:min:sec<br><br>If no value for seconds data available – do not do not use value 00.<br><br>Source = Patient follow up information / ED chart
p_rechosp	dropdown	Receiving Hospital	listid: p_hosp<br><br>** see list items in appendix	What is the name of the Receiving Hospital?<br><br>Source = Patient follow up information

PREDICT - Hospital Variables

Variable	Type	Caption	List Options	Abstraction Instruction
				Pulldown menu = list of hospitals in database<br><br>Unknown Hospital = select when destination hospital will never be known.<br><br>NOTE;<br>Do not provide the names of nursing home, rehabilitation, or other non-acute care facilities.<br>Transfer to one of these three entities constitutes an ''ED/hospital discharge, reclassification, or death''.
p_edecg_div	div	ECG Recording in ED		
p_ecged	dropdown	Does ED ECG Recording Exist?	listid: ny<br><br>0. no<br>1. yes	Did ED personnel record the patient ECG at any time during the ED course of care?<br><br>0=no<br>1=yes<br><br>Indicate no or yes, whether ED ECG recording exists.<br><br>Source = Patient follow up information / ED chart
p_1edecg	div	First ED ECG (first one on arrival in ED)		
p_fedecg	dropdown	Does First ED ECG Recording Exist?	listid: ny<br><br>0. no<br>1. yes	Did ED personnel record the patient ECG on arrival in ED?<br><br>0=no<br>1=yes<br><br>Indicate no or yes, whether ED ECG recording exists.<br><br>Source = Patient follow up information / ED chart
p_fedecgdt	textbox	Date		What was the date of the patient first ECG in ED?<br><br>Date value = yyyy/mm/dd<br><br>Source = Patient follow up information / ED chart
p_fedecgtm	textbox	Time		What was the time of the patient first ECG in ED?<br><br>Numerical value based on 24 hour clock<br><br>00:00:00 – hour:min:sec

PREDICT - Hospital Variables

Variable	Type	Caption	List Options	Abstraction Instruction
				If no value for seconds data available – do not do not use value 00<br><br>Source = Patient follow up information / ED chart
p_efedecgxst	dropdown	Electronic ECG File Exists	listid: ny<br><br>0. no<br>1. yes	Is there an electronic recording of the first ED ECG data available on request?<br><br>0=no<br>1=yes<br><br>Source = Patient follow up information / ED chart
p_fedecgrev	dropdown	First ECG Reviewed	listid: nyu<br><br>0. no<br>1. yes<br>2. unknown	Indicate whether or not the first ED ECG was reviewed by the attending ED physician site or if the attendant annotations and QA data was generated solely by the device software.<br><br>0=no<br>1=yes<br>2=unknown<br><br>The goal is for sites to review the recordings so as to ''correct'' any oversights by the software.<br>Source = Patient follow up information / ED chart
p_fedecgd	dropdown	First ECG Data Exists	listid: ny<br><br>0. no<br>1. yes	Is there the first ED ECG data available on request?<br><br>0=no<br>1=yes<br><br>Source = Patient follow up information / ED chart
p_fedecgdgca	dropdown	First ECG Diagnosis - Computer Assisted	listid: ny<br><br>0. no<br>1. yes	Indicate whether or not there was computer assisted diagnosis for the first ED ECG.<br><br>0=no<br>1=yes<br><br>Source = Patient follow up information / ED chart
p_fedecgdgsp	textbox	First ECG Diagnosis - Computer Assisted - Specify 1		Please specify computer assisted diagnosis for the first ED ECG.<br><br>Value = text<br><br>Source = Patient follow up information / ED chart
p_fedecgdgsp_2	textbox	Diagnosis - Computer Assisted - Specify 2 (cont)		

PREDICT - Hospital Variables

Variable	Type	Caption	List Options	Abstraction Instruction
p_fedecgdgsp_3	textbox	Diagnosis - Computer Assisted - Specify 3 (cont)		
p_fedecgdg	textbox	First ECG Diagnosis 1		Indicate the diagnosis based on patient first ED ECG made by ED attending physician.<br><br>Source = Patient follow up information / ED chart
p_fedecgdg_2	textbox	First ECG Diagnosis 2 (cont)		
p_fedecgdg_3	textbox	First ECG Diagnosis 3 (cont)		
p_stefedecg	dropdown	ST Elevation	listid: nyn<br>0. no<br>1. yes<br>2. not noted	Is there ST elevation on patient first ED ECG?<br><br>0=no<br>1=yes<br><br>Source = Patient follow up information / ED chart
p_stemicrfedecg	dropdown	STEMI Criteria Met	listid: nynr<br><br>0. no<br>1. yes<br>2. not recorded	Is ST Elevation Myocardial Infarction (STEMI) criteria met on patient first ED ECG?<br><br>0=no<br><br>1=yes<br><br>2=not noted<br><br>Source = Patient follow up information / ED chart
p_stemilfedecg	textbox	STEMI Location		Indicate, if possible the STEMI location based on patient first ECG in ED.<br><br>Source = Patient follow up information / ED chart
p_dgedecg	div	Diagnostic ED ECG (prior to fibrinolysis)		
p_dgedecgxst	dropdown	Does Diagnostic ED ECG Recording Exist Just Prior to Reperfusion Therapy?	listid: ny<br><br>0. no<br>1. yes	Did ED personnel record the patient diagnostic ECG in ED just prior to reperfusion therapy?<br><br>0=no<br>1=yes<br><br>Indicate no or yes, whether diagnostic ED ECG recording exists.<br><br>Source = Patient follow up information / ED chart

PREDICT - Hospital Variables

Variable	Type	Caption	List Options	Abstraction Instruction
p_dgedecgdt	textbox	Date		What was the date of the patient diagnostic ECG in ED?<br><br>Date value = yyyy/mm/dd<br><br>Source = Patient follow up information / ED chart
p_dgedecgtm	textbox	Time		What was the time of the patient diagnostic ECG in ED?<br><br>Numerical value based on 24 hour clock<br><br>00:00:00 – hour:min:sec<br><br>If no value for seconds data available – do not do not use value 00<br><br>Source = Patient follow up information / ED chart
p_dgedecgrev	dropdown	Diagnostic ECG Reviewed	listid: nyu<br><br>0. no<br>1. yes<br>2. unknown	Indicate whether or not the diagnostic ED ECG was reviewed by the attending ED physician site or if the attendant annotations and QA data was generated solely by the device software.<br><br>0=no<br>1=yes<br>2=unknown<br><br>The goal is for sites to review the recordings so as to ''correct'' any oversights by the software.<br>Source = Patient follow up information / ED chart
p_dgedecgd	dropdown	Diagnostic ECG Data Exists	listid: ny<br><br>0. no<br>1. yes	Is there the diagnostic ED ECG data available on request?<br><br>0=no<br>1=yes<br><br>Source = Patient follow up information / ED chart
p_dgedecgdgca	dropdown	Diagnostic ECG - Computer Assisted	listid: ny<br><br>0. no<br>1. yes	Indicate whether or not there was computer assisted diagnosis for the diagnostic ED ECG.<br><br>0=no<br>1=yes<br><br>Source = Patient follow up information / ED chart
p_dgedecgdgsp	textbox	Diagnostic ECG - Computer Assisted - Specify 1		Please specify computer assisted diagnosis for the diagnostic ED ECG.

PREDICT - Hospital Variables

Variable	Type	Caption	List Options	Abstraction Instruction
				Value = text<br><br>Source = Patient follow up information / ED chart
p_dgedecgdgsp_2	textbox	Diagnostic ECG - Computer Assisted - Specify 2 (cont)		
p_dgedecgdgsp_3	textbox	Diagnostic ECG - Computer Assisted - Specify 3 (cont)		
p_dgedecgdg	textbox	Diagnostic ECG Diagnosis 1		Indicate the diagnosis based on patient diagnostic ED ECG made by ED attending physician.<br><br>Source = Patient follow up information / ED chart
p_dgedecgdg_2	textbox	Diagnostic ECG Diagnosis 2 (cont)		
p_dgedecgdg_3	textbox	Diagnostic ECG Diagnosis 3 (cont)		
p_stedgedecg	dropdown	ST Elevation	listid: nyn<br>0. no<br>1. yes<br>2. not noted	Is there ST elevation on patient diagnostic ED ECG?<br><br>0=no<br>1=yes<br><br>Source = Patient follow up information / ED chart
p_stemicrdgedecg	dropdown	STEMI Criteria Met	listid: nynr<br><br>0. no<br>1. yes<br>2. not recorded	Is ST Elevation Myocardial Infarction (STEMI) criteria met on patient diagnostic ED ECG?<br><br>0=no<br><br>1=yes<br><br>2=not noted<br><br>Source = Patient follow up information / ED chart
p_stemildgedecg	textbox	STEMI Location		Indicate, if possible the STEMI location based on patient diagnostic ECG in ED.<br><br>Source = Patient follow up information / ED chart
p_pfedecg	div	Posttreatment ED ECG (60-90 [illegible]		
p_ptedecgxst	dropdown	Does Posttreatment ED ECG Recording Exist?	listid: ny<br><br>0. no	Did ED personnel record the patient posttretment ECG 60-90 minutes after reperfusion therapy received in ED?

PREDICT - Hospital Variables

Variable	Type	Caption	List Options	Abstraction Instruction
			1. yes	0=no<br>1=yes<br>Indicate no or yes, whether posttretment ED ECG recording exists. If there are more than one posttretment ED ECG recording enter the date for the last one.<br>Source = Patient follow up information / ED chart
p_pfedecgdt	textbox	Date		What was the date of the patient posttretment [illegible]<br>Date value = yyyy/mm/dd<br>Source = Patient follow up information / ED chart
p_pfedecgtm	textbox	Time		What was the time of the patient posttretment ECG (60-90 minutes) in ED?<br>Numerical value based on 24 hour clock<br>00:00:00 – hour:min:sec<br>If no value for seconds data available – do not do not use value 00<br>Source = Patient follow up information / ED chart
p_pfedecgrev	dropdown	Posttreatment ECG Reviewed	listid: nyu<br>0. no<br>1. yes<br>2. unknown	Indicate whether or not the posttretment ED ECG (60-90 minutes) was reviewed by the attending ED physician site or if the attendant annotations and QA data was generated solely by the device software.<br>0=no<br>1=yes<br>2=unknown<br>The goal is for sites to review the [illegible]<br>Source = Patient follow up information / ED chart
p_pfedecgd	dropdown	Posttreatment ECG Data Exists	listid: ny<br>0. no<br>1. yes	Is there the posttretment ED ECG (60-90 minutes) data available on request?<br>0=no

Variable	Type	Caption	List Options	Abstraction Instruction
				1=yes<br><br>Source = Patient follow up information / ED chart
p_pfedecgdgca	dropdown	Posttreatment ECG Diagnosis - Computer Assisted	listid: ny<br><br>0. no<br>1. yes	Indicate whether or not there was computer assisted diagnosis for the posttretment ED ECG ECG (60-90 minutes).<br><br>0=no<br>1=yes<br><br>Source = Patient follow up information / ED chart
p_pfedecgdgsp	textbox	Posttreatment ECG Diagnosis - Computer Assisted - Specify 1		Please specify computer assisted diagnosis for the posttretment ED ECG (60-90 minutes).<br><br>Value = text<br><br>Source = Patient follow up information / ED chart
p_pfedecgdgsp_2	textbox	Posttreatment ECG Diagnosis - Computer Assisted - Specify 2 (cont)		
p_pfedecgdgsp_3	textbox	Posttreatment ECG Diagnosis - Computer Assisted - Specify 3 (cont)		
p_pfedecgdg	textbox	Posttreatment ECG Diagnosis 1		Indicate the diagnosis based on patient posttretment ED ECG (60-90 minutes) made by ED attending physician.<br><br>Source = Patient follow up information / ED chart
p_pfedecgdg_2	textbox	Posttreatment ECG Diagnosis 2 (cont)		
p_pfedecgdg_3	textbox	Posttreatment ECG Diagnosis 3 (cont)		
p_stepfedecg	dropdown	ST Elevation	listid: nyn<br><br>0. no<br>1. yes<br>2. not noted	Is there ST elevation on patient posttretment ED ECG (60-90 minutes)?<br><br>0=no<br>1=yes<br><br>Source = Patient follow up information / ED chart
p_stemicrpfedecg	dropdown	STEMI Criteria Met	listid: nynr<br><br>0. no<br>1. yes<br>2. not recorded	Is ST Elevation Myocardial Infarction (STEMI) criteria met on patient posttretment ED ECG (60-90 minutes)?<br><br>0=no

Variable	Type	Caption	List Options	Abstraction Instruction
				1=yes<br>2=not noted<br>Source = Patient follow up information / ED chart
p_stemilpfedecg	textbox	STEMI Location		Indicate, if possible the STEMI location based on patient posttretment ED ECG (60-90 minutes) in ED.<br>Source = Patient follow up information / ED chart
p_pfredcp	dropdown	Reduction in the chest pain intensity	listid: nyn<br>0. no<br>1. yes<br>2. not noted	Indicate, if possible, if there is a significant reduction in the patient's chest pain intensity during the posttretment period ED ECG (60-90 minutes).<br>0=no<br>1=yes<br>2=not noted<br>Source = Patient follow up information / ED chart
p_pfredstemi	dropdown	Reduction in ST-segment elevation (i.e. > 50%)	listid: nyn<br>0. no<br>1. yes<br>2. not noted	Indicate, if possible, if there is a significant reduction in ST-segment elevation (i.e. > 50%) during the posttretment period ED ECG (60-90 minutes).<br>0=no<br>1=yes<br>2=not noted<br>Source = Patient follow up information / ED chart
p_edi_div	div	ED Interventions		
p_edi	dropdown	ED Interventions	listid: ny<br>0. no<br>1. yes	Select “Yes” if the ED personnel administered any of the following drugs.<br>0=no<br>1=yes<br>Source = Patient follow up information / ED chart
p_drxtn	div	Drug Therapy Noted		
p_edflytic	dropdown	Fibrinolytic Given	listid: nynr	Did patient receive fibrinolytic therepy during the ED course of care? This may be recorded as TNK, tPA (TPA or alteplase), RPA or thrombolytic.

PREDICT - Hospital Variables

Variable	Type	Caption	List Options	Abstraction Instruction
			0. no<br>1. yes<br>2. not recorded	0=no<br>1=yes<br>2=not noted<br>Source = Patient follow up information / ED chart
p_edflytic inel	dropdown	Fibrinolytic Ineligible	listid: ny<br>0. no<br>1. yes	Was patient ineligible to receive fibrinolytic therepy during the ED course of care?<br>0=no<br>1=yes<br>2=not noted<br>Source = Patient follow up information / ED chart
p_edflytic inelsp	textbox	Specify Reason		Please specify the reason why patient was deemed ineligible to receive fibrinolytic therepy during the ED course of care.<br>Source = Patient follow up information / ED chart
p_edflytic startdt	textbox	Fibrinolysis Start Date		What was the fibrinolysis start date during the ED course of patient care?<br>Date value = yyyy/mm/dd<br>Source = Patient follow up information / ED chart
p_edflytic starttm	textbox	Fibrinolysis Start Time		What was the fibrinolysis start time during the ED course of patient care?<br>Numerical value based on 24 hour clock<br>00:00:00 – hour:min:sec<br>If no value for seconds data available – do not do not use value 00.

Variable	Type	Caption	List Options	Abstraction Instruction
				Source = Patient follow up information / ED chart
p_edflyticdrug	dropdown	Fibrinolytic Drug Given	listid: drug2<br><br>1. TNK<br>2. tPA (TPA)<br>3. Activase (Alteplase)<br>4. RPA<br>5. Other	Which fibrinolytic drug did patient receive during the course of prehospital patient care?<br><br>1=TNK (Tenecteplase, Recombinant TPA) no code<br>2=TPA (Tissue Plasminogen Activator) Code 661<br>3=Activase (Alteplase)<br>4=RPA (Reteplase, Retavase) Code 646<br>5=other<br><br>Source = Patient follow up information / ED chart
p_edflyticdrugsp	textbox	Specify Other		Please specify which other fibrinolytic drug patient received during the ED course of care.<br><br>Source = Patient follow up information / ED chart
p_edflytictotd	textbox	Total Dose Given (mg)		Indicate the total in mg of TNK administered to patient throughout the ED course of patient care.<br><br>Source = Patient follow up information / ED chart<br><br>TNK Code<br><br>TNK dose is usually given with IV administration of 30 mg for patients's weight <60 kg; 35 mg for > 60 to < 70 kg; 40 mg for > 70 to < 80 kg; 45 mg for > 80 to < 90 kg; and 50 mg for > 90 kg.
p_edflytictotd2	textbox	Total Dose Given (mg)		Indicate the total in mg of tPA administered to patient throughout the ED course of patient care.<br><br>Source = Patient follow up information / ED chart<br>.
p_edflytictotd3	textbox	Total Dose Given (mg)		Indicate the total in mg of Activase (Alteplase) administered to patient throughout the ED course of patient care.<br><br>Source = Patient follow up information / ED chart

PREDICT - Hospital Variables

Variable	Type	Caption	List Options	Abstraction Instruction
				Recommended total Activase (Alteplase) dose is based on patient's weight and for AMI the total dose should not exceed 100 mg.<br><br>Activase totsl dose is usually given with IV administration of accelerated infusion (1.5 hours): 15 mg IV bolus for all patients; then: 1) for patients >67 kg 50 mg infused over the next 30 minutes and then 35 mg over the next 60 minutes; 2) for patients ≤67 kg infusion of 0.75 mg/kg over next 30 minutes (not to exceed 50 mg); and then 0.5 mg/kg over 60 minutes (not to exceed 35 mg).<br><br>Activase could also be administered over the 3 hour IV infusion of the total dose of 100 mg: 60 mg in the first hour (bolus dose of 6-10 mg); 20 mg over the second hour and 20 mg over the third hour. For smaller patients (≤65 kg) a dose of 1.25 mg/kg will be administered over the period of 3 hours.
p_edflytic totd4	textbox	Total Dose Given (Units)		Indicate the total in units of RPA administered to patient throughout the ED course of patient care.<br><br>Source = Patient follow up information / ED chart<br><br>RPA dose is usually given with IV administration of 10 U bolus over 2 mintes; 30 minutes later give second 10 U IV bolus over 2 minutes (give NS flush before and after each bolus); give heparin and aspirin conjuctively.
p_edflytic totd5	textbox	Total Dose Given		Indicate the total of other fibrinolytic drug patient received throughout the ED course of patient care.<br><br>Source = Patient follow up information / ED chart
p_edflytic inter	dropdown	Fibrinolysis Interrupted	listid: ny<br><br>0. no<br>1. yes	Was the fibrinolysis administration interrupted during the ED course of patient care?<br><br>0=no<br>1=yes<br><br>Source = Patient follow up information / ED chart
p_edflytic inrep	dropdown	Fibrinolysis Repeated	listid: ny<br><br>0. no<br>1. yes	Was the fibrinolysis adminstration repeated during the ED course of patient care?<br><br>0=no

Variable	Type	Caption	List Options	Abstraction Instruction
				1=yes<br><br>Source = Patient follow up information / ED chart
p_edflyticenddt	textbox	Fibrinolysis End Date		What was the fibrinolysis end date during the ED course of patient care?<br><br>Date value = yyyy/mm/dd<br><br>Source = Patient follow up information / ED chart
p_edflyticendtm	textbox	Fibrinolysis End Time		What was the fibrinolysis end time during the ED course of patient care?<br><br>Numerical value based on 24 hour clock<br><br>00:00:00 – hour:min:sec<br><br>If no value for seconds data available – do not do not use value 00.<br><br>Source = Patient follow up information / ED chart
p_edunfhep	dropdown	Unfractionated Heparin Given	listid: nynr<br><br>0. no<br>1. yes<br>2. not recorded	Did patient recive IV Unfractionated Heparin during the ED course of care.<br><br>0=no<br><br>1=yes<br><br>2=not noted<br><br>Source = Patient follow up information / ED chart<br><br>Indicate if IV Unfractionated Heparin was administered at any time during the ED course of care.
p_edunfhepibdt	textbox	Initial Bolus Date		The date when administration of IV Unfractionated Heparin initial bolus dose is confirmed during the ED course of care.<br><br>Source = Patient follow up information / ED chart
p_edunfhepibtm	textbox	Initial Bolus Time		The earliest time noted when administration of IV Unfractionated Heparin initial bolus dose is confirmed during the ED course of care.

PREDICT - Hospital Variables

Variable	Type	Caption	List Options	Abstraction Instruction
				Source = Patient follow up information / ED chart
p_edunhepibdu	textbox	Initial Bolus Dose (Units)		The total in units of IV Unfractionated Heparin initial bolus dose administered to patient throughout the ED course of patient care.<br><br>Source = Patient follow up information / ED chart<br><br>Unfractionated Heparin initial bolus dose is usually started with IV administration of 60 U/kg to maximum of 4000 U in conjuction with thrombolytics or 80 U/kg to maximum of 5000 U when no trombolytics were administered.
p_edunfhepidt	textbox	Infusion Date		The date when administration of IV Unfractionated Heparin infusion is confirmed during the ED course of patient care.<br><br>Source = Patient follow up information / ED chart
p_edunfhepitm	textbox	Infusion Time		The earliest time noted when administration of IV Unfractionated Heparin infusion is confirmed during the ED course of patient care.<br><br>Source = Patient follow up information / ED chart
p_edunhepidu	textbox	Infusion Dose (Units)		The total in units of IV Unfractionated Heparin infusion dose administered to patient throughout the ED course of patient care.<br><br>Source = Patient follow up information / ED chart<br><br>Unfractionated Heparin initial bolus dose is usually started with IV administration of 12 U/kg/hr to maximum of 1000 U/hr in conjuction with fibrinolytics or 18 U/kg/hr to maximum of 1500 U/hr when no fibrinolytics were administered.

PREDICT - Hospital Variables

Variable	Type	Caption	List Options	Abstraction Instruction
				Dose is adjusted to maintain PTT at 1.5 – 2 times control value.
p_edenox	dropdown	Enoxaparin Given	listid: nynr<br><br>0. no<br>1. yes<br>2. not recorded	Did patient recive IV Enoxaparin during the ED course of patient care?<br><br>0=no<br><br>1=yes<br><br>2=not noted<br><br>Source = Patient follow up information / ED chart<br><br>Indicate if IV Unfractionated Heparin was administered at any time during during the ED course of patient care.
p_edenoini	dropdown	Inital bolus dose given	listid: ny<br>0. no<br>1. yes	
p_edenoxdt	textbox	Initial Bolus Date		The date when administration of IV Enoxaparin initial bolus dose is confirmed during the ED course of patient care.<br><br>Source = Patient follow up information / ED chart
p_edpenoxtm	textbox	Initial Bolus Time		The earliest time noted when administration of IV Enoxaparin initial bolus dose is confirmed during the ED course of patient care.<br><br>Source = Patient follow up information / ED chart
p_edenoxibdmg	textbox	Initial Bolus Dose (mg)		The total in mg of IV Enoxaparin initial bolus dose administered to patient throughout the ED course of patient care.<br><br>Source = Patient follow up information / ED chart

PREDICT - Hospital Variables

Variable	Type	Caption	List Options	Abstraction Instruction
				Enoxaparin initial bolus dose is usually started with IV administration of 30 mg for patients < 75 years of age. Patients ≥ 75 years of age do not receive Enoxaparin IV bolus.
p_edsceno	dropdown	Subcutoneous (sc) Enoxaparin	listid: ny<br>0. no<br>1. yes	
p_edenoxidt	textbox	SC injection date		The date when administration of subcutaneous (SC) Enoxaparin infusion is confirmed during the ED course of patient care.<br><br>Source = Patient follow up information / ED chart
p_edenoxitm	textbox	SC injection time		The earliest time noted when administration of SC Enoxaparin infusion is confirmed during the ED course of patient care.<br><br>Source = Patient follow up information / ED chart
p_edenoxidmg	textbox	SC injection dose (mg)		The total in mg of SC Enoxaparin infusion dose administered to patient throughout the ED course of patient care.<br><br>Source = Patient follow up information / ED chart<br><br>Enoxaparin infusion dose is usually started with SC administration of 1 mg/kg (maximum 100 mg) for patients < 75 years of age and 0.75 mg/kg (maximum 75 mg) for patients ≥ 75 years of age.
p_edlmwh	dropdown	Other LMWH product given	listid: nynr<br>0. no<br>1. yes<br>2. not recorded	
p_edlmwhso	textbox	Specify Other		
p_edlmwhft	textbox	First given time		
p_edlmwhdose	textbox	Total dose (mg)		
p_edclop	dropdown	Clopidogrel (Plavix)	listid: nynr<br>0. no<br>1. yes<br>2. not recorded	Did patient recive Clopidogrel during the ED course of patient care?<br><br>0=no

PREDICT - Hospital Variables

Variable	Type	Caption	List Options	Abstraction Instruction
				1=yes<br><br>2=not noted<br><br>Source = Patient follow up information / ED chart<br><br>Indicate if Clopidogrel was administered at any time during during the ED course of patient care.
p_edcloptm	textbox	First Given Time		The earliest time noted when administration of Clopidogrel is confirmed during the ED course of patient care.<br><br>Source = Patient follow up information / ED chart
p_edclopto td	textbox	Total Dose Given (mg)		Indicate the total in mg of Clopidogrel administered to patient throughout the ED course of care.<br><br>Source = Patient follow up information / ED chart<br><br>Clopidogrel dose is usually given with a loading dose of 300 mg for 75 years and under, and 75 mg for 76 years and older. The decision to administer additional clopidogrel to patients is usually left to the discretion of the interventional cardiologist.
p_edglycin h	dropdown	Glycoprotein llb/llla Inhibitors	listid: nynr<br><br>0. no<br>1. yes<br>2. not recorded	Did patient recive IV glycoprotein IIb/IIIa inhibitors during the ED course of patient care?<br><br>0=no<br><br>1=yes<br><br>2=not noted

PREDICT - Hospital Variables

Variable	Type	Caption	List Options	Abstraction Instruction
				Source = Patient follow up information / ED chart<br><br>Indicate if IV glycoprotein IIb/IIIa inhibitors were administered at any time during during the ED course of patient care.
p_edglycinhtype	dropdown	Glycoprotein llb/llla Inhibitors Type	listid: glycinh2<br><br>1. Abciximab (ReoPro)<br>2. Eptifibatide (Intergrilin)<br>3. Tirofiban (Aggrastat)<br>4. Other	What type of glycoprotein IIb/IIa inhibitor did patient recive during the ED course of patient care?<br><br>1=Abeiximab (ReoPro)<br>2=Eptifibatide (Intergrilin)<br>3=Tirofiban (Aggrastat)<br>4= other (specify)<br><br>Source = Patient follow up information / ED chart<br><br>Indicate what type of glycoprotein IIb/IIIa inhibitor was administered at any time during during the ED course of patient care.
p_edglycinhtypesp	textbox	Specify Other		Please specify which other glycoprotein IIb/IIa inhibitor patient received during the ED course of patient care.<br><br>Source = Patient follow up information / ED chart
p_edglycinhtm	textbox	First Given Time		The earliest time noted when administration of glycoprotein IIb/IIa inhibitor is confirmed during the ED course of patient care.<br><br>Source = Patient follow up information / ED chart
p_edglycinhtotd	textbox	Total Dose Given (mg)		Indicate the total in mg of Abciximab administered to patient throughout the ED course of care.<br><br>Source = Patient follow up information / ED chart<br><br>Abciximab dose is usually given with IV bolus administration of 0.25 mg/kg 10-60 minutes before the start of PCI, followed by a continuous intravenous infusion of 0.125 Âµg/kg/min (to a maximum of 10 Âµg/min) for 12 hours.
p_edglycinhtotd2	textbox	Total Dose Given (mg)		Indicate the total in mg of Eptifibatide administered to patient throughout the ED course of care.

PREDICT - Hospital Variables

Variable	Type	Caption	List Options	Abstraction Instruction
				Source = Patient follow up information / ED chart<br><br>Eptifibatide dose is usually given with IV bolus administration of 180 Âµg/kg as soon as possible followed by a continuous infusion of 2.0 Âµg/kg/min until hospital discharge or initiation of CABG surgery, up to 72 hours. If a patient is to undergo a percutaneous coronary intervention (PCI) while receiving eptifibatide, the infusion should be continued up to hospital discharge, or for up to 18 to 24 hours after the procedure, whichever comes first, allowing for up to 96 hours of therapy .
p_edglycinhtotd3	textbox	Total Dose Given (mg)		Indicate the total in mg of Tirofiban administered to patient throughout the ED course of care.<br><br>Source = Patient follow up information / ED chart<br><br>Tirofiban dose is usually given with IV at an initial rate of 0.4 Âµg/kg/min for 30 minutes and then continued at 0.1 Âµg/kg/min.
p_edflycinhtotd4	textbox	Total Dose Given (mg)		Indicate the total in mg of the other of glycoprotein IIb/IIa inhibitor administered to patient throughout the ED course of care.<br><br>Source = Patient follow up information / ED chart
p_edtrombinh	dropdown	Thrombin Inhibitors	listid: nynr<br>0. no<br>1. yes<br>2. not recorded	Did patient recive thrombin inhibitors during the ED course of patient care?<br><br>0=no<br>1=yes<br>2=not noted<br><br>Source = Patient follow up information / ED chart<br><br>Indicate if trombin inhibitors were administered at any time during during the ED course of patient care.

PREDICT - Hospital Variables

Variable	Type	Caption	List Options	Abstraction Instruction
p_edtrombinhtype	dropdown	Thrombin Inhibitors Type	listid: trombinh<br><br>1. Bivalirudin<br>2. Lepirudin<br>3. Desirudin<br>4. Other	What type of thrombin inhibitor did patient recive during the ED course of patient care?<br><br>1=Bivalirudin (Angiomax)<br>2=Lepirudin (Refludan)<br>3=Desirudin<br>4=Other<br><br>Source = Patient follow up information / ED chart<br><br>Indicate what type of trombin inhibitor administered at any time during during the ED course of patient care.
p_edthrombinhtypesp	textbox	Specify Other		Please specify which other thrombin inhibitor patient received during the ED course of patient care.<br><br>Source = Patient follow up information / ED chart
p_edtrombinhtm	textbox	First Given Time		The earliest time noted when administration of trombin inhibitor is confirmed during the ED course of patient care.<br><br>Source = Patient follow up information / ED chart
p_edtrombinhtotd	textbox	Total Dose Given (mg)		Indicate the total in mg of Bivalirudin (Angiomax) administered to patient throughout the ED course of care.<br><br>Source = Patient follow up information / ED chart<br><br>Bivalrudin total dose is usually given with IV loading dose (fast injection) of 0.75 mg/kg. Additional bolus dose of 0.3 mg/kg might be administered if necessary. This is followed by IV infusion of 1.75 mg/kg/hour for the duration of PCI. After 4 hours additional IV infusion of 0.2 mg/kh/hour for up to 20 hours might be administered.
p_edtrombinhtotd2	textbox	Total Dose Given (mg)		Indicate the total dose in mg of Lepirudin (Refludan) administered to patient throughout the ED course of care.<br><br>Source = Patient follow up information / ED chart

Variable	Type	Caption	List Options	Abstraction Instruction
				Lepirudin total dose is usually given with IV loading dose (slowly) of 0.4 mg/kg (up to 110 kg) followed by IV infusion of 0.15 mg/kg/hour for 2-10 days or londer if clinically needed.
p_edtrombinhtotd3	textbox	Total Dose Given (mg)		Indicate the total dose in mg of Desirudin administered to patient throughout the ED course of care.<br><br>Source = Patient follow up information / ED chart
p_edtrombinhtotd4	textbox	Total Dose Given (mg)		Indicate the total dose in mg of other trombin inhibitor administered to patient throughout the ED course of care.<br><br>Source = Patient follow up information / ED chart
p_edasa2	dropdown	ASA (Aspirin) Given	listid: nynr<br><br>0. no<br>1. yes<br>2. not recorded	Indicate if ASA was administered at any time during the ED course of patient care.<br><br>0=no<br><br>1=yes<br><br>2=not noted<br><br>Source = Patient follow up information / ED chart
p_edasaftm	textbox	First Given Time		The earliest time noted when administration of ASA is confirmed during the ED course of patient care.<br><br>Source = Patient follow up information / ED chart
p_edasamg	textbox	Total Dose (mg)		The total in mg of all doses of ASA administered to patient throughout the ED course of care.<br><br>Â· Usual dose of ASA is 160 mg (2x80 mg) for the patient to chew and swallow. This dose may be administered regardless of any previous ASA self-administration.<br><br>Source = Patient follow up information / ED chart
p_ednitro2	dropdown	Nitroligual Spray Given	listid: nynr<br><br>0. no<br>1. yes	Indicate if Nitrolingual spray was administered at any time during the ED course of patient care.

PREDICT - Hospital Variables

Variable	Type	Caption	List Options	Abstraction Instruction
			2. not recorded	0=no<br>1=yes<br>2=not noted<br>Source = Patient follow up information / ED chart
p_ednitro2ftm	textbox	First Given Time		
p_ednitro2mg	textbox	Total Dose (mg)		
p_ednitropatch	dropdown	Nitro Patch	listid: ny<br>0. no<br>1. yes	
p_ednitroftm	textbox	First Given Time		The earliest time noted when administration of Nitrolingual spray 0.4 mg/spray is confirmed during the ED course of patient care.<br>Source = Patient follow up information / ED chart
p_ednitromg	textbox	Total Dose (mg)		The total in mg of all doses of Nitrolingual spray administered to patient throughout the ED course of care.<br>Â· Maximal number of Nitrolingual spray 0.4 mg/spray is 3; single spray is administered every 5 minutes.<br>Source = Patient follow up information / ED chart
p_ednitrodrip	dropdown	Nitro IV Drip Given	listid: nynr<br>0. no<br>1. yes<br>2. not recorded	Indicate if Nitro IV drip was administered at any time during the ED course of patient care.<br>0=no<br>1=yes<br>2=not noted<br>Source = Patient follow up information / ED chart
p_ednitrodripftm	textbox	First Given Time		The earliest time noted when administration of Nitro IV drip during the ED course of patient care.

PREDICT - Hospital Variables

Variable	Type	Caption	List Options	Abstraction Instruction
				Source = Patient follow up information / ED chart
p_ednitromgmin	textbox	Total Dose (mg)		The total in mg of Nitro IV drip administered throughout the ED course of patient care.<br><br>Â· Nitro drip is usually started with IV administration of 0.1 Âµg/kg/min and then tittred to up to 200 Âµg/min.<br><br>Source = Patient follow up information / ED chart
p_edmor2	dropdown	Morphine Given	listid: nynr<br><br>0. no<br>1. yes<br>2. not recorded	Indicate if IV Morphine Sulphate (2 mg) was administered at any time during the ED course of patient care. .<br><br>0=no<br>1=yes<br>2=not noted<br><br>Source = Patient follow up information / ED chart
p_edmorftm	textbox	First Given Time		The earliest time noted when administration of IV Morphine Sulphate is confirmed during the ED course of patient care.<br><br>Source = Patient follow up information / ED chart
p_edmormg	textbox	Total Dose (mg)		The total in mg of IV Morphine Sulphate (2 mg) administered throughout the ED course of patient care.<br><br>Â· Maximum of 3 doses (single dose 2 mg; total of 6 mg for 3 doses) of IV Morphine Sulphate may be administered; single dose is administered every 5 minutes.<br><br>Source = Patient follow up information / ED chart
p_edepi2	dropdown	Epinephrine Given	listid: nynr<br><br>0. no<br>1. yes	Indicate if epinephrine was administered at any time during the ED course of patient care.

Variable	Type	Caption	List Options	Abstraction Instruction
			2. not recorded	0=no<br>1=yes<br>2=not noted<br>Source = Patient follow up information / ED chart
p_edepftm	textbox	First Given Time		The earliest time noted when administration of Epinephrine is confirmed during the ED course of patient care.<br>Source = Patient follow up information / ED chart
p_edepimg	textbox	Total Dose (mg)		The total of all doses of Epinephrine administered at any time during the ED course of patient care.<br>Source = Patient follow up information / ED chart<br>NOTE: Epinephrine is supplied in two volumes 1:10,000 and 1:1,000. The units of measurement can me mg, ml, amp or units. Please use the following conversions to determine the total volume of epinephrine given in mg:<br>Â· 1:10, 000 reported in mg - no conversion<br>Â· 1:10,000 reported in ml - # ml x 0.1 mg = # mg<br>Â· 1:10,000 reported in amp - # amp x 1.0 mg = # mg<br>Â· 1:1,000 reported in mg – no conversion<br>Â· 1:1,000 reported in ml - # ml x 1.0 mg = # mg<br>Â· 1:1,000 reported in units - # units x 0.1 mg = # mg<br>Â· 1:1,000 reported in amp - # amp x 1 mg = # mg
p_edepiiv	dropdown	IV	listid: nynr<br>0. no<br>1. yes<br>2. not recorded	Did patient receive Epinephrine via IV during the ED course of care?<br>0=no<br>1=yes<br>2=not noted

PREDICT - Hospital Variables

Variable	Type	Caption	List Options	Abstraction Instruction
				Source = Patient follow up information / ED chart
p_edepiet	dropdown	ET/ETT	listid: nynr<br><br>0. no<br>1. yes<br>2. not recorded	Did patient receive Epinephrine via ETT (“down the tube”) during the ED course of care?<br><br>0=no<br><br>1=yes<br><br>2=not noted<br><br>Source = Patient follow up information / ED chart
p_edepiio	dropdown	IO	listid: nynr<br><br>0. no<br>1. yes<br>2. not recorded	Did patient receive Epinephrine via IO (Intraosseous) during the ED course of care?<br><br>0=no<br><br>1=yes<br><br>2=not noted<br><br>Source = Patient follow up information / ED chart<br><br>Route indicated for any Epinephrine given is IO or intraosseous.
p_edamio2	dropdown	Amiodarone Given	listid: nynr<br><br>0. no<br>1. yes<br>2. not recorded	Did patient receive Amiodarone during the ED course of care?<br><br>0=no<br><br>1=yes<br><br>2=not noted

Variable	Type	Caption	List Options	Abstraction Instruction
				Source = Patient follow up information / ED chart<br><br>Indicate if amiodarone was administered at any time during the ED course of patient care.
p_edamftm	textbox	First Given Time		The earliest time noted when administration of Amiodarone is confirmed during the ED course of patient care.<br><br>Source = Patient follow up information / ED chart
p_edamimg	textbox	Total Dose (mg)		The total of all doses of Amiodarone administered at any time during the ED course of patient care.<br><br>Source = Patient follow up information / ED chart<br><br>NOTE: Amiodarone is supplied in two volumes 1:10,000 and 1:1,000. The units of measurement can me mg, ml, amp or units. Please use the following conversions to determine the total volume of amiodarone given in mg:<br><br>Â· 1:10, 000 reported in mg - no conversion<br>Â· 1:10,000 reported in ml - # ml x 0.1 mg = # mg<br>Â· 1:10,000 reported in amp - # amp x 1.0 mg = # mg<br>Â· 1:1,000 reported in mg – no conversion<br>Â· 1:1,000 reported in ml - # ml x 1.0 mg = # mg<br>Â· 1:1,000 reported in units - # units x 0.1 mg = # mg<br>Â· 1:1,000 reported in amp - # amp x 1 mg = # mg
p_edatro2	dropdown	Atropine Given	listid: nynr<br>0. no<br>1. yes<br>2. not recorded	Did patient receive Atropine during the ED course of patient care?<br><br>0=no<br><br>1=yes<br><br>2=not noted

PREDICT - Hospital Variables

Variable	Type	Caption	List Options	Abstraction Instruction
				Source = Patient follow up information / ED chart<br><br>Indicate if atropine was administered at any time during the ED course of patient care.
p_edatftm	textbox	First Given Time		The earliest time noted when administration of Atropine is confirmed during the ED course of patient care.<br><br>Source = Patient follow up information / ED chart
p_edatrmg	textbox	Total Dose (mg)		The total of all doses of Atropine administered at any time during the ED course of patient care.<br><br>Source = Patient follow up information / ED chart<br><br>NOTE: Atropine is supplied in two volumes 1:10,000 and 1:1,000. The units of measurement can me mg, ml, amp or units. Please use the following conversions to determine the total volume of atropine given in mg:<br><br>Â· 1:10, 000 reported in mg - no conversion<br>Â· 1:10,000 reported in ml - # ml x 0.1 mg = # mg<br>Â· 1:10,000 reported in amp - # amp x 1.0 mg = # mg<br>Â· 1:1,000 reported in mg – no conversion<br>Â· 1:1,000 reported in ml - # ml x 1.0 mg = # mg<br>Â· 1:1,000 reported in units - # units x 0.1 mg = # mg<br>Â· 1:1,000 reported in amp - # amp x 1 mg = # mg
p_edlido2	dropdown	Lidocaine	listid: nynr<br>0. no<br>1. yes<br>2. not recorded	Did patient receive Lidocaine during the ED course of care?<br><br>0=no<br><br>1=yes<br><br>2=not noted

Variable	Type	Caption	List Options	Abstraction Instruction
				Source = Patient follow up information / ED chart<br><br>Indicate if lidocaine was administered at any time during the ED course of patient care.
p_edliftm	textbox	First Given Time		The earliest time noted when administration of Lidocaine is confirmed during the ED course of patient care.<br><br>Source = Patient follow up information / ED chart
p_edlidmg	textbox	Total Dose (mg)		The total of all doses of Lidocaine administered at any time during the ED course of patient care.<br><br>Source = Patient follow up information / ED chart<br><br>NOTE: Lidocaine is supplied in two volumes 1:10,000 and 1:1,000. The units of measurement can me mg, ml, amp or units. Please use the following conversions to determine the total volume of lidocaine given in mg:<br><br>Â· 1:10, 000 reported in mg - no conversion<br>Â· 1:10,000 reported in ml - # ml x 0.1 mg = # mg<br>Â· 1:10,000 reported in amp - # amp x 1.0 mg = # mg<br>Â· 1:1,000 reported in mg – no conversion<br>Â· 1:1,000 reported in ml - # ml x 1.0 mg = # mg<br>Â· 1:1,000 reported in units - # units x 0.1 mg = # mg<br>Â· 1:1,000 reported in amp - # amp x 1 mg = # mg
p_eddopa2	dropdown	Dopamine	listid: nynr<br><br>0. no<br>1. yes<br>2. not recorded	Did patient receive Dopamine during the ED course of care?<br><br>0=no<br><br>1=yes<br><br>2=not noted

Variable	Type	Caption	List Options	Abstraction Instruction
				Source = Patient follow up information / ED chart<br><br>Indicate if Dopamine was administered at any time during the ED course of patient care.
p_eddoftm	textbox	First Given Time		The earliest time noted when administration of IV Dopamine is confirmed during the ED course of patient care.<br><br>Source = Patient follow up information / ED chart
p_eddopmg	textbox	Total Dose (mg)		The total in mg of all doses of IV Dopamine administered throughout the ED course of patient care.<br><br>Source = Patient follow up information / ED chart<br><br>Dopamine is usually started with IV administration of 5 Âµg/kg and then tittred to up to 20 Âµg/kg.
p_edcaren	div	ED Lab and Cardiac Enzymes		
p_edlabtest	dropdown	Were Lab Tests Done in ED?	listid: ny<br><br>0. no<br>1. yes	Were lab tests done during the ED course of patient care?<br><br>0=no<br>1=yes<br><br>Source = Patient follow up information / ED chart<br><br>Indicate if lab tests were done at any time during the ED course of patient care.
p_edlabtestdt	textbox	Lab Tests Date		The date when the lab tests were done first time during the ED course of patient care.<br><br>Source = Patient follow up information / ED chart
p_edlabtesttm	textbox	Lab Tests Time		The time when the lab tests were done first time during the ED course of patient care.<br><br>Source = Patient follow up information / ED chart
p_edchol	dropdown	Cholesterol	listid: ny<br><br>0. no<br>1. yes	Indicate if cholesterol was measured during the ED course of patient care?<br><br>0=no<br>1=yes

PREDICT - Hospital Variables

Variable	Type	Caption	List Options	Abstraction Instruction
				Source = Patient follow up information / ED chart
p_edchollev	textbox	Cholesterol Levels (mmol/L)		Indicate the first cholesterol levels measured during the ED course of patient care.<br><br>Source = Patient follow up information / ED chart
p_edhdl	dropdown	HDL	listid: ny<br>0. no<br>1. yes	Indicate if HDL was measured during the ED course of patient care?<br><br>0=no<br>1=yes<br><br>Source = Patient follow up information / ED chart
p_edhdllev	textbox	HDL Levels		Indicate the first HDL levels measured during the ED course of patient care.<br><br>Source = Patient follow up information / ED chart
p_edldl	dropdown	LDL	listid: ny<br>0. no<br>1. yes	Indicate if LDL was measured during the ED course of patient care?<br><br>0=no<br>1=yes<br><br>Source = Patient follow up information / ED chart
p_edldllev	textbox	LDL Levels		Indicate the first LDL levels measured during the ED course of patient care.<br><br>Source = Patient follow up information / ED chart
p_edtriglyc	dropdown	Triglycerides	listid: ny<br>0. no<br>1. yes	Indicate if tryglicerides were measured during the ED course of patient care?<br><br>0=no<br>1=yes<br><br>Source = Patient follow up information / ED chart
p_edtriglyclev	textbox	Triglycerides Levels (mmol/L)		Indicate the first tryglicerides levels measured during the ED course of patient care.<br><br>Source = Patient follow up information / ED chart
p_edgluc	dropdown	Glucose	listid: ny<br>0. no<br>1. yes	Indicate if glucose was measured during the ED course of patient care?<br><br>0=no<br>1=yes

Variable	Type	Caption	List Options	Abstraction Instruction
				Source = Patient follow up information / ED chart
p_edgluclev	textbox	Glucose Levels		Indicate the first glucose levels measured during the ED course of patient care.<br><br>Source = Patient follow up information / ED chart
p_edcreat	dropdown	Creatinine	listid: ny<br>0. no<br>1. yes	Indicate if creatinine was measured during the ED course of patient care?<br><br>0=no<br>1=yes<br><br>Source = Patient follow up information / ED chart
p_edcreatlev	textbox	Creatinine Levels		Indicate the first creatinine levels measured during the ED course of patient care.<br><br>Source = Patient follow up information / ED chart
p_edcarendn	dropdown	Were Cardiac Enzymes Done in ED?	listid: ny<br>0. no<br>1. yes	Were cardiac enzymes done during the ED course of patient care?<br><br>0=no<br>1=yes<br><br>Source = Patient follow up information / ED chart<br><br>Indicate if cardiac enzymes were done at any time during the ED course of patient care.
p_edfcarendt	textbox	First Cardiac Enzymes Measurement Date		The date when the cardiac enzymes were measured first time during the ED course of patient care.<br><br>Source = Patient follow up information / ED chart
p_edfcarentm	textbox	First Cardiac Enzymes measurement Time		The time when the cardiac enzymes were measured first time during the ED course of patient care.<br><br>Source = Patient follow up information / ED chart
p_edfck	dropdown	First Creatine Kinase (CK)	listid: ny<br>0. no<br>1. yes	Indicate if first CK levels were measured during the ED course of patient care?<br><br>0=no<br>1=yes

Variable	Type	Caption	List Options	Abstraction Instruction
				Source = Patient follow up information / ED chart
p_edfcklev	textbox	First CK Levels (U/L)		Indicate the first CK levels measured during the ED course of patient care.<br><br>Source = Patient follow up information / ED chart
p_edfckmb	dropdown	First Creatine Kinase - MB (CK-MB)	listid: ny<br><br>0. no<br>1. yes	Indicate if first CK-MB levels were measured during the ED course of patient care?<br><br>0=no<br>1=yes<br><br>Source = Patient follow up information / ED chart
p_edfckmblev	textbox	First CK-MB Levels (U/L)		Indicate the first CK-MB levels measured during the ED course of patient care.<br><br>Source = Patient follow up information / ED chart
p_edftropi	dropdown	First Troponin I	listid: ny<br><br>0. no<br>1. yes	Indicate if first Troponin I levels were measured during the ED course of patient care?<br><br>0=no<br>1=yes<br><br>Source = Patient follow up information / ED chart
p_edftropilev	textbox	First Troponin I Levels (Âµg/L)		Indicate the first Troponin I levels measured during the ED course of patient care.<br><br>Source = Patient follow up information / ED chart
p_edftropt	dropdown	First Troponin T	listid: ny<br><br>0. no<br>1. yes	Indicate if first Troponin T levels were measured during the ED course of patient care?<br><br>0=no<br>1=yes<br><br>Source = Patient follow up information / ED chart
p_edftroptlev	textbox	First Troponin T Levels (Âµg/L)		Indicate the first Troponin T levels measured during the ED course of patient care.<br><br>Source = Patient follow up information / ED chart

PREDICT - Hospital Variables

Variable	Type	Caption	List Options	Abstraction Instruction
p_edpcarendt	textbox	Peak Cardiac Enzymes Measurement Date		The date when the peak cardiac enzymes were measured during the ED course of patient care.<br><br>Source = Patient follow up information / ED chart
p_edpcarentm	textbox	Peak Cardiac Enzymes Measurement Time		The time when the peak cardiac enzymes were measured during the ED course of patient care.<br><br>Source = Patient follow up information / ED chart
p_edpck	dropdown	Peak Creatine Kinase (CK)	listid: ny<br><br>0. no<br>1. yes	Indicate if peak CK levels were measured during the ED course of patient care?<br><br>0=no<br>1=yes<br><br>Source = Patient follow up information / ED chart
p_edpcklev	textbox	Peak CK Levels (U/L)		Indicate the peak CK levels measured during the ED course of patient care.<br><br>Source = Patient follow up information / ED chart
p_edpckmb	dropdown	Peak Creatine Kinase - MB (CK-MB)	listid: ny<br><br>0. no<br>1. yes	Indicate if peak CK-MB levels were measured during the ED course of patient care?<br><br>0=no<br>1=yes<br><br>Source = Patient follow up information / ED chart
p_edpckmblev	textbox	Peak CK-MB Levels (U/L)		Indicate the peak CK-MB levels measured during the ED course of patient care.<br><br>Source = Patient follow up information / ED chart
p_edptropi	dropdown	Peak Troponin I	listid: ny<br><br>0. no<br>1. yes	Indicate if peak Troponin I levels were measured during the ED course of patient care?<br><br>0=no<br>1=yes<br><br>Source = Patient follow up information / ED chart
p_edptropilev	textbox	Peak Troponin I Levels (Âµg/L)		Indicate the peak Troponin I levels measured during the ED course of patient care.

PREDICT - Hospital Variables

Variable	Type	Caption	List Options	Abstraction Instruction
				Source = Patient follow up information / ED chart
p_edptropt	dropdown	Peak Troponin T	listid: ny<br><br>0. no<br>1. yes	Indicate if peak Troponin T levels were measured during the ED course of patient care?<br><br>0=no<br>1=yes<br><br>Source = Patient follow up information / ED chart
p_edptroptlev	textbox	Peak Troponin T Levels (Âµg/L)		Indicate the peak Troponin T levels measured during the ED course of patient care.<br><br>Source = Patient follow up information / ED chart
p_edd	div	ED Disposition		
p_edddt	textbox	ED Departure Date		What was the date of the patient ED departure?<br><br>Date value = yyyy/mm/dd<br><br>Source = Patient follow up information / ED chart/ Hospital chart
p_eddtm	textbox	ED Departure Time		What was the time of the patients ED departure?<br><br>00:00:00 – hour:min:sec<br><br>Source = Patient follow up information / ED chart/ Hospital chart
p_eddhome	dropdown	Was Patient Discharged Home?	listid: ny<br><br>0. no<br>1. yes	After admission to ED was patient then discharged home?<br><br>0=no<br><br>1=yes<br><br>Source = Patient follow up information / ED chart/ Hospital chart
p_edptshosp	dropdown	Was patient admitted to the same hospital?	listid: ny<br>0. no<br>1. yes	
p_edptxferach	dropdown	Was Patient Transferred to Another Acute Care Hospital?	listid: ny<br><br>0. no<br>1. yes	After initial admission to ED was patient then transferred to another hospital facility?

PREDICT - Hospital Variables

Variable	Type	Caption	List Options	Abstraction Instruction
				0=no<br>1=yes<br>Source = Patient follow up information / ED chart/ Hospital chart
p_edxhosp	dropdown	Transfer Hospital Name	listid: p_hosp<br>** See list items in appendix	What is the name of the hospital patient was transferred to?<br>Source = Patient follow up information / / ED chart/Hospital chart<br>Pulldown menu = list of hospitals in database<br>Unknown Hospital = select thwn destination hospital will never be known.<br>NOTE;<br>Do not provide the names of nursing home, rehabilitation, or other non-acute care facilities.<br>Transfer to one of these three entities constitutes an ''ED/hospital discharge, reclassification, or death''.
p_edptxferpci	dropdown	Was Patient Transferred to PCI Centre?	listid: ny<br>0. no<br>1. yes	After initial admission to ED was patient then transferred to PCI centre?<br>0=no<br>1=yes<br>Source = Patient follow up information / ED chart// Hospital chart
p_edxpci	dropdown	Transfer PCI Centre Name	listid: p_hosp<br>** See list items in appendix	What is the name of the hospital patient was transferred to?<br>Source = Patient follow up information / / ED chart/Hospital chart<br>Pulldown menu = list of hospitals in database<br>Unknown Hospital = select when destination hospital will never be known.<br>NOTE;

PREDICT - Hospital Variables

Variable	Type	Caption	List Options	Abstraction Instruction
				Do not provide the names of nursing home, rehabilitation, or other non-acute care facilities.<br>Transfer to one of these three entities constitutes an ''ED/hospital discharge, reclassification, or death''.
p_eddptrans	dropdown	Transport Mode	listid: trans<br>1. by land<br>2. by air	Where a patient has been transported by both land and air, select air.<br>Source = Patient follow up information / / ED chart/Hospital chart
p_edicd10	div	ICD 10 Codes		
p_edicd10_1	textbox	1		
p_edicd10_2	textbox	2		
p_edicd10_3	textbox	3		
p_edicd10_4	textbox	4		
p_edicd10_5	textbox	5		
p_edicd10_6	textbox	6		
p_edicd10_7	textbox	7		
p_edicd10_8	textbox	8		
p_edicd10_9	textbox	9		
p_edicd10_10	textbox	10		
p_edicd10_11	textbox	11		
p_edicd10_12	textbox	12		
p_edicd10_13	textbox	13		
p_edicd10_14	textbox	14		
p_edicd10_15	textbox	15		
p_edicd10_16	textbox	16		
p_edstatus	dropdown	ED Form Status	listid: p_status<br>0. Incomplete<br>1. Complete<br>2. Pending Source Doc(s)<br>3. Not Required - Patient Deceased	
**p_pciform**	**section**	**Percutaneous Coronary Intervention (PCI) Centre Form**		
p_pihpci	div	Patient Identifiers		

PREDICT - Hospital Variables

Variable	Type	Caption	List Options	Abstraction Instruction
p_ilnamepci	textbox	Surname		This field is used to obtain the PCI/Hospital chart version of the patient's surname.<br><br>Transcribe as-is.<br><br>Caps Lock
p_ifnamepci	textbox	Given Name		This field is used to obtain the PCI/Hospital chart version of a patient's given name.<br><br>Transcribe as-is.<br><br>Caps Lock
p_iaddrpci	textbox	Mailing Address		This field is used to obtain the PCI/Hospital chart version of a patient's mailing/street address.<br><br>Transcribe as-is.<br><br>Caps Lock
p_icitypci	textbox	City/Town		This field is used to obtain the PCI/Hospital chart version of a patient's city / town.<br><br>Transcribe as-is.<br><br>Caps Lock
p_iprovpci	dropdown	Province	listid: prov<br><br>1. AB<br>2. BC<br>3. MB<br>4. NB<br>5. NF<br>6. NS<br>7. NT<br>8. NU<br>9. ON<br>10. PE<br>11. QC<br>12. SK<br>13. YT<br>99. Out of Country	This field is used to obtain the PCI/Hospital chart version of a patient's province.<br><br>Transcribe as-is.<br><br>Caps Lock
p_ipostcpci	textbox	Postal Code		This field is used to obtain the PCI/Hospital chart version of a patient's postal code.<br><br>Transcribe as-is.<br><br>Caps Lock
p_idobpci	textbox	Date of Birth		This field is used to obtain the PCI/Hospital chart version of patients date of birth (MDY).

PREDICT - Hospital Variables

Variable	Type	Caption	List Options	Abstraction Instruction
				Transcribe as-is.<br><br>Caps Lock
p_wght_pci	dropdown	Weight	listid: uy<br>0. unknown/not noted<br><br>1. yes	ACR - Physical Exam – Weight<br><br>AACR - Administration – Weight<br><br>Transcribe as-is<br><br>In the case of multiple vehicles, use the ACR that was completed by the EMS crew that treated and transported the patient.<br><br>In some cases the medic might not be able to ascertain the weight of a patient, i.e., weight = unknown/not noted.
p_wghtkg_pci	textbox	Weight (kg)		ACR - Physical Exam – Weight<br><br>AACR - Administration – Weight<br><br>Transcribe as-is<br><br>In the case of multiple vehicles, use the ACR that was completed by the EMS crew that treated and transported the patient.<br><br>In some cases the medic might not be able to ascertain the weight of a patient, i.e., gender="Unknown".
p_ihospregpci	textbox	ED Registration Number/Patient Chart Number		PCI/Hospital chart number from Health Records Department (not from ACR).<br><br>Transcribe as-is.<br><br>Caps Lock
p_phospregpci	textbox	Hospital Registration Number/Patient Chart Number		Hospital Registration Number<br><br>Transcribe as-is.<br><br>Caps Lock<br><br>Note this field will be not applicable for patients who are not transported to an ED or hospital.
p_hlthcrd_pci	div	Health Card		
p_ihealthcnpci	textbox	Number		This field is used to obtain the PCI/Hospital chart version of Health Card No.

PREDICT - Hospital Variables

Variable	Type	Caption	List Options	Abstraction Instruction
				Transcribe as-is
p_ihealthcvpci	textbox	Version Code		This field is used to obtain the PCI/Hospital chart version of Version No.<br><br>Transcribe as-is
p_isexpci	dropdown	Gender	listid: sex<br>0. female<br>1. male<br>2. not noted	This field is used to obtain the PCI/Hospital chart version of Gender.<br><br>Transcribe as-is
p_iphone1pci	textbox	Phone 1 (Home)		Patient s home telephone number.<br><br>Transcribe as-is
p_iphone2pci	textbox	Phone 2		Another contact number for the patient.<br><br>Transcribe as-is
p_ptrpci	div	PCI Centre Episode Date/Time Record		
p_sodt_pci	textbox	Symptom Onset Date		What was the date of the patient symptom onset?<br><br>Date value = yyyy/mm/dd<br><br>Source = Patient follow up information / PCI/Hospital chart
p_sotm_pci	textbox	Symptom Onset Time		What was the time of the patient symptom onset?<br><br>Numerical value based on 24 hour clock<br><br>00:00:00 – hour:min:sec<br><br>If no value for seconds data available – do not do not use value 00.<br><br>Source = Patient follow up information / PCI/Hospital chart
p_pciardt	textbox	PCI Centre Arrival Date		What was the date of the patient PCI centre arrival?<br><br>Date value = yyyy/mm/dd<br><br>Source = Patient follow up information / PCI/Hospital chart
p_pciartm	textbox	PCI Centre Arrival Time		What was the time of the patient PCI centre arrival?<br><br>Numerical value based on 24 hour clock<br><br>00:00:00 – hour:min:sec<br><br>If no value for seconds data available – do not do not use value 00.

PREDICT - Hospital Variables

Variable	Type	Caption	List Options	Abstraction Instruction
				Source = Patient follow up information / PCI/Hospital chart
p_recpcihosp	dropdown	Receiving PCI Centre/Hospital	listid: p_hosp<br>** See list items in appendix	What is the name of the Receiving PCI Centre/Hospital?<br>Source = Patient follow up information / PCI/Hospital chart<br>Pulldown menu = list of hospitals in database<br>Unknown Hospital = select when destination hospital will never be known<br>NOTE;<br>Do not provide the names of nursing home, rehabilitation, or other non-acute care facilities.<br>Transfer to one of these three entities constitutes an ''ED/hospital discharge, reclassification, or death''.
p_phxpci	dropdown	Patient Past History	listid: nyn<br>0. no<br>1. yes<br>2. not noted	Past History: Patient follow up information/ PCI/Hospital chart<br>If no past history is noted on the follow up information / PCI/Hospital chart, choose not noted.<br>HTNâ€”hypertension<br>Hyperlipidemia<br>Diabetes<br>Renal insufficiency/Failure<br>Chronic renal failure<br>Acute renal failure<br>Dialysis<br>Prior MIâ€”myocardial infarction<br>CADâ€”coronary artery disease<br>CHFâ€”congestive heart failure<br>Prior CABGâ€—coronary artery bypass graph<br>Prior PCI – percutaneous coronary intervention<br>ICDâ€—implantable cardiodefibrillator<br>Pacemaker<br>Peripheral vascular disease<br>DVT – deep venous trombosis<br>Tromboembolic history<br>Pulmonary embolism<br>Stroke/TIA/CVA

PREDICT - Hospital Variables

Variable	Type	Caption	List Options	Abstraction Instruction
				Smoking<br>Alcohol abuse<br>Cancer<br>Positive family history for heart disease
p_phphlt_pci	dropdown	Previously Healthy	listid: nyn<br>0. no<br>1. yes<br>2. not noted	Past History (from Patient follow up information / PCI/Hospital chart).<br>If no past history is noted on the Patient follow up information / PCI/Hospital chart, choose not noted.
p_phpcihyp	dropdown	Hypertension	listid: nyn<br>0. no<br>1. yes<br>2. not noted	Past History (from Patient follow up information / PCI/Hospital chart).<br>If no past history is noted on the Patient follow up information / PCI/Hospital chart, choose not noted.
p_phpcihl	dropdown	Hyperlipidemia	listid: nyn<br>0. no<br>1. yes<br>2. not noted	Past History (from Patient follow up information / PCI/Hospital chart).<br>If no past history is noted on the Patient follow up information / PCI/Hospital chart, choose not noted.
p_phpcidia	dropdown	Diabetes	listid: nyn<br>0. no<br>1. yes<br>2. not noted	Past History (from Patient follow up information / PCI/Hospital chart).<br>If no past history is noted on the Patient follow up information / PCI/Hospital chart, choose not noted.
p_phpcidiatype	dropdown	Diabetes Type	listid: diabtyp<br>0. Type 1<br>1. Type 2	Past History (from Patient follow up information / PCI/Hospital chart).<br>If no past history is noted on the Patient follow up information / PCI/Hospital chart, choose not noted.
p_phpcirif	dropdown	Renal Insufficiency/Failure	listid: nyn<br>0. no<br>1. yes<br>2. not noted	Past History (from Patient follow up information / PCI/Hospital chart).<br>If no past history is noted on the Patient follow up information / PCI/Hospital chart, choose not noted.
p_phpcicrf	dropdown	Chronic Renal Failure	listid: nyn<br>0. no<br>1. yes<br>2. not noted	Past History (from Patient follow up information / PCI/Hospital chart).<br>If no past history is noted on the Patient follow up information / PCI/Hospital chart, choose not noted.
p_phpciarf	dropdown	Acute Renal Failure	listid: nyn	Past History (from Patient follow up information / PCI/Hospital chart).

PREDICT - Hospital Variables

Variable	Type	Caption	List Options	Abstraction Instruction
			0. no<br>1. yes<br>2. not noted	If no past history is noted on the Patient follow up information / PCI/Hospital chart, choose not noted.
p_phpcidial	dropdown	Dialysis	listid: nyn<br>0. no<br>1. yes<br>2. not noted	Past History (from Patient follow up information / PCI/Hospital chart).<br>If no past history is noted on the Patient follow up information / PCI/Hospital chart, choose not noted.
p_phcarpci	dropdown	Cardiac	listid: nyn<br>0. no<br>1. yes<br>2. not noted	Past History (from Patient follow up information / PCI/Hospital chart).<br>If no past history is noted on the Patient follow up information / PCI/Hospital chart, choose not noted.
p_phpcimi	dropdown	Prior MI	listid: nyn<br>0. no<br>1. yes<br>2. not noted	Past History (from Patient follow up information / PCI/Hospital chart).<br>If no past history is noted on the Patient follow up information / PCI/Hospital chart, choose not noted.
p_phpcimidt	textbox	Prior MI date		What was the date of the patient prior MI?<br>Date value = yyyy/mm/dd<br>Source = Patient follow up information / PCI/Hospital chart
p_phpcicad	dropdown	CAD	listid: nyn<br>0. no<br>1. yes<br>2. not noted	Past History (from Patient follow up information / PCI/Hospital chart).<br>If no past history is noted on the Patient follow up information / PCI/Hospital chart, choose not noted.
p_phpcichf	dropdown	CHF	listid: nyn<br>0. no<br>1. yes<br>2. not noted	Past History (from Patient follow up information / PCI/Hospital chart).<br>If no past history is noted on the Patient follow up information / PCI/Hospital chart, choose not noted.
p_phpcicabg	dropdown	Prior CABG	listid: nyn<br>0. no<br>1. yes	Past History (from Patient follow up information / PCI/Hospital chart).

Variable	Type	Caption	List Options	Abstraction Instruction
			2. not noted	If no past history is noted on the Patient follow up information / PCI/Hospital chart, choose not noted.
p_phpcicabgdt	textbox	Prior CABG Date		What was the date of the patient prior CABG?<br><br>Date value = yyyy/mm/dd<br><br>Source = Patient follow up information / PCI/Hospital chart
p_phpcipci	dropdown	Prior PCI	listid: nyn<br>0. no<br>1. yes<br>2. not noted	Past History (from Patient follow up information / PCI/Hospital chart).<br><br>If no past history is noted on the Patient follow up information / PCI/Hospital chart, choose not noted.
p_phpcipcidt	textbox	Prior PCI Date		What was the date of the patient prior PCI?<br><br>Date value = yyyy/mm/dd<br><br>Source = Patient follow up information / PCI/Hospital chart
p_phpciicd	dropdown	ICD	listid: nyn<br>0. no<br>1. yes<br>2. not noted	Past History (from Patient follow up information / PCI/Hospital chart).<br><br>If no past history is noted on the Patient follow up information / PCI/Hospital chart, choose not noted.
p_phpcipac	dropdown	Pacemaker	listid: nyn<br>0. no<br>1. yes<br>2. not noted	Past History (from Patient follow up information / PCI/Hospital chart).<br><br>If no past history is noted on the Patient follow up information / PCI/Hospital chart, choose not noted.
p_phpcipvd	dropdown	Peripheral Vascular Disease	listid: nyn<br>0. no<br>1. yes<br>2. not noted	Past History (from Patient follow up information / PCI/Hospital chart).<br><br>If no past history is noted on the Patient follow up information / PCI/Hospital chart, choose not noted.
p_phpcidvt	dropdown	DVT	listid: nyn<br>0. no<br>1. yes<br>2. not noted	Past History (from Patient follow up information / PCI/Hospital chart).<br><br>If no past history is noted on the Patient follow up information / PCI/Hospital chart, choose not noted.

PREDICT - Hospital Variables

Variable	Type	Caption	List Options	Abstraction Instruction
p_phpciteh	dropdown	Tromboembolic History	listid: nyn<br>0. no<br>1. yes<br>2. not noted	Past History (from Patient follow up information / PCI/Hospital chart).<br>If no past history is noted on the Patient follow up information / PCI/Hospital chart, choose not noted.
p_phpcipe	dropdown	Pulmonary Embolism	listid: nyn<br>0. no<br>1. yes<br>2. not noted	Past History (from Patient follow up information / PCI/Hospital chart).<br>If no past history is noted on the Patient follow up information / PCI/Hospital chart, choose not noted.
p_phpcistr	dropdown	Stroke/TIA/CVA	listid: nyn<br>0. no<br>1. yes<br>2. not noted	Past History (from Patient follow up information / PCI/Hospital chart).<br>If no past history is noted on the Patient follow up information / PCI/Hospital chart, choose not noted.
p_phpcistrdt	textbox	Prior Stroke/TIA/CVA date		What was the date of the patient prior stroke/TIA/CVA?<br>Date value = yyyy/mm/dd<br>Source = Patient follow up information / PCI/Hospital chart
p_phpcismok	dropdown	Smoking	listid: nyn<br>0. no<br>1. yes<br>2. not noted	Past History (from Patient follow up information / PCI/Hospital chart).<br>If no past history is noted on the Patient follow up information / PCI/Hospital chart, choose not noted.
p_phpcicsmok	dropdown	Current Smoker	listid: nyn<br>0. no<br>1. yes<br>2. not noted	Past History (from Patient follow up information / PCI/Hospital chart).<br>If no past history is noted on the Patient follow up information / PCI/Hospital chart, choose not noted.
p_phpciaa	dropdown	Alcohol Abuse	listid: nyn<br>0. no<br>1. yes<br>2. not noted	Past History (from Patient follow up information / PCI/Hospital chart).<br>If no past history is noted on the Patient follow up information / PCI/Hospital chart, choose not noted.
p_phpcican	dropdown	Cancer	listid: nyn<br>0. no	Past History (from Patient follow up information / PCI/Hospital chart).

PREDICT - Hospital Variables

Variable	Type	Caption	List Options	Abstraction Instruction
			1. yes<br>2. not noted	If no past history is noted on the Patient follow up information / PCI/Hospital chart, choose not noted.
p_phpcipfhhd	dropdown	Positive Family History for Heart Diseases	listid: nyn<br>0. no<br>1. yes<br>2. not noted	Past History (from Patient follow up information / PCI/Hospital chart).<br>If no past history is noted on the Patient follow up information / PCI/Hospital chart, choose not noted.
p_mhxpci	dropdown	Patient Past Medication History	listid: nyn<br>0. no<br>1. yes<br>2. not noted	Patient Medication History (from ACR – do not use ED/Hospital records).<br>If no patient medication history is noted on the follow up information / PCI/Hospital chart, choose not noted.<br>Beta blockers<br>ASA – acetyl salicylic acid (Aspirin)<br>Fibrinolytics/Trombolytics<br>Clopidogrel (Plavix)/Ticlopidine<br>Glycoprotein (GP) IIb/IIIa inhibitors<br>Heparin<br>Low molecular weight heparin (LMWH)<br>Coumadin<br>Calcium channel blockers<br>Nitrates<br>ARB antagonists<br>ACE inhibitors<br>Lipid lowering drugs<br>Digitalis<br>Diuretics<br>Amiodarone<br>Insulin<br>Oral hypoglycemic agents<br>Mucomyst<br>Antidepressants<br>Anxiolytic (anti-anxiety)<br>Estrogens/OCP (oral contraceptive pills)<br>Immunosupressive medication<br>Bronchodilators/Inhaled steroids
p_mhno_pci	dropdown	None	listid: nyn<br>0. no<br>1. yes<br>2. not noted	Patient Medication History (Patient follow up information / PCI/Hospital chart).<br>If no patient medication history is noted on the Patient follow up information / PCI/Hospital chart, choose not noted.

PREDICT - Hospital Variables

Variable	Type	Caption	List Options	Abstraction Instruction
p_mhpciasa	dropdown	ASA (Aspirin)	listid: nyn<br><br>0. no<br>1. yes<br><br>2. not noted	Patient Medication History (Patient follow up information / PCI/Hospital chart).<br><br>If no patient medication history is noted on the Patient follow up information / PCI/Hospital chart, choose not noted.
p_mhpcifib	dropdown	Fibrinolytics/Trombolytics	listid: nyn<br><br>0. no<br>1. yes<br><br>2. not noted	Patient Medication History (Patient follow up information / PCI/Hospital chart).<br><br>If no patient medication history is noted on the Patient follow up information / PCI/Hospital chart, choose not noted.
p_mhpcifib type	dropdown	Fibrinolytics/Trombolytics Type	listid: drug<br><br>1. Streptokinase<br>2. TPA<br><br>3. TNK<br>4. RPA	Patient Medication History (Patient follow up information / PCI/Hospital chart).<br><br>If no patient medication history is noted on the Patient follow up information / PCI/Hospital chart, choose not noted.
p_mhpciclo p	dropdown	Clopidogrel (Plavix)/Ticlopidine	listid: nyn<br><br>0. no<br>1. yes<br><br>2. not noted	Patient Medication History (Patient follow up information / PCI/Hospital chart).<br><br>If no patient medication history is noted on the Patient follow up information / PCI/Hospital chart, choose not noted.
p_mhpcigly cinh	dropdown	Glycoprotein (GP) llb/llla Inhibitors	listid: nyn<br><br>0. no<br>1. yes<br><br>2. not noted	Patient Medication History (Patient follow up information / PCI/Hospital chart).<br><br>If no patient medication history is noted on the Patient follow up information / PCI/Hospital chart, choose not noted.
p_mhpcihep	dropdown	Heparin	listid: nyn<br><br>0. no<br>1. yes<br><br>2. not noted	Patient Medication History (Patient follow up information / PCI/Hospital chart).<br><br>If no patient medication history is noted on the Patient follow up information / PCI/Hospital chart, choose not noted.
p_mhpcilmw h	dropdown	Low Molecular Weight Heparin (LMWH)	listid: nyn	Patient Medication History (Patient follow up information / PCI/Hospital chart).

PREDICT - Hospital Variables

Variable	Type	Caption	List Options	Abstraction Instruction
			0. no<br>1. yes<br>2. not noted	If no patient medication history is noted on the Patient follow up information / PCI/Hospital chart, choose not noted.
p_mhpcicoum	dropdown	Coumadin	listid: nyn<br>0. no<br>1. yes<br>2. not noted	Patient Medication History (Patient follow up information / PCI/Hospital chart).<br>If no patient medication history is noted on the Patient follow up information / PCI/Hospital chart, choose not noted.
p_mhpcibb	dropdown	Beta Blockers	listid: nyn<br>0. no<br>1. yes<br>2. not noted	Patient Medication History (Patient follow up information / PCI/Hospital chart).<br>If no patient medication history is noted on the Patient follow up information / PCI/Hospital chart, choose not noted.
p_mhpciccb	dropdown	Calcium Channel Blockers	listid: nyn<br>0. no<br>1. yes<br>2. not noted	Patient Medication History (Patient follow up information / PCI/Hospital chart).<br>If no patient medication history is noted on the Patient follow up information / PCI/Hospital chart, choose not noted.
p_mhpcilant	dropdown	Nitrates/IV/Top	listid: nyn<br>0. no<br>1. yes<br>2. not noted	Patient Medication History (Patient follow up information / PCI/Hospital chart).<br>If no patient medication history is noted on the Patient follow up information / PCI/Hospital chart, choose not noted.
p_mhpciarba	dropdown	ARB Antagonists	listid: nyn<br>0. no<br>1. yes<br>2. not noted	Patient Medication History (Patient follow up information / PCI/Hospital chart).<br>If no patient medication history is noted on the Patient follow up information / PCI/Hospital chart, choose not noted.
p_mhpciacei	dropdown	ACE Inhibitors	listid: nyn<br>0. no	Patient Medication History (Patient follow up information / PCI/Hospital chart).

PREDICT - Hospital Variables

Variable	Type	Caption	List Options	Abstraction Instruction
			1. yes<br>2. not noted	If no patient medication history is noted on the Patient follow up information / PCI/Hospital chart, choose not noted.
p_mpcilld	dropdown	Lipid Lowering Drugs	listid: nyn<br>0. no<br>1. yes<br>2. not noted	Patient Medication History (Patient follow up information / PCI/Hospital chart).<br>If no patient medication history is noted on the Patient follow up information / PCI/Hospital chart, choose not noted.
p_mhpcidig	dropdown	Digitalis	listid: nyn<br>0. no<br>1. yes<br>2. not noted	Patient Medication History (Patient follow up information / PCI/Hospital chart).<br>If no patient medication history is noted on the Patient follow up information / PCI/Hospital chart, choose not noted.
p_mhpcidiu	dropdown	Diuretics	listid: nyn<br>0. no<br>1. yes<br>2. not noted	Patient Medication History (Patient follow up information / PCI/Hospital chart).<br>If no patient medication history is noted on the Patient follow up information / PCI/Hospital chart, choose not noted.
p_mhpciamio	dropdown	Amiodarone	listid: nyn<br>0. no<br>1. yes<br>2. not noted	Patient Medication History (Patient follow up information / PCI/Hospital chart).<br>If no patient medication history is noted on the Patient follow up information / PCI/Hospital chart, choose not noted.
p_mhpciins	dropdown	Insulin	listid: nyn<br>0. no<br>1. yes<br>2. not noted	Patient Medication History (Patient follow up information / PCI/Hospital chart).<br>If no patient medication history is noted on the Patient follow up information / PCI/Hospital chart, choose not noted.
p_mhpcioha	dropdown	Oral Hypoglycemic Agents	listid: nyn<br>0. no<br>1. yes	Patient Medication History (Patient follow up information / PCI/Hospital chart).<br>If no patient medication history is noted on the Patient follow up information / PCI/Hospital chart, choose not noted.

Variable	Type	Caption	List Options	Abstraction Instruction
			2. not noted	
p_mhpcimuc	dropdown	Mucomyst	listid: nyn<br><br>0. no<br>1. yes<br><br>2. not noted	Patient Medication History (Patient follow up information / PCI/Hospital chart).<br><br>If no patient medication history is noted on the Patient follow up information / PCI/Hospital chart, choose not noted.
p_mhpciantidep	dropdown	Antidepressants	listid: nyn<br><br>0. no<br>1. yes<br><br>2. not noted	Patient Medication History (Patient follow up information / PCI/Hospital chart).<br><br>If no patient medication history is noted on the Patient follow up information / PCI/Hospital chart, choose not noted.
p_mhpciantianx	dropdown	Anxiolytic (Anti-Anxiety)	listid: nyn<br><br>0. no<br>1. yes<br><br>2. not noted	Patient Medication History (Patient follow up information / PCI/Hospital chart).<br><br>If no patient medication history is noted on the Patient follow up information / PCI/Hospital chart, choose not noted.
p_mhpciestocp	dropdown	Estrogens/OCP	listid: nyn<br><br>0. no<br>1. yes<br><br>2. not noted	Patient Medication History (Patient follow up information / PCI/Hospital chart).<br><br>If no patient medication history is noted on the Patient follow up information / PCI/Hospital chart, choose not noted.
p_mhpciimmsup	dropdown	Immunosupressive Medication	listid: nyn<br><br>0. no<br>1. yes<br><br>2. not noted	Patient Medication History (Patient follow up information / PCI/Hospital chart).<br><br>If no patient medication history is noted on the Patient follow up information / PCI/Hospital chart, choose not noted.
p_mhpcibis	dropdown	Bronchodilators/Inhaled Steroids	listid: nyn<br><br>0. no<br>1. yes<br><br>2. not noted	Patient Medication History (Patient follow up information / PCI/Hospital chart).<br><br>If no patient medication history is noted on the Patient follow up information / PCI/Hospital chart, choose not noted.
p_mhpcioth	dropdown	Other	listid: nyn	Patient Medication History (Patient follow up information / PCI/Hospital chart).

PREDICT - Hospital Variables

Variable	Type	Caption	List Options	Abstraction Instruction
			0. no<br>1. yes<br>2. not noted	If no patient medication history is noted on the Patient follow up information / PCI/Hospital chart, choose not noted.
p_mhpciospc	textbox	Specify Other Not Listed		Patient Medication History (Patient follow up information / PCI/Hospital chart).<br><br>If no patient medication history is noted on the Patient follow up information / PCI/Hospital chart, choose not noted.
p_mhpcindet	dropdown	Not Determined	listid: nyn<br>0. no<br>1. yes<br>2. not noted	Patient Medication History (Patient follow up information / PCI/Hospital chart).<br><br>The Patient follow up information / PCI/Hospital chart selection of not determined implies the PCI staff (nurse/resident/fellow/doctor) did not ask as opposed to not noted which implies the abstractor could not find the information.
p_pcifib_div	div	PCI Fibrinolysis		
p_pcifib	dropdown	PCI Fibrinolysis	listid: nynr<br>0. no<br>1. yes<br>2. not recorded	Patient follow up information / PCI/Hospital chart<br><br>0=no<br><br>1=yes<br><br>2=not noted<br><br>If no PCI fibrinolysis is noted on the Patient follow up information / PCI/Hospital chart, choose not recorded.
p_pciflytic	dropdown	Fibrinolytic Given	listid: nynr<br>0. no<br>1. yes<br>2. not recorded	Did patient receive fibrinolytic therepy during the PCI course of care?<br><br>0=no<br><br>1=yes<br><br>2=not noted

PREDICT - Hospital Variables

Variable	Type	Caption	List Options	Abstraction Instruction
				Source = Patient follow up information / PCI/Hospital chart
p_pciflyticinel	dropdown	Fibrinolytic Ineligible	listid: ny<br>0. no<br>1. yes	Was patient ineligible to receive fibrinolytic therepy during the PCI course of care?<br>0=no<br>1=yes<br>2=not noted<br>Source = Patient follow up information / PCI/Hospital chart
p_pciflyticinelsp	textbox	Specify Reason		Please specify the reason why patient was deemed ineligible to receive fibrinolytic therepy during the PCI course of care.<br>Source = Patient follow up information / PCI/Hospital chart
p_pciflyticstartdt	textbox	Fibrinolysis Start Date		What was the fibrinolysis start date during the PCI course of patient care?<br>Date value = yyyy/mm/dd<br>Source = Patient follow up information / PCI/Hospital chart
p_pciflyticstarttm	textbox	Fibrinolysis Start Time		What was the fibrinolysis start time during the PCI course of patient care?<br>Numerical value based on 24 hour clock<br>00:00:00 – hour:min:sec<br>If no value for seconds data available – do not do not use value 00.<br>Source = Patient follow up information / PCI/Hospital chart
p_pciflyticdrug	dropdown	Fibrinolytic Drug Given	listid: drug2<br>1. TNK<br>2. tPA (TPA)<br>3. Activase (Alteplase)<br>4. RPA	Which fibrinolytic drug did patient recieve during the PCI course of care?<br>1=TNK (Tenecteplase, Recombinant TPA) no code<br>2=TPA (Tissue Plasminogen Activator) Code 661<br>3=Activase (Alteplase)

Variable	Type	Caption	List Options	Abstraction Instruction
			5. Other	4=RPA (Reteplase, Retavase) Code 646<br>5=other<br><br>Source = Patient follow up information / PCI/Hospital chart
p_pciflyticdrugsp	textbox	Specify Other		Please specify which other fibrinolytic drug patient received during the PCI course of care.<br><br>Source = Patient follow up information / PCI/Hospital chart
p_pciflyticctotd	textbox	Total Dose Given (mg)		Indicate the total in mg of TNK administered to patient throughout the PCI course of patient care.<br><br>Source = Patient follow up information / PCI/Hospital chart<br><br>TNK dose is usually given with IV administration of 30 mg for patients's weight <60 kg; 35 mg for > 60 to < 70 kg; 40 mg for > 70 to < 80 kg; 45 mg for > 80 to < 90 kg; and 50 mg for > 90 kg.
p_pciflyticctotd2	textbox	Total Dose Given (mg)		Indicate the total in mg of tPA administered to patient throughout the PCI course of patient care.<br><br>Source = Patient follow up information / PCI/Hospital chart
p_pciflyticctotd3	textbox	Total Dose Given (mg)		Indicate the total in mg of Activase (Alteplase) administered to patient throughout the PCI course of patient care.<br><br>Source = Patient follow up information / PCI/Hospital chart<br><br>Recommended total Activase (Alteplase) dose is based on patient's weight and for AMI the total dose should not exceed 100 mg.<br><br>Activase totsl dose is usually given with IV administration of accelerated infusion (1.5 hours): 15 mg IV bolus for all patients; then: 1) for patients >67 kg 50 mg infused over the next 30 minutes and then 35 mg over the next 60 minutes; 2) for patients ≤67 kg infusion of 0.75 mg/kg over next 30 minutes (not to exceed 50 mg); and then 0.5 mg/kg over 60 minutes (not to exceed 35 mg).

Variable	Type	Caption	List Options	Abstraction Instruction
				Activase could also be administered over the 3 hour IV infusion of the total dose of 100 mg: 60 mg in the first hour (bolus dose of 6-10 mg); 20 mg over the second hour and 20 mg over the third hour. For smaller patients (≤65 kg) a dose of 1.25 mg/kg will be administered over the period of 3 hours.
p_pciflytictotd4	textbox	Total Dose Given (Units)		Indicate the total in units of RPA administered to patient throughout the PCI course of patient care.<br><br>Source = Patient follow up information / PCI/Hospital chart<br><br>RPA dose is usually given with IV administration of 10 U bolus over 2 mintes; 30 minutes later give second 10 U IV bolus over 2 minutes (give NS flush before and after each bolus); give heparin and aspirin conjuctively.
p_pciflytictotd5	textbox	Total Dose Given		Indicate the total of other fibrinolytic drug patient received throughout the PCI course of patient care.<br><br>Source = Patient follow up information / PCI/Hospital chart
p_pciflyticinter	dropdown	Fibrinolysis Interrupted	listid: ny<br><br>0. no<br>1. yes	Was the fibrinolysis administration interrupted during the PCI course of care?<br><br>0=no<br>1=yes<br><br>Source = Patient follow up information / PCI/Hospital chart
p_pciflyticinrep	dropdown	Fibrinolysis Repeated	listid: ny<br><br>0. no<br>1. yes	Was the fibrinolysis administration repeated during the PCI course of patient care?<br><br>0=no<br>1=yes<br><br>Source = Patient follow up information / PCI/Hospital chart
p_pciflyticenddt	textbox	Fibrinolysis End Date		What was the fibrinolysis end date during the PCI course of patient care?<br><br>Date value = yyyy/mm/dd<br><br>Source = Patient follow up information / PCI/Hospital chart
p_pciflyticendtm	textbox	Fibrinolysis End Time		What was the fibrinolysis end time during the PCI course of patient care?

Variable	Type	Caption	List Options	Abstraction Instruction
				Numerical value based on 24 hour clock<br><br>00:00:00 – hour:min:sec<br><br>If no value for seconds data available – do not do not use value 00.<br><br>Source = Patient follow up information / PCI/Hospital chart
p_pciunfhep	dropdown	Unfractionated Heparin Given	listid: nynr<br><br>0. no<br>1. yes<br>2. not recorded	Did patient recive IV Unfractionated Heparin during the PCI course of care?<br><br>0=no<br><br>1=yes<br><br>2=not noted<br><br>Source = Patient follow up information / PCI/Hospital chart<br><br>Indicate if IV Unfractionated Heparin was administered at any time during the PCI course of care.
p_pciunfhepibdt	textbox	Initial Bolus Date		The date when administration of IV Unfractionated Heparin initial bolus dose is confirmed during the PCI course of care.<br><br>Source = Patient follow up information / PCI/Hospital chart
p_pciunfhepibtm	textbox	Initial Bolus Time		The earliest time noted when administration of IV Unfractionated Heparin initial bolus dose is confirmed during the PCI course of care.<br><br>Source = Patient follow up information / PCI/Hospital chart
p_pciunhepibdu	textbox	Initial Bolus Dose (Units)		The total in units of IV Unfractionated Heparin initial bolus dose administered to patient throughout the PCI course of patient care.<br><br>Source = Patient follow up information / PCI/Hospital chart

PREDICT - Hospital Variables

Variable	Type	Caption	List Options	Abstraction Instruction
				Unfractionated Heparin initial bolus dose is usually started with IV administration of 60 U/kg to maximum of 4000 U in conjuction with thrombolytics or 80 U/kg to maximum of 5000 U when no trombolytics were administered.
p_pciunfhepidt	textbox	Infusion Date		The date when administration of IV Unfractionated Heparin infusion is confirmed during the PCI course of patient care.<br><br>Source = Patient follow up information / PCI/Hospital chart
p_pciunfhepitm	textbox	Infusion Time		The earliest time noted when administration of IV Unfractionated Heparin infusion is confirmed during the PCI course of patient care.<br><br>Source = Patient follow up information / PCI/Hospital chart
p_pciunhepidu	textbox	Infusion Dose (Units)		The total in units of IV Unfractionated Heparin infusion dose administered to patient throughout the PCI course of patient care.<br><br>Source = Patient follow up information / PCI/Hospital chart<br><br>Unfractionated Heparin initial bolus dose is usually started with IV administration of 12 U/kg/hr to maximum of 1000 U/hr in conjuction with thrombolytics or 18 U/kg/hr to maximum of 1500 U/hr when no trombolytics were administered.<br><br>Dose is adjusted to maintain PTT at 1.5 – 2 times control value.
p_pcienox	dropdown	Enoxaparin Given	listid: nynr<br>0. no<br>1. yes<br>2. not recorded	Did patient recive IV Enoxaparin during the PCI course of patient care?<br><br>0=no<br>1=yes<br>2=not noted<br><br>Source = Patient follow up information / PCI/Hospital chart

PREDICT - Hospital Variables

Variable	Type	Caption	List Options	Abstraction Instruction
				Indicate if IV Unfractionated Heparin was administered at any time during during the PCI course of patient care.
p_pcienoini	dropdown	Inital bolus dose given	listid: ny<br><br>0. no<br>1. yes	The date when administration of IV Enoxaparin initial bolus dose is confirmed during the PCI course of patient care.<br><br>Source = Patient follow up information / PCI/Hospital chart
p_pcienoxdt	textbox	Initial Bolus Date		The date when administration of IV Enoxaparin initial bolus dose is confirmed during the PCI course of patient care.<br><br>Source = Patient follow up information / PCI/Hospital chart
p_pcienoxtm	textbox	Initial Bolus Time		The earliest time noted when administration of IV Enoxaparin initial bolus dose is confirmed during the PCI course of patient care.<br><br>Source = Patient follow up information / PCI/Hospital chart
p_pcienoxibdmg	textbox	Initial Bolus Dose (mg)		The total of IV Enoxaparin initial bolus dose administered to patient throughout the PCI course of patient care.<br><br>Source = Patient follow up information / PCI/Hospital chart<br><br>Enoxaparin initial bolus dose is usually started with IV administration of 30 mg for patients < 75 years of age. Patients ≥ 75 years of age do not receive Enoxaparin IV bolus.
p_pcisceno	dropdown	Subcutoneous (sc) Enoxaparin	listid: ny<br>0. no<br>1. yes	
p_pcienoxidt	textbox	SC injection date		The date when administration of subcutaneous (SC) Enoxaparin infusion is confirmed during the PCI course of patient care.<br><br>Source = Patient follow up information / PCI/Hospital chart
p_pcienoxitm	textbox	SC injection time		The earliest time noted when administration of SC Enoxaparin infusion is confirmed during the PCI course of patient care.<br><br>Source = Patient follow up information / PCI/Hospital chart

PREDICT - Hospital Variables

Variable	Type	Caption	List Options	Abstraction Instruction
p_pcienoxidmg	textbox	SC injection dose (mg)		The total of SC Enoxaparin infusion dose administered to patient throughout the PCI course of patient care.<br><br>Source = Patient follow up information / PCI/Hospital chart<br><br>Enoxaparin infusion dose is usually started with SC administration of 1 mg/kg (maximum 100 mg) for patients < 75 years of age and 0.75 mg/kg (maximum 75 mg) for patients ≥ 75 years of age.
p_pcilmwh	dropdown	Other LMWH product given	listid: nynr<br>0. no<br>1. yes<br>2. not recorded	
p_pcilmwhso	textbox	Specify Other		
p_pcilmwhft	textbox	First given time		
p_pcilmwhdose	textbox	Total dose (mg)		
p_pciclop	dropdown	Clopidogrel (Plavix)	listid: nynr<br>0. no<br>1. yes<br>2. not recorded	Did patient recive Clopidogrel during the PCI course of patient care?<br><br>0=no<br>1=yes<br>2=not noted<br><br>Source = Patient follow up information / PCI/Hospital chart<br><br>Indicate if Clopidogrel was administered at any time during during the PCI course of patient care.
p_pciclopttm	textbox	First Given Time		The earliest time noted when administration of Clopidogrel is confirmed during the PCI course of patient care.<br><br>Source = Patient follow up information / PCI/Hospital chart
p_pciclopttotd	textbox	Total Dose Given (mg)		Indicate the total in mg of Clopidogrel administered to patient throughout the PCI course of care.

Variable	Type	Caption	List Options	Abstraction Instruction
				Source = Patient follow up information / PCI/Hospital chart<br><br>Clopidogrel dose is usually given with a loading dose of 300 mg for 75 years and under, and 75 mg for 76 years and older. The decision to administer additional clopidogrel to patients is usually left to the discretion of the interventional cardiologist.
p_pciglycinh	dropdown	Glycoprotein (GP) llb/llla Inhibitors	listid: nynr<br><br>0. no<br>1. yes<br>2. not recorded	Did patient recive IV glycoprotein IIb/IIIa inhibitors during the PCI course of patient care?<br><br>0=no<br><br>1=yes<br><br>2=not noted<br><br>Source = Patient follow up information / PCI/Hospital chart<br><br>Indicate if IV Glycoprotein IIb/IIIa Inhibitors were administered at any time during during the PCI course of patient care.
p_pciglycinhtype	dropdown	Glycoprotein (GP) llb/llla Inhibitor Type	listid: glycinh2<br><br>1. Abciximab (ReoPro)<br><br>2. Eptifibatide (Intergrilin)<br>3. Tirofiban (Aggrastat)<br>4. Other	What type of glycoprotein IIb/IIa inhibitor did patient recive during the PCI course of patient care?<br><br>1=Abciximab (ReoPro)<br><br>2=Eptifibatide (Integrilin)<br><br>3=Tirofiban (Aggrastat)<br>4= other (specify)<br><br>Source = Patient follow up information / PCI/Hospital chart<br><br>Indicate what type of Glycoprotein IIb/IIIa Inhibitor was administered at any time during during the PCI course of patient care.
p_pciglycinhtypesp	textbox	Specify Other		Please specify which other glycoprotein IIb/IIa inhibitor patient received during the PCI course of patient care.

PREDICT - Hospital Variables

Variable	Type	Caption	List Options	Abstraction Instruction
				Source = Patient follow up information / PCI/Hospital chart
p_pciglycinhtm	textbox	First Given Time		The earliest time noted when administration of glycoprotein IIb/IIa inhibitor is confirmed during the PCI course of patient care.<br><br>Source = Patient follow up information / PCI/Hospital chart
p_pciglycinhtotd	textbox	Total Dose Given (mg)		Indicate the total in mg of Abciximab administered to patient throughout the PCI course of care.<br><br>Source = Patient follow up information / PCI/Hospital chart<br><br>Abciximab dose is usually given with IV bolus administration of 0.25 mg/kg 10-60 minutes before the start of PCI, followed by a continuous intravenous infusion of 0.125 Âµg/kg/min (to a maximum of 10 Âµg/min) for 12 hours.
p_pciglycinhtotd2	textbox	Total Dose Given (mg)		Indicate the total in mg of Eptifibatide administered to patient throughout the PCI course of care.<br><br>Source = Patient follow up information / PCI/Hospital chart<br><br>Eptifibatide dose is usually given with IV bolus administration of 180 Âµg/kg as soon as possible followed by a continuous infusion of 2.0 Âµg/kg/min until hospital discharge or initiation of CABG surgery, up to 72 hours. If a patient is to undergo a percutaneous coronary intervention (PCI) while receiving eptifibatide, the infusion should be continued up to hospital discharge, or for up to 18 to 24 hours after the procedure, whichever comes first, allowing for up to 96 hours of therapy .
p_pciglycinhtotd3	textbox	Total Dose Given (mg)		Indicate the total in mg of Tirofiban administered to patient throughout the PCI course of care.<br><br>Source = Patient follow up information / PCI/Hospital chart<br><br>Tirofiban dose is usually given with IV at an initial rate of 0.4 Âµg/kg/min for 30 minutes and then continued at 0.1 Âµg/kg/min.

PREDICT - Hospital Variables

Variable	Type	Caption	List Options	Abstraction Instruction
p_pciglycinhtotd4	textbox	Total Dose Given (mg)		Indicate the total in mg of the other of glycoprotein IIb/IIa inhibitor administered to patient throughout the PCI course of care.<br><br>Source = Patient follow up information / PCI/Hospital chart
p_pcitrombinh	dropdown	Thrombin Inhibitors	listid: nynr<br><br>0. no<br>1. yes<br>2. not recorded	Did patient recive trombin inhibitors during the PCI course of patient care?<br><br>0=no<br><br>1=yes<br><br>2=not noted<br><br>Source = Patient follow up information / PCI/Hospital chart<br><br>Indicate if trombin inhibitors were administered at any time during during the PCI course of patient care.
p_pcitrombinhtype	dropdown	Thrombin Inhibitors Type	listid: trombinh<br><br>1. Bivalirudin<br>2. Lepirudin<br>3. Desirudin<br>4. Other	What type of trombin inhibitor did patient recive during the PCI course of patient care?<br><br>1=Bivalirudin (Angiomax)<br>2=Lepirudin (Refludan)<br>3=Desirudin<br>4=Other<br><br>Source = Patient follow up information / PCI/Hospital chart<br><br>Indicate what type of trombin inhibitor administered at any time during during the PCI course of patient care.
p_pcithrombinhtypesp	textbox	Specify Other		Please specify which other thrombin inhibitor patient received during the PCI course of patient care.<br><br>Source = Patient follow up information / ED chart

PREDICT - Hospital Variables

Variable	Type	Caption	List Options	Abstraction Instruction
p_pcitrombinhtm	textbox	First Given Time		The earliest time noted when administration of trombin inhibitor is confirmed during the PCI course of patient care.<br><br>Source = Patient follow up information / PCI/Hospital chart
p_pcitrombinhtotd1	textbox	Total Does Given (mg)		Indicate the total in mg of Bivalirudin (Angiomax) administered to patient throughout the PCI course of care.<br><br>Source = Patient follow up information / PCI/Hospital chart<br><br>Bivalrudin total dose is usually given with IV loading dose (fast injection) of 0.75 mg/kg. Additional bolus dose of 0.3 mg/kg might be administered if necessary. This is followed by IV infusion of 1.75 mg/kg/hour for the duration of PCI. After 4 hours additional IV infusion of 0.2 mg/kh/hour for up to 20 hours might be administered.
p_pcitrombinhtotd2	textbox	Total Dose Given (mg)		Indicate the total in mg of Lepirudin (Refludan) administered to patient throughout the PCI course of care.<br><br>Source = Patient follow up information / PCI/Hospital chart<br><br>Lepirudin total dose is usually given with IV loading dose (slowly) of 0.4 mg/kg (up to 110 kg) followed by IV infusion of 0.15 mg/kg/hour for 2-10 days or londer if clinically needed.
p_pcitrombinhtotd3	textbox	Total Dose Given (mg)		Indicate the total in mg of Desirudin administered to patient throughout the PCI course of care.<br><br>Source = Patient follow up information / PCI/Hospital chart
p_pcitrombinhtotd4	textbox	Total Dose Given (mg)		Indicate the total in mg of other trombin inhibitor administered to patient throughout the PCI course of care.

Variable	Type	Caption	List Options	Abstraction Instruction
				Source = Patient follow up information / PCI/Hospital chart
p_pciasa2	dropdown	ASA (Aspirin) Given	listid: nynr<br><br>0. no<br>1. yes<br>2. not recorded	Indicate if ASA was administered at any time during the PCI course of patient care.<br><br>0=no<br><br>1=yes<br><br>2=not noted<br><br>Source = Patient follow up information / PCI/Hospital chart
p_pciasaftm	textbox	First Given Time		The earliest time noted when administration of ASA is confirmed during the PCI course of patient care.<br><br>Source = Patient follow up information / PCI/Hospital chart
p_pciasamg	textbox	Total Dose (mg)		The total in mg of all doses of ASA administered to patient throughout the PCI course of patient care.<br><br>Â· Usual dose of ASA is 160 mg (2x80 mg) for the patient to chew and swallow.<br><br>Source = Patient follow up information / PCI/Hospital chart
p_pcinitro2	dropdown	Nitrolingual Spray Given	listid: nynr<br><br>0. no<br>1. yes<br>2. not recorded	Indicate if Nitrolingual spray was administered at any time during the PCI course of patient care.<br><br>0=no<br><br>1=yes<br><br>2=not noted<br><br>Source = Patient follow up information / PCI/Hospital chart
p_pcinitro2ftm	textbox	First Given Time		

PREDICT - Hospital Variables

Variable	Type	Caption	List Options	Abstraction Instruction
p_pcinitro2mg	textbox	Total Dose(mg)		
p_pcinitropatch	dropdown	Nitro Patch	listid: ny<br>0. no<br>1. yes	
p_pcinitroftm	textbox	First Given Time		The earliest time noted when administration of Nitrolingual spray 0.4 mg/spray is confirmed during the PCI course of patient care.<br><br>Source = Patient follow up information / PCI/Hospital chart
p_pcinitromg	textbox	Total Dose (mg)		The total in mg of all doses of Nitrolingual spray administered to patient throughout the PCI course of care.<br><br>Â· Maximal number of Nitrolingual spray 0.4 mg/spray is 3; single spray is administered every 5 minutes.<br><br>Source = Patient follow up information / PCI/Hospital chart
p_pcinitrodrip	dropdown	Nitro IV Drip Given	listid: nynr<br><br>0. no<br>1. yes<br>2. not recorded	Indicate if Nitro IV drip was administered at any time during the PCI course of patient care.<br><br>0=no<br><br>1=yes<br><br>2=not noted<br><br>Source = Patient follow up information / PCI/Hospital chart
p_pcinitrodripftm	textbox	First Given Time		The earliest time noted when administration of Nitro IV drip during the PCI course of patient care.<br><br>Source = Patient follow up information / PCI/Hospital chart
p_pcinitromgugmin	textbox	Total Dose (mg)		The total in mg of Nitro IV drip administered to patient throughout the PCI course of care.<br><br>Â· Nitro drip is usually started with IV administration of 0.1 Âµg/kg/min and then tittred to up to 200 Âµg/min.<br><br>Source = Patient follow up information / PCI/Hospital chart

PREDICT - Hospital Variables

Variable	Type	Caption	List Options	Abstraction Instruction
p_pcicaren_div	div	PCI Lab and Cardiac Enzymes		
p_pcilabtest	dropdown	Were Lab Tests Done in PCI?	listid: ny<br><br>0. no<br>1. yes	Were lab tests done during the PCI course of patient care?<br><br>0=no<br>1=yes<br><br>Source = Patient follow up information / PCI/Hospital chart<br><br>Indicate if lab tests were done at any time during the PCI course of patient care.
p_pcilabtestdt	textbox	Lab Tests Date		The date when the lab tests were done first time during the PCI course of patient care.<br><br>Source = Patient follow up information / PCI/Hospital chart
p_pcilabtesttm	textbox	Lab Tests Time		The time when the lab tests were done first time during the PCI course of patient care.<br><br>Source = Patient follow up information / PCI/Hospital chart
p_pcichol	dropdown	Cholesterol	listid: ny<br><br>0. no<br>1. yes	Indicate if cholesterol was measured during the PCI course of patient care?<br><br>0=no<br>1=yes<br><br>Source = Patient follow up information / PCI/Hospital chart
p_pcicholllev	textbox	Choesterol Levels (mmol/L)		Indicate the first cholesterol levels measured during the PCI course of patient care.<br><br>Source = Patient follow up information / PCI/Hospital chart
p_pcihdl	dropdown	HDL	listid: ny<br><br>0. no<br>1. yes	Indicate if HDL was measured during the PCI course of patient care?<br><br>0=no<br>1=yes<br><br>Source = Patient follow up information / PCI/Hospital chart
p_pcihdllev	textbox	HDL Levels		Indicate the first HDL levels measured during the PCI course of patient care.<br><br>Source = Patient follow up information / PCI/Hospital chart
p_pcildl	dropdown	LDL	listid: ny<br><br>0. no<br>1. yes	Indicate if LDL was measured during the PCI course of patient care?<br><br>0=no

Variable	Type	Caption	List Options	Abstraction Instruction
				1=yes<br><br>Source = Patient follow up information / PCI/Hospital chart
p_pcildllev	textbox	LDL Levels		Indicate the first LDL levels measured during the PCI course of patient care.<br><br>Source = Patient follow up information / PCI/Hospital chart
p_pcitriglyc	dropdown	Triglycerides	listid: ny<br><br>0. no<br>1. yes	Indicate if tryglicerides were measured during the PCI course of patient care?<br><br>0=no<br>1=yes<br><br>Source = Patient follow up information / PCI/Hospital chart
p_pcitriglyclev	textbox	Triglycerides Levels (mmol/L)		Indicate the first tryglicerides levels measured during the PCI course of patient care.<br><br>Source = Patient follow up information / PCI/Hospital chart
p_pcigluc	dropdown	Glucose	listid: ny<br><br>0. no<br>1. yes	Indicate if glucose was measured during the PCI course of patient care?<br><br>0=no<br>1=yes<br><br>Source = Patient follow up information / PCI/Hospital chart
p_pcigluclev	textbox	Glucose Levels		Indicate the first glucose levels measured during the PCI course of patient care.<br><br>Source = Patient follow up information / PCI/Hospital chart
p_pcicreat	dropdown	Creatinine	listid: ny<br><br>0. no<br>1. yes	Indicate if creatinine was measured during the PCI course of patient care?<br><br>0=no<br>1=yes<br><br>Source = Patient follow up information / PCI/Hospital chart
p_pcicreatlev	textbox	Creatinine Levels		Indicate the first creatinine levels measured during the PCI course of patient care.<br><br>Source = Patient follow up information / PCI/Hospital chart
p_pcicaren	dropdown	Were Cardiac Enzymes Done in PCI?	listid: ny<br><br>0. no	Were cardiac anzymes done during the PCI course of patient care?

PREDICT - Hospital Variables

Variable	Type	Caption	List Options	Abstraction Instruction
			1. yes	0=no<br>1=yes<br><br>Source = Patient follow up information / PCI/Hospital chart<br><br>Indicate if cardiac enzymes were done at any time during the PCI course of patient care.
p_pcifcarendt	textbox	First Cardiac Enzymes Measurement Date		The date when the cardiac enzymes were measured first time during the PCI course of patient care.<br><br>Source = Patient follow up information / PCI/Hospital chart
p_pcifcarentm	textbox	First Cardiac Enzymes Measurement Time		The time when the cardiac enzymes were measured first time during the PCI course of patient care.<br><br>Source = Patient follow up information / PCI/Hospital chart
p_pcifck	dropdown	First Creatine Kinase (CK)	listid: ny<br><br>0. no<br>1. yes	Indicate if first CK levels were measured during the PCI course of patient care?<br><br>0=no<br>1=yes<br><br>Source = Patient follow up information / PCI/Hospital chart
p_pcifcklev	textbox	First CK Levels (U/L)		Indicate the first CK levels measured during the PCI course of patient care.<br><br>Source = Patient follow up information / PCI/Hospital chart
p_pcifckmb	dropdown	First Creatine Kinase - MB (CK-MB)	listid: ny<br><br>0. no<br>1. yes	Indicate if first CK-MB levels were measured during the PCI course of patient care?<br><br>0=no<br>1=yes<br><br>Source = Patient follow up information / PCI/Hospital chart
p_pcifckmblev	textbox	First CK-MB Levels (U/L)		Indicate the first CK-MB levels measured during the PCI course of patient care.<br><br>Source = Patient follow up information / PCI/Hospital chart
p_pciftropi	dropdown	First Troponin I	listid: ny<br><br>0. no<br>1. yes	Indicate if first Troponin I levels were measured during the PCI course of patient care?<br><br>0=no<br>1=yes

Variable	Type	Caption	List Options	Abstraction Instruction
				Source = Patient follow up information / PCI/Hospital chart
p_pciftropilev	textbox	First Troponin I Levels (Âµg/L)		Indicate the first Troponin I levels measured during the PCI course of patient care.<br><br>Source = Patient follow up information / PCI/Hospital chart
p_pciftropt	dropdown	First Troponin T	listid: ny<br><br>0. no<br>1. yes	Indicate if first Troponin T levels were measured during the PCI course of patient care?<br><br>0=no<br>1=yes<br><br>Source = Patient follow up information / PCI/Hospital chart
p_pciftroptlev	textbox	First Troponin T Levels (Âµg/L)		Indicate the first Troponin T levels measured during the PCI course of patient care.<br><br>Source = Patient follow up information / PCI/Hospital chart
p_pcipcarendt	textbox	Peak Cardiac Enzymes Measurement Date		The date when the peak cardiac enzymes were measured during the PCI course of patient care.<br><br>Source = Patient follow up information / PCI/Hospital chart
p_pcipcarentm	textbox	Peak Cardiac Enzymes Measurement Time		The time when the peak cardiac enzymes were measured during the PCI course of patient care.<br><br>Source = Patient follow up information / PCI/Hospital chart
p_pcipck	dropdown	Peak Creatine Kinase (CK)	listid: ny<br><br>0. no<br>1. yes	Indicate if peak CK levels were measured during the PCI course of patient care?<br><br>0=no<br>1=yes<br><br>Source = Patient follow up information / PCI/Hospital chart
p_pcipcklev	textbox	Peak CK Levels (U/L)		Indicate the peak CK levels measured during the PCI course of patient care.<br><br>Source = Patient follow up information / PCI/Hospital chart
p_pcipckmb	dropdown	Peak Creatine Kinase - MB (CK-MB)	listid: ny<br><br>0. no<br>1. yes	Indicate if peak CK-MB levels were measured during the PCI course of patient care?

PREDICT - Hospital Variables

Variable	Type	Caption	List Options	Abstraction Instruction
				0=no<br>1=yes<br>Source = Patient follow up information / PCI/Hospital chart
p_pcipckmblev	textbox	Peak CK-MB Levels (U/L)		Indicate the peak CK-MB levels measured during the PCI course of patient care.<br>Source = Patient follow up information / PCI/Hospital chart
p_pciptropi	dropdown	Peak Troponin I	listid: ny<br>0. no<br>1. yes	Indicate if peak Troponin I levels were measured during the PCI course of patient care?<br>0=no<br>1=yes<br>Source = Patient follow up information / PCI/Hospital chart
p_pciptropilev	textbox	Peak Troponin I Levels (Âµg/L)		Indicate the peak Troponin I levels measured during the PCI course of patient care.<br>Source = Patient follow up information / PCI/Hospital chart
p_pciptropt	dropdown	Peak Troponin T	listid: ny<br>0. no<br>1. yes	Indicate if peak Troponin T levels were measured during the PCI course of patient care?<br>0=no<br>1=yes<br>Source = Patient follow up information / PCI/Hospital chart
p_pciptroptlev	textbox	Peak Troponin T Levels		Indicate the peak Troponin T levels measured during the PCI course of patient care.<br>Source = Patient follow up information / PCI/Hospital chart
p_pciedecg_div	div	ECG Recording in PCI		
p_pciecged	dropdown	Does PCI ECG Recording Exist?	listid: ny<br>0. no<br>1. yes	Did PCI personnel record the patient ECG?<br>0=no<br>1=yes

Variable	Type	Caption	List Options	Abstraction Instruction
				Indicate no or yes, whether PCI ECG recording exists.<br><br>Source = Patient follow up information / PCI/Hospital chart
p_pcifecg_div	div	First PCI ECG (first one on arrival in PCI)		
p_pcifecg	dropdown	Does First PCI ECG Recording Exist?	listid: ny<br><br>0. no<br>1. yes	Did PCI personnel record the patient ECG on arrival in PCI?<br><br>0=no<br>1=yes<br><br>Indicate no or yes, whether PCI ECG recording exists.<br><br>Source = Patient follow up information / PCI/Hospital chart
p_pcifedecgdt	textbox	Date		What was the date of the patient first ECG in PCI?<br><br>Date value = yyyy/mm/dd<br><br>Source = Patient follow up information / PCI/Hospital chart
p_pcifedecgtm	textbox	Time		What was the time of the patient first ECG in PCI?<br><br>Numerical value based on 24 hour clock<br><br>00:00:00 – hour:min:sec<br><br>If no value for seconds data available – do not do not use value 00<br><br>Source = Patient follow up information / PCI/Hospital chart
p_pcifedecgrev	dropdown	First ECG Reviewed	listid: nyu<br><br>0. no<br>1. yes<br>2. unknown	Indicate whether or not the first PCI ECG was reviewed by the attending PCI physician site or if the attendant annotations and QA data was generated solely by the device software.<br><br>0=no<br>1=yes<br>2=unknown<br><br>The goal is for sites to review the recordings so as to ''correct'' any oversights by the software.<br>Source = Patient follow up information / PCI/Hospital chart
p_pcifedecgd	dropdown	First ECG Data Exists	listid: ny	Is there the first PCI ECG data available on request?

PREDICT - Hospital Variables

Variable	Type	Caption	List Options	Abstraction Instruction
			0. no<br>1. yes	0=no<br>1=yes<br><br>Source = Patient follow up information / PCI/Hospital chart
p_pcifedecgdgca	dropdown	First ECG Diagnosis - Computer Assisted	listid: ny<br><br>0. no<br>1. yes	Indicate whether or not there was computer assisted diagnosis for the first PCI ECG.<br><br>0=no<br>1=yes<br><br>Source = Patient follow up information / PCI/Hospital chart
p_pcifedecgdgsp	textbox	First ECG Diagnosis - Computer Assisted - Specify 1		Please specify computer assisted diagnosis for the first PCI ECG.<br><br>Value = text<br><br>Source = Patient follow up information / PCI/Hospital chart
p_pcifedecgdgsp_2	textbox	First ECG Diagnosis - Computer Assisted - Specify 2 (cont)		
p_pcifedecgdgsp_3	textbox	First ECG Diagnosis - Computer Assisted - Specify 3 (cont)		
p_pcifedecgdg	textbox	First ECG Diagnosis 1		Indicate the diagnosis based on patient first PCI ECG made by PCI attending physician.<br><br>Source = Patient follow up information / PCI/Hospital chart
p_pcifedecgdg_2	textbox	First ECG Diagnosis 2 (cont)		
p_pcifedecgdg_3	textbox	First ECG Diagnosis 3 (cont)		
p_pcistefedecg	dropdown	ST Elevation	listid: nyn<br><br>0. no<br>1. yes<br>2. not noted	Is there ST elevation on patient first PCI ECG?<br><br>0=no<br>1=yes<br><br>Source = Patient follow up information / PCI/Hospital chart
p_pcistemicrfedecg	dropdown	STEMI Criteria Met	listid: nynr<br><br>0. no<br>1. yes<br>2. not recorded	Is ST Elevation Myocardial Infarction (STEMI) criteria met on patient diagnostic PCI ECG?<br><br>0=no<br><br>1=yes

PREDICT - Hospital Variables

Variable	Type	Caption	List Options	Abstraction Instruction
				2=not noted<br><br>Source = Patient follow up information / PCI/Hospital chart
p_pcistemilfedecg	textbox	STEMI Location		Indicate, if possible the STEMI location based on patient first ECG in PCI.<br><br>Source = Patient follow up information / PCI/Hospital chart
p_pcidgedecg_div	div	Diagnostic PCI ECG (prior to fibrinolysis)		
p_pcidgedecg	dropdown	Does Diagnostic PCI ECG Recording Exist Just Prior to Reperfusion Therapy?	listid: ny<br><br>0. no<br>1. yes	Did PCI personnel record the patient diagnostic ECG in PCI just prior to reperfusion therapy?<br><br>0=no<br>1=yes<br><br>Indicate no or yes, whether diagnostic PCI ECG recording exists.<br><br>Source = Patient follow up information / PCI/Hospital chart
p_pcidgedecgdt	textbox	Date		What was the date of the patient diagnostic ECG in PCI?<br><br>Date value = yyyy/mm/dd<br><br>Source = Patient follow up information / PCI/Hospital chart
p_pcidgedecgtm	textbox	Time		What was the time of the patient diagnostic ECG in PCI?<br><br>Numerical value based on 24 hour clock<br><br>00:00:00 – hour:min:sec<br><br>If no value for seconds data available – do not do not use value 00<br><br>Source = Patient follow up information / PCI/Hospital chart
p_pcidgedecgrev	dropdown	Diagnostic ECG Reviewed	listid: nyu<br><br>0. no<br>1. yes<br>2. unknown	Indicate whether or not the diagnostic PCI ECG was reviewed by the attending PCI physician site or if the attendant annotations and QA data was generated solely by the device software.<br><br>0=no<br>1=yes<br>2=unknown

PREDICT - Hospital Variables

Variable	Type	Caption	List Options	Abstraction Instruction
				The goal is for sites to review the recordings so as to ''correct'' any oversights by the software.<br>Source = Patient follow up information / PCI/Hospital chart
p_pcidgedecgd	dropdown	Diagnostic ECG Data Exists	listid: ny<br>0. no<br>1. yes	Is there the diagnostic PCI ECG data available on request?<br>0=no<br>1=yes<br>Source = Patient follow up information / PCI/Hospital chart
p_pcidgedecgdgca	dropdown	Diagnostic ECG Diagnosis - Computer Assisted	listid: ny<br>0. no<br>1. yes	Indicate whether or not there was computer assisted diagnosis for the diagnostic PCI ECG.<br>0=no<br>1=yes<br>Source = Patient follow up information / PCI/Hospital chart
p_pcidgedecgdgsp	textbox	Diagnostic ECG Diagnosis - Computer Assisted - Specify 1		Please specify computer assisted diagnosis for the diagnostic PCI ECG.<br>Value = text<br>Source = Patient follow up information / PCI/Hospital chart
p_pcidgedecgdgsp_2	textbox	Diagnostic ECG Diagnosis - Computer Assisted - Specify 2 (cont)		
p_pcidgedecgdgsp_3	textbox	Diagnostic ECG Diagnosis - Computer Assisted - Specify 3 (cont)		
p_pcidgedecgdg	textbox	Diagnostic ECG Diagnosis 1		Indicate the diagnosis based on patient diagnostic PCI ECG made by PCI attending physician.<br>Source = Patient follow up information / PCI/Hospital chart
p_pcidgedecgdg_2	textbox	Diagnostic ECG Diagnosis 2 (cont)		
p_pcidgedecgdg_3	textbox	Diagnostic ECG Diagnosis 3 (cont)		
p_pcistedgedecg	dropdown	ST Elevation	listid: nyn<br>0. no<br>1. yes<br>2. not noted	Is there ST elevation on patient diagnostic PCI ECG?<br>0=no<br>1=yes<br>Source = Patient follow up information / PCI/Hospital chart

PREDICT - Hospital Variables

Variable	Type	Caption	List Options	Abstraction Instruction
p_pcistemicrdgedecg	dropdown	STEMI Criteria Met	listid: nynr<br><br>0. no<br>1. yes<br>2. not recorded	Is ST Elevation Myocardial Infarction (STEMI) criteria met on patient diagnostic PCI ECG?<br><br>0=no<br>1=yes<br><br>Source = Patient follow up information / PCI/Hospital chart
p_pcistemildgedecg	textbox	STEMI Location		Indicate, if possible the STEMI location based on patient diagnostic ECG in PCI.<br><br>Source = Patient follow up information / PCI/Hospital chart
p_pcipfedecgxst_div	div	Postfibrinolytic PCI ECG (60-90 minutes after fibrinolysis)		
p_pcipfedecgxst	dropdown	Does Posttrombolytic PCI ECG Recording Exist?	listid: ny<br><br>0. no<br>1. yes	Did PCI personnel record the patient posttretment ECG 60-90 minutes after tretment received in PCI?<br><br>0=no<br>1=yes<br><br>Indicate no or yes, whether posttretment (60-90 min) PCI ECG recording exists.<br><br>Source = Patient follow up information / PCI/Hospital chart
p_pcipfedecgdt	textbox	Date		What was the date of the patient posttretment (60-90 min) ECG in PCI?<br><br>Date value = yyyy/mm/dd<br><br>Source = Patient follow up information / PCI/Hospital chart
p_pcipfedecgtm	textbox	Time		What was the time of the patient posttretment (60-90 min) ECG in PCI?<br><br>Numerical value based on 24 hour clock<br><br>00:00:00 – hour:min:sec<br><br>If no value for seconds data available – do not do not use value 00<br><br>Source = Patient follow up information / PCI/Hospital chart
p_pcipfedecgrev	dropdown	Posttreatment ECG (60-90 minutes) Reviewed	listid: nyu<br><br>0. no	Indicate whether or not the posttretment PCI ECG (60-90 minutes) was reviewed by the attending PCI physician site or if the attendant annotations and QA data was generated solely by the device software.

Variable	Type	Caption	List Options	Abstraction Instruction
			1. yes<br>2. unknown	0=no<br>1=yes<br>2=unknown<br><br>The goal is for sites to review the recordings so as to ''correct'' any oversights by the software.<br>Source = Patient follow up information / PCI/Hospital chart
p_pcipfedecgd	dropdown	Posttreatment ECG (60-90 minutes) Data Exists	listid: ny<br><br>0. no<br>1. yes	Is there the posttretment PCI ECG (60-90 minutes) data available on request?<br><br>0=no<br>1=yes<br><br>Source = Patient follow up information / PCI/Hospital chart
p_pcipfedecgdgca	dropdown	Posttreatment ECG (60-90 minutes) Diagnosis - Computer Assisted	listid: ny<br><br>0. no<br>1. yes	Indicate whether or not there was computer assisted diagnosis for the posttretment PCI ECG (60-90 minutes).<br><br>0=no<br>1=yes<br><br>Source = Patient follow up information / PCI/Hospital chart
p_pcipfedecgdgsp	textbox	Posttreatment ECG (60-90 minutes) Diagnosis - Computer Assisted - Specify		Please specify computer assisted diagnosis for the posttretment PCI ECG (60-90 minutes).<br><br>Value = text<br><br>Source = Patient follow up information / PCI/Hospital chart
p_pcipfedecgdgsp_2	textbox	Posttreatment ECG (60-90 minutes) Diagnosis - Computer Assisted - Specify 2 (cont)		
p_pcipfedecgdgsp_3	textbox	Posttreatment ECG (60-90 minutes) Diagnosis - Computer Assisted - Specify 3 (cont)		
p_pcipfedecgdg	textbox	Posttreatment ECG (60-90 minutes) Diagnosis 1		Indicate the diagnosis based on patient posttretment PCI ECG (60-90 minutes) made by PCI attending physician.<br><br>Source = Patient follow up information / PCI/Hospital chart
p_pcipfedecgdg_2	textbox	Posttreatment ECG (60-90 minutes) Diagnosis 2 (cont)		
p_pcipfedecgdg_3	textbox	test		
p_pcistepfedecg	dropdown	ST Elevation	listid: nyn<br><br>0. no	Is there ST elevation on patient posttretment PCI ECG (60-90 minutes)?

Variable	Type	Caption	List Options	Abstraction Instruction
			1. yes<br>2. not noted	0=no<br>1=yes<br><br>Source = Patient follow up information / PCI/Hospital chart
p_pcistemicrpfedecg	dropdown	STEMI Criteria Met	listid: nynr<br><br>0. no<br>1. yes<br>2. not recorded	Is ST Elevation Myocardial Infarction (STEMI) criteria met on patient posttretment PCI ECG (60-90 minutes)?<br><br>0=no<br>1=yes<br><br>Source = Patient follow up information / PCI/Hospital chart
p_pcistemilpfedecg	textbox	STEMI Location		Indicate, if possible the STEMI location based on patient posttretment PCI ECG (60-90 minutes).<br><br>Source = Patient follow up information / PCI/Hospital chart
p_pcipfredcp	dropdown	Reduction in the chest pain intensity	listid: nyn<br><br>0. no<br>1. yes<br>2. not noted	Indicate, if possible, if there is a significant reduction in the patient's chest pain intensity during the posttretment (60-90 min) period.<br><br>0=no<br>1=yes<br>2=not noted<br><br>Source = Patient follow up information / PCI/Hospital chart
p_pcipfredstemi	dropdown	Reduction in ST-segment elevation (i.e. >50%)	listid: nyn<br><br>0. no<br>1. yes<br>2. not noted	Indicate, if possible, if there is a significant reduction in ST-segment elevation (i.e. > 50%) during the posttretment (60-90 min) period.<br><br>0=no<br>1=yes<br>2=not noted<br><br>Source = Patient follow up information / PCI/Hospital chart
p_pf2pciecg_div	div	Posttreatment PCI ECG (6 hours after fibrinolysis/PCI)		
p_pf2pciecg	dropdown	Does Posttreatment PCI ECG Recording Exist (6 hours)?	listid: ny<br><br>0. no<br>1. yes	Did PCI personnel record the patient posttretment ECG 60-90 minutes after tretment received in PCI?<br><br>0=no<br><br>1=yes

PREDICT - Hospital Variables

Variable	Type	Caption	List Options	Abstraction Instruction
				Indicate no or yes, whether posttretment (60-90 min) PCI ECG recording exists.<br><br>Source = Patient follow up information / PCI/Hospital chart
p_pf2pciecgdt	textbox	Date		What was the date of the patient posttretment ECG (60-90 minutes) in PCI?<br><br>Date value = yyyy/mm/dd<br><br>Source = Patient follow up information / PCI/Hospital chart
p_pf2pciecgtm	textbox	Time		What was the time of the patient posttretment ECG (60-90 minutes) in PCI?<br><br>Numerical value based on 24 hour clock<br><br>00:00:00 – hour:min:sec<br><br>If no value for seconds data available – do not do not use value 00<br><br>Source = Patient follow up information / PCI/Hospital chart
p_pf2pciecgrev	dropdown	Posttreatment ECG (6 hours) Reviewed	listid: nyu<br><br>0. no<br>1. yes<br>2. unknown	Indicate whether or not the posttretment PCI ECG (6 hours) was reviewed by the attending PCI physician site or if the attendant annotations and QA data was generated solely by the device software.<br><br>0=no<br>1=yes<br>2=unknown<br><br>The goal is for sites to review the recordings so as to ''correct'' any oversights by the software.<br>Source = Patient follow up information / PCI/Hospital chart
p_pf2pciecgd	dropdown	Posttreatment ECG (6 hours) Data Exists	listid: ny<br><br>0. no<br>1. yes	Is there the posttretment PCI ECG (6 hours) data available on request?<br><br>0=no<br>1=yes<br><br>Source = Patient follow up information / PCI/Hospital chart

Variable	Type	Caption	List Options	Abstraction Instruction
p_pf2pciecgdgca	dropdown	Posttreatment ECG (6 hours) Diagnosis - Computer Assisted	listid: ny<br><br>0. no<br>1. yes	Indicate whether or not there was computer assisted diagnosis for the posttretment PCI ECG (6 hours).<br><br>0=no<br>1=yes<br><br>Source = Patient follow up information / PCI/Hospital chart
p_pf2pciecgdgsp	textbox	Posttreatment ECG (6 hours) Diagnosis - Computer Assisted - Specify 1		Please specify computer assisted diagnosis for the posttretment PCI ECG (6 hours).<br><br>Value = text<br><br>Source = Patient follow up information / PCI/Hospital chart
p_pf2pciecgdgsp_2	textbox	Posttreatment ECG (6 hours) Diagnosis - Computer Assisted - Specify 2 (cont)		
p_pf2pciecgdgsp_3	textbox	Posttreatment ECG (6 hours) Diagnosis - Computer Assisted - Specify 3 (cont)		
p_pf2pciecgdg	textbox	Posttreatment ECG (6 hours) Diagnosis 1		Indicate the diagnosis based on patient posttretment PCI ECG (6 hours) made by PCI attending physician.<br><br>Source = Patient follow up information / PCI/Hospital chart
p_pf2pciecgdg_2	textbox	Posttreatment ECG (6 hours) Diagnosis 2 (cont)		
p_pf2pciecgdg_3	textbox	Posttreatment ECG (6 hours) Diagnosis 3 (cont)		
p_stepf2pciecg	dropdown	ST Elevation	listid: nyn<br><br>0. no<br>1. yes<br>2. not noted	Is there ST elevation on patient posttretment PCI ECG (6 hours)?<br><br>0=no<br>1=yes<br><br>Source = Patient follow up information / PCI/Hospital chart
p_stemicrpf2pciecg	dropdown	STEMI Criteria Met	listid: nynr<br><br>0. no<br>1. yes<br>2. not recorded	Is ST Elevation Myocardial Infarction (STEMI) criteria met on patient posttretment PCI ECG (6 hours)?<br><br>0=no<br><br>1=yes<br><br>2=not noted

PREDICT - Hospital Variables

Variable	Type	Caption	List Options	Abstraction Instruction
				Source = Patient follow up information / PCI/Hospital chart
p_stemilpf2pciecg	textbox	STEMI Location		Indicate, if possible the STEMI location based on patient posttretment PCI ECG (6 hours).<br><br>Source = Patient follow up information / PCI/Hospital chart
p_pfp2ciredcp	dropdown	Reduction in the chest pain intensity	listid: nyn<br><br>0. no<br>1. yes<br>2. not noted	Indicate, if possible, if there is a significant reduction in the patient's chest pain intensity during the posttretment period (6 hours).<br><br>0=no<br>1=yes<br>2=not noted<br><br>Source = Patient follow up information / PCI/Hospital chart
p_pfp2ciredstemi	dropdown	Reduction in ST-segment elevation (i.e. > 50%)	listid: nyn<br><br>0. no<br>1. yes<br>2. not noted	Indicate, if possible, if there is a significant reduction in ST-segment elevation (i.e. > 50%) during the posttretment period (6 hours).<br><br>0=no<br>1=yes<br>2=not noted<br><br>Source = Patient follow up information / PCI/Hospital chart
p_pciangint_div	div	Angiography/Intervention		
p_pciangint	dropdown	Procedure Type	listid: angint<br><br>0. Elective<br>1. Urgent<br>2. Rescue<br>3. Primary<br>4. Facilitated<br>5. Salvage<br>6. Lab (Cath)<br>7. Cardiac Shock	Indicate the procedure type that was performed during the PCI course of patient care.<br><br>1= Elective<br>2= Urgent<br>3= Rescue<br>4= Primary<br>5= Facilitated<br>6= Salvage<br>7= Lab (Cath)<br>8= Cardiac Shock<br><br>Source = Patient follow up information / PCI/Hospital chart
p_pcidgcath	dropdown	Diagnostic Catheterization Performed	listid: nyn<br><br>0. no<br>1. yes	Indicate, if diagnostic catheterization was performed during the PCI course of patient care.<br><br>0=no

Variable	Type	Caption	List Options	Abstraction Instruction
			2. not noted	1=yes<br>2=not noted<br><br>Source = Patient follow up information / PCI/Hospital chart
p_pcidgcathdt	textbox	Diagnostic Catheterization Date		What was the date of the patient diagnostic catheterization?<br><br>Date value = yyyy/mm/dd<br><br>Source = Patient follow up information / PCI/Hospital chart
p_pcidgcathtm	textbox	Diagnostic Catheterization Time		What was the time of the patient diagnostic catheterization?<br><br>Numerical value based on 24 hour clock<br><br>00:00:00 – hour:min:sec<br><br>If no value for seconds data available – do not do not use value 00<br><br>Source = Patient follow up information / PCI/Hospital chart
p_pciira	dropdown	Infarct Related Artery (IRA)	listid: nyn<br><br>0. no<br>1. yes<br>2. not noted	Indicate if the infarct related artery was determined during the PCI course of patient care.<br>0=no<br>1=yes<br>2=not noted<br><br>Source = Patient follow up information / PCI/Hospital chart
p_pciiralm	dropdown	Infarct Related Artery (IRA) - Left Main (LM) Coronary Artery	listid: ny<br><br>0. no<br>1. yes	Indicate if the infarct related artery determined during the PCI course of patient care was left main coronary artery.<br><br>0=no<br>1=yes<br><br>Source = Patient follow up information / PCI/Hospital chart
p_pciiralad	dropdown	Infarct Related Artery (IRA) - Left Anterior Descendent (LAD) Coronary Artery	listid: ny<br><br>0. no<br>1. yes	Indicate if the infarct related artery determined during the PCI course of patient care was left anterior descedent coronary artery.<br><br>0=no<br>1=yes<br><br>Source = Patient follow up information / PCI/Hospital chart

Variable	Type	Caption	List Options	Abstraction Instruction
p_pciiralcx	dropdown	Infarct Related Artery (IRA) - Left Circumflex (LCX) Coronary Artery	listid: ny<br><br>0. no<br>1. yes	Indicate if the infarct related artery determined during the PCI course of patient care was left circumflex coronary artery.<br><br>0=no<br>1=yes<br><br>Source = Patient follow up information / PCI/Hospital chart
p_pciirarca	dropdown	Infarct Related Artery (IRA) - Right Coronary Artery (RCA)	listid: ny<br><br>0. no<br>1. yes	Indicate if the infarct related artery determined during the PCI course of patient care was right coronary artery.<br><br>0=no<br>1=yes<br><br>Source = Patient follow up information / PCI/Hospital chart
p_pciiraunknown	dropdown	Infarct Related Artery (IRA) - Unknown	listid: ny<br><br>0. no<br>1. yes	Indicate if the infarct related artery was not determined during the PCI course of patient care (uknown).<br><br>0=no<br>1=yes<br><br>Source = Patient follow up information / PCI/Hospital chart
p_pciiranca	dropdown	Infarct Related Artery (IRA) - Normal Coronary Arteries	listid: ny<br><br>0. no<br>1. yes	Indicate if during the PCI course of patient care it was determined that the coronary arteries were normal.<br><br>0=no<br>1=yes<br><br>Source = Patient follow up information / PCI/Hospital chart
p_pcimstirapre	dropdown	Maximum Stenosis for IRA Preintervention	listid: nyn<br><br>0. no<br>1. yes<br>2. not noted	Indicate if the maximum stenosis for IRA before the intervention was determined during the PCI course of patient care.<br><br>0=no<br>1=yes<br>2=not noted<br><br>Source = Patient follow up information / PCI/Hospital chart
p_pcimstiraprelm	textbox	Maximum Stenosis for LM Preintervention		Indicate the maximum stenosis for LM artery before the intervention determined during the PCI course of patient care.

PREDICT - Hospital Variables

Variable	Type	Caption	List Options	Abstraction Instruction
				Source = Patient follow up information / PCI/Hospital chart
p_pcimstir aprelad	textbox	Maximum Stenosis for LAD Preintervention		Indicate the maximum stenosis for LAD artery before the intervention determined during the PCI course of patient care.<br><br>Source = Patient follow up information / PCI/Hospital chart
p_pcimstir aprelcx	textbox	Maximum Stenosis for LCX Preintervention		
p_pcimstir aprerca	textbox	Maximum Stenosis for RCA Preintervention		
p_pcimstir apost	dropdown	Maximum Stenosis for IRA Postintervention	listid: nyn<br><br>0. no<br>1. yes<br>2. not noted	Indicate if the maximum stenosis for IRA after the intervention was determined during the PCI course of patient care.<br><br>0=no<br>1=yes<br>2=not noted<br><br>Source = Patient follow up information / PCI/Hospital chart
p_pcimstir apostlm	textbox	Maximum Stenosis for LM Postintervention		
p_pcimstir apostlad	textbox	Maximum Stenosis for LAD Postintervention		
p_pcimstir apostlcx	textbox	Maximum Stenosis for LCX Postintervention		
p_pcimstir apostrca	textbox	Maximum Stenosis for RCA Post intervention		
p_pcitimip re	dropdown	TIMI Grade Preintervention	listid: timipint<br><br>0. 0<br>1. 1<br>2. 2<br>3. 3<br>4. Not Noted	Indicate the TIMI grade measured before the intervention during the PCI course of patient care.<br><br>0= 0<br>1= 1<br>2= 2<br>3= 3<br>4= Not noted<br><br>Source = Patient follow up information / PCI/Hospital chart
p_pcitimip ost	dropdown	TIMI Grade Postintervention	listid: timipint	Indicate the TIMI grade measured after the intervention during the PCI course of patient care.

Variable	Type	Caption	List Options	Abstraction Instruction
			0. 0<br>1. 1<br>2. 2<br>3. 3<br>4. Not Noted	0= 0<br>1= 1<br>2= 2<br>3= 3<br>4= Not noted<br><br>Source = Patient follow up information / PCI/Hospital chart
p_pcilvef	dropdown	Left Ventricle Ejection Fraction	listid: lvef<br><br>0. >50%<br>1. 35-50%<br>2. 20-34%<br>3. <20%<br>4. Note Noted	Indicate the left ventricle ejection fraction measured during the PCI course of patient care.<br><br>1= >50% (normal)<br>2= 35-50% (mild)<br>3= 20-34% (moderate)<br>4= < 20% (severe)<br>5= Not noted<br><br>Source = Patient follow up information / PCI/Hospital chart
p_pciperf	dropdown	PCI Performed	listid: nyn<br><br>0. no<br>1. yes<br>2. not noted	Indicate if PCI intervention was performed during the PCI course of patient care.<br><br>0=no<br>1=yes<br>2=not noted<br><br>Source = Patient follow up information / PCI/Hospital chart
p_pciinel	dropdown	PCI Intervention Ineligible	listid: nyn<br><br>0. no<br>1. yes<br>2. not noted	Indicate if patient was ineligible for PCI intervention?<br><br>0=no<br>1=yes<br>2=not noted<br><br>Source = Patient follow up information / PCI/Hospital chart
p_pciinelspec	textbox	Specify the Reason		Specify the reason why patient was ineligible for PCI intervention.<br><br>Source = Patient follow up information / PCI/Hospital chart
p_pciinterdt	textbox	PCI Intervention Date		What was the date of the patient PCI intervention?<br><br>Date value = yyyy/mm/dd<br><br>Source = Patient follow up information / PCI/Hospital chart
p_pciintertm	textbox	PCI Intervention Time		What was the time of the patient PCI intervention?

PREDICT - Hospital Variables

Variable	Type	Caption	List Options	Abstraction Instruction
				Numerical value based on 24 hour clock<br><br>00:00:00 – hour:min:sec<br><br>If no value for seconds data available – do not do not use value 00<br><br>Source = Patient follow up information / PCI/Hospital chart
p_pciinter	dropdown	Intervention	listid: nyn<br><br>0. no<br>1. yes<br>2. not noted	Indicate if any intervention from the list below was performed during the PCI course of patient care.<br><br>0=no<br>1=yes<br>2=not noted<br><br>List of intervention:<br>BA (balloon angioplasty)<br>DA (directional atherectomy)<br>RA (rotational atherectomy)<br>TECA (TEC atherectomy)<br>TE (trombectomy0<br>DP (distal protection)<br>Stent<br>GC (guiding catheters)<br>IVUS<br>Flow wire<br>Pressure wire<br>Filter wire<br><br>Source = Patient follow up information / PCI/Hospital chart
p_pciinter ba	dropdown	Balloon Angioplasty (BA)	listid: ny<br><br>0. no<br>1. yes	Indicate if balloon angioplasty (BA) was performed during the PCI course of patient care.<br><br>0=no<br>1=yes<br><br>Source = Patient follow up information / PCI/Hospital chart
p_pciinter da	dropdown	Directional Atherectomy (DA)	listid: ny<br><br>0. no<br>1. yes	Indicate if directional atherectomy (DA) was performed during the PCI course of patient care.<br><br>0=no<br>1=yes<br><br>Source = Patient follow up information / PCI/Hospital chart
p_pciinter ra	dropdown	Rotational Atherectomy (RA)	listid: ny<br><br>0. no	Indicate if rotational atherectomy (RA) was performed during the PCI course of patient care.

Variable	Type	Caption	List Options	Abstraction Instruction
			1. yes	0=no<br>1=yes<br><br>Source = Patient follow up information / PCI/Hospital chart
p_pciinter teca	dropdown	TEC Atherectomy (TECA)	listid: ny<br><br>0. no<br>1. yes	Indicate if TEC atherectomy (TECA) was performed during the PCI course of patient care.<br><br>0=no<br>1=yes<br><br>Source = Patient follow up information / PCI/Hospital chart
p_pciinter te	dropdown	Trombectomy (TE)	listid: ny<br><br>0. no<br>1. yes	Indicate if trombectomy (TE) was performed during the PCI course of patient care.<br><br>0=no<br>1=yes<br><br>Source = Patient follow up information / PCI/Hospital chart
p_pciinter dp	dropdown	Distal Protection (DP)	listid: ny<br><br>0. no<br>1. yes	Indicate if balloon angioplasty (BA) was performed during the PCI course of patient care.<br><br>0=no<br>1=yes<br><br>Source = Patient follow up information / PCI/Hospital chart
p_pciinter si	dropdown	Stent Intervention	listid: ny<br><br>0. no<br>1. yes	Indicate if stent intervention was performed during the PCI course of patient care.<br><br>0=no<br>1=yes<br><br>Source = Patient follow up information / PCI/Hospital chart
p_pciinter gc	dropdown	Guiding Catheters (GC) Intervention	listid: ny<br><br>0. no<br>1. yes	Indicate if guiding catheters (GC) intervention was performed during the PCI course of patient care.<br><br>0=no<br>1=yes<br><br>Source = Patient follow up information / PCI/Hospital chart
p_pciinter ivus	dropdown	IVUS Intervention	listid: ny<br><br>0. no<br>1. yes	Indicate if IVUS intervention was performed during the PCI course of patient care.<br><br>0=no<br>1=yes

PREDICT - Hospital Variables

Variable	Type	Caption	List Options	Abstraction Instruction
				Source = Patient follow up information / PCI/Hospital chart
p_pciinterflwi	dropdown	Flow Wire Intervention	listid: ny<br><br>0. no<br>1. yes	Indicate if flow wire intervention was performed during the PCI course of patient care.<br><br>0=no<br>1=yes<br><br>Source = Patient follow up information / PCI/Hospital chart
p_pciinterpwi	dropdown	Pressure Wire Intervention	listid: ny<br><br>0. no<br>1. yes	Indicate if pressure wire intervention was performed during the PCI course of patient care.<br><br>0=no<br>1=yes<br><br>Source = Patient follow up information / PCI/Hospital chart
p_pciinterfwi	dropdown	Filter Wire Intervention	listid: ny<br><br>0. no<br>1. yes	Indicate if filter wire intervention was performed during the PCI course of patient care.<br><br>0=no<br>1=yes<br><br>Source = Patient follow up information / PCI/Hospital chart
p_pcibalnum	textbox	Balloon number		Indicate the number of balloons used for the intervention during the PCI course of patient care.<br><br>Source = Patient follow up information / PCI/Hospital chart
p_pcistentbare	dropdown	Bare Stent Used	listid: nyn<br><br>0. no<br>1. yes<br>2. not noted	Indicate if the bare stent was used for the intervention during the PCI course of patient care.<br><br>0=no<br>1=yes<br>2=not noted<br><br>Source = Patient follow up information / PCI/Hospital chart
p_pcistentbarenum	textbox	Bare Stent Number		Indicate the number of bare stents used for the intervention during the PCI course of patient care.<br><br>Source = Patient follow up information / PCI/Hospital chart
p_pcistentcoat	dropdown	Coated Stent Used	listid: nyn<br><br>0. no<br>1. yes	Indicate if the coated stent was used for the intervention during the PCI course of patient care.<br><br>0=no

Variable	Type	Caption	List Options	Abstraction Instruction
			2. not noted	1=yes<br>2=not noted<br><br>Source = Patient follow up information / PCI/Hospital chart
p_pcistentcoatnum	textbox	Coated Stent Number		Indicate the number of coated stents used for the intervention during the PCI course of patient care.<br><br>Source = Patient follow up information / PCI/Hospital chart
p_pcicdinter	dropdown	Complications During Intervention	listid: nyn<br><br>0. no<br>1. yes<br>2. not noted	Indicate if any complication from the list below occurred during the intervention performed during the PCI course of patient care.<br><br>List of complications:<br>Abrupt closure<br>Major dissection<br>Minor dissection<br>No reflow / distal embolisation<br>Perforation<br>Extravascular staining<br>Other<br><br>Source = Patient follow up information / PCI/Hospital chart
p_pcicdinterac	dropdown	Abrupt Closure	listid: ny<br><br>0. no<br>1. yes	Indicate if abrupt closure occurred during the intervention performed during the PCI course of patient care.<br><br>0=no<br>1=yes<br><br>Source = Patient follow up information / PCI/Hospital chart
p_pcicdintermajd	dropdown	Major Dissection	listid: ny<br><br>0. no<br>1. yes	Indicate if major dissection occurred during the intervention performed during the PCI course of patient care.<br><br>0=no<br>1=yes<br><br>Source = Patient follow up information / PCI/Hospital chart
p_pcicdintermind	dropdown	Minor Dissection	listid: ny<br><br>0. no<br>1. yes	Indicate if minor dissection occurred during the intervention performed during the PCI course of patient care.<br><br>0=no<br>1=yes<br><br>Source = Patient follow up information / PCI/Hospital chart

PREDICT - Hospital Variables

Variable	Type	Caption	List Options	Abstraction Instruction
p_pcicdinternrde	dropdown	No Reflow / Distal Embolisation	listid: ny<br><br>0. no<br>1. yes	Indicate if no reflow / distal embolisation occurred during the intervention performed during the PCI course of patient care.<br><br>0=no<br>1=yes<br><br>Source = Patient follow up information / PCI/Hospital chart
p_pcicdinterper	dropdown	Perforation	listid: ny<br><br>0. no<br>1. yes	Indicate if perforation occurred during the intervention performed during the PCI course of patient care.<br><br>0=no<br>1=yes<br><br>Source = Patient follow up information / PCI/Hospital chart
p_pcicdinterexst	dropdown	Extravascular Staining	listid: ny<br><br>0. no<br>1. yes	Indicate if extravascular staining occurred during the intervention performed during the PCI course of patient care.<br><br>0=no<br>1=yes<br><br>Source = Patient follow up information / PCI/Hospital chart
p_pcicdinteroth	dropdown	Other	listid: ny<br><br>0. no<br>1. yes	Indicate if any other complication occurred during the intervention performed during the PCI course of patient care.<br><br>0=no<br>1=yes<br><br>Source = Patient follow up information / PCI/Hospital chart
p_pcicdinteroths	textbox	Specify		Please specify what other complication occurred during the intervention performed during the PCI course of patient care.<br><br>Source = Patient follow up information / PCI/Hospital chart
p_pcivasc	dropdown	Vascular Access Site Complication	listid: nyn<br><br>0. no<br>1. yes<br>2. not noted	Indicate if any vascular access site complication from the list below occurred during the intervention performed during the PCI course of patient care.<br><br>List of complications:<br><br>Hematoma<br>Pseudoaneurysm

PREDICT - Hospital Variables

Variable	Type	Caption	List Options	Abstraction Instruction
				Arteriovenous fistula<br>Severe limb ischemia<br>Access site rebleed<br>Other<br><br>Source = Patient follow up information / PCI/Hospital chart
p_pcivaschem	dropdown	Hematoma	listid: ny<br><br>0. no<br>1. yes	Indicate if hematoma occurred during the intervention performed during the PCI course of patient care.<br><br>0=no<br>1=yes<br><br>Source = Patient follow up information / PCI/Hospital chart
p_pcivascpa	dropdown	Pseudoaneurysm	listid: ny<br><br>0. no<br>1. yes	Indicate if pseudoaneurysm occurred during the intervention performed during the PCI course of patient care.<br><br>0=no<br>1=yes<br><br>Source = Patient follow up information / PCI/Hospital chart
p_pcivascavf	dropdown	Arteriovenous Fistula	listid: ny<br><br>0. no<br>1. yes	Indicate if arteriovenous fistula occurred during the intervention performed during the PCI course of patient care.<br><br>0=no<br>1=yes<br><br>Source = Patient follow up information / PCI/Hospital chart
p_pcivascsli	dropdown	Severe Limb Ischemia	listid: ny<br><br>0. no<br>1. yes	Indicate if severe limb ischemia occurred during the intervention performed during the PCI course of patient care.<br><br>0=no<br>1=yes<br><br>Source = Patient follow up information / PCI/Hospital chart
p_pcivascasr	dropdown	Access Site Rebleed	listid: ny<br><br>0. no<br>1. yes	Indicate if access site rebleed occurred during the intervention performed during the PCI course of patient care.<br><br>0=no<br>1=yes<br><br>Source = Patient follow up information / PCI/Hospital chart

Variable	Type	Caption	List Options	Abstraction Instruction
p_pcivascoth	dropdown	Other	listid: ny<br>0. no<br>1. yes	Indicate if any other vascular access site complication occurred during the intervention performed during the PCI course of patient care.<br>0=no<br>1=yes<br>Source = Patient follow up information / PCI/Hospital chart
p_pcivascoths	textbox	Specify		Please specify what other vascular access site complication occurred during the intervention performed during the PCI course of patient care.<br>Source = Patient follow up information / PCI/Hospital chart
p_pciiabp	dropdown	Intra-aortic Balloon Pump (IABP)	listid: nyn<br>0. no<br>1. yes<br>2. not noted	Indicate if the itra-aortic balloon pump was inserted at any time during the PCI course of patient care.<br>0=no<br>1=yes<br>2=not noted<br>Source = Patient follow up information / PCI/Hospital chart
p_pciiabpdt	textbox	Intra-aortic Balloon Pump (IABP) Date		What was the date of the patient IABP intervention?<br>Date value = yyyy/mm/dd<br>Source = Patient follow up information / PCI/Hospital chart
p_pciiabptm	textbox	Intra-aortic Balloon Pump (IABP) Time		What was the time of the patient IABP intervention?<br>Numerical value based on 24 hour clock<br>00:00:00 – hour:min:sec<br>If no value for seconds data available – do not do not use value 00<br>Source = Patient follow up information / PCI/Hospital chart
p_pciclinev	dropdown	Clinical Events Occured in PCI Centre	listid: nyn<br>0. no<br>1. yes<br>2. not noted	Indicate if any clinical events from the list below occurred during the PCI course of patient care.<br>List of clinical events:<br>Death<br>Cardiogenic shock<br>New of worsening CHF<br>Recurrent ischemia<br>Myocardial reinfarction

PREDICT - Hospital Variables

Variable	Type	Caption	List Options	Abstraction Instruction
				Suspected TIA/Stroke<br>Revascularization<br>Bleeding<br>Transfusion<br>Temporary pacemaker<br>Cardiac arrest<br>Other<br><br>Source = Patient follow up information / PCI/Hospital chart
p_pcicline vdth	dropdown	Death	listid: ny<br><br>0. no<br>1. yes	Indicate if patient died during the PCI course of patient care.<br><br>0=no<br>1=yes<br><br>Source = Patient follow up information / PCI/Hospital chart
p_pcicline vdthdt	textbox	Death Date		What was the date of the patient's death in PCI centre?<br><br>Date value = yyyy/mm/dd<br><br>Source = Patient follow up information / PCI/Hospital chart
p_pcicline vdthtm	textbox	Deat Time		What was the time of the patient's death in PCI centre?<br><br>Numerical value based on 24 hour clock<br><br>00:00:00 – hour:min:sec<br><br>If no value for seconds data available – do not do not use value 00<br><br>Source = Patient follow up information / PCI/Hospital chart
p_pcicline vcs	dropdown	Cardiogenic Shock	listid: ny<br><br>0. no<br>1. yes	Indicate if patient had cardiogenic shock during the PCI course of patient care.<br><br>0=no<br>1=yes<br><br>Source = Patient follow up information / PCI/Hospital chart
p_pcicline vnwchf	dropdown	New or Worsening CHF	listid: ny<br><br>0. no<br>1. yes	Indicate if patient had new or worsening congestive heart failure (CHF) during the PCI course of patient care.<br><br>0=no<br>1=yes

PREDICT - Hospital Variables

Variable	Type	Caption	List Options	Abstraction Instruction
				Source = Patient follow up information / PCI/Hospital chart
p_pciclinevrisch	dropdown	Recurrent Ischemia	listid: ny<br><br>0. no<br>1. yes	Indicate if patient had recurrent ischemia during the PCI course of patient care.<br><br>0=no<br>1=yes<br><br>Source = Patient follow up information / PCI/Hospital chart
p_pciclinevmrein	dropdown	Myocardial Reinfarction	listid: ny<br><br>0. no<br>1. yes	Indicate if patient had myocardial reinfarction during the PCI course of patient care.<br><br>0=no<br>1=yes<br><br>Source = Patient follow up information / PCI/Hospital chart
p_pciclinevtiastr	dropdown	Suspected TIA/Stroke	listid: ny<br><br>0. no<br>1. yes	Indicate if patient had TIA/stroke during the PCI course of patient care.<br><br>0=no<br>1=yes<br><br>Source = Patient follow up information / PCI/Hospital chart
p_pciclinevrev	dropdown	Revascularization	listid: ny<br><br>0. no<br>1. yes	Indicate if patient had revascularization during the PCI course of patient care.<br><br>0=no<br>1=yes<br><br>Source = Patient follow up information / PCI/Hospital chart
p_pciclinevbleed	dropdown	Bleeding	listid: ny<br><br>0. no<br>1. yes	Indicate if patient had bleeding during the PCI course of patient care.<br><br>0=no<br>1=yes<br><br>Source = Patient follow up information / PCI/Hospital chart
p_pciclinevtransf	dropdown	Transfusion	listid: ny<br><br>0. no<br>1. yes	Indicate if patient had transfusion during the PCI course of patient care.<br><br>0=no<br>1=yes<br><br>Source = Patient follow up information / PCI/Hospital chart

PREDICT - Hospital Variables

Variable	Type	Caption	List Options	Abstraction Instruction
p_pciclinevtempac	dropdown	Temporary Pacemaker	listid: ny<br><br>0. no<br>1. yes	Indicate if patient had temporaty pacemaker inserted during the PCI course of patient care.<br><br>0=no<br>1=yes<br><br>Source = Patient follow up information / PCI/Hospital chart
p_pciclinevcararr	dropdown	Cardiac Arrest	listid: ny<br><br>0. no<br>1. yes	Indicate if patient had cardiac arrest during the PCI course of patient care.<br><br>0=no<br>1=yes<br><br>Source = Patient follow up information / PCI/Hospital chart
p_pciclinevoth	dropdown	Other	listid: ny<br><br>0. no<br>1. yes	Indicate if any other clinical events occurred during the PCI course of patient care.<br><br>0=no<br>1=yes<br><br>Source = Patient follow up information / PCI/Hospital chart
p_pciclinevoths	textbox	Specify		Please specify what other clinical event occurred during the PCI course of patient care.<br><br>Source = Patient follow up information / PCI/Hospital chart
p_pcimed	dropdown	Additional Medications Given in PCI Centre	listid: nyn<br><br>0. no<br>1. yes<br>2. not noted	Indicate what additional medications, if any, were administered during the PCI course of patient care.<br><br>0= None<br>1= Beta blocker<br>2= IV vasodilator (other than nitrates)<br><br>3= ACE inhibitor<br>4= ARB antagonist<br>5= Lipid-lowering agents<br>6= Diuretic<br>7= IV inotrope<br>8= Oral anticoagulant (e.g. coumadin/warfarin)<br>9= Other<br>10 = Not noted<br><br>Check all that apply.<br>Source = Patient follow up information / PCI/Hospital chart
p_pcimedbb	dropdown	Beta Blockers	listid: nynr	Indicate if beta blocker was administered to the patient duing the PCI course of care.

PREDICT - Hospital Variables

Variable	Type	Caption	List Options	Abstraction Instruction
			0. no<br>1. yes<br>2. not recorded	0=no<br>1=yes<br>2=not noted<br>Source = Patient follow up information / PCI/Hospital chart
p_pcimedivvas	dropdown	IV Vasodilator (Other Than Nitrates)	listid: nynr<br>0. no<br>1. yes<br>2. not recorded	Indicate if IV vasodilatoe (other than nitrates) was administered to the patient duing the PCI course of care.<br>0=no<br>1=yes<br>2=not noted<br>Source = Patient follow up information / PCI/Hospital chart
p_pcimedacei	dropdown	Angiotensin Converting Enzyme (ACE) Inhibitor	listid: nynr<br>0. no<br>1. yes<br>2. not recorded	Indicate if angiotensin converting enzyme (ACE) inhibitor was administered to the patient duing the PCI course of care.<br>0=no<br>1=yes<br>2=not noted<br>Source = Patient follow up information / PCI/Hospital chart
p_pcimedarb	dropdown	Angiotensin Receptor Blocker (ARB)	listid: nynr<br>0. no<br>1. yes<br>2. not recorded	Indicate if angiotensin receptor blocker (ARB) was administered to the patient duing the PCI course of care.<br>0=no

PREDICT - Hospital Variables

Variable	Type	Caption	List Options	Abstraction Instruction
				1=yes<br><br>2=not noted<br><br>Source = Patient follow up information / PCI/Hospital chart
p_pcimedlla	dropdown	Lipid Lowering Agent	listid: nynr<br><br>0. no<br>1. yes<br>2. not recorded	Indicate if lipid lowering agent was administered to the patient duing the PCI course of care.<br><br>0=no<br><br>1=yes<br><br>2=not noted<br><br>Source = Patient follow up information / PCI/Hospital chart
p_pcimeddiu	dropdown	Diuretic	listid: nynr<br><br>0. no<br>1. yes<br>2. not recorded	Indicate if diuteric was administered to the patient duing the PCI course of care.<br><br>0=no<br><br>1=yes<br><br>2=not noted<br><br>Source = Patient follow up information / PCI/Hospital chart
p_pcimedivino	dropdown	IV Inotrope	listid: nynr<br><br>0. no<br>1. yes<br>2. not recorded	Indicate if IV inotrope was administered to the patient duing the PCI course of care.<br><br>0=no<br><br>1=yes<br><br>2=not noted

Variable	Type	Caption	List Options	Abstraction Instruction
				Source = Patient follow up information / PCI/Hospital chart
p_pcimedoacoa	dropdown	Oral Anticoagulant (e.g. Coumadin/Warfarin)	listid: nynr<br>0. no<br>1. yes<br>2. not recorded	Indicate if oral anticoagulant (e.g. coumadin/warfarin) was administered to the patient duing the PCI course of care.<br>0=no<br>1=yes<br>2=not noted<br>Source = Patient follow up information / PCI/Hospital chart
p_pcimedoth	dropdown	Other	listid: nynr<br>0. no<br>1. yes<br>2. not recorded	Indicate if any other medication was administered to the patient duing the PCI course of care.<br>0=no<br>1=yes<br>2=not noted<br>Source = Patient follow up information / PCI/Hospital chart
p_pcimedoths	textbox	Specify		Please specify what other medication was administered to the patient duing the PCI course of care.<br>Source = Patient follow up information / PCI/Hospital chart
p_pcimhndet	dropdown	Not Determined	listid: nyn<br>0. no<br>1. yes<br>2. not noted	Patient Medication History: (from Patient follow up information / PCI/Hospital chart<br>The Patient follow up information / PCI/Hospital chart selection of not determined implies the PCI staff (nurse/resident/fellow/staff physician) did not ask as opposed to not noted which implies the abstractor could not find the information.
p_pcid	div	PCI Centre Disposition		

PREDICT - Hospital Variables

Variable	Type	Caption	List Options	Abstraction Instruction
p_pciddt	textbox	PCI Centre Departure Date		What was the date of the patient PCI centre departure?<br><br>Date value = yyyy/mm/dd<br><br>Source = Patient follow up information / PCI/Hospital chart
p_pcidtm	textbox	PCI Departure Time		What was the time of the patients PCI center departure?<br><br>00:00:00 – hour:min:sec<br><br>Source = Patient follow up information / PCI/Hospital chart
p_pcidhome	dropdown	Was Patient Discharged Home?	listid: ny<br><br>0. no<br>1. yes	After admission to PCI was patient then discharged home?<br><br>0=no<br><br>1=yes<br><br>Source = Patient follow up information / PCI/Hospital chart
p_pciptxferah	dropdown	Was Patient Transferred to Another Hospital?	listid: ny<br><br>0. no<br>1. yes	After admission to PCI was patient then transferred to another hospital?<br><br>0=no<br>1=yes<br><br>Source = Patient follow up information / PCI/Hospital chart
p_pcitxhosp	dropdown	Transfer Hospital Name	listid: p_hosp<br><br>** See list items in appendix	What is the name of the hospital patient was transferred to?<br><br>Source = Patient follow up information / PCI/Hospital chart<br><br>Pulldown menu = list of hospitals in database<br><br>Unknown Hospital = select when destination will never be known<br><br>NOTE;<br>Do not provide the names of nursing home, rehabilitation, or other non-acute care facilities.

Variable	Type	Caption	List Options	Abstraction Instruction
				Transfer to one of these three entities constitutes an 'hospital discharge, reclassification, or death''.
p_pciptxferrh	dropdown	Was Patient Transferred to Referring Hospital?	listid: ny<br>0. no<br>1. yes	After admission to PCI was patient then transferred back to referring hospital?<br>0=no<br>1=yes<br>Source = Patient follow up information / PCI/Hospital chart
p_pcitxrhosp	dropdown	Transfer Hospital Name	listid: p_hosp<br>** See list items in appendix	What is the name of the referring hospital patient was transferred to?<br>Source = Patient follow up information / PCI/Hospital chart<br>Pulldown menu = list of hospitals in database<br>Unknown Hospital = select when destination hospital will never be known.<br>NOTE;<br>Do not provide the names of nursing home, rehabilitation, or other non-acute care facilities.<br>Transfer to one of these three entities constitutes an 'hospital discharge, reclassification, or death''.
p_pcitrans	dropdown	Transport Mode	listid: trans<br>1. by land<br>2. by air	Where a patient has been transported by both land and air, select air.<br>Source = Patient follow up information / PCI/Hospital chart
p_pciccuicu_div	div	Location and Length of Stay in PCI Centre		
p_pciccuicu	dropdown	CCU/ICU	listid: ny<br>0. no<br>1. yes	Did patient stay at CCU/ICU during the PCI course of patient care.<br>0=no<br>1=yes

PREDICT - Hospital Variables

Variable	Type	Caption	List Options	Abstraction Instruction
				Source = Patient follow up information / PCI/Hospital chart
p_pciccuicudays	textbox	CCU/ICU Days		The total number of days patient stayed at CCU/ICU during the PCI course of patient care.<br><br>Source = Patient follow up information / PCI/Hospital chart
p_pcitelward	dropdown	Telemetry Ward	listid: ny<br>0. no<br>1. yes	Did patient stay at telemetry ward during the PCI course of patient care.<br><br>0=no<br>1=yes<br><br>Source = Patient follow up information / PCI/Hospital chart
p_pcitelwarddays	textbox	Telemetry Ward Days		The total number of days patient stayed at telemetry ward during the PCI course of patient care.<br><br>Source = Patient follow up information / PCI/Hospital chart
p_pciward	dropdown	Ward	listid: ny<br>0. no<br>1. yes	Did patient stay at ward during the PCI course of patient care.<br><br>0=no<br>1=yes<br><br>Source = Patient follow up information / PCI/Hospital chart
p_pciwarddays	textbox	Ward Days		The total number of days patient stayed at ward during the PCI course of patient care.<br><br>Source = Patient follow up information / PCI/Hospital chart
p_pcitotallos	textbox	Total Length of Stay (Total LOS) in Days		The total number of days patient stayed at PCI center.<br><br>Total = discharge – arrival<br><br>Source = Patient follow up information / PCI/Hospital chart
p_pciicd10	div	ICD 10 Codes		
p_pciicd10_1	textbox	1		
p_pciicd10_2	textbox	2		
p_pciicd10_3	textbox	3		
p_pciicd10_4	textbox	4		

PREDICT - Hospital Variables

Variable	Type	Caption	List Options	Abstraction Instruction
p_pciicd10_5	textbox	5		
p_pciicd10_6	textbox	6		
p_pciicd10_7	textbox	7		
p_pciicd10_8	textbox	8		
p_pciicd10_9	textbox	9		
p_pciicd10_10	textbox	10		
p_pciicd10_11	textbox	11		
p_pciicd10_12	textbox	12		
p_pciicd10_13	textbox	13		
p_pciicd10_14	textbox	14		
p_pciicd10_15	textbox	15		
p_pciicd10_16	textbox	16		
p_pcistatus	dropdown	PCI Form Status	listid: p_status<br>0. Incomplete<br>1. Complete<br>2. Pending Source Doc(s)<br>3. Not Required - Patient Deceased	
**p_inhform**	**section**	**Inhospital Form**		
p_hospital	dropdown	Was Patient Transferred to Hospital?	listid: ny<br>0. no<br>1. yes	
p_pihih	div	Patient Identifiers		
p_ilnameih	textbox	Surname		This field is used to obtain the Hospital chart version of the patient's surname.<br><br>Transcribe as-is.<br><br>Caps Lock
p_ifnameih	textbox	Given Name		This field is used to obtain the Hospital chart version of a patient's given name.<br><br>Transcribe as-is.<br><br>Caps Lock
p_iaddrih	textbox	Mailing Address		This field is used to obtain the Hospital chart version of a patient's mailing/street address.<br><br>Transcribe as-is.

Variable	Type	Caption	List Options	Abstraction Instruction
				Caps Lock
p_icityih	textbox	City/Town		This field is used to obtain the Hospital chart version of a patient's city / town.<br><br>Transcribe as-is.<br><br>Caps Lock
p_iprovih	dropdown	Province	listid: prov<br><br>1. AB<br>2. BC<br>3. MB<br>4. NB<br>5. NF<br>6. NS<br>7. NT<br>8. NU<br>9. ON<br>10. PE<br>11. QC<br>12. SK<br>13. YT<br>99. Out of Country	This field is used to obtain the Hospital chart version of a patient's province.<br><br>Transcribe as-is.<br><br>Caps Lock
p_ipostcih	textbox	Postal Code		This field is used to obtain the Hospital chart version of a patient's postal code.<br><br>Transcribe as-is.<br><br>Caps Lock
p_idobih	textbox	Date of Birth		This field is used to obtain the Hospital chart version of patient's date of birth (MDY).<br><br>Transcribe as-is.<br><br>Caps Lock
p_wghtih	dropdown	Weight	listid: uy<br><br>0. unknown/not noted<br><br>1. yes	A This field is used to obtain the Hospital chart version of a patient's weight<br><br>Transcribe as-is
p_wghtkgih	textbox	Weight(kg)		A This field is used to obtain the Hospital chart version of a patient's weight<br><br>Transcribe as-is
p_ihospregih	textbox	Registration Number/Patient Chart Number		Hospital chart number from Health Records Department (not from ACR).<br><br>Transcribe as-is.

PREDICT - Hospital Variables

Variable	Type	Caption	List Options	Abstraction Instruction
				Caps Lock
p_phospregih	textbox	Hospital Registration Number / Patient Chart Number		Hospital Registration Number<br><br>Transcribe as-is.<br><br>Caps Lock<br><br>Note this field will be not applicable for patients who are not transported to an ED or hospital.
p_ihealthcrd	div	Health Card		
p_ihealthcnih	textbox	Number		This field is used to obtain the Hospital chart version of Health Card No.<br><br>Transcribe as-is
p_ihealthcvih	textbox	Version Code		This field is used to obtain the Hospital chart version of Version No.<br><br>Transcribe as-is
p_isexih	dropdown	Gender	listid: sex<br>0. female<br>1. male<br>2. not noted	This field is used to obtain the Hospital chart version of Gender.<br><br>Transcribe as-is
p_iphone1ih	textbox	Phone 1 (Home)		Patient''s home telephone number.<br><br>Transcribe as-is
p_iphone2ih	textbox	Phone 2		Another contact number for the patient.<br><br>Transcribe as-is
p_ptrih	div	Hospital Episode/Time Record		
p_sodtih	textbox	Symptom Onset Date		What was the date of the patient symptom onset?<br><br>Date value = yyyy/mm/dd<br><br>Source = Patient follow up information / Hospital chart
p_sotmih	textbox	Symptom Onset Time		What was the time of the patient symptom onset?<br><br>Numerical value based on 24 hour clock<br><br>00:00:00 – hour:min:sec<br><br>If no value for seconds data available – do not do not use value 00.

PREDICT - Hospital Variables

Variable	Type	Caption	List Options	Abstraction Instruction
				Source = Patient follow up information /Hospital chart
p_ihardt	textbox	Hospital Arrival Date		What was the date of the patient hospital arrival?<br><br>Date value = yyyy/mm/dd<br><br>Source = Patient follow up information / Hospital chart
p_ihartm	textbox	Hospital Arrival Time		What was the time of the patient hospital arrival?<br><br>Numerical value based on 24 hour clock<br><br>00:00:00 – hour:min:sec<br><br>If no value for seconds data available – do not do not use value 00.<br><br>Source = Patient follow up information / Hospital chart
p_recihospih	dropdown	Receiving/Hospital	listid: p_hosp<br><br>** See list items in appendix	What is the name of the Receiving Hospital?<br><br>Source = Patient follow up information Hospital chart<br><br>Pulldown menu = list of hospitals in database<br><br>Unknown Hospital = select when destination will never be known.<br><br>NOTE;<br>Do not provide the names of nursing home, rehabilitation, or other non-acute care facilities.<br>Transfer to one of these three entities constitutes an ''hospital discharge, reclassification, or death''.
p_phxih	dropdown	Patient Past History	listid: nyn<br><br>0. no<br>1. yes<br><br>2. not noted	Past History: Patient follow up information / Hospital chart<br><br>If no past history is noted on the follow up information / Hospital chart, choose not noted.<br><br>HTNâ€”hypertension<br>Hyperlipidemia<br>Diabetes<br>Renal insufficiency/Failure<br>Chronic renal failure

Variable	Type	Caption	List Options	Abstraction Instruction
				Acute renal failure<br>Dialysis<br>Prior MIâ€"myocardial infarction<br>CADâ€"coronary artery disease<br>CHFâ€"congestive heart failure<br>Prior CABGâ€"coronary artery bypass graph<br>Prior PCI – percutaneous coronary intervention<br>ICDâ€"implantable cardiodefibrillator<br>Pacemaker<br>Peripheral vascular disease<br>DVT – deep venous trombosis<br>Tromboembolic history<br>Pulmonary embolism<br>Stroke/TIA/CVA<br>Smoking<br>Alcohol abuse<br>Cancer<br>Positive family history for heart disease
p_phihphlt	dropdown	Previously Healthy	listid: nyn<br>0. no<br>1. yes<br>2. not noted	Past History (from Patient follow up information / Hospital chart).<br>If no past history is noted on the Patient follow up information / Hospital chart, choose not noted.
p_phihhyp	dropdown	Hypertension	listid: nyn<br>0. no<br>1. yes<br>2. not noted	Past History (from Patient follow up information / Hospital chart).<br>If no past history is noted on the Patient follow up information / Hospital chart, choose not noted.
p_phihhl	dropdown	Hyperlipidemia	listid: nyn<br>0. no<br>1. yes<br>2. not noted	Past History (from Patient follow up information / Hospital chart).<br>If no past history is noted on the Patient follow up information / Hospital chart, choose not noted.
p_phihdia	dropdown	Diabetes	listid: nyn<br>0. no<br>1. yes<br>2. not noted	Past History (from Patient follow up information / Hospital chart).<br>If no past history is noted on the Patient follow up information / Hospital chart, choose not noted.
p_phihdiatype	dropdown	Diabetes Type	listid: diabtyp<br>0. Type 1	Past History (from Patient follow up information / Hospital chart).

Variable	Type	Caption	List Options	Abstraction Instruction
			1. Type 2	If no past history is noted on the Patient follow up information / Hospital chart, choose not noted.
p_phihrif	dropdown	Renal Insufficiency/Failure	listid: nyn<br>0. no<br>1. yes<br>2. not noted	Past History (from Patient follow up information / Hospital chart).<br>If no past history is noted on the Patient follow up information / Hospital chart, choose not noted.
p_phihcrf	dropdown	Chronic Renal Failure	listid: nyn<br>0. no<br>1. yes<br>2. not noted	Past History (from Patient follow up information / Hospital chart).<br>If no past history is noted on the Patient follow up information / Hospital chart, choose not noted.
p_phiharf	dropdown	Acute Renal Failure	listid: nyn<br>0. no<br>1. yes<br>2. not noted	Past History (from Patient follow up information / Hospital chart).<br>If no past history is noted on the Patient follow up information / Hospital chart, choose not noted.
p_phihdial	dropdown	Dialysis	listid: nyn<br>0. no<br>1. yes<br>2. not noted	Past History (from Patient follow up information / Hospital chart).<br>If no past history is noted on the Patient follow up information / Hospital chart, choose not noted.
p_phihcar	dropdown	Cardiac	listid: nyn<br>0. no<br>1. yes<br>2. not noted	Past History (from Patient follow up information / Hospital chart).<br>If no past history is noted on the Patient follow up information / Hospital chart, choose not noted.
p_phihmi	dropdown	Prior MI	listid: nyn<br>0. no<br>1. yes<br>2. not noted	Past History (from Patient follow up information / Hospital chart).<br>If no past history is noted on the Patient follow up information / Hospital chart, choose not noted.
p_phihmidt	textbox	Prior MI Date		What was the date of the patient prior MI?<br>Date value = yyyy/mm/dd<br>Source = Patient follow up information / PCI/Hospital chart
p_phihcad	dropdown	CAD	listid: nyn<br>0. no	Patient History (from Patient follow uo information / Hospital chart).

PREDICT - Hospital Variables

Variable	Type	Caption	List Options	Abstraction Instruction
			1. yes<br>2. not noted	If no past history is noted on the Patient follow up information / Hospital chart, choose not noted.
p_phihchf	dropdown	CHF	listid: nyn<br>0. no<br>1. yes<br>2. not noted	Patient History (from Patient follow uo information / Hospital chart).<br>If no past history is noted on the Patient follow up information / Hospital chart, choose not noted.
p_phihcabg	dropdown	Prior CABG	listid: nyn<br>0. no<br>1. yes<br>2. not noted	Patient History (from Patient follow uo information / Hospital chart).<br>If no past history is noted on the Patient follow up information / Hospital chart, choose not noted.
p_phihcabgdt	textbox	Prior CABG Date		What was the date of the patient prior CABG?<br>Date value = yyyy/mm/dd<br>Source = Patient follow up information / PCI/Hospital chart
p_phihpci	dropdown	Prior PCI	listid: nyn<br>0. no<br>1. yes<br>2. not noted	Past History (from Patient follow up information / Hospital chart).<br>If no past history is noted on the Patient follow up information / Hospital chart, choose not noted.
p_phihpcidt	textbox	Prior PCI Date		What was the date of the patient prior PCI?<br>Date value = yyyy/mm/dd<br>Source = Patient follow up information / PCI/Hospital chart
p_phihicd	dropdown	ICD	listid: nyn<br>0. no<br>1. yes<br>2. not noted	Past History (from Patient follow up information /PCI/Hospital chart).<br>If no past history is noted on the Patient follow up information / PCI/Hospital chart, choose not noted.
p_phihpac	dropdown	Pacemaker	listid: nyn<br>0. no<br>1. yes<br>2. not noted	Past History (from Patient follow up information /PCI/Hospital chart).<br>If no past history is noted on the Patient follow up information / PCI/Hospital chart, choose not noted.
p_phihpvd	dropdown	Peripheral Vascular Disease	listid: nyn<br>0. no	Past History (from Patient follow up information /PCI/Hospital chart).

Variable	Type	Caption	List Options	Abstraction Instruction
			1. yes<br>2. not noted	If no past history is noted on the Patient follow up information / PCI/Hospital chart, choose not noted.
p_phihdvt	dropdown	DVT	listid: nyn<br>0. no<br>1. yes<br>2. not noted	Past History (from Patient follow up information /PCI/Hospital chart).<br>If no past history is noted on the Patient follow up information / PCI/Hospital chart, choose not noted.
p_phihteh	dropdown	Tromboembolic History	listid: nyn<br>0. no<br>1. yes<br>2. not noted	Past History (from Patient follow up information /PCI/Hospital chart).<br>If no past history is noted on the Patient follow up information / PCI/Hospital chart, choose not noted.
p_phihpe	dropdown	Pulmonary Embolism	listid: nyn<br>0. no<br>1. yes<br>2. not noted	Past History (from Patient follow up information /PCI/Hospital chart).<br>If no past history is noted on the Patient follow up information / PCI/Hospital chart, choose not noted.
p_phihstr	dropdown	Stroke/TIA/CVA	listid: nyn<br>0. no<br>1. yes<br>2. not noted	Past History (from Patient follow up information /PCI/Hospital chart).<br>If no past history is noted on the Patient follow up information / PCI/Hospital chart, choose not noted.
p_phihstrdt	textbox	Prior Stroke/TIA/CVA Date		What was the date of the patient prior stroke/TIA/CVA?<br>Date value = yyyy/mm/dd<br>Source = Patient follow up information / PCI/Hospital chart
p_phihsmok	dropdown	Smoking	listid: nyn<br>0. no<br>1. yes<br>2. not noted	Past History (from Patient follow up information /PCI/Hospital chart).<br>If no past history is noted on the Patient follow up information / PCI/Hospital chart, choose not noted.
p_phihcsmok	dropdown	Current Smoker	listid: nyn<br>0. no<br>1. yes<br>2. not noted	Past History (from Patient follow up information /PCI/Hospital chart).<br>If no past history is noted on the Patient follow up information / PCI/Hospital chart, choose not noted.
p_phihaa	dropdown	Alcohol Abuse	listid: nyn<br>0. no	Past History (from Patient follow up information /PCI/Hospital chart).

PREDICT - Hospital Variables

Variable	Type	Caption	List Options	Abstraction Instruction
			1. yes<br>2. not noted	If no past history is noted on the Patient follow up information / PCI/Hospital chart, choose not noted.
p_phihcan	dropdown	Cancer	listid: nyn<br>0. no<br>1. yes<br>2. not noted	Past History (from Patient follow up information /PCI/Hospital chart).<br>If no past history is noted on the Patient follow up information / PCI/Hospital chart, choose not noted.
p_phihpfhhd	dropdown	Positive Family History for Heart Diseases	listid: nyn<br>0. no<br>1. yes<br>2. not noted	Past History (from Patient follow up information /PCI/Hospital chart).<br>If no past history is noted on the Patient follow up information / PCI/Hospital chart, choose not noted.
p_phihoth	dropdown	Other	listid: nyn<br>0. no<br>1. yes<br>2. not noted	Past History (from Patient follow up information / Hosipital chart)<br>If no past history is noted on the Patient follow up information / Hospital chart, choose not noted.
p_phihospc	textbox	Specify Other Not Listed		Past History (from Patient follow up information / Hospital chart)
p_mhxih	dropdown	Patient Past Medication History	listid: nyn<br>0. no<br>1. yes<br>2. not noted	Patient Medication History: Patient follow up information / Hospital chart<br>If no patient medication history is noted on the follow up information / Hospital chart, choose not noted.<br>Beta blockers<br>ASA – acetyl salicylic acid (Aspirin)<br>Fibrinolytics/Trombolytics<br>Clopidogrel (Plavix)/Ticlopidine<br>Glycoprotein (GP) IIb/IIIa inhibitors<br>Heparin<br>Low molecular weight heparin (LMWH)<br>Coumadin<br>Calcium channel blockers<br>Nitrates<br>ARB antagonists<br>ACE inhibitors<br>Lipid lowering drugs<br>Digitalis<br>Diuretics<br>Amiodarone<br>Insulin<br>Oral hypoglycemic agents<br>Mucomyst<br>Antidepressants

Variable	Type	Caption	List Options	Abstraction Instruction
				Anxiolytic (anti-anxiety)<br>Estrogens/OCP (oral contraceptive pills)<br>Immunosupressive medication<br>Bronchodilators/Inhaled steroids
p_mhihno	dropdown	None	listid: nyn<br>0. no<br>1. yes<br>2. not noted	Patient Medication History (Patient follow up information / Hospital chart).<br>If no patient medication history is noted on the Patient follow up information / Hospital chart, choose not noted.
p_mhihasa	dropdown	ASA (Aspirin)	listid: nyn<br>0. no<br>1. yes<br>2. not noted	Patient Medication History (Patient follow up information / Hospital chart).<br>If no patient medication history is noted on the Patient follow up information / Hospital chart, choose not noted.
p_mhihfib	dropdown	Fibrinolytics/Trombolytics	listid: nyn<br>0. no<br>1. yes<br>2. not noted	Patient Medication History (Patient follow up information / Hospital chart).<br>If no patient medication history is noted on the Patient follow up information / Hospital chart, choose not noted.
p_mhihfibtype	dropdown	Fibrinolytics/Trombolytics Type	listid: drug<br>1. Streptokinase<br>2. TPA<br>3. TNK<br>4. RPA	Patient Medication History (Patient follow up information / Hospital chart).<br>If no patient medication history is noted on the Patient follow up information / Hospital chart, choose not noted.
p_mhihclop	dropdown	Clopidogrel (Plavix)/Ticlopidine	listid: nyn<br>0. no<br>1. yes<br>2. not noted	Patient Medication History (Patient follow up information / Hospital chart).<br>If no patient medication history is noted on the Patient follow up information / Hospital chart, choose not noted.
p_mhihglycinh	dropdown	Glycoprotein (GP) llb/llla Inhibitors	listid: nyn<br>0. no	Patient Medication History (Patient follow up information / Hospital chart).

Variable	Type	Caption	List Options	Abstraction Instruction
			1. yes<br><br>2. not noted	If no patient medication history is noted on the Patient follow up information / Hospital chart, choose not noted.
p_mhihhep	dropdown	Heparin	listid: nyn<br><br>0. no<br>1. yes<br><br>2. not noted	Patient Medication History (Patient follow up information / Hospital chart).<br><br>If no patient medication history is noted on the Patient follow up information / Hospital chart, choose not noted.
p_mhihlmwh	dropdown	Low Molecular Weight Heparin (LMWH)	listid: nyn<br><br>0. no<br>1. yes<br><br>2. not noted	Patient Medication History (Patient follow up information / Hospital chart).<br><br>If no patient medication history is noted on the Patient follow up information / Hospital chart, choose not noted.
p_mhihbiv	dropdown	Bivalirudin (Angiomax)	listid: nyn<br><br>0. no<br>1. yes<br><br>2. not noted	Patient Medication History (Patient follow up information / Hospital chart).<br><br>If no patient medication history is noted on the Patient follow up information / Hospital chart, choose not noted.
p_mhihcoum	dropdown	Coumadin	listid: nyn<br><br>0. no<br>1. yes<br><br>2. not noted	Patient Medication History (Patient follow up information / Hospital chart).<br><br>If no patient medication history is noted on the Patient follow up information / Hospital chart, choose not noted.
p_mhihbb	dropdown	Beta Blockers	listid: nyn<br><br>0. no<br>1. yes<br><br>2. not noted	Patient Medication History (Patient follow up information / Hospital chart).<br><br>If no patient medication history is noted on the Patient follow up information / Hospital chart, choose not noted.
p_mhihccb	dropdown	Calcium Channel Blockers	listid: nyn<br><br>0. no<br>1. yes	Patient Medication History (Patient follow up information / Hospital chart).<br><br>If no patient medication history is noted on the Patient follow up information / Hospital chart, choose not noted.

PREDICT - Hospital Variables

Variable	Type	Caption	List Options	Abstraction Instruction
			2. not noted	
p_mhihlant	dropdown	Nitrates/IV/Top	listid: nyn<br>0. no<br>1. yes<br>2. not noted	Patient Medication History (Patient follow up information / Hospital chart).<br>If no patient medication history is noted on the Patient follow up information / Hospital chart, choose not noted.
p_mhiharba	dropdown	ARB Antagonists	listid: nyn<br>0. no<br>1. yes<br>2. not noted	Patient Medication History (Patient follow up information / Hospital chart).<br>If no patient medication history is noted on the Patient follow up information / Hospital chart, choose not noted.
p_mhihacei	dropdown	ACE Inhibitors	listid: nyn<br>0. no<br>1. yes<br>2. not noted	Patient Medication History (Patient follow up information / Hospital chart).<br>If no patient medication history is noted on the Patient follow up information / Hospital chart, choose not noted.
p_mhihlld	dropdown	Lipid Lowering Drugs	listid: nyn<br>0. no<br>1. yes<br>2. not noted	Patient Medication History (Patient follow up information / Hospital chart).<br>If no patient medication history is noted on the Patient follow up information / Hospital chart, choose not noted.
p_mhihdig	dropdown	Digitalis	listid: nyn<br>0. no<br>1. yes<br>2. not noted	Patient Medication History (Patient follow up information / Hospital chart).<br>If no patient medication history is noted on the Patient follow up information / Hospital chart, choose not noted.
p_mhihdiu	dropdown	Diuretics	listid: nyn<br>0. no<br>1. yes<br>2. not noted	Patient Medication History (Patient follow up information / Hospital chart).<br>If no patient medication history is noted on the Patient follow up information / Hospital chart, choose not noted.
p_mhihamio	dropdown	Amiodarone	listid: nyn	Patient Medication History (Patient follow up information / Hospital chart).

PREDICT - Hospital Variables

Variable	Type	Caption	List Options	Abstraction Instruction
			0. no<br>1. yes<br>2. not noted	If no patient medication history is noted on the Patient follow up information / Hospital chart, choose not noted.
p_mhihins	dropdown	Insulin	listid: nyn<br>0. no<br>1. yes<br>2. not noted	Patient Medication History (Patient follow up information / Hospital chart).<br>If no patient medication history is noted on the Patient follow up information / Hospital chart, choose not noted.
p_mhihoha	dropdown	Oral Hyoglycemic Agents	listid: nyn<br>0. no<br>1. yes<br>2. not noted	Patient Medication History (Patient follow up information / Hospital chart).<br>If no patient medication history is noted on the Patient follow up information / Hospital chart, choose not noted.
p_mhihmuc	dropdown	Mucomyst	listid: nyn<br>0. no<br>1. yes<br>2. not noted	Patient Medication History (Patient follow up information / Hospital chart).<br>If no patient medication history is noted on the Patient follow up information / Hospital chart, choose not noted.
p_mhihantidep	dropdown	Antidepressants	listid: nyn<br>0. no<br>1. yes<br>2. not noted	Patient Medication History (Patient follow up information / Hospital chart).<br>If no patient medication history is noted on the Patient follow up information / Hospital chart, choose not noted.
p_mhihantianx	dropdown	Anxiolytic (Anti-Anxiety)	listid: nyn<br>0. no<br>1. yes<br>2. not noted	Patient Medication History (Patient follow up information / Hospital chart).<br>If no patient medication history is noted on the Patient follow up information / Hospital chart, choose not noted.
p_mhihestocp	dropdown	Estrogens/OCP	listid: nyn<br>0. no	Patient Medication History (Patient follow up information / Hospital chart).

PREDICT - Hospital Variables

Variable	Type	Caption	List Options	Abstraction Instruction
			1. yes<br>2. not noted	If no patient medication history is noted on the Patient follow up information / Hospital chart, choose not noted.
p_mhihimmsup	dropdown	Immunosupressive Medication	listid: nyn<br>0. no<br>1. yes<br>2. not noted	Patient Medication History (Patient follow up information / Hospital chart).<br>If no patient medication history is noted on the Patient follow up information / Hospital chart, choose not noted.
p_mhihbis	dropdown	Bronchodilators/Inhaled Steroids	listid: nyn<br>0. no<br>1. yes<br>2. not noted	Patient Medication History (Patient follow up information / Hospital chart).<br>If no patient medication history is noted on the Patient follow up information / Hospital chart, choose not noted.
p_mhihoth	dropdown	Other	listid: nyn<br>0. no<br>1. yes<br>2. not noted	Patient Medication History (Patient follow up information / Hospital chart).<br>If no patient medication history is noted on the Patient follow up information / Hospital chart, choose not noted.
p_mhihospc	textbox	Specify Other Not Listed		Patient Medication History (Patient follow up information / Hospital chart).
p_ihmhndet	dropdown	Not Determined	listid: nyn<br>0. no<br>1. yes<br>2. not noted	
p_ihfib_div	div	Inhospital Medication		
p_ihfib	dropdown	Inhospital Medication	listid: nynr<br>0. no<br>1. yes<br>2. not recorded	Patient follow up information / Hospital chart<br>0=no<br>1=yes<br>2=not noted<br>If no PCI fibrinolysis is noted on the Patient follow up information / PCI/Hospital chart, choose not recorded.

PREDICT - Hospital Variables

Variable	Type	Caption	List Options	Abstraction Instruction
p_ihflytic	dropdown	Fibrinolytic Given	listid: nynr<br><br>0. no<br>1. yes<br>2. not recorded	Did patient receive fibrinolytic therepy during the inhospital course of care?<br><br>0=no<br><br>1=yes<br><br>2=not noted<br><br>Source = Patient follow up information / Hospital chart
p_ihflytic inel	dropdown	Fibrinolytic Ineligible	listid: ny<br><br>0. no<br>1. yes	Was patient ineligible to receive fibrinolytic therepy during the inhospital course of care?<br><br>0=no<br><br>1=yes<br><br>2=not noted<br><br>Source = Patient follow up information / Hospital chart
p_ihflytic inelsp	textbox	Specify Reason		Please specify the reason why patient was deemed ineligible to receive fibrinolytic therepy during the inhospital course of care.<br><br>Source = Patient follow up information / Hospital chart
p_ihflytic startdt	textbox	Fibrinolysis Start Date		What was the fibrinolysis start date during the inhospital couse of patient care?<br><br>Date value = yyyy/mm/dd<br><br>Source = Patient follow up information / ospital chart
p_ihflytic starttm	textbox	Fibrinolysis Start Time		What was the fibrinolysis start time during the inhospital course of patient care?<br><br>Numerical value based on 24 hour clock<br><br>00:00:00 – hour:min:sec

Variable	Type	Caption	List Options	Abstraction Instruction
				If no value for seconds data available – do not do not use value 00.<br><br>Source = Patient follow up information / Hospital chart
p_ihflytic drug	dropdown	Fibrinolytic Drug Given	listid: drug2<br><br>1. TNK<br>2. tPA (TPA)<br>3. Activase (Alteplase)<br>4. RPA<br>5. Other	Which fibrinolytic drug did patient receive during the inhospital course of care?<br><br>1=TNK (Tenecteplase, Recombinant TPA) no code<br><br>2=TPA (Tissue Plasminogen Activator) Code 661<br><br>3=Activase (Alteplase)<br><br>4=RPA (Reteplase, Retavase) Code 646<br><br>5=other<br><br>Source = Patient follow up information / Hospital chart
p_ihflytic totd	textbox	Total Dose Given (mg)		Indicate the total in mg of TNK administered to patient throughout the inhospital course of patient care.<br><br>Source = Patient follow up information / Hospital chart<br><br>TNK dose is usually given with IV administration of 30 mg for patients's weight <60 kg; 35 mg for > 60 to < 70 kg; 40 mg for > 70 to < 80 kg; 45 mg for > 80 to < 90 kg; and 50 mg for > 90 kg.
p_ihflytic totd2	textbox	Total Dose Given (mg)		Indicate the total dose of tPA administered to patient during the inhospital course of care.<br><br>Source = Patient follow up information / Hospital chart

PREDICT - Hospital Variables

Variable	Type	Caption	List Options	Abstraction Instruction
				Recommended total tPA dose is basen on patient's weight and for AMI the total dose should not exceed 100 mg. tPA totsl dose is usually given with IV administration of accelerated infusion (1.5 hours): 15 mg IV bolus; then 0.75 mg/kg over next 30 minutes (not to exceed 50 mg); then 0.5 mg/kg over 60 minutes (not to exceed 35 mg).
p_ihflytic totd3	textbox	Total Dose Given (mg)		Indicate the total in mg of Activase (Alteplase) administered to patient throughout the inhospital course of patient care.<br><br>Source = Patient follow up information / Hospital chart<br><br>Recommended total Activase (Alteplase) dose is based on patient's weight and for AMI the total dose should not exceed 100 mg.<br><br>Activase totsl dose is usually given with IV administration of accelerated infusion (1.5 hours): 15 mg IV bolus for all patients; then: 1) for patients >67 kg 50 mg infused over the next 30 minutes and then 35 mg over the next 60 minutes; 2) for patients ≤67 kg infusion of 0.75 mg/kg over next 30 minutes (not to exceed 50 mg); and then 0.5 mg/kg over 60 minutes (not to exceed 35 mg).<br><br>Activase could also be administered over the 3 hour IV infusion of the total dose of 100 mg: 60 mg in the first hour (bolus dose of 6-10 mg); 20 mg over the second hour and 20 mg over the third hour. For smaller patients (≤65 kg) a dose of 1.25 mg/kg will be administered over the period of 3 hours.
p_ihflytic totd4	textbox	Total Dose Given (Units)		Indicate the total dose of RPA administered to patient during the inhospital course of care.<br><br>Source = Patient follow up information / Hospital chart

PREDICT - Hospital Variables

Variable	Type	Caption	List Options	Abstraction Instruction
				RPA dose is usually given with IV administration of 10 U bolus over 2 mintes; 30 minutes later give second 10 U IV bolus over 2 minutes (give NS flush before and after each bolus); give heparin and aspirin conjuctively.
p_ihflytic totd5	textbox	Total Dose Given		Indicate the total of other fibrinolytic drug patient received throughout the inhospital course of patient care.<br><br>Source = Patient follow up information / Hospital chart
p_ihflytic inter	dropdown	Fibrinolysis Interrupted	listid: ny<br><br>0. no<br>1. yes	Was the fibrinolysis administration interrupted during the inhospital course of patient care?<br><br>0=no<br>1=yes<br><br>Source = Patient follow up information / Hospital chart
p_ihflytic inrep	dropdown	Fibrinolysis Repeated	listid: ny<br><br>0. no<br>1. yes	Was the fibrinolysis administration repeated during the inhospital course of patient care?<br><br>0=no<br>1=yes<br><br>Source = Patient follow up information / Hospital chart
p_ihflytic enddt	textbox	Fibrinolysis End Date		What was the fibrinolysis end date during the inhospital course of patient care?<br><br>Date value = yyyy/mm/dd<br><br>Source = Patient follow up information / Hospital chart
p_ihflytic endtm	textbox	Fibrinolysis End Time		What was the fibrinolysis end time during the inhospital course of patient care?<br><br>Numerical value based on 24 hour clock<br><br>00:00:00 – hour:min:sec<br><br>If no value for seconds data available – do not do not use value 00.<br><br>Source = Patient follow up information / Hospital chart

PREDICT - Hospital Variables

Variable	Type	Caption	List Options	Abstraction Instruction
p_ihunfhep	dropdown	Unfractionated Heparin Given	listid: nynr<br><br>0. no<br>1. yes<br>2. not recorded	Did patient recive IV Unfractionated Heparin during the inhospital course of care.<br><br>0=no<br><br>1=yes<br><br>2=not noted<br><br>Source = Patient follow up information / Hospital chart<br><br>Indicate if IV Unfractionated Heparin was administered at any time during the inhospital course of care.
p_ihunfhep ibdt	textbox	Initial Bolus Date		The date when administration of IV Unfractionated Heparin initial bolus dose is confirmed during the inhospital course of care.<br><br>Source = Patient follow up information / Hospital chart
p_ihunfhep ibtm	textbox	Initial Bolus Time		The earliest time noted when administration of IV Unfractionated Heparin initial bolus dose is confirmed during the inhospital course of care.<br><br>Source = Patient follow up information / Hospital chart
p_ihunfhep ibdu	textbox	Initial Bolus Dose (Units)		The total in units of IV Unfractionated Heparin initial bolus dose administered to patient throughout the inhospital course of patient care.<br><br>Source = Patient follow up information / Hospital chart<br><br>Unfractionated Heparin initial bolus dose is usually started with IV administration of 60 U/kg to maximum of 4000 U in conjuction with thrombolytics or 80 U/kg to maximum of 5000 U when no trombolytics were administered.
p_ihunfhep idt	textbox	Infusion Date		The date when administration of IV Unfractionated Heparin infusion is confirmed during the inhospital course of patient care.

PREDICT - Hospital Variables

Variable	Type	Caption	List Options	Abstraction Instruction
				Source = Patient follow up information / Hospital chart
p_ihunfhepitm	textbox	Infusion Time		The earliest time noted when administration of IV Unfractionated Heparin infusion is confirmed during the inhospital course of patient care.<br><br>Source = Patient follow up information / PCI/Hospital chart
p_ihunfhepidu	textbox	Infusion Dose (Units)		The total in units of IV Unfractionated Heparin infusion dose administered to patient throughout the inhospital course of patient care.<br><br>Source = Patient follow up information / Hospital chart<br><br>Unfractionated Heparin initial bolus dose is usually started with IV administration of 12 U/kg/hr to maximum of 1000 U/hr in conjuction with thrombolytics or 18 U/kg/hr to maximum of 1500 U/hr when no trombolytics were administered.<br><br>Dose is adjusted to maintain PTT at 1.5 – 2 times control value.
p_ihenox	dropdown	Enoxaparin Given	listid: nynr<br><br>0. no<br>1. yes<br>2. not recorded	Did patient recive IV Enoxaparin during the inhospital course of patient care?<br><br>0=no<br><br>1=yes<br><br>2=not noted<br><br>Source = Patient follow up information / Hospital chart<br><br>Indicate if IV Unfractionated Heparin was administered at any time during during the inhospital course of patient care.
p_ihenoini	dropdown	Inital bolus dose given	listid: ny<br>0. no<br>1. yes	
p_ihenoxdt	textbox	Initial Bolus Date		The date when administration of IV Enoxaparin initial bolus dose is confirmed during the inhospital course of patient care.

PREDICT - Hospital Variables

Variable	Type	Caption	List Options	Abstraction Instruction
				Source = Patient follow up information / Hospital chart
p_ihenoxtm	textbox	Initial Bolus Time		The earliest time noted when administration of IV Enoxaparin initial bolus dose is confirmed during the inhospital course of patient care.<br><br>Source = Patient follow up information / Hospital chart
p_ihenoxibdmg	textbox	Initial Bolus Dose (mg)		The total of IV Enoxaparin initial bolus dose administered to patient throughout the inhospital course of patient care.<br><br>Source = Patient follow up information / Hospital chart<br><br>Enoxaparin initial bolus dose is usually started with IV administration of 30 mg for patients < 75 years of age. Patients ≥ 75 years of age do not receive Enoxaparin IV bolus.
p_ihsceno	dropdown	Subcutoneous (sc) Enoxaparin	listid: ny<br>0. no<br>1. yes	
p_ihenoxidt	textbox	SC injection date		The date when administration of subcutaneous (SC) Enoxaparin infusion is confirmed during the inhospital course of patient care.<br><br>Source = Patient follow up information / Hospital chart
p_ihenoxitm	textbox	SC injection time		The earliest time noted when administration of SC Enoxaparin infusion is confirmed during the inhospital course of patient care.<br><br>Source = Patient follow up information / Hospital chart
p_ihenoxidmg	textbox	SC injection dose (mg)		The total of SC Enoxaparin infusion dose administered to patient throughout the inhospital course of patient care.<br><br>Source = Patient follow up information / Hospital chart<br><br>Enoxaparin infusion dose is usually started with SC administration of 1 mg/kg (maximum 100 mg) for patients < 75 years of age and 0.75 mg/kg (maximum 75 mg) for patients ≥ 75 years of age.
p_ihlmwh	dropdown	Other LMWH product given	listid: nynr<br>0. no<br>1. yes	

PREDICT - Hospital Variables

Variable	Type	Caption	List Options	Abstraction Instruction
			2. not recorded	
p_ihlmwhso	textbox	Specify Other		
p_ihlmwhft	textbox	First given time		
p_ihlmwhdo se	textbox	Total dose (mg)		
p_ihclop	dropdown	Clopidogrel (Plavix)	listid: nynr<br>0. no<br>1. yes<br>2. not recorded	Did patient recive Clopidogrel during the inhospital course of patient care?<br>0=no<br>1=yes<br>2=not noted<br>Source = Patient follow up information / Hospital chart<br>Indicate if Clopidogrel was administered at any time during during the inhospital course of patient care.
p_ihcloptm	textbox	First Given Time		The earliest time noted when administration of Clopidogrel is confirmed during the inhospital course of patient care.<br>Source = Patient follow up information / Hospital chart
p_ihclopto td	textbox	Total Dose Given (mg)		Indicate the total in mg of Clopidogrel administered to patient throughout the inhospital course of care.<br>Source = Patient follow up information / Hospital chart<br>Clopidogrel dose is usually given with a loading dose of 300 mg for 75 years and under, and 75 mg for 67 years and older. The decision to administer additional clopidogrel to patients is usually left to the discretion of the interventional cardiologist.
p_ihglycin h	dropdown	Glycoprotein (GP) llb/llla Inhibitors	listid: nynr<br>0. no<br>1. yes<br>2. not recorded	Did patient recive IV glycoprotein IIb/IIIa inhibitors during the inhospital course of patient care?

PREDICT - Hospital Variables

Variable	Type	Caption	List Options	Abstraction Instruction
				0=no<br><br>1=yes<br><br>2=not noted<br><br>Source = Patient follow up information / Hospital chart<br><br>Indicate if IV Glycoprotein IIb/IIIa Inhibitors were administered at any time during during the inhsopital course of patient care.
p_ihglycinhtype	dropdown	Glycoprotein (GP) llb/llla Inhibitors Type	listid: glycinh2<br><br>1. Abciximab (ReoPro)<br><br>2. Eptifibatide (Intergrilin)<br>3. Tirofiban (Aggrastat)<br>4. Other	What type of glycoprotein IIb/IIa inhibitor did patient recive during the inhospital course of patient care?<br><br>1=Abeiximab (ReoPro)<br><br>2=Eptifibatide (Intergrillin)<br><br>3=Tirofiban (Aggrastat)<br>4= other (specify)<br><br>Source = Patient follow up information / Hospital chart<br><br>Indicate what type of Glycoprotein IIb/IIIa Inhibitor was administered at any time during during the inhospital course of patient care.
p_ihglycinhtypesp	textbox	Specify Other		Please specify which other glycoprotein IIb/IIa inhibitor patient received during the inhospital course of patient care.<br><br>Source = Patient follow up information / Hospital chart
p_ihglycinhtm	textbox	First Given Time		The earliest time noted when administration of glycoprotein IIb/IIa inhibitor is confirmed during the inhospital course of patient care.<br><br>Source = Patient follow up information / Hospital chart
p_ihglycinhtotd	textbox	Total Dose Given (mg)		Indicate the total dose of Abeiximab administered to patient during the inhospital course of care.

Variable	Type	Caption	List Options	Abstraction Instruction
				Source = Patient follow up information / Hospital chart<br><br>Abeiximab dose is usually ggiven with IV bolus administration of 0.25 mg/kg 10-60 minutes before the start of PCI, followed by a continuous intravenous infusion of 0.125 Âµg/kg/min (to a maximum of 10 Âµg/min) for 12 hours.
p_ihglycinhtotd2	textbox	Total Dose Given (mg)		Indicate the total dose of Eptifibatide administered to patient during the inhospital course of care.<br><br>Source = Patient follow up information / Hospital chart<br><br>Eptifibatide dose is usually given with IV bolus administration of 180 Âµg/kg as soon as possible followed by a continuous infusion of 2.0 Âµg/kg/min until hospital discharge or initiation of CABG surgery, up to 72 hours. If a patient is to undergo a percutaneous coronary intervention (PCI) while receiving eptifibatide, the infusion should be continued up to hospital discharge, or for up to 18 to 24 hours after the procedure, whichever comes first, allowing for up to 96 hours of therapy .
p_ihglycinhtotd3	textbox	Total Dose Given (mg)		Indicate the total dose of Tirofiban administered to patient during the inhospital course of care.<br><br>Source = Patient follow up information / Hospital chart<br><br>Tirofiban dose is usually given with IV at an initial rate of 0.4 mcg/kg/min for 30 minutes and then continued at 0.1 mcg/kg/min.
p_ihglycinhtotd4	textbox	Total Dose Given (mg)		Indicate the total in mg of the other of glycoprotein IIb/IIa inhibitor administered to patient throughout the inhospital course of care.<br><br>Source = Patient follow up information / Hospital chart
p_ihtrombinh	dropdown	Thrombin Inhibitors	listid: nynr<br><br>0. no<br>1. yes<br>2. not recorded	Did patient recive trombin inhibitors during the inhospital course of patient care?<br><br>0=no

PREDICT - Hospital Variables

Variable	Type	Caption	List Options	Abstraction Instruction
				1=yes<br>2=not noted<br>Source = Patient follow up information / Hospital chart<br>Indicate if trombin inhibitors were administered at any time during during the inhospital course of patient care.
p_ihtrombinhtype	dropdown	Thrombin Inhibitors Type	listid: trombinh<br>1. Bivalirudin<br>2. Lepirudin<br>3. Desirudin<br>4. Other	What type of trombin inhibitor did patient recive during the inhospital course of patient care?<br>=Bivalirudin (Angiomax)<br>2=Lepirudin (Refludan)<br>3=Desirudin<br>4=Other<br>Source = Patient follow up information / PCI/Hospital chart<br>Indicate what type of trombin inhibitor administered at any time during during the inhospital course of patient care.
p_ihtrombinhtm	textbox	First Given Time		The earliest time noted when administration of trombin inhibitor is confirmed during the inhospital course of patient care.<br>Source = Patient follow up information / Hospital chart
p_ihtrombinhtotd	textbox	Total Dose Given (mg)		Indicate the total in mg of Desirudin administered to patient throughout the inhospital course of care.

Variable	Type	Caption	List Options	Abstraction Instruction
				Source = Patient follow up information / Hospital chart
p_ihtrombinhtotd2	textbox	Total Dose Given (mg)		*Indicate the total in mg of Desirudin administered to patient throughout the inhospital course of care.*<br><br>*Source = Patient follow up information / Hospital chart*
p_ihtrombinhtotd3	textbox	Total Dose Given (mg)		Indicate the total in mg of other trombin inhibitor administered to patient throughout the inhospital course of care.<br><br>Source = Patient follow up information / Hospital chart
p_ihasa2	dropdown	ASA (Aspirin) Given	listid: nynr<br><br>0. no<br>1. yes<br>2. not recorded	Indicate if ASA was administered at any time during the inhospital course of patient care.<br><br>0=no<br>1=yes<br>2=not noted<br><br>Source = Patient follow up information / Hospital chart
p_ihasaftm	textbox	First Given Time		The earliest time noted when administration of ASA is confirmed during the inhospital course of patient care.<br><br>Source = Patient follow up information / Hospital chart
p_ihasamg	textbox	Total Dose (mg)		The total in mg of all doses of ASA administered to patient throughout the inhospital course of care.<br><br>Â· Usual dose of ASA is 160 mg (2x80 mg) for the patient to chew and swallow.<br><br>Source = Patient follow up information / Hospital chart

PREDICT - Hospital Variables

Variable	Type	Caption	List Options	Abstraction Instruction
p_ihnitro2	dropdown	Nitrolingual Spray Given	listid: nynr<br>0. no<br>1. yes<br>2. not recorded	Indicate if Nitrolingual spray 0.4 mg/spray was administered at any time during the inhospital course of patient care.<br>0=no<br>1=yes<br>2=not noted<br>Source = Patient follow up information / Hospital chart
p_ihnitro2ftm	textbox	First Given Time		
p_ihnitro2mg	textbox	Total Dose(mg)		
p_ihnitropatch	dropdown	Nitro Patch	listid: ny<br>0. no<br>1. yes	
p_ihnitroftm	textbox	First Given Time		The earliest time noted when administration of Nitrolingual spray 0.4 mg/spray is confirmed during the inhospital course of patient care.<br>Source = Patient follow up information / Hospital chart
p_ihnitromg	textbox	Total Dose (mg)		The total in mg of all doses of Nitrolingual spray administered to patient throughout the inhospital course of care.<br>Â· Maximal number of Nitrolingual spray 0.4 mg/spray is 3; single spray is administered every 5 minutes.<br>Source = Patient follow up information / Hospital chart
p_ihnitrodrip	dropdown	Nitro IV Drip Given	listid: nynr<br>0. no<br>1. yes<br>2. not recorded	Indicate if Nitro IV drip was administered at any time during the inhospital course of patient care.<br>0=no<br>1=yes<br>2=not noted

PREDICT - Hospital Variables

Variable	Type	Caption	List Options	Abstraction Instruction
				Source = Patient follow up information / Hospital chart
p_ihnitrodripftm	textbox	First Given Time		The earliest time noted when administration of Nitro IV drip during the inhospital course of patient care.<br><br>Source = Patient follow up information / Hospital chart
p_ihnitromg2	textbox	Total Dose (Âµg/min)		The total in mg of Nitro IV drip administered to patient throughout the inhospital course of care.<br><br>Â· Nitro drip is usually started with IV administration of 0.1 Âµg/kg/min and then tittred to up to 200 Âµg/min.<br><br>Source = Patient follow up information / Hospital chart
p_ihlabtest_div	div	Inhospital Lab and Cardiac Enzymes		
p_ihlabtest	dropdown	Were Lab Tests Done during inhospital stay?	listid: ny<br>0. no<br>1. yes	Were lab tests done during the inhospital course of patient care?<br><br>0=no<br>1=yes<br><br>Source = Patient follow up information / Hospital chart<br><br>Indicate if lab tests were done at any time during the inhospital course of patient care.
p_ihlabtestdt	textbox	Lab Tests Date		The date when the lab tests were done first time during the inhospital course of patient care.<br><br>Source = Patient follow up information / Hospital chart
p_ihlabtesttm	textbox	Lab Tests Time		The time when the lab tests were done first time during the inhospital course of patient care.<br><br>Source = Patient follow up information / Hospital chart
p_ihchol	dropdown	Cholesterol	listid: ny<br>0. no<br>1. yes	Indicate if cholesterol was measured during the inhospital course of patient care?<br><br>0=no<br>1=yes<br><br>Source = Patient follow up information / Hospital chart

PREDICT - Hospital Variables

Variable	Type	Caption	List Options	Abstraction Instruction
p_ihchollev	textbox	Cholesterol Levels (mmol/L)		Indicate the first cholesterol levels measured during the inhospital course of patient care.<br><br>Source = Patient follow up information / Hospital chart
p_ihhdl	dropdown	HDL	listid: ny<br>0. no<br>1. yes	Indicate if HDL was measured during the inhospital course of patient care?<br><br>0=no<br>1=yes<br><br>Source = Patient follow up information / Hospital chart
p_ihhdllev	textbox	HDL Levels		Indicate the first HDL levels measured during the inhospital course of patient care.<br><br>Source = Patient follow up information / Hospital chart
p_ihldl	dropdown	LDL	listid: ny<br>0. no<br>1. yes	Indicate if LDL was measured during the inhospital course of patient care?<br><br>0=no<br>1=yes<br><br>Source = Patient follow up information / Hospital chart
p_ihldllev	textbox	LDL Levels		Indicate the first LDL levels measured during the inhospital course of patient care.<br><br>Source = Patient follow up information / Hospital chart
p_ihtriglyc	dropdown	Triglycerides	listid: ny<br>0. no<br>1. yes	Indicate if tryglicerides were measured during the inhospital course of patient care?<br><br>0=no<br>1=yes<br><br>Source = Patient follow up information / Hospital chart
p_ihtriglyclev	textbox	Triglyercrides Levels (mmol/L)		Indicate the first tryglicerides levels measured during the inhospital course of patient care.<br><br>Source = Patient follow up information / Hospital chart
p_ihgluc	dropdown	Glucose	listid: ny<br>0. no<br>1. yes	Indicate if glucose was measured during the inhospital course of patient care?<br><br>0=no<br>1=yes

Variable	Type	Caption	List Options	Abstraction Instruction
				Source = Patient follow up information / Hospital chart
p_ihglucle v	textbox	Glucose Levels		Indicate the first glucose levels measured during the inhospital course of patient care.<br><br>Source = Patient follow up information / Hospital chart
p_ihcreat	dropdown	Creatinine	listid: ny<br><br>0. no<br>1. yes	Indicate if creatinine was measured during the inhospital course of patient care?<br><br>0=no<br>1=yes<br><br>Source = Patient follow up information / Hospital chart
p_ihcreatl ev	textbox	Creatinine Levels		Indicate the first creatinine levels measured during the inhospital course of patient care.<br><br>Source = Patient follow up information / Hospital chart
p_ihcaren	dropdown	Were Cardiac Enzymes Done During Inhospital Stay?	listid: ny<br><br>0. no<br>1. yes	Were cardiac enzymes done during the inhospital course of patient care?<br><br>0=no<br>1=yes<br><br>Source = Patient follow up information / Hospital chart<br><br>Indicate if cardiac enzymes were done at any time during the inhospital course of patient care.
p_ihfcaren dt	textbox	First Cardiac Enzymes Measurement Date		The date when the cardiac enzymes were measured first time during the inhospital course of patient care.<br><br>Source = Patient follow up information / Hospital chart
p_ihfcaren tm	textbox	First Cardiac Enzymes Measurement Time		The time when the cardiac enzymes were measured first time during the inhospital course of patient care.<br><br>Source = Patient follow up information / Hospital chart
p_ihfck	dropdown	First Creatine Kinase (CK)	listid: ny<br><br>0. no<br>1. yes	Indicate if first CK levels were measured during the inhospital course of patient care?<br><br>0=no<br>1=yes

PREDICT - Hospital Variables

Variable	Type	Caption	List Options	Abstraction Instruction
				Source = Patient follow up information / Hospital chart
p_ihfcklev	textbox	First CK Levels (U/L)		Indicate the first CK levels measured during the inhospital course of patient care.<br><br>Source = Patient follow up information / Hospital chart
p_ihfckmb	dropdown	First Creatine Kinase - MB (CK-MB)	listid: ny<br><br>0. no<br>1. yes	Indicate if first CK-MB levels were measured during the inhospital course of patient care?<br><br>0=no<br>1=yes<br><br>Source = Patient follow up information / Hospital chart
p_ihfckmblev	textbox	First CK-MB Levels (U/L)		Indicate the first CK-MB levels measured during the inhospital course of patient care.<br><br>Source = Patient follow up information / Hospital chart
p_ihftropi	dropdown	First Troponin I	listid: ny<br><br>0. no<br>1. yes	Indicate if first Troponin I levels were measured during the inhospital course of patient care?<br><br>0=no<br>1=yes<br><br>Source = Patient follow up information / Hospital chart
p_ihftropilev	textbox	First Troponin I Levels (Âµg/L)		Indicate the first Troponin I levels measured during the inhospital course of patient care.<br><br>Source = Patient follow up information / Hospital chart
p_ihftropt	dropdown	First Troponin T	listid: ny<br><br>0. no<br>1. yes	Indicate if first Troponin T levels were measured during the inhospital course of patient care?<br><br>0=no<br>1=yes<br><br>Source = Patient follow up information / Hospital chart
p_ihftroptlev	textbox	First Troponin T Levels (Âµg/L)		Indicate the first Troponin T levels measured during the inhospital course of patient care.<br><br>Source = Patient follow up information / Hospital chart
p_ihpcarendt	textbox	Peak Cardiac Enzymes Measurement Date		The date when the peak cardiac enzymes were measured during the inhospital course of patient care.

Variable	Type	Caption	List Options	Abstraction Instruction
				Source = Patient follow up information / Hospital chart
p_ihpcarentm	textbox	Peak Cardiac Enzymes Measurement Time		The time when the peak cardiac enzymes were measured during the inhospital course of patient care.<br><br>Source = Patient follow up information / Hospital chart
p_ihpck	dropdown	Peak Creatine Kinase (CK)	listid: ny<br><br>0. no<br>1. yes	Indicate if peak CK levels were measured during the inhospital course of patient care?<br><br>0=no<br>1=yes<br><br>Source = Patient follow up information / Hospital chart
p_ihpcklev	textbox	Peak CK Levels (U/L)		Indicate the peak CK levels measured during the inhospital course of patient care.<br><br>Source = Patient follow up information / Hospital chart
p_ihpckmb	dropdown	Peak Creatine Kinase - MB (CK-MB)	listid: ny<br><br>0. no<br>1. yes	Indicate the peak CK-MB levels measured during the inhospital course of patient care.<br><br>Source = Patient follow up information / Hospital chart
p_ihpckmblev	textbox	Peak CK-MB Levels (U/L)		Indicate the peak CK-MB levels measured during the inhospital course of patient care.<br><br>Source = Patient follow up information / Hospital chart
p_ihptropi	dropdown	Peak Troponin I	listid: ny<br><br>0. no<br>1. yes	Indicate if peak Troponin I levels were measured during the inhospital course of patient care?<br><br>0=no<br>1=yes<br><br>Source = Patient follow up information / Hospital chart
p_ihptropilev	textbox	Peak Troponin I Levels (Âµg/L)		Indicate the peak Troponin I levels measured during the inhospital course of patient care.<br><br>Source = Patient follow up information / Hospital chart
p_ihptropt	dropdown	Peak Troponin T	listid: ny<br><br>0. no	Indicate if peak Troponin T levels were measured during the inhospital course of patient care?

PREDICT - Hospital Variables

Variable	Type	Caption	List Options	Abstraction Instruction
			1. yes	0=no<br>1=yes<br><br>Source = Patient follow up information / Hospital chart
p_ihptroptlev	textbox	Peak Troponin T Levels (Âµg/L)		Indicate the peak Troponin T levels measured during the inhospital course of patient care.<br><br>Source = Patient follow up information / Hospital chart
p_ihecg_div	div	ECG Recording in Hospital		
p_ihecg	dropdown	Does Inhospital ECG Recording Exist?	listid: ny<br>0. no<br>1. yes	Did hospital personnel record the patient ECG?<br><br>0=no<br>1=yes<br><br>Indicate no or yes, whether inhospital ECG recording exists.<br><br>Source = Patient follow up information / Hospital chart
p_ihecgrec	dropdown	How many ECG Recordings (total number)	listid: 3plus<br>1. one<br>2. two<br>3. three<br>4. more than three	How many recordings of the ECG process (total number) were made in hospital?<br><br>1=one<br><br>2=two<br><br>3=three<br><br>4=more than three<br><br>Source = Patient follow up information / Hospital chart
p_ihecgrecnum	textbox	How many?		
p_fihecg_div	div	First Inhospital ECG (first one on arrival in hospital)		
p_fihecg	dropdown	Does First Inhospital ECG Recording Exist?	listid: ny<br>0. no<br>1. yes	Did hospital personnel record the patient ECG on arrival in hospital?<br><br>0=no<br>1=yes<br><br>Indicate no or yes, whether first inhospital ECG recording exists.

Variable	Type	Caption	List Options	Abstraction Instruction
				Source = Patient follow up information / Hospital chart
p_fihecgdt	textbox	Date		What was the date of the patient first ECG in hospital?<br><br>Date value = yyyy/mm/dd<br><br>Source = Patient follow up information / Hospital chart
p_fihecgtm	textbox	Time		What was the time of the patient first ECG in hospital?<br><br>Numerical value based on 24 hour clock<br><br>00:00:00 – hour:min:sec<br><br>If no value for seconds data available – do not do not use value 00<br><br>Source = Patient follow up information / Hospital chart
p_fihecgrev	dropdown	First ECG Reviewed	listid: nyu<br><br>0. no<br>1. yes<br>2. unknown	Indicate whether or not the first inhospital ECG was reviewed by the attending hospital physician site or if the attendant annotations and QA data was generated solely by the device software.<br><br>0=no<br>1=yes<br>2=unknown<br><br>The goal is for sites to review the recordings so as to ''correct'' any oversights by the software.<br>Source = Patient follow up information / Hospital chart
p_fihecgd	dropdown	First ECG Data Exists	listid: ny<br><br>0. no<br>1. yes	Is there the first inhospital ECG data available on request?<br><br>0=no<br>1=yes<br><br>Source = Patient follow up information / Hospital chart
p_fihecgdgca	dropdown	First ECG Diagnosis - Computer Assisted	listid: ny<br><br>0. no<br>1. yes	Indicate whether or not there was computer assisted diagnosis for the first inhospital ECG.<br><br>0=no<br>1=yes<br><br>Source = Patient follow up information / Hospital chart
p_fihecgdgsp	textbox	First ECG Diagnosis - Computer Assisted - Specify 1		Please specify computer assisted diagnosis for the first inhospital ECG.

PREDICT - Hospital Variables

Variable	Type	Caption	List Options	Abstraction Instruction
				Value = text<br><br>Source = Patient follow up information / Hospital chart
p_fihecgdgsp_2	textbox	First ECG Diagnosis - Computer Assisted - Specify 2 (cont)		
p_fihecgdgsp_3	textbox	First ECG Diagnosis - Computer Assisted - Specify 3 (cont)		
p_fihecgdg	textbox	First ECG Diagnosis 1		Indicate the diagnosis based on patient first inhospital ECG made by hospital attending physician.<br><br>Source = Patient follow up information / Hospital chart
p_fihecgdg_2	textbox	First ECG Diagnosis 2 (cont)		
p_fihecgdg_3	textbox	First ECG Diagnosis 3 (cont)		
p_stefihecg	dropdown	ST Elevation	listid: nyn<br>0. no<br>1. yes<br>2. not noted	Is there ST elevation on patient first inhospital ECG?<br><br>0=no<br>1=yes<br><br>Source = Patient follow up information / Hospital chart
p_stemicrfihecg	dropdown	STEMI Criteria Met	listid: nynr<br><br>0. no<br>1. yes<br>2. not recorded	Is ST Elevation Myocardial Infarction (STEMI) criteria met on patient first inhospital ECG?<br><br>0=no<br><br>1=yes<br><br>2=not recorded<br><br>Source = Patient follow up information / Hospital chart
p_stemilfihecg	textbox	STEMI Location		Indicate, if possible the STEMI location based on patient first ECG in hsopital.<br><br>Source = Patient follow up information / Hospital chart
p_ihecg6h_div	div	Inhospital ECG (5 hours after arrival in hospital)		
p_ihecg6h	dropdown	Does Inhospital ECG Recording Exist (6 hours after arrival)?	listid: ny	Did hospital personnel record the patient ECG 6 hours after arrival in hospital

PREDICT - Hospital Variables

Variable	Type	Caption	List Options	Abstraction Instruction
			0. no<br>1. yes	0=no<br>1=yes<br><br>Indicate no or yes, whether 6 hour inhospital ECG recording exists.<br><br>Source = Patient follow up information / Hospital chart
p_ihecg6hdt	textbox	Date		What was the date of the patient ECG recorded 6 hours after arrival in hospital?<br><br>Date value = yyyy/mm/dd<br><br>Source = Patient follow up information / Hospital chart
p_ihecg6htm	textbox	Time		What was the time of the patient ECG recorded 6 hours after arrival in hospital?<br><br>Numerical value based on 24 hour clock<br><br>00:00:00 – hour:min:sec<br><br>If no value for seconds data available – do not do not use value 00<br><br>Source = Patient follow up information / Hospital chart
p_ihecg6hrev	dropdown	Inhospital ECG (6 hours) Reviewed	listid: nyu<br><br>0. no<br>1. yes<br>2. unknown	Indicate whether or not the patient ECG recorded 6 hours after arrival in hospital was reviewed by the attending hospital hysician site or if the attendant annotations and QA data was generated solely by the device software.<br><br>0=no<br>1=yes<br>2=unknown<br><br>The goal is for sites to review the recordings so as to ''correct'' any oversights by the software.<br>Source = Patient follow up information / Hospital chart
p_ihecg6hd	dropdown	Inhospital ECG (6 hours) Data Exists	listid: ny<br><br>0. no<br>1. yes	Is there data available on request for the patient ECG recorded 6 hours after arrival in hospital?<br><br>0=no<br>1=yes<br><br>Source = Patient follow up information / Hospital chart

PREDICT - Hospital Variables

Variable	Type	Caption	List Options	Abstraction Instruction
p_ihecg6hdgca	dropdown	Inhospital ECG (6 hours) Diagnosis - Computer Assisted	listid: ny<br><br>0. no<br>1. yes	Indicate whether or not there was computer assisted diagnosis for the patient ECG recorded 6 hours after arrival in hospital.<br><br>0=no<br>1=yes<br><br>Source = Patient follow up information / Hospital chart
p_ihecg6hdgsp	textbox	Inhospital ECG (6 hours) Diagnosis - Computer Assisted - Specify 1		Please specify computer assisted diagnosis for the patient ECG recorded 6 hours after arrival in hospital.<br><br>Value = text<br><br>Source = Patient follow up information / Hospital chart
p_ihecg6hdgsp_2	textbox	Inhospital ECG (6 hours) Diagnosis - Computer Assisted - Specify 2 (cont)		
p_ihecg6hdgsp_3	textbox	Inhospital ECG (6 hours) Diagnosis - Computer Assisted - Specify 3 (cont)		
p_ihecg6hg	textbox	Inhospital ECG (6 hours) Diagnosis 1		Indicate the diagnosis based on patient ECG recorded 6 hours after arrival in hospital made by hospital attending physician.<br><br>Source = Patient follow up information / Hospital chart
p_ihecg6hg_2	textbox	Inhospital ECG (6 hours) Diagnosis 2 (cont)		
p_ihecg6hg_3	textbox	Inhospital ECG (6 hours) Diagnosis 3 (cont)		
p_steihecg6h	dropdown	ST Elevation	listid: nyn<br><br>0. no<br>1. yes<br>2. not noted	Is there ST elevation on patient ECG recorded 6 hours after arrival in hospital?<br><br>0=no<br>1=yes<br><br>Source = Patient follow up information / Hospital chart
p_stemicrihecg6h	dropdown	STEMI Criteria Met	listid: nynr<br><br>0. no<br>1. yes<br>2. not recorded	Is ST Elevation Myocardial Infarction (STEMI) criteria met on patient ECG recorded 6 hours after arrival in hospital?<br><br>0=no

Variable	Type	Caption	List Options	Abstraction Instruction
				1=yes<br>2=not noted<br>Source = Patient follow up information / Hospital chart
p_stemilihecg6h	textbox	STEMI Location		Indicate, if possible the STEMI location based on patient ECG recorded 6 hours after arrival in hospital.<br>Source = Patient follow up information / PCI/Hospital chart
p_ihecg6hredcp	dropdown	Reduction in the chest pain intensity	listid: nyn<br>0. no<br>1. yes<br>2. not noted	Indicate, if possible, if there is a significant reduction in the patient chest pain intensity during the 6 hour inhospital period.<br>0=no<br>1=yes<br>2=not noted<br>Source = Patient follow up information / Hospital chart
p_ihecg6hrestemi	dropdown	Reduction in ST-segment elevation (i.e. > 50%)	listid: nyn<br>0. no<br>1. yes<br>2. not noted	Indicate, if possible, if there is a significant reduction in ST-segment elevation (i.e. > 50%) on patient ECG recorded 6 hours after arrival in hospital.<br>0=no<br>1=yes<br>2=not noted<br>Source = Patient follow up information / Hospital chart
p_ihecg24h_div	div	Inhospital ECG (24 hours after arrival)?		
p_ihecg24h	dropdown	Does Inhospital ECG Recording Exist (24 hours after arrival in hospital)?	listid: ny<br>0. no<br>1. yes	Did hospital personnel record the patient ECG 24 hours after arrival in hospital<br>0=no<br>1=yes<br>Indicate no or yes, whether 24 hour inhospital ECG recording exists.<br>Source = Patient follow up information / Hospital chart
p_ihecg24hdt	textbox	Date		What was the date of the patient ECG recorded 24 hours after arrival in hospital?

Variable	Type	Caption	List Options	Abstraction Instruction
				Date value = yyyy/mm/dd<br><br>Source = Patient follow up information / Hospital chart
p_ihecg24htm	textbox	Time		What was the time of the patient ECG recorded 24 hours after arrival in hospital?<br><br>Numerical value based on 24 hour clock<br><br>00:00:00 – hour:min:sec<br><br>If no value for seconds data available – do not do not use value 00<br><br>Source = Patient follow up information / Hospital chart
p_ihecg24hrev	dropdown	Inhospital ECG (24 hours) Reviewed	listid: nyu<br><br>0. no<br>1. yes<br>2. unknown	Indicate whether or not the 24 hour inhospital ECG was reviewed by the attending hospital hysician site or if the attendant annotations and QA data was generated solely by the device software.<br><br>0=no<br>1=yes<br>2=unknown<br><br>The goal is for sites to review the recordings so as to ''correct'' any oversights by the software.<br>Source = Patient follow up information / Hospital chart
p_ihecg24hd	dropdown	Inhospital ECG (24 hours) Data Exists	listid: ny<br><br>0. no<br>1. yes	Is there data available on request for the patient ECG recorded 24 hours after arrival in hospital?<br><br>0=no<br>1=yes<br><br>Source = Patient follow up information / Hospital chart
p_ihecg24hdgca	dropdown	Inhospital ECG (24 hours) Diagnosis - Computer Assisted	listid: ny<br><br>0. no<br>1. yes	Indicate whether or not there was computer assisted diagnosis for the patient ECG recorded 24 hours after arrival in hospital.<br><br>0=no<br>1=yes<br><br>Source = Patient follow up information / Hospital chart
p_ihecg24hdgsp	textbox	Inhospital ECG (24 hours) Diagnosis - Computer Assisted - Specify 1		Please specify computer assisted diagnosis for the patient ECG recorded 24 hours after arrival in hospital.

PREDICT - Hospital Variables

Variable	Type	Caption	List Options	Abstraction Instruction
				.<br><br>Value = text<br><br>Source = Patient follow up information / Hospital chart
p_ihecg24hdgsp_2	textbox	Inhospital ECG (24 hours) Diagnosis - Computer Assisted - Specify 2 (cont)		
p_ihecg24hdgsp_3	textbox	Inhospital ECG (24 hours) Diagnosis - Computer Assisted - Specify 3 (cont)		
p_ihecg24hdg	textbox	Inhospital ECG (24 hours) Diagnosis 1		Indicate the diagnosis based on patient ECG recorded 24 hours after arrival in hospital.<br><br>made by hospital attending physician.<br><br>Source = Patient follow up information / Hospital chart
p_ihecg24hdg_2	textbox	Inhospital ECG (24 hours) Diagnosis 2 (cont)		
p_ihecg24hdg_3	textbox	Inhospital ECG (24 hours) Diagnosis 3 (cont)		
p_steihecg24h	dropdown	ST Elevation	listid: nyn<br><br>0. no<br>1. yes<br>2. not noted	Is there ST elevation on patient ECG recorded 24 hours after arrival in hospital?<br><br>0=no<br>1=yes<br><br>Source = Patient follow up information / Hospital chart
p_stemicrihecg24h	dropdown	STEMI Criteria Met	listid: nynr<br><br>0. no<br>1. yes<br>2. not recorded	Is ST Elevation Myocardial Infarction (STEMI) criteria met on patient ECG recorded 24 hours after arrival in hospital?<br><br>0=no<br><br>1=yes<br><br>2=not noted<br><br>Source = Patient follow up information / Hospital chart
p_stemilihecg24h	textbox	STEMI Location		Indicate, if possible the STEMI location based on patient ECG recorded 24 hours after arrival in hospital.

Variable	Type	Caption	List Options	Abstraction Instruction
				Source = Patient follow up information / PCI/Hospital chart
p_ihecg24h redecg	dropdown	Reduction in the chest pain intensity	listid: nyu<br>0. no<br>1. yes<br>2. unknown	Indicate, if possible, if there is a significant reduction in the patient's chest pain intensity during the 24 hour inhospital period.<br>0=no<br>1=yes<br>2=not noted<br>Source = Patient follow up information / Hospital chart
p_ihecg24h redstat	dropdown	Reduction in ST-Segment elevation (i.e. > 50%)	listid: nyn<br>0. no<br>1. yes<br>2. not noted	Indicate, if possible, if there is a significant reduction in ST-segment elevation (i.e. > 50%) on patient ECG recorded 24 hours after arrival in hospital.<br>0=no<br>1=yes<br>2=not noted<br>Source = Patient follow up information / Hospital chart
p_ihecg48h _div	div	Inhospital ECG (48 hours after arrival)?		
p_ihecg48h	dropdown	Does Inhospital ECG Recording Exist (48 hours after arrival in hospital)?	listid: ny<br>0. no<br>1. yes	Did hospital personnel record the patient ECG 48 hours after arrival in hospital<br>0=no<br>1=yes<br>Indicate no or yes, whether 48 hour inhospital ECG recording exists.<br>Source = Patient follow up information / Hospital chart
p_ihecg48h dt	textbox	Date		What was the date of the patient ECG recorded 48 hours after arrival in hospital?<br>Date value = yyyy/mm/dd<br>Source = Patient follow up information / Hospital chart
p_ihecg48h tm	textbox	Time		What was the time of the patient ECG recorded 48 hours after arrival in hospital?<br>Numerical value based on 24 hour clock<br>00:00:00 – hour:min:sec

Variable	Type	Caption	List Options	Abstraction Instruction
				If no value for seconds data available – do not do not use value 00<br><br>Source = Patient follow up information / Hospital chart
p_ihecg48hrev	dropdown	Inhospital ECG (48 hours) Reviewed	listid: nyu<br><br>0. no<br>1. yes<br>2. unknown	Indicate whether or not the patient ECG recorded 48 hours after arrival in hospital was reviewed by the attending hospital hysician site or if the attendant annotations and QA data was generated solely by the device software.<br><br>0=no<br>1=yes<br>2=unknown<br><br>The goal is for sites to review the recordings so as to ''correct'' any oversights by the software.<br>Source = Patient follow up information / Hospital chart
p_ihecg48hd	dropdown	Inhospital ECG (48 hours) Data Exists	listid: ny<br><br>0. no<br>1. yes	Is there data available on request for the patient ECG recorded 48 hours after arrival in hospital?<br><br>0=no<br>1=yes<br><br>Source = Patient follow up information / Hospital chart
p_ihecg48hdgca	dropdown	Inhospital ECG (48 hours) Diagnosis - Computer Assisted	listid: ny<br><br>0. no<br>1. yes	Indicate whether or not there was computer assisted diagnosis for patient ECG recorded 48 hours after arrival in hospital.<br><br>0=no<br>1=yes<br><br>Source = Patient follow up information / Hospital chart
p_ihecg48hdgsp	textbox	Inhospital ECG (48 hours) Diagnosis - Computer Assisted - Specify 1	listid: ny<br><br>0. no<br>1. yes	Please specify computer assisted diagnosis for the the patient ECG recorded 48 hours after arrival in hospital.<br><br>.<br><br>Value = text<br><br>Source = Patient follow up information / Hospital chart
p_ihecg48hdgsp_2	textbox	Inhospital ECG (48 hours) Diagnosis - Computer Assisted - Specify 2 (cont)		

PREDICT - Hospital Variables

Variable	Type	Caption	List Options	Abstraction Instruction
p_ihecg48hdgsp_3	textbox	Inhospital ECG (48 hours) Diagnosis - Computer Assisted - Specify 3 (cont)		
p_ihecg48hdg	textbox	Inhospital ECG (48 hours) Diagnosis 1	listid: ny<br><br>0. no<br>1. yes	Indicate the diagnosis based on patient ECG recorded 48 hours after arrival in hospital.<br><br>made by hospital attending physician.<br><br>Source = Patient follow up information / Hospital chart
p_ihecg48hdg_2	textbox	Inhospital ECG (48 hours) Diagnosis 2 (cont)		
p_ihecg48hdg_3	textbox	Inhospital ECG (48 hours) Diagnosis 3 (cont)		
p_steihecg48h	dropdown	ST Elevation	listid: nyn<br><br>0. no<br>1. yes<br>2. not noted	Is there ST elevation on patient ECG recorded 48 hours after arrival in hospital?<br><br>0=no<br>1=yes<br><br>Source = Patient follow up information / Hospital chart
p_stemicrihecg48h	dropdown	STEMI Criteria Met	listid: nynr<br><br>0. no<br>1. yes<br>2. not recorded	Is ST Elevation Myocardial Infarction (STEMI) criteria met on patient ECG recorded 48 hours after arrival in hospital?<br><br>0=no<br><br>1=yes<br><br>2=not noted<br><br>Source = Patient follow up information / Hospital chart
p_stemilihecg48h	textbox	STEMI Location		Indicate, if possible the STEMI location based on patient ECG recorded 48 hours after arrival in hospital.<br><br>Source = Patient follow up information / PCI/Hospital chart
p_ihecg48hredecg	dropdown	Reduction in the chest pain intensity	listid: nyu<br><br>0. no<br>1. yes<br>2. unknown	Indicate, if possible, if there is a significant reduction in the patient's chest pain intensity during the 48 hour inhospital period.<br><br>0=no<br>1=yes

PREDICT - Hospital Variables

Variable	Type	Caption	List Options	Abstraction Instruction
				2=not noted<br><br>Source = Patient follow up information / Hospital chart
p_ihecg48hredstat	dropdown	Reduction in ST-Segment elevation (i.e. >50%)	listid: nyn<br><br>0. no<br>1. yes<br>2. not noted	Indicate, if possible, if there is a significant reduction in ST-segment elevation (i.e. > 50%) on patient ECG recorded 48 hours after arrival in hospital.<br><br>0=no<br>1=yes<br>2=not noted<br><br>Source = Patient follow up information / Hospital chart
p_ihclinev	dropdown	Clinical Events Occurred in Hospital	listid: nyn<br><br>0. no<br>1. yes<br>2. not noted	Indicate if any clinical events from the list below occurred during the inhospital course of patient care.<br><br>List of clinical events:<br>Death<br>Cardiogenic shock<br>New of worsening CHF<br>Recurrent ischemia<br>Myocardial reinfarction<br>Suspected TIA/Stroke<br>Revascularization<br>Bleeding<br>Transfusion<br>Temporary pacemaker<br>Cardiac arrest<br>Other<br><br>Source = Patient follow up information / Hospital chart
p_ihclinevdth	dropdown	Death	listid: ny<br><br>0. no<br>1. yes	Indicate if patient died during the PCI course of patient care.<br><br>0=no<br>1=yes<br><br>Source = Patient follow up information / PCI/Hospital chart
p_ihclinevdthdt	textbox	Death Date		What was the date of the patient's death in hospital?<br><br>Date value = yyyy/mm/dd<br><br>Source = Patient follow up information / Hospital chart
p_ihclinevdthtm	textbox	Death Time		What was the time of the patient's death in hospital?<br><br>Numerical value based on 24 hour clock

Variable	Type	Caption	List Options	Abstraction Instruction
				00:00:00 – hour:min:sec<br><br>If no value for seconds data available – do not do not use value 00<br><br>Source = Patient follow up information / Hospital chart
p_ihclinevcs	dropdown	Cardiogenic Shock	listid: ny<br><br>0. no<br>1. yes	Indicate if patient had cardiogenic shock during the inhospital course of patient care.<br><br>0=no<br>1=yes<br><br>Source = Patient follow up information / PCI/Hospital chart
p_ihclinevcsdt	textbox	Cardiogenic Shock Date		What was the date of the patient's cardiogenic shock i during the inhospital course of patient care?<br><br>Date value = yyyy/mm/dd<br><br>Source = Patient follow up information / Hospital chart
p_ihclinevcstm	textbox	Cardiogenic Shock Time		What was the time of the patient's cardiogenic shock i during the inhospital course of patient care?<br><br>Numerical value based on 24 hour clock<br><br>00:00:00 – hour:min:sec<br><br>If no value for seconds data available – do not do not use value 00<br><br>Source = Patient follow up information / Hospital chart
p_ihclinevnwchf	dropdown	New or Worsening CHF	listid: ny<br><br>0. no<br>1. yes	Indicate if patient had new or worsening congestive heart failure (CHF) during the inhospital course of patient care.<br><br>0=no<br>1=yes<br><br>Source = Patient follow up information / Hospital chart
p_ihclinevnwchfdt	textbox	New or Worsening CHF Date		What was the date of the patient's new or worsening congestive heart failure (CHF) during the inhospital course of patient care?<br><br>Date value = yyyy/mm/dd

Variable	Type	Caption	List Options	Abstraction Instruction
				Source = Patient follow up information / Hospital chart
p_ihclinev nwchftm	textbox	New or Worsening CHF Time		What was the time of the patient's new or worsening congestive heart failure (CHF) during the inhospital course of patient care?<br><br>Numerical value based on 24 hour clock<br><br>00:00:00 – hour:min:sec<br><br>If no value for seconds data available – do not do not use value 00<br><br>Source = Patient follow up information / Hospital chart
p_ihclinev risch	dropdown	Recurrent Ischemia	listid: ny<br><br>0. no<br>1. yes	Indicate if patient had recurrent ischemia during the inhsopital course of patient care.<br><br>0=no<br>1=yes<br><br>Source = Patient follow up information / Hospital chart
p_ihclinev rischdt	textbox	Reccurrent Ischemia Date		What was the date of the patient's recurrent ischemia during the inhospital course of patient care?<br><br>Date value = yyyy/mm/dd<br><br>Source = Patient follow up information / Hospital chart
p_ihclinev rischtm	textbox	Recurrent Ischemia Time		What was the time of the patient's recurrent ischemia during the inhospital course of patient care?<br><br>Numerical value based on 24 hour clock<br><br>00:00:00 – hour:min:sec<br><br>If no value for seconds data available – do not do not use value 00<br><br>Source = Patient follow up information / Hospital chart
p_ihclinev mrein	dropdown	Myocardial Reinfarction	listid: ny<br><br>0. no<br>1. yes	Indicate if patient had myocardial reinfarction during the inhospital course of patient care.<br><br>0=no<br>1=yes<br><br>Source = Patient follow up information / Hospital chart

PREDICT - Hospital Variables

Variable	Type	Caption	List Options	Abstraction Instruction
p_ihclinev mreindt	textbox	Myocardial Reinfarction Date		What was the date of the patient's myocardial reinfarction during the inhospital course of patient care?<br><br>Date value = yyyy/mm/dd<br><br>Source = Patient follow up information / Hospital chart
p_ihclinev mreintm	textbox	Myocardial Reinfarction Time		What was the time of the patient's myocardial reinfarction during the inhospital course of patient care?<br><br>Numerical value based on 24 hour clock<br><br>00:00:00 – hour:min:sec<br><br>If no value for seconds data available – do not do not use value 00<br><br>Source = Patient follow up information / Hospital chart
p_ihclinev tiastr	dropdown	Suspected TIA/Stroke	listid: ny<br><br>0. no<br>1. yes	Indicate if patient had suspected TIA/stroke during the inhospital course of patient care.<br><br>0=no<br>1=yes<br><br>Source = Patient follow up information / Hospital chart
p_ihclinev tiastrdt	textbox	Suspected TIA/Stroke Date		What was the date of the patient's suspected TIA/stroke during the inhospital course of patient care?<br><br>Date value = yyyy/mm/dd<br><br>Source = Patient follow up information / Hospital chart
p_ihclinev tiastrtm	textbox	Suspected TIA/Stroke Time		What was the time of the patient's suspected TIA/stroke during the inhospital course of patient care?<br><br>Numerical value based on 24 hour clock<br><br>00:00:00 – hour:min:sec<br><br>If no value for seconds data available – do not do not use value 00<br><br>Source = Patient follow up information / Hospital chart
p_ihclinev rev	dropdown	Revascularization	listid: ny<br><br>0. no<br>1. yes	Indicate if patient had revascularization during the inhospital course of patient care.<br><br>0=no

Variable	Type	Caption	List Options	Abstraction Instruction
				1=yes<br><br>Source = Patient follow up information / /Hospital chart
p_ihclinev revdt	textbox	Revascularization Date		What was the date of the patient's revascularization procedure during the inhospital course of patient care?<br><br>Date value = yyyy/mm/dd<br><br>Source = Patient follow up information / Hospital chart
p_ihclinev revtm	textbox	Revascularization Time		What was the time of the patient's revascularization procedure during the inhospital course of patient care?<br><br>Numerical value based on 24 hour clock<br><br>00:00:00 – hour:min:sec<br><br>If no value for seconds data available – do not do not use value 00<br><br>Source = Patient follow up information / Hospital chart
p_ihclinev bleed	dropdown	Bleeding	listid: ny<br><br>0. no<br>1. yes	Indicate if patient had bleeding during the inhospital course of patient care.<br><br>0=no<br>1=yes<br><br>Source = Patient follow up information / Hospital chart
p_ihclinev bleeddt	textbox	Bleeding Date		What was the date of the patient's bleeding event during the inhospital course of patient care?<br><br>Date value = yyyy/mm/dd<br><br>Source = Patient follow up information / Hospital chart
p_ihclinev bleedtm	textbox	Bleeding Time		What was the time of the patient's bleeding event during the inhospital course of patient care?<br><br>Numerical value based on 24 hour clock<br><br>00:00:00 – hour:min:sec<br><br>If no value for seconds data available – do not do not use value 00<br><br>Source = Patient follow up information / Hospital chart

Variable	Type	Caption	List Options	Abstraction Instruction
p_ihclinevtransf	dropdown	Transfusion	listid: ny<br><br>0. no<br>1. yes	Indicate if patient had transfusion during the inhospital course of patient care.<br><br>0=no<br>1=yes<br><br>Source = Patient follow up information / Hospital chart
p_ihclinevtransfdt	textbox	Transfusion Date		What was the date of the patient's transfusion procedure during the inhospital course of patient care?<br><br>Date value = yyyy/mm/dd<br><br>Source = Patient follow up information / Hospital chart
p_ihclinevtransftm	textbox	Transfusion Time		What was the time of the patient's transfusion procedure during the inhospital course of patient care?<br><br>Numerical value based on 24 hour clock<br><br>00:00:00 – hour:min:sec<br><br>If no value for seconds data available – do not do not use value 00<br><br>Source = Patient follow up information / Hospital chart
p_ihclinevtempac	dropdown	Temporary Pacemaker	listid: ny<br><br>0. no<br>1. yes	Indicate if patient had temporaty pacemaker inserted during the inhospital course of patient care.<br><br>0=no<br>1=yes<br><br>Source = Patient follow up information / Hospital chart
p_ihclinevtempacdt	textbox	Temporary Pacemaker Date		What was the date when the patient temporaty pacemaker was inserted during the inhospital course of patient care?<br><br>Date value = yyyy/mm/dd<br><br>Source = Patient follow up information / Hospital chart
p_ihclinevtempactm	textbox	Temporary Pacemaker Time		What was the time when the patient temporaty pacemaker was inserted during the inhospital course of patient care?<br><br>Numerical value based on 24 hour clock<br><br>00:00:00 – hour:min:sec

Variable	Type	Caption	List Options	Abstraction Instruction
				If no value for seconds data available – do not do not use value 00<br><br>Source = Patient follow up information / Hospital chart
p_ihclinevcararr	dropdown	Cardiac Arrest	listid: ny<br><br>0. no<br>1. yes	Indicate if patient had cardiac arrest during the inhospital course of patient care.<br><br>0=no<br>1=yes<br><br>Source = Patient follow up information / Hospital chart
p_ihclinevcararrdt	textbox	Cardiac Arrest Date		What was the date of the patient's cardiac arrest during the inhospital course of patient care?<br><br>Date value = yyyy/mm/dd<br><br>Source = Patient follow up information / Hospital chart
p_ihclinevcararrtm	textbox	Cardiac Arrest Time		What was the time of the patient's cardiac arrest during the inhospital course of patient care?<br><br>Numerical value based on 24 hour clock<br><br>00:00:00 – hour:min:sec<br><br>If no value for seconds data available – do not do not use value 00<br><br>Source = Patient follow up information / Hospital chart
p_ihclinevoth	dropdown	Other	listid: ny<br><br>0. no<br>1. yes	Indicate if any other clinical events occurred during the inhospital course of patient care.<br><br>0=no<br>1=yes<br><br>Source = Patient follow up information / Hospital chart
p_ihclinevoths	textbox	Specify		Please specify what other clinical event occurred during the inhospital course of patient care.<br><br>Source = Patient follow up information / Hospital chart
p_ihmed	dropdown	Additional Medications Given in Hospital	listid: nyn<br><br>0. no<br>1. yes	Indicate if any additional medications from the list below were administered to the patient duing the inhospital course of care.<br><br>List of medications:

Variable	Type	Caption	List Options	Abstraction Instruction
			2. not noted	Beta blocker<br>IV vasodilator (other than nitrates)<br>ACE inhibitor<br>ARB t<br>Lipid lowering agent<br>Diuretic<br>IV inotrope<br>Oral anticoagulant (e.g. coumadin/warfarin)<br>Other<br><br>Source = Patient follow up information / Hospital chart
p_ihmedbb	dropdown	Beta Blocker	listid: nyn<br><br>0. no<br>1. yes<br>2. not noted	Indicate if beta blocker was administered to the patient duing the inhospital course of care.<br><br>0=no<br>1=yes<br>2=not noted<br><br>Source = Patient follow up information / Hospital chart
p_ihmedivvas	dropdown	IV Vasodilator (Other Than Nitrates)	listid: nyn<br><br>0. no<br>1. yes<br>2. not noted	Indicate if IV vasodilatoe (other than nitrates) was administered to the patient duing the inhospital course of care.<br><br>0=no<br>1=yes<br>2=not noted<br><br>Source = Patient follow up information / Hospital chart
p_ihmedacei	dropdown	Angiotensin Converting Enzyme (ACE) Inhibitor	listid: nyn<br><br>0. no<br>1. yes<br>2. not noted	Indicate if angiotensin converting enzyme (ACE) inhibitor was administered to the patient duing the inhospital course of care.<br><br>0=no<br>1=yes<br>2=not noted<br><br>Source = Patient follow up information / Hospital chart
p_ihmedarb	dropdown	Angiotensin Receptor Blocker (ARB)	listid: nyn<br><br>0. no<br>1. yes<br>2. not noted	Indicate if angiotensin receptor blocker (ARB) was administered to the patient duing the inhospital course of care.<br><br>0=no<br>1=yes<br>2=not noted

Variable	Type	Caption	List Options	Abstraction Instruction
				Source = Patient follow up information / Hospital chart
p_ihmedlla	dropdown	Lipid Lowering Agent	listid: nyn<br><br>0. no<br>1. yes<br>2. not noted	Indicate if lipid lowering agent was administered to the patient duing the inhospital course of care.<br><br>0=no<br>1=yes<br>2=not noted<br><br>Source = Patient follow up information / Hospital chart
p_ihmeddiu	dropdown	Diuretic	listid: nyn<br><br>0. no<br>1. yes<br>2. not noted	Indicate if diuteric was administered to the patient duing the inhospital course of care.<br><br>0=no<br>1=yes<br>2=not noted<br><br>Source = Patient follow up information / Hospital chart
p_ihmedivino	dropdown	IV Inotrope	listid: nyn<br><br>0. no<br>1. yes<br>2. not noted	Indicate if IV inotrope was administered to the patient duing the inhospital course of care.<br><br>0=no<br>1=yes<br>2=not noted<br><br>Source = Patient follow up information / Hospital chart
p_ihmedoacoa	dropdown	Oral Anticoagulant (e.g. Coumadin/Warfarin)	listid: nyn<br><br>0. no<br>1. yes<br>2. not noted	Indicate if oral anticoagulant (e.g. coumadin/warfarin) was administered to the patient duing the inhospital course of care.<br><br>0=no<br>1=yes<br>2=not noted<br><br>Source = Patient follow up information / Hospital chart
p_ihmedoth	dropdown	Other	listid: nyn<br><br>0. no<br>1. yes<br>2. not noted	Indicate if any other medication was administered to the patient duing the inhospital course of care.<br><br>0=no<br>1=yes<br>2=not noted<br><br>Source = Patient follow up information / Hospital chart

PREDICT - Hospital Variables

Variable	Type	Caption	List Options	Abstraction Instruction
p_ihmedoths	textbox	Specify		Please specify what other medication was administered to the patient duing the inhospital course of care.<br><br>Source = Patient follow up information / Hospital chart
p_ihmhndet_ahg	dropdown	Not Determined	listid: nyn<br><br>0. no<br>1. yes<br><br>2. not noted	Patient Medication History: (from Patient follow up information / Hospital chart<br><br>The Patient follow up information / Hospital chart selection of not determined implies the inhospital staff (nurse/resident/fellow/staff physician) did not ask as opposed to not noted which implies the abstractor could not find the information.
p_ihd	div	Hospital Disposition		
p_ihddt	textbox	Hospital Departure Date		What was the date of the patient hospital departure?<br><br>Date value = yyyy/mm/dd<br><br>Source = Patient follow up information / Hospital chart
p_ihdtm	textbox	Hospital Departure Time		What was the time of the patient hospital departure?<br><br>00:00:00 – hour:min:sec<br><br>Source = Patient follow up information / Hospital chart
p_ihdhome	dropdown	Was Patient Discharged Home?	listid: ny<br><br>0. no<br>1. yes	After admission to hospital was patient then discharged home?<br><br>0=no<br>1=yes<br><br>Source = Patient follow up information / Hospital chart
p_ihptxferah	dropdown	Was Patient Transferred to Another Hospital?	listid: ny<br><br>0. no<br>1. yes	After admission to hospital was patient then transferred to another hospital?<br><br>0=no<br>1=yes<br><br>Source = Patient follow up information / Hospital chart
p_ihtxhosp	dropdown	Transfer Hospital Name	listid: p_hosp<br><br>** See list items in appendix	What is the name of the hospital patient was transferred to?<br><br>Source = Patient follow up information / Hospital chart

PREDICT - Hospital Variables

Variable	Type	Caption	List Options	Abstraction Instruction
				Pulldown menu = list of hospitals in database<br><br>Unknown Hospital = select when destination hospital will never be known.<br><br>NOTE;<br>Do not provide the names of nursing home, rehabilitation, or other non-acute care facilities.<br>Transfer to one of these three entities constitutes an 'hospital discharge, reclassification, or death''.
p_ihptxferpci	dropdown	Was Patient Transferred to PCI Centre?	listid: ny<br><br>0. no<br>1. yes	After admission to hospital was patient then transferred to PCI centre?<br><br>0=no<br>1=yes<br><br>Source = Patient follow up information / Hospital chart
p_ihtxrpci	dropdown	Transfer Hospital Name	listid: p_hosp<br><br>** See list items in appendix	What is the name of the PCI centre patient was transferred to?<br><br>Source = Patient follow up information / PCI/Hospital chart<br><br>Pulldown menu = list of hospitals in database<br><br>Unknown Hospital = select when destination hospital will never be known.<br><br>NOTE;<br>Do not provide the names of nursing home, rehabilitation, or other non-acute care facilities.<br>Transfer to one of these three entities constitutes an 'hospital discharge, reclassification, or death''.

PREDICT - Hospital Variables

Variable	Type	Caption	List Options	Abstraction Instruction
p_ihtrans	dropdown	Transport Mode	listid: trans<br>1. by land<br>2. by air	
p_ihllsh	div	Location and Length of Stay in Hospital		
p_ihccuicu	dropdown	CCU/ICU	listid: ny<br><br>0. no<br>1. yes	Did patient stay at CCU/ICU during the inhospital course of patient care.<br><br>0=no<br>1=yes<br><br>Source = Patient follow up information / Hospital chart
p_ihccuicudays	textbox	CCU/ICU Days		The total number of days patient stayed at CCU/ICU during the inhospital course of patient care.<br><br>Source = Patient follow up information / Hospital chart
p_ihtelward	dropdown	Telemetry Ward	listid: ny<br><br>0. no<br>1. yes	Did patient stay at telemetry ward during the inhospital course of patient care.<br><br>0=no<br>1=yes<br><br>Source = Patient follow up information / Hospital chart
p_ihtelwarddays	textbox	Telemetry Ward Days		The total number of days patient stayed at telemetry ward during the inhospital course of patient care.<br><br>Source = Patient follow up information / Hospital chart
p_ihward	dropdown	Ward	listid: ny<br><br>0. no<br>1. yes	Did patient stay at ward during the inhospital course of patient care.<br><br>0=no<br>1=yes<br><br>Source = Patient follow up information / Hospital chart
p_ihwarddays	textbox	Ward Days		The total number of days patient stayed at ward during the inhospital course of patient care.<br><br>Source = Patient follow up information / Hospital chart
p_ihtotallos	textbox	Total Length of Stay (Total LOS) in Days		The total number of days patient stayed at the hospital.<br><br>Total = discharge – arrival<br><br>Source = Patient follow up information / Hospital chart
p_ihicd10	div	ICD 10 Codes		

PREDICT - Hospital Variables

Variable	Type	Caption	List Options	Abstraction Instruction
p_ihicd10_1	textbox	1		
p_ihicd10_2	textbox	2		
p_ihicd10_3	textbox	3		
p_ihicd10_4	textbox	4		
p_ihicd10_5	textbox	5		
p_ihicd10_6	textbox	6		
p_ihicd10_7	textbox	7		
p_ihicd10_8	textbox	8		
p_ihicd10_9	textbox	9		
p_ihicd10_10	textbox	10		
p_ihicd10_11	textbox	11		
p_ihicd10_12	textbox	12		
p_ihicd10_13	textbox	13		
p_ihicd10_14	textbox	14		
p_ihicd10_15	textbox	15		
p_ihicd10_16	textbox	16		
p_ihstatus	dropdown	Inhospital form status	listid: p_status<br>0. Incomplete<br>1. Complete<br>2. Pending Source Doc(s)<br>3. Not Required - Patient Deceased	
**p_2inhform**	**section**	**2nd Inhospital Form**		
p_2hospital	dropdown	Was Patient Transferred to Hospital?	listid: ny<br>0. no<br>1. yes	
p_2pihih	div	Patient Identifiers		
p_2ilnameih	textbox	Surname		This field is used to obtain the Hospital chart version of the patient's surname.<br><br>Transcribe as-is.<br><br>Caps Lock
p_2ifnameih	textbox	Given Name		This field is used to obtain the Hospital chart version of a patient's given name.<br><br>Transcribe as-is.

PREDICT - Hospital Variables

Variable	Type	Caption	List Options	Abstraction Instruction
				Caps Lock
p_2iaddrih	textbox	Mailing Address		This field is used to obtain the Hospital chart version of a patient's mailing/street address.<br><br>Transcribe as-is.<br><br>Caps Lock
p_2icityih	textbox	City/Town		This field is used to obtain the Hospital chart version of a patient's city / town.<br><br>Transcribe as-is.<br><br>Caps Lock
p_2iprovih	dropdown	Province	listid: prov<br><br>1. AB<br>2. BC<br>3. MB<br>4. NB<br>5. NF<br>6. NS<br>7. NT<br>8. NU<br>9. ON<br>10. PE<br>11. QC<br>12. SK<br>13. YT<br>99. Out of Country	This field is used to obtain the Hospital chart version of a patient's province.<br><br>Transcribe as-is.<br><br>Caps Lock
p_2ipostcih	textbox	Postal Code		This field is used to obtain the Hospital chart version of a patient's postal code.<br><br>Transcribe as-is.<br><br>Caps Lock
p_2idobih	textbox	Date of Birth		This field is used to obtain the Hospital chart version of patient's date of birth (MDY).<br><br>Transcribe as-is.<br><br>Caps Lock
p_2wghtih	dropdown	Weight	listid: uy<br><br>0. unknown/not noted<br><br>1. yes	A This field is used to obtain the Hospital chart version of a patient's weight<br><br>Transcribe as-is

PREDICT - Hospital Variables

Variable	Type	Caption	List Options	Abstraction Instruction
p_2wghtkgih	textbox	Weight(kg)		A This field is used to obtain the Hospital chart version of a patient's weight<br><br>Transcribe as-is
p_2ihospregih	textbox	Registration Number/Patient Chart Number		Hospital chart number from Health Records Department (not from ACR).<br><br>Transcribe as-is.<br><br>Caps Lock
p_2phospregih	textbox	Hospital Registration Number / Patient Chart Number		Hospital Registration Number<br><br>Transcribe as-is.<br><br>Caps Lock<br><br>Note this field will be not applicable for patients who are not transported to an ED or hospital.
p_2ihealthcrd	div	Health Card		
p_2ihealthcnih	textbox	Number		This field is used to obtain the Hospital chart version of Health Card No.<br><br>Transcribe as-is
p_2ihealthcvih	textbox	Version Code		This field is used to obtain the Hospital chart version of Version No.<br><br>Transcribe as-is
p_2isexih	dropdown	Gender	listid: sex<br><br>0. female<br>1. male<br>2. not noted	This field is used to obtain the Hospital chart version of Gender.<br><br>Transcribe as-is
p_2iphone1ih	textbox	Phone 1 (Home)		Patient's home telephone number.<br><br>Transcribe as-is
p_2iphone2ih	textbox	Phone 2		Another contact number for the patient.<br><br>Transcribe as-is
p_2ptrih	div	Hospital Episode/Time Record		
p_2sodtih	textbox	Symptom Onset Date		What was the date of the patient symptom onset?<br><br>Date value = yyyy/mm/dd<br><br>Source = Patient follow up information / Hospital chart
p_2sotmih	textbox	Symptom Onset Time		What was the time of the patient symptom onset?

Variable	Type	Caption	List Options	Abstraction Instruction
				Numerical value based on 24 hour clock<br><br>00:00:00 – hour:min:sec<br><br>If no value for seconds data available – do not do not use value 00.<br><br>Source = Patient follow up information /Hospital chart
p_2ihardt	textbox	Hospital Arrival Date		What was the date of the patient hospital arrival?<br><br>Date value = yyyy/mm/dd<br><br>Source = Patient follow up information / Hospital chart
p_2ihartm	textbox	Hospital Arrival Time		What was the time of the patient hospital arrival?<br><br>Numerical value based on 24 hour clock<br><br>00:00:00 – hour:min:sec<br><br>If no value for seconds data available – do not do not use value 00.<br><br>Source = Patient follow up information / Hospital chart
p_2recihospih	dropdown	Receiving/Hospital	listid: p_hosp<br><br>** See list items in appendix	What is the name of the Receiving Hospital?<br><br>Source = Patient follow up information Hospital chart<br><br>Pulldown menu = list of hospitals in database<br><br>Unknown Hospital = select when destination will never be known.<br><br>NOTE;<br>Do not provide the names of nursing home, rehabilitation, or other non-acute care facilities.<br>Transfer to one of these three entities constitutes an 'hospital discharge, reclassification, or death'.
p_2phxih	dropdown	Patient Past History	listid: nyn<br><br>0. no	Past History: Patient follow up information / Hospital chart

PREDICT - Hospital Variables

Variable	Type	Caption	List Options	Abstraction Instruction
			1. yes<br><br>2. not noted	If no past history is noted on the follow up information / Hospital chart, choose not noted.<br><br>HTNâ€”hypertension<br>Hyperlipidemia<br>Diabetes<br>Renal insufficiency/Failure<br>Chronic renal failure<br>Acute renal failure<br>Dialysis<br>Prior MIâ€”myocardial infarction<br>CADâ€”coronary artery disease<br>CHFâ€”congestive heart failure<br>Prior CABGâ€”coronary artery bypass graph<br>Prior PCI – percutaneous coronary intervention<br>ICDâ€”implantable cardiodefibrillator<br>Pacemaker<br>Peripheral vascular disease<br>DVT – deep venous trombosis<br>Tromboembolic history<br>Pulmonary embolism<br>Stroke/TIA/CVA<br>Smoking<br>Alcohol abuse<br>Cancer<br>Positive family history for heart disease
p_2phihphlt	dropdown	Previously Healthy	listid: nyn<br>0. no<br>1. yes<br>2. not noted	Past History (from Patient follow up information / Hospital chart).<br><br>If no past history is noted on the Patient follow up information / Hospital chart, choose not noted.
p_2phihhyp	dropdown	Hypertension	listid: nyn<br>0. no<br>1. yes<br>2. not noted	Past History (from Patient follow up information / Hospital chart).<br><br>If no past history is noted on the Patient follow up information / Hospital chart, choose not noted.
p_2phihhl	dropdown	Hyperlipidemia	listid: nyn<br>0. no<br>1. yes<br>2. not noted	Past History (from Patient follow up information / Hospital chart).<br><br>If no past history is noted on the Patient follow up information / Hospital chart, choose not noted.
p_2phihdia	dropdown	Diabetes	listid: nyn<br>0. no	Past History (from Patient follow up information / Hospital chart).

Variable	Type	Caption	List Options	Abstraction Instruction
			1. yes<br>2. not noted	If no past history is noted on the Patient follow up information / Hospital chart, choose not noted.
p_2phihdiatype	dropdown	Diabetes Type	listid: diabtyp<br>0. Type 1<br>1. Type 2	Past History (from Patient follow up information / Hospital chart).<br>If no past history is noted on the Patient follow up information / Hospital chart, choose not noted.
p_2phihrif	dropdown	Renal Insufficiency/Failure	listid: nyn<br>0. no<br>1. yes<br>2. not noted	Past History (from Patient follow up information / Hospital chart).<br>If no past history is noted on the Patient follow up information / Hospital chart, choose not noted.
p_2phihcrf	dropdown	Chronic Renal Failure	listid: nyn<br>0. no<br>1. yes<br>2. not noted	Past History (from Patient follow up information / Hospital chart).<br>If no past history is noted on the Patient follow up information / Hospital chart, choose not noted.
p_2phiharf	dropdown	Acute Renal Failure	listid: nyn<br>0. no<br>1. yes<br>2. not noted	Past History (from Patient follow up information / Hospital chart).<br>If no past history is noted on the Patient follow up information / Hospital chart, choose not noted.
p_2phihdial	dropdown	Dialysis	listid: nyn<br>0. no<br>1. yes<br>2. not noted	Past History (from Patient follow up information / Hospital chart).<br>If no past history is noted on the Patient follow up information / Hospital chart, choose not noted.
p_2phihcar	dropdown	Cardiac	listid: nyn<br>0. no<br>1. yes<br>2. not noted	Past History (from Patient follow up information / Hospital chart).<br>If no past history is noted on the Patient follow up information / Hospital chart, choose not noted.
p_2phihmi	dropdown	Prior MI	listid: nyn<br>0. no<br>1. yes<br>2. not noted	Past History (from Patient follow up information / Hospital chart).<br>If no past history is noted on the Patient follow up information / Hospital chart, choose not noted.
p_2phihmidt	textbox	Prior MI Date		What was the date of the patient prior MI?<br>Date value = yyyy/mm/dd

PREDICT - Hospital Variables

Variable	Type	Caption	List Options	Abstraction Instruction
				Source = Patient follow up information / PCI/Hospital chart
p_2phihcad	dropdown	CAD	listid: nyn<br><br>0. no<br>1. yes<br><br>2. not noted	Patient History (from Patient follow uo information / Hospital chart).<br><br>If no past history is noted on the Patient follow up information / Hospital chart, choose not noted.
p_2phihchf	dropdown	CHF	listid: nyn<br><br>0. no<br>1. yes<br><br>2. not noted	Patient History (from Patient follow uo information / Hospital chart).<br><br>If no past history is noted on the Patient follow up information / Hospital chart, choose not noted.
p_2phihcabg	dropdown	Prior CABG	listid: nyn<br><br>0. no<br>1. yes<br><br>2. not noted	Patient History (from Patient follow uo information / Hospital chart).<br><br>If no past history is noted on the Patient follow up information / Hospital chart, choose not noted.
p_2phihcabgdt	textbox	Prior CABG Date		What was the date of the patient prior CABG?<br><br>Date value = yyyy/mm/dd<br><br>Source = Patient follow up information / PCI/Hospital chart
p_2phihpci	dropdown	Prior PCI	listid: nyn<br><br>0. no<br>1. yes<br><br>2. not noted	Past History (from Patient follow up information / Hospital chart).<br><br>If no past history is noted on the Patient follow up information / Hospital chart, choose not noted.
p_2phihpcidt	textbox	Prior PCI Date		What was the date of the patient prior PCI?<br><br>Date value = yyyy/mm/dd<br><br>Source = Patient follow up information / PCI/Hospital chart
p_2phihicd	dropdown	ICD	listid: nyn<br><br>0. no<br>1. yes<br><br>2. not noted	Past History (from Patient follow up information /PCI/Hospital chart).<br><br>If no past history is noted on the Patient follow up information / PCI/Hospital chart, choose not noted.
p_2phihpac	dropdown	Pacemaker	listid: nyn<br><br>0. no	Past History (from Patient follow up information /PCI/Hospital chart).

Variable	Type	Caption	List Options	Abstraction Instruction
			1. yes<br>2. not noted	If no past history is noted on the Patient follow up information / PCI/Hospital chart, choose not noted.
p_2phihpvd	dropdown	Peripheral Vascular Disease	listid: nyn<br>0. no<br>1. yes<br>2. not noted	Past History (from Patient follow up information /PCI/Hospital chart).<br>If no past history is noted on the Patient follow up information / PCI/Hospital chart, choose not noted.
p_2phihdvt	dropdown	DVT	listid: nyn<br>0. no<br>1. yes<br>2. not noted	Past History (from Patient follow up information /PCI/Hospital chart).<br>If no past history is noted on the Patient follow up information / PCI/Hospital chart, choose not noted.
p_2phihteh	dropdown	Tromboembolic History	listid: nyn<br>0. no<br>1. yes<br>2. not noted	Past History (from Patient follow up information /PCI/Hospital chart).<br>If no past history is noted on the Patient follow up information / PCI/Hospital chart, choose not noted.
p_2phihpe	dropdown	Pulmonary Embolism	listid: nyn<br>0. no<br>1. yes<br>2. not noted	Past History (from Patient follow up information /PCI/Hospital chart).<br>If no past history is noted on the Patient follow up information / PCI/Hospital chart, choose not noted.
p_2phihstr	dropdown	Stroke/TIA/CVA	listid: nyn<br>0. no<br>1. yes<br>2. not noted	Past History (from Patient follow up information /PCI/Hospital chart).<br>If no past history is noted on the Patient follow up information / PCI/Hospital chart, choose not noted.
p_2phihstrdt	textbox	Prior Stroke/TIA/CVA Date		What was the date of the patient prior stroke/TIA/CVA?<br>Date value = yyyy/mm/dd<br>Source = Patient follow up information / PCI/Hospital chart
p_2phihsmok	dropdown	Smoking	listid: nyn<br>0. no<br>1. yes<br>2. not noted	Past History (from Patient follow up information /PCI/Hospital chart).<br>If no past history is noted on the Patient follow up information / PCI/Hospital chart, choose not noted.
p_2phicsmok	dropdown	Current Smoker	listid: nyn<br>0. no	Past History (from Patient follow up information /PCI/Hospital chart).

PREDICT - Hospital Variables

Variable	Type	Caption	List Options	Abstraction Instruction
			1. yes<br>2. not noted	If no past history is noted on the Patient follow up information / PCI/Hospital chart, choose not noted.
p_2phihaa	dropdown	Alcohol Abuse	listid: nyn<br>0. no<br>1. yes<br>2. not noted	Past History (from Patient follow up information /PCI/Hospital chart).<br>If no past history is noted on the Patient follow up information / PCI/Hospital chart, choose not noted.
p_2phihcan	dropdown	Cancer	listid: nyn<br>0. no<br>1. yes<br>2. not noted	Past History (from Patient follow up information /PCI/Hospital chart).<br>If no past history is noted on the Patient follow up information / PCI/Hospital chart, choose not noted.
p_2phihpfhhd	dropdown	Positive Family History for Heart Diseases	listid: nyn<br>0. no<br>1. yes<br>2. not noted	Past History (from Patient follow up information /PCI/Hospital chart).<br>If no past history is noted on the Patient follow up information / PCI/Hospital chart, choose not noted.
p_2phihoth	dropdown	Other	listid: nyn<br>0. no<br>1. yes<br>2. not noted	Past History (from Patient follow up information / Hosipital chart)<br>If no past history is noted on the Patient follow up information / Hospital chart, choose not noted.
p_2phihospc	textbox	Specify Other Not Listed		Past History (from Patient follow up information / Hospital chart)
p_2mhxih	dropdown	Patient Past Medication History	listid: nyn<br>0. no<br>1. yes<br>2. not noted	Patient Medication History: Patient follow up information / Hospital chart<br>If no patient medication history is noted on the follow up information / Hospital chart, choose not noted.<br>Beta blockers<br>ASA – acetyl salicylic acid (Aspirin)<br>Fibrinolytics/Trombolytics<br>Clopidogrel (Plavix)/Ticlopidine<br>Glycoprotein (GP) IIb/IIIa inhibitors<br>Heparin<br>Low molecular weight heparin (LMWH)<br>Coumadin<br>Calcium channel blockers<br>Nitrates<br>ARB antagonists<br>ACE inhibitors<br>Lipid lowering drugs

PREDICT - Hospital Variables

Variable	Type	Caption	List Options	Abstraction Instruction
				Digitalis<br>Diuretics<br>Amiodarone<br>Insulin<br>Oral hypoglycemic agents<br>Mucomyst<br>Antidepressants<br>Anxiolytic (anti-anxiety)<br>Estrogens/OCP (oral contraceptive pills)<br>Immunosupressive medication<br>Bronchodilators/Inhaled steroids
p_2mhihno	dropdown	None	listid: nyn<br>0. no<br>1. yes<br>2. not noted	Patient Medication History (Patient follow up information / Hospital chart).<br>If no patient medication history is noted on the Patient follow up information / Hospital chart, choose not noted.
p_2mhihasa	dropdown	ASA (Aspirin)	listid: nyn<br>0. no<br>1. yes<br>2. not noted	Patient Medication History (Patient follow up information / Hospital chart).<br>If no patient medication history is noted on the Patient follow up information / Hospital chart, choose not noted.
p_2mhihfib	dropdown	Fibrinolytics/Trombolytics	listid: nyn<br>0. no<br>1. yes<br>2. not noted	Patient Medication History (Patient follow up information / Hospital chart).<br>If no patient medication history is noted on the Patient follow up information / Hospital chart, choose not noted.
p_2mhihfib type	dropdown	Fibrinolytics/Trombolytics Type	listid: drug<br>1. Streptokinase<br>2. TPA<br>3. TNK<br>4. RPA	Patient Medication History (Patient follow up information / Hospital chart).<br>If no patient medication history is noted on the Patient follow up information / Hospital chart, choose not noted.
p_2mhihclo p	dropdown	Clopidogrel (Plavix)/Ticlopidine	listid: nyn<br>0. no	Patient Medication History (Patient follow up information / Hospital chart).

PREDICT - Hospital Variables

Variable	Type	Caption	List Options	Abstraction Instruction
			1. yes<br>2. not noted	If no patient medication history is noted on the Patient follow up information / Hospital chart, choose not noted.
p_2mhihglycinh	dropdown	Glycoprotein (GP) llb/llla Inhibitors	listid: nyn<br>0. no<br>1. yes<br>2. not noted	Patient Medication History (Patient follow up information / Hospital chart).<br>If no patient medication history is noted on the Patient follow up information / Hospital chart, choose not noted.
p_2mhihhep	dropdown	Heparin	listid: nyn<br>0. no<br>1. yes<br>2. not noted	Patient Medication History (Patient follow up information / Hospital chart).<br>If no patient medication history is noted on the Patient follow up information / Hospital chart, choose not noted.
p_2mhihlmwh	dropdown	Low Molecular Weight Heparin (LMWH)	listid: nyn<br>0. no<br>1. yes<br>2. not noted	Patient Medication History (Patient follow up information / Hospital chart).<br>If no patient medication history is noted on the Patient follow up information / Hospital chart, choose not noted.
p_2mhihbiv	dropdown	Bivalirudin (Angiomax)	listid: nyn<br>0. no<br>1. yes<br>2. not noted	Patient Medication History (Patient follow up information / Hospital chart).<br>If no patient medication history is noted on the Patient follow up information / Hospital chart, choose not noted.
p_2mhihcoum	dropdown	Coumadin	listid: nyn<br>0. no<br>1. yes<br>2. not noted	Patient Medication History (Patient follow up information / Hospital chart).<br>If no patient medication history is noted on the Patient follow up information / Hospital chart, choose not noted.
p_2mhihbb	dropdown	Beta Blockers	listid: nyn<br>0. no<br>1. yes	Patient Medication History (Patient follow up information / Hospital chart).<br>If no patient medication history is noted on the Patient follow up information / Hospital chart, choose not noted.

Variable	Type	Caption	List Options	Abstraction Instruction
			2. not noted	
p_2mhihccb	dropdown	Calcium Channel Blockers	listid: nyn<br>0. no<br>1. yes<br>2. not noted	Patient Medication History (Patient follow up information / Hospital chart).<br>If no patient medication history is noted on the Patient follow up information / Hospital chart, choose not noted.
p_2mhihlant	dropdown	Nitrates/IV/Top	listid: nyn<br>0. no<br>1. yes<br>2. not noted	Patient Medication History (Patient follow up information / Hospital chart).<br>If no patient medication history is noted on the Patient follow up information / Hospital chart, choose not noted.
p_2mhiharba	dropdown	ARB Antagonists	listid: nyn<br>0. no<br>1. yes<br>2. not noted	Patient Medication History (Patient follow up information / Hospital chart).<br>If no patient medication history is noted on the Patient follow up information / Hospital chart, choose not noted.
p_2mhihacei	dropdown	ACE Inhibitors	listid: nyn<br>0. no<br>1. yes<br>2. not noted	Patient Medication History (Patient follow up information / Hospital chart).<br>If no patient medication history is noted on the Patient follow up information / Hospital chart, choose not noted.
p_2mhihlld	dropdown	Lipid Lowering Drugs	listid: nyn<br>0. no<br>1. yes<br>2. not noted	Patient Medication History (Patient follow up information / Hospital chart).<br>If no patient medication history is noted on the Patient follow up information / Hospital chart, choose not noted.
p_2mhihdig	dropdown	Digitalis	listid: nyn<br>0. no<br>1. yes<br>2. not noted	Patient Medication History (Patient follow up information / Hospital chart).<br>If no patient medication history is noted on the Patient follow up information / Hospital chart, choose not noted.
p_2mhihdiu	dropdown	Diuretics	listid: nyn	Patient Medication History (Patient follow up information / Hospital chart).

PREDICT - Hospital Variables

Variable	Type	Caption	List Options	Abstraction Instruction
			0. no<br>1. yes<br>2. not noted	If no patient medication history is noted on the Patient follow up information / Hospital chart, choose not noted.
p_2mhihamio	dropdown	Amiodarone	listid: nyn<br>0. no<br>1. yes<br>2. not noted	Patient Medication History (Patient follow up information / Hospital chart).<br>If no patient medication history is noted on the Patient follow up information / Hospital chart, choose not noted.
p_2mhihins	dropdown	Insulin	listid: nyn<br>0. no<br>1. yes<br>2. not noted	Patient Medication History (Patient follow up information / Hospital chart).<br>If no patient medication history is noted on the Patient follow up information / Hospital chart, choose not noted.
p_2mhihoha	dropdown	Oral Hyoglycemic Agents	listid: nyn<br>0. no<br>1. yes<br>2. not noted	Patient Medication History (Patient follow up information / Hospital chart).<br>If no patient medication history is noted on the Patient follow up information / Hospital chart, choose not noted.
p_2mhihmuc	dropdown	Mucomyst	listid: nyn<br>0. no<br>1. yes<br>2. not noted	Patient Medication History (Patient follow up information / Hospital chart).<br>If no patient medication history is noted on the Patient follow up information / Hospital chart, choose not noted.
p_2mhihantidep	dropdown	Antidepressants	listid: nyn<br>0. no<br>1. yes<br>2. not noted	Patient Medication History (Patient follow up information / Hospital chart).<br>If no patient medication history is noted on the Patient follow up information / Hospital chart, choose not noted.
p_2mhihantianx	dropdown	Anxiolytic (Anti-Anxiety)	listid: nyn<br>0. no	Patient Medication History (Patient follow up information / Hospital chart).

PREDICT - Hospital Variables

Variable	Type	Caption	List Options	Abstraction Instruction
			1. yes<br>2. not noted	If no patient medication history is noted on the Patient follow up information / Hospital chart, choose not noted.
p_2mhihestocp	dropdown	Estrogens/OCP	listid: nyn<br>0. no<br>1. yes<br>2. not noted	Patient Medication History (Patient follow up information / Hospital chart).<br>If no patient medication history is noted on the Patient follow up information / Hospital chart, choose not noted.
p_2mhihimmsup	dropdown	Immunosupressive Medication	listid: nyn<br>0. no<br>1. yes<br>2. not noted	Patient Medication History (Patient follow up information / Hospital chart).<br>If no patient medication history is noted on the Patient follow up information / Hospital chart, choose not noted.
p_2mhihbis	dropdown	Bronchodilators/Inhaled Steroids	listid: nyn<br>0. no<br>1. yes<br>2. not noted	Patient Medication History (Patient follow up information / Hospital chart).<br>If no patient medication history is noted on the Patient follow up information / Hospital chart, choose not noted.
p_2mhihoth	dropdown	Other	listid: nyn<br>0. no<br>1. yes<br>2. not noted	Patient Medication History (Patient follow up information / Hospital chart).<br>If no patient medication history is noted on the Patient follow up information / Hospital chart, choose not noted.
p_2mhihospc	textbox	Specify Other Not Listed		Patient Medication History (Patient follow up information / Hospital chart).
p_2ihmhndet	dropdown	Not Determined	listid: nyn<br>0. no<br>1. yes<br>2. not noted	
p_2ihfib_div	div	Inhospital Medication		
p_2ihfib	dropdown	Inhospital Medication	listid: nynr<br>0. no<br>1. yes<br>2. not recorded	Patient follow up information / Hospital chart<br>0=no<br>1=yes

PREDICT - Hospital Variables

Variable	Type	Caption	List Options	Abstraction Instruction
				2=not noted<br><br>If no PCI fibrinolysis is noted on the Patient follow up information / PCI/Hospital chart, choose not recorded.
p_2ihflytic	dropdown	Fibrinolytic Given	listid: nynr<br><br>0. no<br>1. yes<br>2. not recorded	Did patient receive fibrinolytic therepy during the inhospital course of care?<br><br>0=no<br><br>1=yes<br><br>2=not noted<br><br>Source = Patient follow up information / Hospital chart
p_2ihflyticinel	dropdown	Fibrinolytic Ineligible	listid: ny<br><br>0. no<br>1. yes	Was patient ineligible to receive fibrinolytic therepy during the inhospital course of care?<br><br>0=no<br><br>1=yes<br><br>2=not noted<br><br>Source = Patient follow up information / Hospital chart
p_2ihflyticinelsp	textbox	Specify Reason		Please specify the reason why patient was deemed ineligible to receive fibrinolytic therepy during the inhospital course of care.<br><br>Source = Patient follow up information / Hospital chart
p_2ihflyticstartdt	textbox	Fibrinolysis Start Date		What was the fibrinolysis start date during the inhospital couse of patient care?<br><br>Date value = yyyy/mm/dd

PREDICT - Hospital Variables

Variable	Type	Caption	List Options	Abstraction Instruction
				Source = Patient follow up information / ospital chart
p_2ihflyticstarttm	textbox	Fibrinolysis Start Time		What was the fibrinolysis start time during the inhospital course of patient care?<br><br>Numerical value based on 24 hour clock<br><br>00:00:00 – hour:min:sec<br><br>If no value for seconds data available – do not do not use value 00.<br><br>Source = Patient follow up information / Hospital chart
p_2ihflyticdrug	dropdown	Fibrinolytic Drug Given	listid: drug2<br><br>1. TNK<br>2. tPA (TPA)<br>3. Activase (Alteplase)<br>4. RPA<br>5. Other	Which fibrinolytic drug did patient receive during the inhospital course of care?<br><br>1=TNK (Tenecteplase, Recombinant TPA) no code<br><br>2=TPA (Tissue Plasminogen Activator) Code 661<br><br>3=Activase (Alteplase)<br><br>4=RPA (Reteplase, Retavase) Code 646<br><br>5=other<br><br>Source = Patient follow up information / Hospital chart
p_2ihflyticttotd	textbox	Total Dose Given (mg)		Indicate the total in mg of TNK administered to patient throughout the inhospital course of patient care.<br><br>Source = Patient follow up information / Hospital chart<br><br>TNK dose is usually given with IV administration of 30 mg for patients's weight <60 kg; 35 mg for > 60 to < 70 kg; 40 mg for > 70 to < 80 kg; 45 mg for > 80 to < 90 kg; and 50 mg for > 90 kg.

Variable	Type	Caption	List Options	Abstraction Instruction
p_2ihflyticototd2	textbox	Total Dose Given (mg)		Indicate the total dose of tPA administered to patient during the inhospital course of care.<br><br>Source = Patient follow up information / Hospital chart<br><br>Recommended total tPA dose is basen on patient's weight and for AMI the total dose should not exceed 100 mg. tPA totsl dose is usually given with IV administration of accelerated infusion (1.5 hours): 15 mg IV bolus; then 0.75 mg/kg over next 30 minutes (not to exceed 50 mg); then 0.5 mg/kg over 60 minutes (not to exceed 35 mg).
p_2ihflyticototd3	textbox	Total Dose Given (mg)		Indicate the total in mg of Activase (Alteplase) administered to patient throughout the inhospital course of patient care.<br><br>Source = Patient follow up information / Hospital chart<br><br>Recommended total Activase (Alteplase) dose is based on patient's weight and for AMI the total dose should not exceed 100 mg.<br><br>Activase totsl dose is usually given with IV administration of accelerated infusion (1.5 hours): 15 mg IV bolus for all patients; then: 1) for patients >67 kg 50 mg infused over the next 30 minutes and then 35 mg over the next 60 minutes; 2) for patients ≤67 kg infusion of 0.75 mg/kg over next 30 minutes (not to exceed 50 mg); and then 0.5 mg/kg over 60 minutes (not to exceed 35 mg).<br><br>Activase could also be administered over the 3 hour IV infusion of the total dose of 100 mg: 60 mg in the first hour (bolus dose of 6-10 mg); 20 mg over the second hour and 20 mg over the third hour. For smaller patients (≤65 kg) a dose of 1.25 mg/kg will be administered over the period of 3 hours.
p_2ihflyticototd4	textbox	Total Dose Given (Units)		Indicate the total dose of RPA administered to patient during the inhospital course of care.

PREDICT - Hospital Variables

Variable	Type	Caption	List Options	Abstraction Instruction
				Source = Patient follow up information / Hospital chart<br><br>RPA dose is usually given with IV administration of 10 U bolus over 2 mintes; 30 minutes later give second 10 U IV bolus over 2 minutes (give NS flush before and after each bolus); give heparin and aspirin conjuctively.
p_2ihflytictotd5	textbox	Total Dose Given		Indicate the total of other fibrinolytic drug patient received throughout the inhospital course of patient care.<br><br>Source = Patient follow up information / Hospital chart
p_2ihflyticinter	dropdown	Fibrinolysis Interrupted	listid: ny<br><br>0. no<br>1. yes	Was the fibrinolysis administration interrupted during the inhospital course of patient care?<br><br>0=no<br>1=yes<br><br>Source = Patient follow up information / Hospital chart
p_2ihflyticinrep	dropdown	Fibrinolysis Repeated	listid: ny<br><br>0. no<br>1. yes	Was the fibrinolysis administration repeated during the inhospital course of patient care?<br><br>0=no<br>1=yes<br><br>Source = Patient follow up information / Hospital chart
p_2ihflyticenddt	textbox	Fibrinolysis End Date		What was the fibrinolysis end date during the inhospital course of patient care?<br><br>Date value = yyyy/mm/dd<br><br>Source = Patient follow up information / Hospital chart
p_2ihflyticendtm	textbox	Fibrinolysis End Time		What was the fibrinolysis end time during the inhospital course of patient care?<br><br>Numerical value based on 24 hour clock<br><br>00:00:00 – hour:min:sec<br><br>If no value for seconds data available – do not do not use value 00.

PREDICT - Hospital Variables

Variable	Type	Caption	List Options	Abstraction Instruction
				Source = Patient follow up information / Hospital chart
p_2ihunfhep	dropdown	Unfractionated Heparin Given	listid: nynr<br><br>0. no<br>1. yes<br>2. not recorded	Did patient recive IV Unfractionated Heparin during the inhospital course of care.<br><br>0=no<br><br>1=yes<br><br>2=not noted<br><br>Source = Patient follow up information / Hospital chart<br><br>Indicate if IV Unfractionated Heparin was administered at any time during the inhospital course of care.
p_2ihunfhepibdt	textbox	Initial Bolus Date		The date when administration of IV Unfractionated Heparin initial bolus dose is confirmed during the inhospital course of care.<br><br>Source = Patient follow up information / Hospital chart
p_2ihunfhepibtm	textbox	Initial Bolus Time		The earliest time noted when administration of IV Unfractionated Heparin initial bolus dose is confirmed during the inhospital course of care.<br><br>Source = Patient follow up information / Hospital chart
p_2ihunfhepibdu	textbox	Initial Bolus Dose (Units)		The total in units of IV Unfractionated Heparin initial bolus dose administered to patient throughout the inhospital course of patient care.<br><br>Source = Patient follow up information / Hospital chart<br><br>Unfractionated Heparin initial bolus dose is usually started with IV administration of 60 U/kg to maximum of 4000 U in conjuction with thrombolytics or 80 U/kg to maximum of 5000 U when no trombolytics were administered.

PREDICT - Hospital Variables

Variable	Type	Caption	List Options	Abstraction Instruction
p_2ihunfhepidt	textbox	Infusion Date		The date when administration of IV Unfractionated Heparin infusion is confirmed during the inhospital course of patient care.<br><br>Source = Patient follow up information / Hospital chart
p_2ihunfhepitm	textbox	Infusion Time		The earliest time noted when administration of IV Unfractionated Heparin infusion is confirmed during the inhospital course of patient care.<br><br>Source = Patient follow up information / PCI/Hospital chart
p_2ihunfhepidu	textbox	Infusion Dose (Units)		The total in units of IV Unfractionated Heparin infusion dose administered to patient throughout the inhospital course of patient care.<br><br>Source = Patient follow up information / Hospital chart<br><br>Unfractionated Heparin initial bolus dose is usually started with IV administration of 12 U/kg/hr to maximum of 1000 U/hr in conjuction with thrombolytics or 18 U/kg/hr to maximum of 1500 U/hr when no trombolytics were administered.<br><br>Dose is adjusted to maintain PTT at 1.5 – 2 times control value.
p_2ihenox	dropdown	Enoxaparin Given	listid: nynr<br><br>0. no<br>1. yes<br>2. not recorded	Did patient recive IV Enoxaparin during the inhospital course of patient care?<br><br>0=no<br><br>1=yes<br><br>2=not noted<br><br>Source = Patient follow up information / Hospital chart<br><br>Indicate if IV Unfractionated Heparin was administered at any time during during the inhospital course of patient care.
p_2ihenoini	dropdown	Inital bolus dose given	listid: ny<br><br>0. no	

Variable	Type	Caption	List Options	Abstraction Instruction
			1. yes	
p_2ihenoxdt	textbox	Initial Bolus Date		The date when administration of IV Enoxaparin initial bolus dose is confirmed during the inhospital course of patient care.<br><br>Source = Patient follow up information / Hospital chart
p_2ihenoxtm	textbox	Initial Bolus Time		The earliest time noted when administration of IV Enoxaparin initial bolus dose is confirmed during the inhospital course of patient care.<br><br>Source = Patient follow up information / Hospital chart
p_2ihenoxibdmg	textbox	Initial Bolus Dose (mg)		The total of IV Enoxaparin initial bolus dose administered to patient throughout the inhospital course of patient care.<br><br>Source = Patient follow up information / Hospital chart<br><br>Enoxaparin initial bolus dose is usually started with IV administration of 30 mg for patients < 75 years of age. Patients ≥ 75 years of age do not receive Enoxaparin IV bolus.
p_2ihsceno	dropdown	Subcutoneous (sc) Enoxaparin	listid: ny<br><br>0. no<br>1. yes	
p_2ihenoxidt	textbox	SC injection date		The date when administration of subcutaneous (SC) Enoxaparin infusion is confirmed during the inhospital course of patient care.<br><br>Source = Patient follow up information / Hospital chart
p_2ihenoxitm	textbox	SC injection time		The earliest time noted when administration of SC Enoxaparin infusion is confirmed during the inhospital course of patient care.<br><br>Source = Patient follow up information / Hospital chart
p_2ihenoxidmg	textbox	SC injection dose (mg)		The total of SC Enoxaparin infusion dose administered to patient throughout the inhospital course of patient care.<br><br>Source = Patient follow up information / Hospital chart

PREDICT - Hospital Variables

Variable	Type	Caption	List Options	Abstraction Instruction
				Enoxaparin infusion dose is usually started with SC administration of 1 mg/kg (maximum 100 mg) for patients < 75 years of age and 0.75 mg/kg (maximum 75 mg) for patients $\geq$ 75 years of age.
p_2ihlmwh	dropdown	Other LMWH product given	listid: nynr<br>0. no<br>1. yes<br>2. not recorded	
p_2ihlmwhso	textbox	Specify Other		
p_2ihlmwhft	textbox	First given time		
p_2ihlmwhdose	textbox	Total dose (mg)		
p_2ihclop	dropdown	Clopidogrel (Plavix)	listid: nynr<br>0. no<br>1. yes<br>2. not recorded	Did patient recive Clopidogrel during the inhospital course of patient care?<br>0=no<br>1=yes<br>2=not noted<br>Source = Patient follow up information / Hospital chart<br>Indicate if Clopidogrel was administered at any time during during the inhospital course of patient care.
p_2ihcloptm	textbox	First Given Time		The earliest time noted when administration of Clopidogrel is confirmed during the inhospital course of patient care.<br>Source = Patient follow up information / Hospital chart
p_2ihcloptotd	textbox	Total Dose Given (mg)		Indicate the total in mg of Clopidogrel administered to patient throughout the inhospital course of care.<br>Source = Patient follow up information / Hospital chart

PREDICT - Hospital Variables

Variable	Type	Caption	List Options	Abstraction Instruction
				Clopidogrel dose is usually given with a loading dose of 300 mg for 75 years and under, and 75 mg for 67 years and older. The decision to administer additional clopidogrel to patients is usually left to the discretion of the interventional cardiologist.
p_2ihglycinh	dropdown	Glycoprotein (GP) llb/llla Inhibitors	listid: nynr<br><br>0. no<br>1. yes<br>2. not recorded	Did patient recive IV glycoprotein IIb/IIIa inhibitors during the inhospital course of patient care?<br><br>0=no<br><br>1=yes<br><br>2=not noted<br><br>Source = Patient follow up information / Hospital chart<br><br>Indicate if IV Glycoprotein IIb/IIIa Inhibitors were administered at any time during during the inhsopital course of patient care.
p_2ihglycinhtype	dropdown	Glycoprotein (GP) llb/llla Inhibitors Type	listid: glycinh2<br><br>1. Abciximab (ReoPro)<br><br>2. Eptifibatide (Intergrilin)<br>3. Tirofiban (Aggrastat)<br>4. Other	What type of glycoprotein IIb/IIa inhibitor did patient recive during the inhospital course of patient care?<br><br>1=Abeiximab (ReoPro)<br><br>2=Eptifibatide (Intergrillin)<br><br>3=Tirofiban (Aggrastat)<br>4= other (specify)<br><br>Source = Patient follow up information / Hospital chart<br><br>Indicate what type of Glycoprotein IIb/IIIa Inhibitor was administered at any time during during the inhospital course of patient care.
p_2ihglycinhtypesp	textbox	Specify Other		Please specify which other glycoprotein IIb/IIa inhibitor patient received during the inhospital course of patient care.

Variable	Type	Caption	List Options	Abstraction Instruction
				Source = Patient follow up information / Hospital chart
p_2ihglycinhtm	textbox	First Given Time		The earliest time noted when administration of glycoprotein IIb/IIa inhibitor is confirmed during the inhospital course of patient care.<br><br>Source = Patient follow up information / Hospital chart
p_2ihglycinhtotd	textbox	Total Dose Given (mg)		Indicate the total dose of Abeiximab administered to patient during the inhospital course of care.<br><br>Source = Patient follow up information / Hospital chart<br><br>Abeiximab dose is usually ggiven with IV bolus administration of 0.25 mg/kg 10-60 minutes before the start of PCI, followed by a continuous intravenous infusion of 0.125 Âµg/kg/min (to a maximum of 10 Âµg/min) for 12 hours.
p_2ihglycinhtotd2	textbox	Total Dose Given (mg)		Indicate the total dose of Eptifibatide administered to patient during the inhospital course of care.<br><br>Source = Patient follow up information / Hospital chart<br><br>Eptifibatide dose is usually given with IV bolus administration of 180 Âµg/kg as soon as possible followed by a continuous infusion of 2.0 Âµg/kg/min until hospital discharge or initiation of CABG surgery, up to 72 hours. If a patient is to undergo a percutaneous coronary intervention (PCI) while receiving eptifibatide, the infusion should be continued up to hospital discharge, or for up to 18 to 24 hours after the procedure, whichever comes first, allowing for up to 96 hours of therapy .
p_2ihglycinhtotd3	textbox	Total Dose Given (mg)		Indicate the total dose of Tirofiban administered to patient during the inhospital course of care.<br><br>Source = Patient follow up information / Hospital chart<br><br>Tirofiban dose is usually given with IV at an initial rate of 0.4 mcg/kg/min for 30 minutes and then continued at 0.1 mcg/kg/min.

PREDICT - Hospital Variables

Variable	Type	Caption	List Options	Abstraction Instruction
p_2ihglycinhtotd4	textbox	Total Dose Given (mg)		Indicate the total in mg of the other of glycoprotein IIb/IIa inhibitor administered to patient throughout the inhospital course of care.<br><br>Source = Patient follow up information / Hospital chart
p_2ihtrombinh	dropdown	Thrombin Inhibitors	listid: nynr<br><br>0. no<br>1. yes<br>2. not recorded	Did patient recive trombin inhibitors during the inhospital course of patient care?<br><br>0=no<br>1=yes<br>2=not noted<br><br>Source = Patient follow up information / Hospital chart<br><br>Indicate if trombin inhibitors were administered at any time during during the inhospital course of patient care.
p_2ihtrombinhtype	dropdown	Thrombin Inhibitors Type	listid: trombinh<br><br>1. Bivalirudin<br>2. Lepirudin<br>3. Desirudin<br>4. Other	What type of trombin inhibitor did patient recive during the inhospital course of patient care?<br><br>=Bivalirudin (Angiomax)<br>2=Lepirudin (Refludan)<br>3=Desirudin<br>4=Other<br><br>Source = Patient follow up information / PCI/Hospital chart

PREDICT - Hospital Variables

Variable	Type	Caption	List Options	Abstraction Instruction
				Indicate what type of trombin inhibitor administered at any time during during the inhospital course of patient care.
p_2ihtromb inhtm	textbox	First Given Time		The earliest time noted when administration of trombin inhibitor is confirmed during the inhospital course of patient care.<br><br>Source = Patient follow up information / Hospital chart
p_2ihtromb inhtotd	textbox	Total Dose Given (mg)		Indicate the total in mg of Desirudin administered to patient throughout the inhospital course of care.<br><br>Source = Patient follow up information / Hospital chart
p_2ihtromb inhtotd2	textbox	Total Dose Given (mg)		*Indicate the total in mg of Desirudin administered to patient throughout the inhospital course of care.*<br><br>*Source = Patient follow up information / Hospital chart*
p_2ihtromb inhtotd3	textbox	Total Dose Given (mg)		Indicate the total in mg of other trombin inhibitor administered to patient throughout the inhospital course of care.<br><br>Source = Patient follow up information / Hospital chart
p_2ihasa2	dropdown	ASA (Aspirin) Given	listid: nynr<br><br>0. no<br>1. yes<br>2. not recorded	Indicate if ASA was administered at any time during the inhospital course of patient care.<br><br>0=no<br><br>1=yes<br><br>2=not noted<br><br>Source = Patient follow up information / Hospital chart

PREDICT - Hospital Variables

Variable	Type	Caption	List Options	Abstraction Instruction
p_2ihasaftm	textbox	First Given Time		The earliest time noted when administration of ASA is confirmed during the inhospital course of patient care.<br><br>Source = Patient follow up information / Hospital chart
p_2ihasamg	textbox	Total Dose (mg)		The total in mg of all doses of ASA administered to patient throughout the inhospital course of care.<br><br>Â· Usual dose of ASA is 160 mg (2x80 mg) for the patient to chew and swallow.<br><br>Source = Patient follow up information / Hospital chart
p_2ihnitro2	dropdown	Nitrolingual Spray Given	listid: nynr<br><br>0. no<br>1. yes<br>2. not recorded	Indicate if Nitrolingual spray 0.4 mg/spray was administered at any time during the inhospital course of patient care.<br><br>0=no<br><br>1=yes<br><br>2=not noted<br><br>Source = Patient follow up information / Hospital chart
p_2ihnitro2ftm	textbox	First Given Time		
p_2ihnitro2mg	textbox	Total Dose(mg)		
p_2ihnitropatch	dropdown	Nitro Patch	listid: ny<br><br>0. no<br>1. yes	
p_2ihnitroftm	textbox	First Given Time		The earliest time noted when administration of Nitrolingual spray 0.4 mg/spray is confirmed during the inhospital course of patient care.<br><br>Source = Patient follow up information / Hospital chart
p_2ihnitromg	textbox	Total Dose (mg)		The total in mg of all doses of Nitrolingual spray administered to patient throughout the inhospital course of care.

PREDICT - Hospital Variables

Variable	Type	Caption	List Options	Abstraction Instruction
				Â· Maximal number of Nitrolingual spray 0.4 mg/spray is 3; single spray is administered every 5 minutes.<br><br>Source = Patient follow up information / Hospital chart
p_2ihnitrodrip	dropdown	Nitro IV Drip Given	listid: nynr<br><br>0. no<br>1. yes<br>2. not recorded	Indicate if Nitro IV drip was administered at any time during the inhospital course of patient care.<br><br>0=no<br><br>1=yes<br><br>2=not noted<br><br>Source = Patient follow up information / Hospital chart
p_2ihnitrodripftm	textbox	First Given Time		The earliest time noted when administration of Nitro IV drip during the inhospital course of patient care.<br><br>Source = Patient follow up information / Hospital chart
p_2ihnitromg2	textbox	Total Dose (Âµg/min)		The total in mg of Nitro IV drip administered to patient throughout the inhospital course of care.<br><br>Â· Nitro drip is usually started with IV administration of 0.1 Âµg/kg/min and then tittred to up to 200 Âµg/min.<br><br>Source = Patient follow up information / Hospital chart
p_2ihlabtest_div	div	Inhospital Lab and Cardiac Enzymes		
p_2ihlabtest	dropdown	Were Lab Tests Done during inhospital stay?	listid: ny<br><br>0. no<br>1. yes	Were lab tests done during the inhospital course of patient care?<br><br>0=no<br>1=yes<br><br>Source = Patient follow up information / Hospital chart<br><br>Indicate if lab tests were done at any time during the inhospital course of patient care.
p_2ihlabtestdt	textbox	Lab Tests Date		The date when the lab tests were done first time during the inhospital course of patient care.

Variable	Type	Caption	List Options	Abstraction Instruction
				Source = Patient follow up information / Hospital chart
p_2ihlabtesttm	textbox	Lab Tests Time		The time when the lab tests were done first time during the inhospital course of patient care.<br><br>Source = Patient follow up information / Hospital chart
p_2ihchol	dropdown	Cholesterol	listid: ny<br><br>0. no<br>1. yes	Indicate if cholesterol was measured during the inhospital course of patient care?<br><br>0=no<br>1=yes<br><br>Source = Patient follow up information / Hospital chart
p_2ihchollev	textbox	Cholesterol Levels (mmol/L)		Indicate the first cholesterol levels measured during the inhospital course of patient care.<br><br>Source = Patient follow up information / Hospital chart
p_2ihhdl	dropdown	HDL	listid: ny<br><br>0. no<br>1. yes	Indicate if HDL was measured during the inhospital course of patient care?<br><br>0=no<br>1=yes<br><br>Source = Patient follow up information / Hospital chart
p_2ihhdllev	textbox	HDL Levels		Indicate the first HDL levels measured during the inhospital course of patient care.<br><br>Source = Patient follow up information / Hospital chart
p_2ihldl	dropdown	LDL	listid: ny<br><br>0. no<br>1. yes	Indicate if LDL was measured during the inhospital course of patient care?<br><br>0=no<br>1=yes<br><br>Source = Patient follow up information / Hospital chart
p_2ihldllev	textbox	LDL Levels		Indicate the first LDL levels measured during the inhospital course of patient care.<br><br>Source = Patient follow up information / Hospital chart
p_2ihtriglyc	dropdown	Triglycerides	listid: ny<br><br>0. no<br>1. yes	Indicate if tryglicerides were measured during the inhospital course of patient care?<br><br>0=no

PREDICT - Hospital Variables

Variable	Type	Caption	List Options	Abstraction Instruction
				1=yes<br><br>Source = Patient follow up information / Hospital chart
p_2ihtriglyclev	textbox	Triglyercrides Levels (mmol/L)		Indicate the first tryglicerides levels measured during the inhospital course of patient care.<br><br>Source = Patient follow up information / Hospital chart
p_2ihgluc	dropdown	Glucose	listid: ny<br><br>0. no<br>1. yes	Indicate if glucose was measured during the inhospital course of patient care?<br><br>0=no<br>1=yes<br><br>Source = Patient follow up information / Hospital chart
p_2ihgluclev	textbox	Glucose Levels		Indicate the first glucose levels measured during the inhospital course of patient care.<br><br>Source = Patient follow up information / Hospital chart
p_2ihcreat	dropdown	Creatinine	listid: ny<br><br>0. no<br>1. yes	Indicate if creatinine was measured during the inhospital course of patient care?<br><br>0=no<br>1=yes<br><br>Source = Patient follow up information / Hospital chart
p_2ihcreatlev	textbox	Creatinine Levels		Indicate the first creatinine levels measured during the inhospital course of patient care.<br><br>Source = Patient follow up information / Hospital chart
p_2ihcaren	dropdown	Were Cardiac Enzymes Done During Inhospital Stay?	listid: ny<br><br>0. no<br>1. yes	Were cardiac enzymes done during the inhospital course of patient care?<br><br>0=no<br>1=yes<br><br>Source = Patient follow up information / Hospital chart<br><br>Indicate if cardiac enzymes were done at any time during the inhospital course of patient care.

PREDICT - Hospital Variables

Variable	Type	Caption	List Options	Abstraction Instruction
p_2ihfcarendt	textbox	First Cardiac Enzymes Measurement Date		The date when the cardiac enzymes were measured first time during the inhospital course of patient care.<br><br>Source = Patient follow up information / Hospital chart
p_2ihfcarentm	textbox	First Cardiac Enzymes Measurement Time		The time when the cardiac enzymes were measured first time during the inhospital course of patient care.<br><br>Source = Patient follow up information / Hospital chart
p_2ihfck	dropdown	First Creatine Kinase (CK)	listid: ny<br><br>0. no<br>1. yes	Indicate if first CK levels were measured during the inhospital course of patient care?<br><br>0=no<br>1=yes<br><br>Source = Patient follow up information / Hospital chart
p_2ihfcklev	textbox	First CK Levels (U/L)		Indicate the first CK levels measured during the inhospital course of patient care.<br><br>Source = Patient follow up information / Hospital chart
p_2ihfckmb	dropdown	First Creatine Kinase - MB (CK-MB)	listid: ny<br><br>0. no<br>1. yes	Indicate if first CK-MB levels were measured during the inhospital course of patient care?<br><br>0=no<br>1=yes<br><br>Source = Patient follow up information / Hospital chart
p_2ihfckmblev	textbox	First CK-MB Levels (U/L)		Indicate the first CK-MB levels measured during the inhospital course of patient care.<br><br>Source = Patient follow up information / Hospital chart
p_2ihftropi	dropdown	First Troponin I	listid: ny<br><br>0. no<br>1. yes	Indicate if first Troponin I levels were measured during the inhospital course of patient care?<br><br>0=no<br>1=yes<br><br>Source = Patient follow up information / Hospital chart
p_2ihftropilev	textbox	First Troponin I Levels (Âµg/L)		Indicate the first Troponin I levels measured during the inhospital course of patient care.<br><br>Source = Patient follow up information / Hospital chart

PREDICT - Hospital Variables

Variable	Type	Caption	List Options	Abstraction Instruction
p_2ihftropt	dropdown	First Troponin T	listid: ny<br><br>0. no<br>1. yes	Indicate if first Troponin T levels were measured during the inhospital course of patient care?<br><br>0=no<br>1=yes<br><br>Source = Patient follow up information / Hospital chart
p_2ihftroptlev	textbox	First Troponin T Levels (Âµg/L)		Indicate the first Troponin T levels measured during the inhospital course of patient care.<br><br>Source = Patient follow up information / Hospital chart
p_2ihpcarendt	textbox	Peak Cardiac Enzymes Measurement Date		The date when the peak cardiac enzymes were measured during the inhospital course of patient care.<br><br>Source = Patient follow up information / Hospital chart
p_2ihpcarentm	textbox	Peak Cardiac Enzymes Measurement Time		The time when the peak cardiac enzymes were measured during the inhospital course of patient care.<br><br>Source = Patient follow up information / Hospital chart
p_2ihpck	dropdown	Peak Creatine Kinase (CK)	listid: ny<br><br>0. no<br>1. yes	Indicate if peak CK levels were measured during the inhospital course of patient care?<br><br>0=no<br>1=yes<br><br>Source = Patient follow up information / Hospital chart
p_2ihpcklev	textbox	Peak CK Levels (U/L)		Indicate the peak CK levels measured during the inhospital course of patient care.<br><br>Source = Patient follow up information / Hospital chart
p_2ihpckmb	dropdown	Peak Creatine Kinase - MB (CK-MB)	listid: ny<br><br>0. no<br>1. yes	Indicate the peak CK-MB levels measured during the inhospital course of patient care.<br><br>Source = Patient follow up information / Hospital chart
p_2ihpckmblev	textbox	Peak CK-MB Levels (U/L)		Indicate the peak CK-MB levels measured during the inhospital course of patient care.<br><br>Source = Patient follow up information / Hospital chart

PREDICT - Hospital Variables

Variable	Type	Caption	List Options	Abstraction Instruction
p_2ihptropi	dropdown	Peak Troponin I	listid: ny<br><br>0. no<br>1. yes	Indicate if peak Troponin I levels were measured during the inhospital course of patient care?<br><br>0=no<br>1=yes<br><br>Source = Patient follow up information / Hospital chart
p_2ihptropilev	textbox	Peak Troponin I Levels (Âµg/L)		Indicate the peak Troponin I levels measured during the inhospital course of patient care.<br><br>Source = Patient follow up information / Hospital chart
p_2ihptropt	dropdown	Peak Troponin T	listid: ny<br><br>0. no<br>1. yes	Indicate if peak Troponin T levels were measured during the inhospital course of patient care?<br><br>0=no<br>1=yes<br><br>Source = Patient follow up information / Hospital chart
p_2ihptroptlev	textbox	Peak Troponin T Levels (Âµg/L)		Indicate the peak Troponin T levels measured during the inhospital course of patient care.<br><br>Source = Patient follow up information / Hospital chart
p_2ihecg_div	div	ECG Recording in Hospital		
p_2ihecg	dropdown	Does Inhospital ECG Recording Exist?	listid: ny<br><br>0. no<br>1. yes	Did hospital personnel record the patient ECG?<br><br>0=no<br>1=yes<br><br>Indicate no or yes, whether inhospital ECG recording exists.<br><br>Source = Patient follow up information / Hospital chart
p_2ihecgrec	dropdown	How many ECG Recordings (total number)	listid: 3plus<br><br>1. one<br>2. two<br>3. three<br>4. more than three	How many recordings of the ECG process (total number) were made in hospital?<br><br>1=one<br><br>2=two<br><br>3=three<br><br>4=more than three

PREDICT - Hospital Variables

Variable	Type	Caption	List Options	Abstraction Instruction
				Source = Patient follow up information / Hospital chart
p_2ihecgrecnum	textbox	How many?		
p_2fihecg_div	div	First Inhospital ECG (first one on arrival in hospital)		
p_2fihecg	dropdown	Does First Inhospital ECG Recording Exist?	listid: ny<br>0. no<br>1. yes	Did hospital personnel record the patient ECG on arrival in hospital?<br>0=no<br>1=yes<br>Indicate no or yes, whether first inhospital ECG recording exists.<br>Source = Patient follow up information / Hospital chart
p_2fihecgdt	textbox	Date		What was the date of the patient first ECG in hospital?<br>Date value = yyyy/mm/dd<br>Source = Patient follow up information / Hospital chart
p_2fihecgtm	textbox	Time		What was the time of the patient first ECG in hospital?<br>Numerical value based on 24 hour clock<br>00:00:00 – hour:min:sec<br>If no value for seconds data available – do not do not use value 00<br>Source = Patient follow up information / Hospital chart
p_2fihecgrev	dropdown	First ECG Reviewed	listid: nyu<br>0. no<br>1. yes<br>2. unknown	Indicate whether or not the first inhospital ECG was reviewed by the attending hospital physician site or if the attendant annotations and QA data was generated solely by the device software.<br>0=no<br>1=yes<br>2=unknown<br>The goal is for sites to review the recordings so as to 'correct' any oversights by the software.<br>Source = Patient follow up information / Hospital chart

PREDICT - Hospital Variables

Variable	Type	Caption	List Options	Abstraction Instruction
p_2fihecgd	dropdown	First ECG Data Exists	listid: ny<br><br>0. no<br>1. yes	Is there the first inhospital ECG data available on request?<br><br>0=no<br>1=yes<br><br>Source = Patient follow up information / Hospital chart
p_2fihecgdgca	dropdown	First ECG Diagnosis - Computer Assisted	listid: ny<br><br>0. no<br>1. yes	Indicate whether or not there was computer assisted diagnosis for the first inhospital ECG.<br><br>0=no<br>1=yes<br><br>Source = Patient follow up information / Hospital chart
p_2fihecgdgsp	textbox	First ECG Diagnosis - Computer Assisted - Specify 1		Please specify computer assisted diagnosis for the first inhospital ECG.<br><br>Value = text<br><br>Source = Patient follow up information / Hospital chart
p_2fihecgdgsp_2	textbox	First ECG Diagnosis - Computer Assisted - Specify 2 (cont)		
p_2fihecgdgsp_3	textbox	First ECG Diagnosis - Computer Assisted - Specify 3 (cont)		
p_2fihecgdg	textbox	First ECG Diagnosis 1		Indicate the diagnosis based on patient first inhospital ECG made by hospital attending physician.<br><br>Source = Patient follow up information / Hospital chart
p_2fihecgdg_2	textbox	First ECG Diagnosis 2 (cont)		
p_2fihecgdg_3	textbox	First ECG Diagnosis 3 (cont)		
p_2stefihecg	dropdown	ST Elevation	listid: nyn<br><br>0. no<br>1. yes<br>2. not noted	Is there ST elevation on patient first inhospital ECG?<br><br>0=no<br>1=yes<br><br>Source = Patient follow up information / Hospital chart
p_2stemicrfihecg	dropdown	STEMI Criteria Met	listid: nynr<br><br>0. no<br>1. yes<br>2. not recorded	Is ST Elevation Myocardial Infarction (STEMI) criteria met on patient first inhospital ECG?<br><br>0=no

PREDICT - Hospital Variables

Variable	Type	Caption	List Options	Abstraction Instruction
				1=yes<br><br>2=not recorded<br><br>Source = Patient follow up information / Hospital chart
p_2stemilfihecg	textbox	STEMI Location		Indicate, if possible the STEMI location based on patient first ECG in hsopital.<br><br>Source = Patient follow up information / Hospital chart
p_2ihecg6h_div	div	Inhospital ECG (5 hours after arrival in hospital)		
p_2ihecg6h	dropdown	Does Inhospital ECG Recording Exist (6 hours after arrival)?	listid: ny<br><br>0. no<br>1. yes	Did hospital personnel record the patient ECG 6 hours after arrival in hospital<br><br>0=no<br>1=yes<br><br>Indicate no or yes, whether 6 hour inhospital ECG recording exists.<br><br>Source = Patient follow up information / Hospital chart
p_2ihecg6hdt	textbox	Date		What was the date of the patient ECG recorded 6 hours after arrival in hospital?<br><br>Date value = yyyy/mm/dd<br><br>Source = Patient follow up information / Hospital chart
p_2ihecg6htm	textbox	Time		What was the time of the patient ECG recorded 6 hours after arrival in hospital?<br><br>Numerical value based on 24 hour clock<br><br>00:00:00 – hour:min:sec<br><br>If no value for seconds data available – do not do not use value 00<br><br>Source = Patient follow up information / Hospital chart
p_2ihecg6hrev	dropdown	Inhospital ECG (6 hours) Reviewed	listid: nyu	Indicate whether or not the patient ECG recorded 6 hours after arrival in hospital was reviewed by the attending hospital hysician site or if the attendant annotations and QA data was generated solely by the device software.

Variable	Type	Caption	List Options	Abstraction Instruction
			0. no<br>1. yes<br>2. unknown	0=no<br>1=yes<br>2=unknown<br><br>The goal is for sites to review the recordings so as to 'correct' any oversights by the software.<br>Source = Patient follow up information / Hospital chart
p_2ihecg6hd	dropdown	Inhospital ECG (6 hours) Data Exists	listid: ny<br><br>0. no<br>1. yes	Is there data available on request for the patient ECG recorded 6 hours after arrival in hospital?<br><br>0=no<br>1=yes<br><br>Source = Patient follow up information / Hospital chart
p_2ihecg6hdgca	dropdown	Inhospital ECG (6 hours) Diagnosis - Computer Assisted	listid: ny<br><br>0. no<br>1. yes	Indicate whether or not there was computer assisted diagnosis for the patient ECG recorded 6 hours after arrival in hospital.<br><br>0=no<br>1=yes<br><br>Source = Patient follow up information / Hospital chart
p_2ihecg6hdgsp	textbox	Inhospital ECG (6 hours) Diagnosis - Computer Assisted - Specify 1		Please specify computer assisted diagnosis for the patient ECG recorded 6 hours after arrival in hospital.<br><br>Value = text<br><br>Source = Patient follow up information / Hospital chart
p_2ihecg6hdgsp_2	textbox	Inhospital ECG (6 hours) Diagnosis - Computer Assisted - Specify 2 (cont)		
p_2ihecg6hdgsp_3	textbox	Inhospital ECG (6 hours) Diagnosis - Computer Assisted - Specify 3 (cont)		
p_2ihecg6hg	textbox	Inhospital ECG (6 hours) Diagnosis 1		Indicate the diagnosis based on patient ECG recorded 6 hours after arrival in hospital made by hospital attending physician.<br><br>Source = Patient follow up information / Hospital chart
p_2ihecg6hg_2	textbox	Inhospital ECG (6 hours) Diagnosis 2 (cont)		

Variable	Type	Caption	List Options	Abstraction Instruction
p_2ihecg6hg_3	textbox	Inhospital ECG (6 hours) Diagnosis 3 (cont)		
p_2steihecg6h	dropdown	ST Elevation	listid: nyn<br>0. no<br>1. yes<br>2. not noted	Is there ST elevation on patient ECG recorded 6 hours after arrival in hospital?<br>0=no<br>1=yes<br>Source = Patient follow up information / Hospital chart
p_2stemicrihecg6h	dropdown	STEMI Criteria Met	listid: nynr<br>0. no<br>1. yes<br>2. not recorded	Is ST Elevation Myocardial Infarction (STEMI) criteria met on patient ECG recorded 6 hours after arrival in hospital?<br>0=no<br>1=yes<br>2=not noted<br>Source = Patient follow up information / Hospital chart
p_2stemilihecg6h	textbox	STEMI Location		Indicate, if possible the STEMI location based on patient ECG recorded 6 hours after arrival in hospital.<br>Source = Patient follow up information / PCI/Hospital chart
p_2ihecg6hredcp	dropdown	Reduction in the chest pain intensity	listid: nyn<br>0. no<br>1. yes<br>2. not noted	Indicate, if possible, if there is a significant reduction in the patient chest pain intensity during the 6 hour inhospital period.<br>0=no<br>1=yes<br>2=not noted<br>Source = Patient follow up information / Hospital chart
p_2ihecg6hrestemi	dropdown	Reduction in ST-segment elevation (i.e. > 50%)	listid: nyn<br>0. no<br>1. yes<br>2. not noted	Indicate, if possible, if there is a significant reduction in ST-segment elevation (i.e. > 50%) on patient ECG recorded 6 hours after arrival in hospital.<br>0=no<br>1=yes

PREDICT - Hospital Variables

Variable	Type	Caption	List Options	Abstraction Instruction
				2=not noted<br><br>Source = Patient follow up information / Hospital chart
p_2ihecg24h_div	div	Inhospital ECG (24 hours after arrival)?		
p_2ihecg24h	dropdown	Does Inhospital ECG Recording Exist (24 hours after arrival in hospital)?	listid: ny<br><br>0. no<br>1. yes	Did hospital personnel record the patient ECG 24 hours after arrival in hospital<br><br>0=no<br>1=yes<br><br>Indicate no or yes, whether 24 hour inhospital ECG recording exists.<br><br>Source = Patient follow up information / Hospital chart
p_2ihecg24hdt	textbox	Date		What was the date of the patient ECG recorded 24 hours after arrival in hospital?<br><br>Date value = yyyy/mm/dd<br><br>Source = Patient follow up information / Hospital chart
p_2ihecg24htm	textbox	Time		What was the time of the patient ECG recorded 24 hours after arrival in hospital?<br><br>Numerical value based on 24 hour clock<br><br>00:00:00 – hour:min:sec<br><br>If no value for seconds data available – do not do not use value 00<br><br>Source = Patient follow up information / Hospital chart
p_2ihecg24hrev	dropdown	Inhospital ECG (24 hours) Reviewed	listid: nyu<br><br>0. no<br>1. yes<br>2. unknown	Indicate whether or not the 24 hour inhospital ECG was reviewed by the attending hospital hysician site or if the attendant annotations and QA data was generated solely by the device software.<br><br>0=no<br>1=yes<br>2=unknown<br><br>The goal is for sites to review the recordings so as to 'correct' any oversights by the software.<br>Source = Patient follow up information / Hospital chart

PREDICT - Hospital Variables

Variable	Type	Caption	List Options	Abstraction Instruction
p_2ihecg24hd	dropdown	Inhospital ECG (24 hours) Data Exists	listid: ny<br><br>0. no<br>1. yes	Is there data available on request for the patient ECG recorded 24 hours after arrival in hospital?<br><br>0=no<br>1=yes<br><br>Source = Patient follow up information / Hospital chart
p_2ihecg24hdgca	dropdown	Inhospital ECG (24 hours) Diagnosis - Computer Assisted	listid: ny<br><br>0. no<br>1. yes	Indicate whether or not there was computer assisted diagnosis for the patient ECG recorded 24 hours after arrival in hospital.<br><br>0=no<br>1=yes<br><br>Source = Patient follow up information / Hospital chart
p_2ihecg24hdgsp	textbox	Inhospital ECG (24 hours) Diagnosis - Computer Assisted - Specify 1		Please specify computer assisted diagnosis for the patient ECG recorded 24 hours after arrival in hospital.<br><br>.<br><br>Value = text<br><br>Source = Patient follow up information / Hospital chart
p_2ihecg24hdgsp_2	textbox	Inhospital ECG (24 hours) Diagnosis - Computer Assisted - Specify 2 (cont)		
p_2ihecg24hdgsp_3	textbox	Inhospital ECG (24 hours) Diagnosis - Computer Assisted - Specify 3 (cont)		
p_2ihecg24hdg	textbox	Inhospital ECG (24 hours) Diagnosis 1		Indicate the diagnosis based on patient ECG recorded 24 hours after arrival in hospital.<br><br>made by hospital attending physician.<br><br>Source = Patient follow up information / Hospital chart
p_2ihecg24hdg_2	textbox	Inhospital ECG (24 hours) Diagnosis 2 (cont)		
p_2ihecg24hdg_3	textbox	Inhospital ECG (24 hours) Diagnosis 3 (cont)		
p_2steihecg24h	dropdown	ST Elevation	listid: nyn<br><br>0. no<br>1. yes<br>2. not noted	Is there ST elevation on patient ECG recorded 24 hours after arrival in hospital?<br><br>0=no<br>1=yes

Variable	Type	Caption	List Options	Abstraction Instruction
				Source = Patient follow up information / Hospital chart
p_2stemicrihecg24h	dropdown	STEMI Criteria Met	listid: nynr<br>0. no<br>1. yes<br>2. not recorded	Is ST Elevation Myocardial Infarction (STEMI) criteria met on patient ECG recorded 24 hours after arrival in hospital?<br>0=no<br>1=yes<br>2=not noted<br>Source = Patient follow up information / Hospital chart
p_2stemilihecg24h	textbox	STEMI Location		Indicate, if possible the STEMI location based on patient ECG recorded 24 hours after arrival in hospital.<br>Source = Patient follow up information / PCI/Hospital chart
p_2ihecg24hredecg	dropdown	Reduction in the chest pain intensity	listid: nyu<br>0. no<br>1. yes<br>2. unknown	Indicate, if possible, if there is a significant reduction in the patient's chest pain intensity during the 24 hour inhospital period.<br>0=no<br>1=yes<br>2=not noted<br>Source = Patient follow up information / Hospital chart
p_2ihecg24hredstat	dropdown	Reduction in ST-Segment elevation (i.e. > 50%)	listid: nyn<br>0. no<br>1. yes<br>2. not noted	Indicate, if possible, if there is a significant reduction in ST-segment elevation (i.e. > 50%) on patient ECG recorded 24 hours after arrival in hospital.<br>0=no<br>1=yes<br>2=not noted<br>Source = Patient follow up information / Hospital chart
p_2ihecg48h_div	div	Inhospital ECG (48 hours after arrival)?		
p_2ihecg48h	dropdown	Does Inhospital ECG Recording Exist (48 hours after arrival in hospital)?	listid: ny	Did hospital personnel record the patient ECG 48 hours after arrival in hospital

PREDICT - Hospital Variables

Variable	Type	Caption	List Options	Abstraction Instruction
			0. no<br>1. yes	0=no<br>1=yes<br><br>Indicate no or yes, whether 48 hour inhospital ECG recording exists.<br><br>Source = Patient follow up information / Hospital chart
p_2ihecg48hdt	textbox	Date		What was the date of the patient ECG recorded 48 hours after arrival in hospital?<br><br>Date value = yyyy/mm/dd<br><br>Source = Patient follow up information / Hospital chart
p_2ihecg48htm	textbox	Time		What was the time of the patient ECG recorded 48 hours after arrival in hospital?<br><br>Numerical value based on 24 hour clock<br><br>00:00:00 – hour:min:sec<br><br>If no value for seconds data available – do not do not use value 00<br><br>Source = Patient follow up information / Hospital chart
p_2ihecg48hrev	dropdown	Inhospital ECG (48 hours) Reviewed	listid: nyu<br><br>0. no<br>1. yes<br>2. unknown	Indicate whether or not the patient ECG recorded 48 hours after arrival in hospital was reviewed by the attending hospital hysician site or if the attendant annotations and QA data was generated solely by the device software.<br><br>0=no<br>1=yes<br>2=unknown<br><br>The goal is for sites to review the recordings so as to 'correct' any oversights by the software.<br>Source = Patient follow up information / Hospital chart
p_2ihecg48hd	dropdown	Inhospital ECG (48 hours) Data Exists	listid: ny<br><br>0. no<br>1. yes	Is there data available on request for the patient ECG recorded 48 hours after arrival in hospital?<br><br>0=no<br>1=yes<br><br>Source = Patient follow up information / Hospital chart

PREDICT - Hospital Variables

Variable	Type	Caption	List Options	Abstraction Instruction
p_2ihecg48hdgca	dropdown	Inhospital ECG (48 hours) Diagnosis - Computer Assisted	listid: ny<br>0. no<br>1. yes	Indicate whether or not there was computer assisted diagnosis for patient ECG recorded 48 hours after arrival in hospital.<br>0=no<br>1=yes<br>Source = Patient follow up information / Hospital chart
p_2ihecg48hdgsp	textbox	Inhospital ECG (48 hours) Diagnosis - Computer Assisted - Specify 1	listid: ny<br>0. no<br>1. yes	Please specify computer assisted diagnosis for the the patient ECG recorded 48 hours after arrival in hospital.<br>.<br>Value = text<br>Source = Patient follow up information / Hospital chart
p_2ihecg48hdgsp_2	textbox	Inhospital ECG (48 hours) Diagnosis - Computer Assisted - Specify 2 (cont)		
p_2ihecg48hdgsp_3	textbox	Inhospital ECG (48 hours) Diagnosis - Computer Assisted - Specify 3 (cont)		
p_2ihecg48hdg	textbox	Inhospital ECG (48 hours) Diagnosis 1	listid: ny<br>0. no<br>1. yes	Indicate the diagnosis based on patient ECG recorded 48 hours after arrival in hospital.<br>made by hospital attending physician.<br>Source = Patient follow up information / Hospital chart
p_2ihecg48hdg_2	textbox	Inhospital ECG (48 hours) Diagnosis 2 (cont)		
p_2ihecg48hdg_3	textbox	Inhospital ECG (48 hours) Diagnosis 3 (cont)		
p_2steihecg48h	dropdown	ST Elevation	listid: nyn<br>0. no<br>1. yes<br>2. not noted	Is there ST elevation on patient ECG recorded 48 hours after arrival in hospital?<br>0=no<br>1=yes<br>Source = Patient follow up information / Hospital chart
p_2stemicrihecg48h	dropdown	STEMI Criteria Met	listid: nynr<br>0. no<br>1. yes<br>2. not recorded	Is ST Elevation Myocardial Infarction (STEMI) criteria met on patient ECG recorded 48 hours after arrival in hospital?

PREDICT - Hospital Variables

Variable	Type	Caption	List Options	Abstraction Instruction
				0=no<br>1=yes<br>2=not noted<br>Source = Patient follow up information / Hospital chart
p_2stemilihecg48h	textbox	STEMI Location		Indicate, if possible the STEMI location based on patient ECG recorded 48 hours after arrival in hospital.<br>Source = Patient follow up information / PCI/Hospital chart
p_2ihecg48hredecg	dropdown	Reduction in the chest pain intensity	listid: nyu<br>0. no<br>1. yes<br>2. unknown	Indicate, if possible, if there is a significant reduction in the patient's chest pain intensity during the 48 hour inhospital period.<br>0=no<br>1=yes<br>2=not noted<br>Source = Patient follow up information / Hospital chart
p_2ihecg48hredstat	dropdown	Reduction in ST-Segment elevation (i.e. >50%)	listid: nyn<br>0. no<br>1. yes<br>2. not noted	Indicate, if possible, if there is a significant reduction in ST-segment elevation (i.e. > 50%) on patient ECG recorded 48 hours after arrival in hospital.<br>0=no<br>1=yes<br>2=not noted<br>Source = Patient follow up information / Hospital chart
p_2ihclinev	dropdown	Clinical Events Occurred in Hospital	listid: nyn<br>0. no<br>1. yes<br>2. not noted	Indicate if any clinical events from the list below occurred during the inhospital course of patient care.<br>List of clinical events:<br>Death<br>Cardiogenic shock<br>New of worsening CHF<br>Recurrent ischemia<br>Myocardial reinfarction<br>Suspected TIA/Stroke<br>Revascularization<br>Bleeding<br>Transfusion<br>Temporary pacemaker

Variable	Type	Caption	List Options	Abstraction Instruction
				Cardiac arrest<br>Other<br><br>Source = Patient follow up information / Hospital chart
p_2ihclinevdth	dropdown	Death	listid: ny<br><br>0. no<br>1. yes	Indicate if patient died during the PCI course of patient care.<br><br>0=no<br>1=yes<br><br>Source = Patient follow up information / PCI/Hospital chart
p_2ihclinevdthdt	textbox	Death Date		What was the date of the patient's death in hospital?<br><br>Date value = yyyy/mm/dd<br><br>Source = Patient follow up information / Hospital chart
p_2ihclinevdthtm	textbox	Death Time		What was the time of the patient's death in hospital?<br><br>Numerical value based on 24 hour clock<br><br>00:00:00 – hour:min:sec<br><br>If no value for seconds data available – do not do not use value 00<br><br>Source = Patient follow up information / Hospital chart
p_2ihclinevcs	dropdown	Cardiogenic Shock	listid: ny<br><br>0. no<br>1. yes	Indicate if patient had cardiogenic shock during the inhospital course of patient care.<br><br>0=no<br>1=yes<br><br>Source = Patient follow up information / PCI/Hospital chart
p_2ihclinevcsdt	textbox	Cardiogenic Shock Date		What was the date of the patient's cardiogenic shock i during the inhospital course of patient care?<br><br>Date value = yyyy/mm/dd<br><br>Source = Patient follow up information / Hospital chart
p_2ihclinevcstm	textbox	Cardiogenic Shock Time		What was the time of the patient's cardiogenic shock i during the inhospital course of patient care?<br><br>Numerical value based on 24 hour clock<br><br>00:00:00 – hour:min:sec

PREDICT - Hospital Variables

Variable	Type	Caption	List Options	Abstraction Instruction
				If no value for seconds data available – do not do not use value 00<br><br>Source = Patient follow up information / Hospital chart
p_2ihclinevnwchf	dropdown	New or Worsening CHF	listid: ny<br><br>0. no<br>1. yes	Indicate if patient had new or worsening congestive heart failure (CHF) during the inhospital course of patient care.<br><br>0=no<br>1=yes<br><br>Source = Patient follow up information / Hospital chart
p_2ihclinevnwchfdt	textbox	New or Worsening CHF Date		What was the date of the patient's new or worsening congestive heart failure (CHF) during the inhospital course of patient care?<br><br>Date value = yyyy/mm/dd<br><br>Source = Patient follow up information / Hospital chart
p_2ihclinevnwchftm	textbox	New or Worsening CHF Time		What was the time of the patient's new or worsening congestive heart failure (CHF) during the inhospital course of patient care?<br><br>Numerical value based on 24 hour clock<br><br>00:00:00 – hour:min:sec<br><br>If no value for seconds data available – do not do not use value 00<br><br>Source = Patient follow up information / Hospital chart
p_2ihclinevrisch	dropdown	Recurrent Ischemia	listid: ny<br><br>0. no<br>1. yes	Indicate if patient had recurrent ischemia during the inhsopital course of patient care.<br><br>0=no<br>1=yes<br><br>Source = Patient follow up information / Hospital chart
p_2ihclinevrischdt	textbox	Reccurent Ischemia Date		What was the date of the patient's recurrent ischemia during the inhospital course of patient care?<br><br>Date value = yyyy/mm/dd<br><br>Source = Patient follow up information / Hospital chart

Variable	Type	Caption	List Options	Abstraction Instruction
p_2ihclinevrischtm	textbox	Recurrent Ischemia Time		What was the time of the patient's recurrent ischemia during the inhospital course of patient care?<br><br>Numerical value based on 24 hour clock<br><br>00:00:00 – hour:min:sec<br><br>If no value for seconds data available – do not do not use value 00<br><br>Source = Patient follow up information / Hospital chart
p_2ihclinevmrein	dropdown	Myocardial Reinfarction	listid: ny<br><br>0. no<br>1. yes	Indicate if patient had myocardial reinfarction during the inhospital course of patient care.<br><br>0=no<br>1=yes<br><br>Source = Patient follow up information / Hospital chart
p_2ihclinevmreindt	textbox	Myocardial Reinfarction Date		What was the date of the patient's myocardial reinfarction during the inhospital course of patient care?<br><br>Date value = yyyy/mm/dd<br><br>Source = Patient follow up information / Hospital chart
p_2ihclinevmreintm	textbox	Myocardial Reinfarction Time		What was the time of the patient's myocardial reinfarction during the inhospital course of patient care?<br><br>Numerical value based on 24 hour clock<br><br>00:00:00 – hour:min:sec<br><br>If no value for seconds data available – do not do not use value 00<br><br>Source = Patient follow up information / Hospital chart
p_2ihclinevtiastr	dropdown	Suspected TIA/Stroke	listid: ny<br><br>0. no<br>1. yes	Indicate if patient had suspected TIA/stroke during the inhospital course of patient care.<br><br>0=no<br>1=yes<br><br>Source = Patient follow up information / Hospital chart
p_2ihclinevtiastrdt	textbox	Suspected TIA/Stroke Date		What was the date of the patient's suspected TIA/stroke during the inhospital course of patient care?

PREDICT - Hospital Variables

Variable	Type	Caption	List Options	Abstraction Instruction
				Date value = yyyy/mm/dd<br><br>Source = Patient follow up information / Hospital chart
p_2ihclinevtiastrtm	textbox	Suspected TIA/Stroke Time		What was the time of the patient's suspected TIA/stroke during the inhospital course of patient care?<br><br>Numerical value based on 24 hour clock<br><br>00:00:00 – hour:min:sec<br><br>If no value for seconds data available – do not do not use value 00<br><br>Source = Patient follow up information / Hospital chart
p_2ihclinevrev	dropdown	Revascularization	listid: ny<br><br>0. no<br>1. yes	Indicate if patient had revascularization during the inhospital course of patient care.<br><br>0=no<br>1=yes<br><br>Source = Patient follow up information / /Hospital chart
p_2ihclinevrevdt	textbox	Revascularization Date		What was the date of the patient's revascularization procedure during the inhospital course of patient care?<br><br>Date value = yyyy/mm/dd<br><br>Source = Patient follow up information / Hospital chart
p_2ihclinevrevtm	textbox	Revascularization Time		What was the time of the patient's revascularization procedure during the inhospital course of patient care?<br><br>Numerical value based on 24 hour clock<br><br>00:00:00 – hour:min:sec<br><br>If no value for seconds data available – do not do not use value 00<br><br>Source = Patient follow up information / Hospital chart
p_2ihclinevbleed	dropdown	Bleeding	listid: ny<br><br>0. no<br>1. yes	Indicate if patient had bleeding during the inhospital course of patient care.<br><br>0=no<br>1=yes<br><br>Source = Patient follow up information / Hospital chart

PREDICT - Hospital Variables

Variable	Type	Caption	List Options	Abstraction Instruction
p_2ihcline vbleeddt	textbox	Bleeding Date		What was the date of the patient's bleeding event during the inhospital course of patient care?<br><br>Date value = yyyy/mm/dd<br><br>Source = Patient follow up information / Hospital chart
p_2ihcline vbleedtm	textbox	Bleeding Time		What was the time of the patient's bleeding event during the inhospital course of patient care?<br><br>Numerical value based on 24 hour clock<br><br>00:00:00 – hour:min:sec<br><br>If no value for seconds data available – do not do not use value 00<br><br>Source = Patient follow up information / Hospital chart
p_2ihcline vtransf	dropdown	Transfusion	listid: ny<br><br>0. no<br>1. yes	Indicate if patient had transfusion during the inhospital course of patient care.<br><br>0=no<br>1=yes<br><br>Source = Patient follow up information / Hospital chart
p_2ihcline vtransfdt	textbox	Transfusion Date		What was the date of the patient's transfusion procedure during the inhospital course of patient care?<br><br>Date value = yyyy/mm/dd<br><br>Source = Patient follow up information / Hospital chart
p_2ihcline vtransftm	textbox	Transfusion Time		What was the time of the patient's transfusion procedure during the inhospital course of patient care?<br><br>Numerical value based on 24 hour clock<br><br>00:00:00 – hour:min:sec<br><br>If no value for seconds data available – do not do not use value 00<br><br>Source = Patient follow up information / Hospital chart
p_2ihcline vtempac	dropdown	Temporary Pacemaker	listid: ny<br><br>0. no<br>1. yes	Indicate if patient had temporaty pacemaker inserted during the inhospital course of patient care.<br><br>0=no

Variable	Type	Caption	List Options	Abstraction Instruction
				1=yes<br><br>Source = Patient follow up information / Hospital chart
p_2ihclinevtempacdt	textbox	Temporary Pacemaker Date		What was the date when the patient temporaty pacemaker was inserted during the inhospital course of patient care?<br><br>Date value = yyyy/mm/dd<br><br>Source = Patient follow up information / Hospital chart
p_2ihclinevtempactm	textbox	Temporary Pacemaker Time		What was the time when the patient temporaty pacemaker was inserted during the inhospital course of patient care?<br><br>Numerical value based on 24 hour clock<br><br>00:00:00 – hour:min:sec<br><br>If no value for seconds data available – do not do not use value 00<br><br>Source = Patient follow up information / Hospital chart
p_2ihclinevcararr	dropdown	Cardiac Arrest	listid: ny<br><br>0. no<br>1. yes	Indicate if patient had cardiac arrest during the inhospital course of patient care.<br><br>0=no<br>1=yes<br><br>Source = Patient follow up information / Hospital chart
p_2ihclinevcararrdt	textbox	Cardiac Arrest Date		What was the date of the patient's cardiac arrest during the inhospital course of patient care?<br><br>Date value = yyyy/mm/dd<br><br>Source = Patient follow up information / Hospital chart
p_2ihclinevcararrtm	textbox	Cardiac Arrest Time		What was the time of the patient's cardiac arrest during the inhospital course of patient care?<br><br>Numerical value based on 24 hour clock<br><br>00:00:00 – hour:min:sec<br><br>If no value for seconds data available – do not do not use value 00

PREDICT - Hospital Variables

Variable	Type	Caption	List Options	Abstraction Instruction
				Source = Patient follow up information / Hospital chart
p_2ihclinevoth	dropdown	Other	listid: ny<br><br>0. no<br>1. yes	Indicate if any other clinical events occurred during the inhospital course of patient care.<br><br>0=no<br>1=yes<br><br>Source = Patient follow up information / Hospital chart
p_2ihclinevoths	textbox	Specify		Please specify what other clinical event occurred during the inhospital course of patient care.<br><br>Source = Patient follow up information / Hospital chart
p_2ihmed	dropdown	Additional Medications Given in Hospital	listid: nyn<br><br>0. no<br>1. yes<br>2. not noted	Indicate if any additional medications from the list below were administered to the patient duing the inhospital course of care.<br><br>List of medications:<br><br>Beta blocker<br>IV vasodilator (other than nitrates)<br>ACE inhibitor<br>ARB t<br>Lipid lowering agent<br>Diuretic<br>IV inotrope<br>Oral anticoagulant (e.g. coumadin/warfarin)<br><br>Other<br><br>Source = Patient follow up information / Hospital chart
p_2ihmedbb	dropdown	Beta Blocker	listid: nyn<br><br>0. no<br>1. yes<br>2. not noted	Indicate if beta blocker was administered to the patient duing the inhospital course of care.<br><br>0=no<br>1=yes<br>2=not noted<br><br>Source = Patient follow up information / Hospital chart
p_2ihmedivvas	dropdown	IV Vasodilator (Other Than Nitrates)	listid: nyn<br><br>0. no<br>1. yes<br>2. not noted	Indicate if IV vasodilatoe (other than nitrates) was administered to the patient duing the inhospital course of care.<br><br>0=no<br>1=yes<br>2=not noted

Variable	Type	Caption	List Options	Abstraction Instruction
				Source = Patient follow up information / Hospital chart
p_2ihmedacei	dropdown	Angiotensin Converting Enzyme (ACE) Inhibitor	listid: nyn<br><br>0. no<br>1. yes<br>2. not noted	Indicate if angiotensin converting enzyme (ACE) inhibitor was administered to the patient duing the inhospital course of care.<br><br>0=no<br>1=yes<br>2=not noted<br><br>Source = Patient follow up information / Hospital chart
p_2ihmedarb	dropdown	Angiotensin Receptor Blocker (ARB)	listid: nyn<br><br>0. no<br>1. yes<br>2. not noted	Indicate if angiotensin receptor blocker (ARB) was administered to the patient duing the inhospital course of care.<br><br>0=no<br>1=yes<br>2=not noted<br><br>Source = Patient follow up information / Hospital chart
p_2ihmedlla	dropdown	Lipid Lowering Agent	listid: nyn<br><br>0. no<br>1. yes<br>2. not noted	Indicate if lipid lowering agent was administered to the patient duing the inhospital course of care.<br><br>0=no<br>1=yes<br>2=not noted<br><br>Source = Patient follow up information / Hospital chart
p_2ihmeddiu	dropdown	Diuretic	listid: nyn<br><br>0. no<br>1. yes<br>2. not noted	Indicate if diuteric was administered to the patient duing the inhospital course of care.<br><br>0=no<br>1=yes<br>2=not noted<br><br>Source = Patient follow up information / Hospital chart
p_2ihmedivino	dropdown	IV Inotrope	listid: nyn<br><br>0. no<br>1. yes<br>2. not noted	Indicate if IV inotrope was administered to the patient duing the inhospital course of care.<br><br>0=no<br>1=yes<br>2=not noted<br><br>Source = Patient follow up information / Hospital chart

PREDICT - Hospital Variables

Variable	Type	Caption	List Options	Abstraction Instruction
p_2ihmedoacoa	dropdown	Oral Anticoagulant (e.g. Coumadin/Warfarin)	listid: nyn<br><br>0. no<br>1. yes<br>2. not noted	Indicate if oral anticoagulant (e.g. coumadin/warfarin) was administered to the patient duing the inhospital course of care.<br><br>0=no<br>1=yes<br>2=not noted<br><br>Source = Patient follow up information / Hospital chart
p_2ihmedoth	dropdown	Other	listid: nyn<br><br>0. no<br>1. yes<br>2. not noted	Indicate if any other medication was administered to the patient duing the inhospital course of care.<br><br>0=no<br>1=yes<br>2=not noted<br><br>Source = Patient follow up information / Hospital chart
p_2ihmedoths	textbox	Specify		Please specify what other medication was administered to the patient duing the inhospital course of care.<br><br>Source = Patient follow up information / Hospital chart
p_2ihmhndet_ahg	dropdown	Not Determined	listid: nyn<br><br>0. no<br>1. yes<br><br>2. not noted	Patient Medication History: (from Patient follow up information / Hospital chart<br><br>The Patient follow up information / Hospital chart selection of not determined implies the inhospital staff (nurse/resident/fellow/staff physician) did not ask as opposed to not noted which implies the abstractor could not find the information.
p_2ihd	div	Hospital Disposition		
p_2ihddt	textbox	Hospital Departure Date		What was the date of the patient hospital departure?<br><br>Date value = yyyy/mm/dd<br><br>Source = Patient follow up information / Hospital chart
p_2ihdtm	textbox	Hospital Departure Time		What was the time of the patient hospital departure?<br><br>00:00:00 – hour:min:sec<br><br>Source = Patient follow up information / Hospital chart
p_2ihdhome	dropdown	Was Patient Discharged Home?	listid: ny<br><br>0. no	After admission to hospital was patient then discharged home?

Variable	Type	Caption	List Options	Abstraction Instruction
			1. yes	0=no<br>1=yes<br><br>Source = Patient follow up information / Hospital chart
p_2ihptxferah	dropdown	Was Patient Transferred to Another Hospital?	listid: ny<br><br>0. no<br>1. yes	After admission to hospital was patient then transferred to another hospital?<br><br>0=no<br>1=yes<br><br>Source = Patient follow up information / Hospital chart
p_2ihtxhosp	dropdown	Transfer Hospital Name	listid: p_hosp<br><br>** See list items in appendix	What is the name of the hospital patient was transferred to?<br><br>Source = Patient follow up information / Hospital chart<br><br>Pulldown menu = list of hospitals in database<br><br>Unknown Hospital = select when destination hospital will never be known.<br><br>NOTE;<br>Do not provide the names of nursing home, rehabilitation, or other non-acute care facilities.<br>Transfer to one of these three entities constitutes an 'hospital discharge, reclassification, or death'.
p_2ihptxferpci	dropdown	Was Patient Transferred to PCI Centre?	listid: ny<br><br>0. no<br>1. yes	After admission to hospital was patient then transferred to PCI centre?<br><br>0=no<br>1=yes<br><br>Source = Patient follow up information / Hospital chart
p_2ihtxrpci	dropdown	Transfer Hospital Name	listid: p_hosp<br><br>** See list items in appendix	What is the name of the PCI centre patient was transferred to?<br><br>Source = Patient follow up information / PCI/Hospital chart

Variable	Type	Caption	List Options	Abstraction Instruction
				Pulldown menu = list of hospitals in database<br><br>Unknown Hospital = select when destination hospital will never be known.<br><br>NOTE;<br><br>Do not provide the names of nursing home, rehabilitation, or other non-acute care facilities.<br>Transfer to one of these three entities constitutes an 'hospital discharge, reclassification, or death'.
p_2ihtrans	dropdown	Transport Mode	listid: trans<br><br>1. by land<br>2. by air	
p_2ihllsh	div	Location and Length of Stay in Hospital		
p_2ihccuicu	dropdown	CCU/ICU	listid: ny<br><br>0. no<br>1. yes	Did patient stay at CCU/ICU during the inhospital course of patient care.<br><br>0=no<br>1=yes<br><br>Source = Patient follow up information / Hospital chart
p_2ihccuicudays	textbox	CCU/ICU Days		The total number of days patient stayed at CCU/ICU during the inhospital course of patient care.<br><br>Source = Patient follow up information / Hospital chart
p_2ihtelward	dropdown	Telemetry Ward	listid: ny<br><br>0. no<br>1. yes	Did patient stay at telemetry ward during the inhospital course of patient care.<br><br>0=no<br>1=yes<br><br>Source = Patient follow up information / Hospital chart
p_2ihtelwarddays	textbox	Telemetry Ward Days		The total number of days patient stayed at telemetry ward during the inhospital course of patient care.<br><br>Source = Patient follow up information / Hospital chart

PREDICT - Hospital Variables

Variable	Type	Caption	List Options	Abstraction Instruction
p_2ihward	dropdown	Ward	listid: ny<br>0. no<br>1. yes	Did patient stay at ward during the inhospital course of patient care.<br>0=no<br>1=yes<br>Source = Patient follow up information / Hospital chart
p_2ihwarddays	textbox	Ward Days		The total number of days patient stayed at ward during the inhospital course of patient care.<br>Source = Patient follow up information / Hospital chart
p_2ihtotallos	textbox	Total Length of Stay (Total LOS) in Days		The total number of days patient stayed at the hospital.<br>Total = discharge – arrival<br>Source = Patient follow up information / Hospital chart
p_2ihicd10	div	ICD 10 Codes		
p_2ihicd10_1	textbox	1		
p_2ihicd10_2	textbox	2		
p_2ihicd10_3	textbox	3		
p_2ihicd10_4	textbox	4		
p_2ihicd10_5	textbox	5		
p_2ihicd10_6	textbox	6		
p_2ihicd10_7	textbox	7		
p_2ihicd10_8	textbox	8		
p_2ihicd10_9	textbox	9		
p_2ihicd10_10	textbox	10		
p_2ihicd10_11	textbox	11		
p_2ihicd10_12	textbox	12		
p_2ihicd10_13	textbox	13		
p_2ihicd10_14	textbox	14		
p_2ihicd10_15	textbox	15		
p_2ihicd10_16	textbox	16		
p_2ihstatus	dropdown	Inhospital form status	listid: p_status<br>0. Incomplete	

PREDICT - Hospital Variables

Variable	Type	Caption	List Options	Abstraction Instruction
			1. Complete<br>2. Pending Source Doc(s)<br>3. Not Required - Patient Deceased	

Appendix:

Hospital List

1696. Alexandra Hospital
1206. Alexandra Marine & General Hospital
2057. Blind River District Health
4418. Bluewater Health - CEEH
4415. Bluewater Health - Mitton
1006. Brant Community Healthcare - Brantford
4309. Brant Community Healthcare - Willett
1905. Cambridge Memorial Hospital
1597. Campbellford Memorial Hospital
2173. Chapleau Health Services
4238. Chatham-Kent Health Alliance
1239. Chatham-Kent Health Alliance - Sydenham
4197. Collingwood General and Marine Hospital
2174. Espanola General Hospital
1507. Four Counties Health Services
2175. Geraldton District Hospital
3734. Grand River Hospital - K-W
1030. Grey Bruce Health Services - Lion's Head
4025. Grey Bruce Health Services - Markdale
4027. Grey Bruce Health Services - Meaford
3944. Grey Bruce Health Services - Owen Sound
4030. Grey Bruce Health Services - Southhampton
4033. Grey Bruce Health Services - Wiarton
1936. Groves Memorial Community Hospital
1946. Guelph General Hospital
1146. Haldimand War Memorial Hospital
3737. Haliburton Highlands Health Services - Haliburton
4192. Halton Heathcare Services - Oakville-Trafalgar
4246. Halton Heathcare Services - Georgetown
4193. Halton Heathcare Services - Milton
1982. Hamilton Health Sciences Corporation - General
1983. Hamilton Health Sciences Corporation - Henderson
1994. Hamilton Health Sciences Corporation - McMaster
1124. Hanover & District Hospital
4161. Headwaters Health - Orangeville (Dufferin)
2061. Hornepayne Community Hospital
4260. Humber River Regional Hospital - Church
4089. Humber River Regional Hospital - Finch
1199. Huron Perth Healthcare Alliance - Clinton
1213. Huron Perth Healthcare Alliance - Seaforth
1748. Huron Perth Healthcare Alliance - St. Mary's
1754. Huron Perth Healthcare Alliance - Stratford
4142. HÃ´tel-Dieu Grace Hospital
4144. Joseph Brant Memorial Hospital
2211. Kirkland & District Hospital, Kirkland Lake
2076. Lady Dunn Health Centre
2078. Lady Minto Hospital
4171. Lakeridge Health Corporation - Oshawa
4170. Lakeridge Health Corporation - Bowmanville
4172. Lakeridge Health Corporation - Port Perry
1067. Leamington District Hospital
1740. Listowel Memorial Hospital
4310. London Health Sciences Centre - University Hospital
4247. London Health Sciences Centre - Victoria Hospital
2121. Manitoulin Health Centre - Little Current
2123. Manitoulin Health Centre - Mindemoya
2176. Manitouwadge General Hospital
4235. Markham-Stouffville Hospital Corporation
4467. Markham-Stouffville Hospital Corporation - Uxbridge
2126. Mattawa Hospital
4110. Mount Sinai Hospital
4258. Muskoka Algonquin - Huntsville District Memorial Hospital
4320. Muskoka Algonquin - South Muskoka Memorial Hospital
4210. Niagara Health System - Douglas Memorial Hospital
4213. Niagara Health System - Greater Niagara General
4219. Niagara Health System - Port Colborne General

4224. Niagara Health System - St. Catharines General
4227. Niagara Health System - Welland Hospital
2178. Nipigon District Memorial Hospital
1591. Norfolk General Hospital
3411. North Bay General Hospital
4241. Georgian Bay General Hospital (Huronia District Hospital)
4323. North Wellington Health Care - Mount Forest
4326. North Wellington Health Care - Palmerston
4233. North York General Hospital
4234. North York General Hospital - Branson
3860. Northumberland Hills Hospital
2082. Notre -Dame Hospital Hearst
4108. Orillia Soldiers' Memorial Hospital
1768. Peterborough General Hospital
2115. Red Lake Margaret Cochenour Memorial Hospital
2148. Riverside Health Care Facilities Inc . - Emo
2150. Riverside Health Care Facilities Inc . - Laverendye
2153. Riverside Health Care Facilities Inc . - Rainy River
4177. Ross Memorial Hospital
4140. Rouge Valley Health System - Ajax Pickering
4139. Rouge Valley Health System - Centennary
2064. Sault Area Hospitals - Mathews Memorial
4407. Sault Area Hospitals - S. S.Marie
2075. Sault Area Hospitals - Thessalon
2088. Sensenbrenner Hospital
4353. Sioux Lookout Meno Ya Win Health Centre
2094. Smooth Rock Falls Hospital
4042. South Bruce Grey Health Centre - Chesley
4036. South Bruce Grey Health Centre - Durham
3907. South Bruce Grey Health Centre - Kincardine
4039. South Bruce Grey Health Centre - Walkerton
1203. South Huron Hospital Alliance
4001. Southlake Regional Health
2058. St. Joseph's General Hospital
4056. St. Joseph's Health Centre
2003. St. Joseph's Healthcare
1921. St. Mary's Hospital
3985. St. Michael's Hospital
1059. St. Thomas Elgin General Hospital
4249. The Stevenson Memorial Hospital Alliston
1515. Strathroy Middlesex General Hospital
4063. Sudbury Regional Hospital - Laurentian
4069. Sudbury Regional Hospital - Memorial
4066. Sudbury Regional Hospital - St. Joseph's
4205. Sunnybrook Health Sciences Centre
2207. Temiskaming Hospital
3986. The Credit Valley Hospital
2180. The McCausland Hospital
3987. The Royal Victoria Hospital
3975. The Scarborough Hospital - General
3984. The Scarborough Hospital - Grace
3853. Thunder Bay Regional Health Sciences
1709. Tillsonburg District Memorial Hospital
3414. Timmins & District Base Hospital Program
4209. Toronto East General Hospital
4090. Trillium Health Centre
4265. University Health Network - General
4266. University Health Network - Western
1149. West Haldimand General Hospital
1538. West Lincoln Memorial Hospital
2812. West Nipissing General Hospital
4236. West Parry Sound Health Centre
4685. William Osler Health System - Brampton
4245. William Osler Health System - Etobicoke
2177. Wilson Memorial General Hospital
1079. Windsor Regional Hospital - Metropolitan
1217. Wingham & District Hospital
1716. Woodstock General Hospital Trust
4138. York Central Hospital
